# Respiratory modulation of cognitive performance during the retrieval process

**DOI:** 10.1371/journal.pone.0204021

**Published:** 2018-09-14

**Authors:** Nozomu H. Nakamura, Masaki Fukunaga, Yoshitaka Oku

**Affiliations:** 1 Division of Physiome, Department of Physiology, Hyogo College of Medicine, Mukogawa cho, Nishinomiya, Hyogo Japan; 2 Division of Cerebral Integration, Department of System Neuroscience, National Institute of Physiological Sciences, Okazaki, Aichi Japan; Tokai University, JAPAN

## Abstract

Recent research suggests that cognitive performance might be altered by the respiratory-synchronized activity generated in the brain. Previous human studies, however, have yielded inconsistent results when assessing task performance during distinct respiratory phases (inspiratory phase vs. expiratory phase). We therefore tested whether cognitive performance was regulated based on the timing of breathing components (e.g., expiratory-to-inspiratory (EI) phase transition) during the retrieval process. To determine the role of respiration in performance, the present study employed healthy subjects (n = 18) in a delayed matching-to-sample visual recognition task where a test cue was given in the respiratory phase-locked (Phased) or regularly paced (Non-phased) presentation paradigm. During the Phased session but not during the Non-phased session, the response time (RT) of the task increased by 466 ms (*p* = 0.003), and accuracy decreased by 21.4% (*p* = 0.004) when the retrieval process encompassed the EI transition. Breathing-dependent changes were particularly prominent when the EI transition occurred during the middle step of the retrieval process. Meanwhile, changes in the RT and accuracy were not observed when the retrieval process encompassed the inspiratory-to-expiratory phase transition. This is the first time that a certain phase transition in the respiratory cycle has been shown to modulate performance on a time scale of several seconds in a cognitive task. We propose that attenuation of these breathing-dependent cognitive fluctuations might be crucial for the maintenance and stability of successful performance in daily life and sports.

## Introduction

A growing body of research proposes that the act of breathing may shape and disturb cognitive function [1–5]. Breathing exercises in healthy subjects before performing a task can alter the state of the brain and improve subsequent cognitive performance and motor skills [6–9]. However, whether breathing components directly affect task performance remains unclear.

Recent findings have suggested a link between breathing and task performance [10, 11]. Respiration modulates neural oscillations (e.g., gamma oscillations) in the neocortex [12]. In particular, the phase in the rhythm of nasal respiration modulates memory performance [1] and gamma oscillation activities in the olfactory bulb, hippocampus, prefrontal cortex, and other areas with possible consequences for cognitive function [2–5, 13–15]. Meanwhile, memory performance is enhanced by the release of noradrenaline from the locus coeruleus (LC) in the prefrontal cortex [16–18] and hippocampus [19]. A recent study demonstrated that the inspiratory (I) activity generated by the brainstem [i.e., by the preBötzinger complex (preBötC)] modulates arousal and active states by means of noradrenaline release in the LC [20]. Therefore, respiratory activity could improve cognitive performance via several possible systems in the brain.

Another potential mode of breathing-dependent regulation of cognition is that a particular respiratory phase may modulate a specific phase of the retrieval process during a cognitive task. In the last half century, numerous studies using various behavioral tasks in healthy subjects have investigated whether exposing retrieval cues during the inspiratory (I) phase or the expiratory (E) phase affects performance [e.g., response time (RT) and accuracy]; however, these studies have produced inconsistent results [1, 21–24]. We think that the inconsistency between these studies is due to the measurement of the timing of the retrieval process started during the respiratory phase (I phase vs. E phase). Timing the retrieval cues (i.e., the beginning of the retrieval process) to occur between the I and E phases may be insufficient to identify an association between retrieval and breathing because the retrieval process involves multiple subprocesses/concepts (e.g., accessing what-where-when information and accessibility vs. availability) [25–27]. Rather, a possible key component affecting performance is whether the retrieval process crosses a respiratory phase point [e.g., the E-to-I (EI) or I-to-E (IE) phase transition] at which most of the respiratory neurons in the brainstem fire synchronously [28, 29].

To test this hypothesis, we sought to identify a specific point during the respiratory cycle and to determine whether this point affects performance in a cognitive task. In the present study, we designed a version of a delayed matching-to-sample (DMTS) task with a short delay [30–33]. Using an experimental paradigm in which the subjects’ respiratory-phase information was monitored and linked to the presentation of visual cues in the task (referred to hereafter as “phase-locked”), we demonstrated for the first time that respiratory phase properties differentially modulate cognitive performance during the retrieval process.

## Materials and methods

### Subjects

The participants were 18 healthy right-handed individuals (10 males and 8 females; age: 22.2 ± 0.5 years, range: 20–27 years). All had abstained from caffeine-containing beverages for at least 12 hours before the experiment. No subject received regular medication, and none had a known history of respiratory, cardiovascular, or endocrine disease. Written informed consent was obtained from all participants. All procedures performed on humans were in accordance with the Declaration of Helsinki (Ethical Principles for Medical Research Involving Human Subjects) and Ethical Guidelines for Medical and Health Research Involving Human Subjects, Japan, and were approved by the Ethics Committee of Hyogo College of Medicine, Japan (No.1825).

### Apparatus

The I and E phases during the respiratory cycle were continuously recorded via a flow sensor nasal cannula (Flow Nasal Cannula A, Atom Medical, Japan) equipped with a differential pressure transmitter (Model KL17, Nagano Keiki, Japan) [34]. The respiratory waveform and trigger signals used to present visual information for the task were sampled at 1 kHz using the PowerLab data acquisition system (PowerLab, AD Instruments, Dunedin, New Zealand) and were computed online using LabChart software (LabChart 7.1, AD Instruments). Our preliminary experiments confirmed that the respiratory data from not only nasal breathing but also mouth breathing were captured by the nasal cannula (data not shown). Participants were also monitored by infrared video camera (Panasonic, Japan) and electrocardiogram (ECG), the signals from which were recorded using a differential biological amplifier (Bioamp, AD Instruments, Sydney, Australia).

### Task paradigm

We developed a delayed matching-to-sample (DMTS) version [27, 33] of a visual recognition memory task with a short delay for humans [30, 31]. The DMTS task consisted of a study section, a delay, and a test section and followed a delayed matching-to-sample rule. Task paradigms were created by NBS Presentation® software (Presentation 18.3, Neurobehavioral Systems).

The DMTS task required the memorization and recognition of a figure (configuration), its color, its position, and the number of replicated figures shown on a computer screen (47.7 x 26.8 cm, 1920 x 1080 resolution, 60 Hz refresh rate). The shape (configuration) was a circle, triangle, rectangle, cross, crescent, or heart; the color was red, blue, green, yellow, pink, or sky-blue; the number of figures was one, two, three, four, five, or six; and the position on the screen was center, right, left, top-center, bottom-right, or bottom-left. Thus, there were 1296 (6 x 6 x 6 x 6 variables) possible combinations.

**Fig 1:**
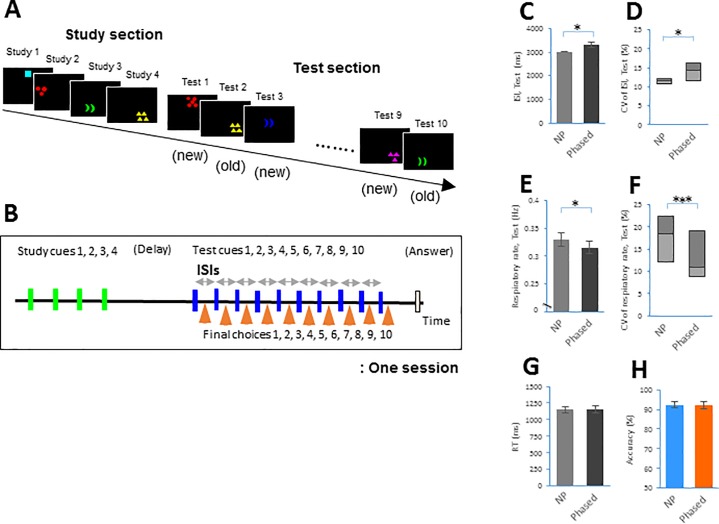
A delayed matching-to-sample (DMTS) task in the Non-phased and phased sessions. **A**. Schematic drawings showing examples of figures used during the DMTS task. **B**. The task was composed of a study section (study cues are represented by green bars), a delay, a test section (test cues are represented by blue bars), and a scoring instruction (a white bar). Each subject presses a button (final choices are represented by orange arrowheads) indicating his or her decision following an individual test cue. **C-H**. The ISI (mean ± s.e.m. in **C**) and its CV (median with quartiles in **D**); the respiratory rate (mean ± s.e.m. in **E**) and its CV (median with quartiles in **F**) during the test section; and response time (RT) and accuracy (mean ± s.e.m. in **G**, and **H**, respectively) were compared between the Non-phased (NP) and Phased sessions across individual subjects (n = 18). * *p* < 0.05 and *** *p* < 0.005 for each relevant pair.

During the study section, each subject was exposed to a series of four out of a total of 1296 figures displayed one at a time on a computer screen under dim light in a darkened room (Fig 1A and 1B). After a short delay, each subject was tested ten separate times on the ability to distinguish between the figure presented during the study section (“matching figure” or “old figure”) and a “mismatching figure” or “new figure” that was one of 1296 figures not presented during the study section (Fig 1A). The subjects had to respond after each test cue whether the presented figure was the same as one of four figures (study cues) presented during the study section and then to press the corresponding button (see Fig 1B).

A “matching figure” (or “old figure”) was defined as the figure consisting of the same four elements: shape (configuration), color, position, and number of figures presented during the study section. A “mismatching figure” (or “new figure”) was defined as a figure that did not contain more than one of the four elements presented during the study section. The subjects pressed the “Z” or “C” key on a standard keyboard using fingers on their left hand once they had identified the figure shown during the test section as a “matching figure” or “mismatching figure”, respectively. In each session, five “matching figures” and five “mismatching figures” were presented during the test section in random manner. Each subject was informed of the percentage of correct answers after the test section (i.e., scoring instruction). Before the experiments, the subjects were instructed on how to perform the task and told to maintain relaxed natural breathing using both the mouth and nose during the task. No subjects identified their own respiratory patterns before or during the experiment.

**Fig 2:**
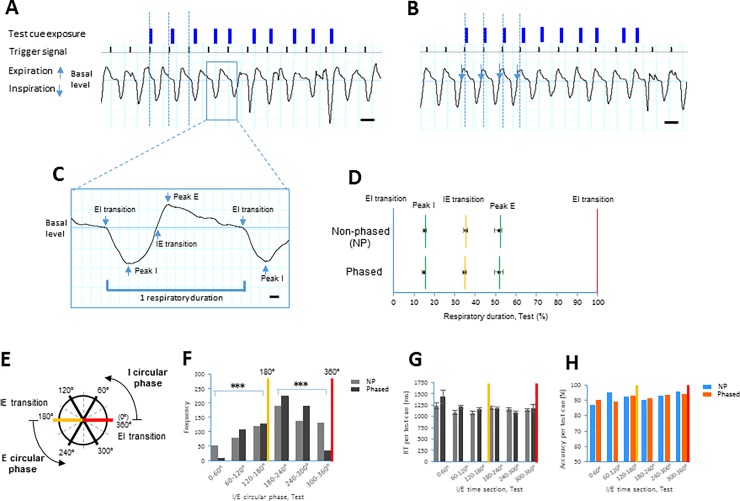
Respiratory waveforms in Non-phased vs. Phased sessions during the test section. **A.** Line graphs showing a representative raw waveform of respiration (on the lower) with timing of test cue exposure (blue bars) in the Non-phased session. After a 3-s trigger interval (vertical dotted lines), test cues are exposed with the different time lags. **B.** Line graphs showing a representative raw waveform of respiration (on the lower) with timing of test cue exposure (blue bars) in the Phased session (i.e., “IE transition setup” session). In the “IE transition setup” session, trigger signals (vertical dotted lines) are produced by the IE transition points in a filtered waveform. Consequently, trigger signals have an approximately 400-ms time lag from the IE transition points (arrows, see Methods). **C.** Higher magnification of insets in **A** showing a representative raw waveform of respiration with the EI transition, peak I, IE transition, and peak E. **D.** The timing of peak I, IE transition, and peak E from the EI transition in the raw respiratory waveform was compared between the Non-phased (NP) and Phased sessions across individual subjects (n = 18). **E**. The I/E circular phase represents the respiratory cycle in degrees: 0° represents the EI transition, 180° represents the IE transition (yellow line), and 360° represents the next EI transition (red line). **F**. Bar plots indicating the frequency of test cue exposure (y-axis) against groups in the I/E circular phase (x-axis) in the NP and Phased sessions. **G.** RT (mean ± s.e.m.) of individual test trials is compared between the Non-phased and Phased sessions (sample sizes shown in **F**) at the divided phase sections. **H.** Accuracy (mean) of individual test trials in the Non-phased vs. Phased sessions (sample sizes shown in **F**) at the divided phase sections. * *p* < 0.05 and *** *p* < 0.005 compared to relevant groups. Scale bars, 2 s in **A**, and **B**, 200 ms in **C**.

Each subject performed a total of eight sessions of the task, including four Non-phased and four Phased sessions in random order [720 responses in Non-phased sessions and 720 responses in Phased sessions, i.e., 1440 total, are the responses of all 18 subjects combined]. In Non-phased sessions, the interstimulus interval (ISI) between test cues was 3 s, with one of three different time lags (0, 300, or 600 ms after a trigger signal, in random order, see Fig 2A). In Phased sessions, test cues were set to occur at the EI or IE transition point with one of three different time lags (0, 300, or 600 ms after a trigger signal, in random order, see Fig 2B). To avoid the subject’s awareness that the timing of exposing test cues was synchronized with their respiratory phases, these different time lags in both sessions constituted the degree of variable ISIs between test cues. It was a critical point to make subjects perform the task with their respiration phase-locked to test cues without allowing them to predict the test conditions in the present study.

For the detection of the EI or IE transition point during the Phased sessions, we used a real-time monitoring method with a filtered respiratory waveform. The waveform with a low-pass (< 2Hz) filter was sampled to eliminate the effect of noise and isolate the respiratory form. Then, a trigger signal was immediately driven by the timing of the EI or IE transition point when the filtered waveform was crossing the basal level. Consequently, each trigger signal had an approximately 400-ms time lag from the EI or IE transition point in the raw waveform (Fig 2B). Thus, the four Phased sessions consisted of two “EI transition setup” and two “IE transition setup” sessions (see Fig 2B). Prior to the experiments, the subjects were not informed of the timing of the test cues during the tasks. No subjects reported awareness of the cues being locked to their own respiratory cycle, or of intentionally adjusting their breathing to correspond with the timing of cue exposure to improve their performance.

### Data analysis

During the experiments, we measured cognitive parameters–the time of cue exposure, time of button pressing, and accuracy–using NBS Presentation software, and respiratory parameters–the onset of every I and E phase, and the time of every I and E peak of nasal tidal pressure amplitude in the raw respiratory waveform (Fig 2C)–using LabChart software. At a preset level, the onset of every I and E phase was defined as the time at which the flow first crossed the basal level and shifted toward the opposite domain by over ± 2 standard deviations from baseline noise after reaching every I and E peak. Thus, the onsets of the I and E phases corresponded to the EI transition and IE transition, respectively. The series of respiratory parameters in each trial was synchronized with the series of cognitive parameters. To identify the distribution of test cues between the I and E phases, exposure timing of individual test cues was rearranged on a scale according to the I/E circular phase in degrees: 0° represented the EI transition, 180° represented the IE transition, and 360° represented the next EI transition (Fig 2E and 2F), similar to a previous study [35].

**Fig 3:**
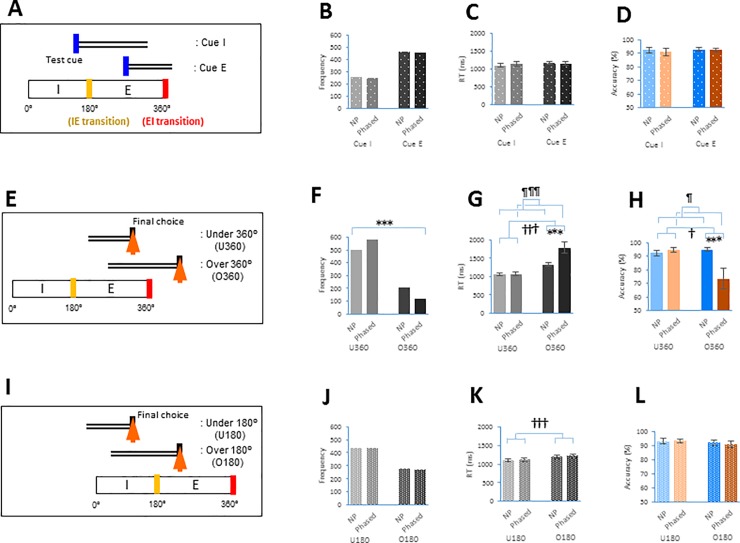
RT and accuracy of Stable vs. Phased sessions during the I and E phases, under and over 360° and under and over 180°. **A.** Schematics showing the division of individual test trials into respiratory-dependent groups. Individual test trials were divided into two groups according to the timing of cue exposure in individual test trials during the I phase and E phase, respectively (Cue I and Cue E). **B**, Bar graph showing the frequency of individual test trials (y-axis) between Non-phased (NP) and Phased sessions for the Cue I and Cue E groups. **C,D**. RT (**C**) and accuracy (**D**) (mean ± s.e.m.) were compared between the NP and Phased sessions for the Cue I and Cue E phase groups across individual subjects (Cue I-NP: n = 18, Cue I-Phased: n = 18, Cue E-NP: n = 18, Cue E-Phased: n = 18). **E**. Individual test trials were divided into those not containing the 360° point of the respiratory phase (under 360°, U360) and those containing the 360° point (over 360°, O360). **F**. Bar graphs showing the frequency of test trials (y-axis) in the U360 and O360 groups between the NP and Phased sessions. **G,H**. RT (**G**) and accuracy (**H**) (mean ± s.e.m.) were compared between the NP and Phased sessions for the U360 and O360 groups across individual subjects (U360-NP: n = 18, U360-Phased: n = 18, O360-NP: n = 18, O360-Phased: n = 17). **I**. Individual test trials were divided according to whether they did not (under 180°, U180) or did contain the 180° point (over 180°, O180). **J**. Bar graphs showing the frequency of test trials (y-axis) between the NP and Phased sessions in the U180 and O180 groups. **K,L**. RT (**K**) and accuracy (**L**) (mean ± s.e.m.) were compared between the NP and Phased sessions in the U180 and O180 groups across individual subjects (U180-NP: n = 18, U180-Phased: n = 18, O180-NP: n = 18, O180-Phased: n = 18). ¶ *p*< 0.05 and ¶¶¶ *p*< 0.005 indicate the significant main effect of session (NP vs. Phased) by two-way repeated-measures ANOVA. † *p* < 0.05 and ††† *p* < 0.005 indicate the significant main effect of transition (U360 vs. O360 or U180 vs. O180). *** *p* < 0.005 compared to relevant groups.

**Fig 4:**
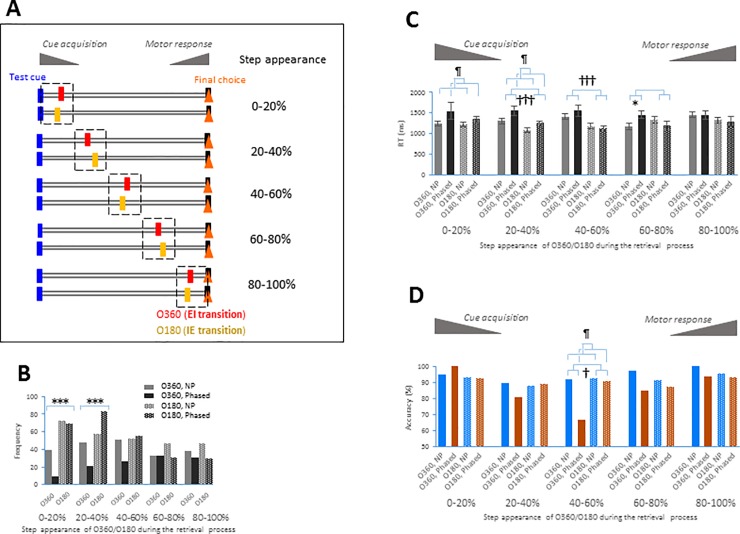
RT and accuracy in the step appearance of the 360° and 180° points of the respiratory cycle during the retrieval process. **A.** Schematics showing the five steps (the 0–20%, 20–40%, 40–60%, and 80–100% step groups) of test trials encompassing the 360° point (O360, solid bars) and test trials encompassing the 180° point (O180, hashed bars). **B.** Bar graphs showing the frequency of O360 (solid bars), and O180 (hashed bars) test trials (y-axis) in Non-phased (NP, light-gray bars) and Phased (dark-gray bars) sessions against the five steps (x-axis, the 0–20%, 20–40%, 40–60%, 60–80%, and 80–100% step groups). **C,D**. RT (mean ± s.e.m. shown in **C**) and accuracy (mean shown in **D**) were compared between the O360 and O180 groups in the NP and Phased sessions (sample sizes shown in **B**). ¶ *p* < 0.05 indicates the significant main effect of session (NP vs. Phased) by two-way repeated-measures ANOVA. † *p* < 0.05 and ††† *p* < 0.005 indicate the significant main effect of transition (O360 vs. O180). * *p* < 0.05 and *** *p* < 0.005 compared to relevant groups.

A test trial or a retrieval process was defined as the period from the exposure of a test cue to the pressing of a button (a final choice). The RT was calculated by the time scale between test cue exposure and final choice. To determine the effect of respiratory phases on performance, we prepared three copies of datasets of test trials: i) one dataset was divided into two groups, according to the timing of test cue exposure during the I and E phases, as the Cue I and Cue E groups, respectively (Fig 3A); ii) another dataset was divided into the following two groups: those that did not contain the EI transition point (i.e., those that stayed under 360°: referred to as under 360° or U360, hereafter, Fig 3E) and those that contained the EI transition point (i.e., those that passed through the 360° phase point: referred to as over 360° or O360, hereafter, Fig 3E); and iii) the other dataset was divided into the following two groups: those that did not contain the IE transition point (i.e., those that stayed under 180°: referred to as under 180° or U180, hereafter, Fig 3I) and those that contained the IE transition point (i.e., those that passed through the 180° phase point: referred to as over 180° or O180, hereafter, Fig 3I). Regarding the step appearance analysis, the RT was normalized as 1, and divided into five steps (the 0–20%, 20–40%, 40–60%, 60–80%, and 80–100%). Then, we categorized O360 and O180 test trials into five step groups based on the step at which the EI or IE transition appeared (Fig 4A).

In each session, fewer than 2% of test trials were excluded for the following reasons: i) quick changes between the I and E phases resulting in missing stimulus cues during the task, or ii) the extended duration of the retrieval process (greater than 360°) complicated the identification of the respiratory phase-dependent effect on cognitive performance. These criteria eliminated 25 out of 1440 test trials. All relevant data supporting the conclusion of the study are available from the authors without restriction.

### Statistical analysis

Across individual subjects, the two-tailed paired *t*-test was used to assess the significance of differences between relevant pairs based on ISI, respiratory rate, RT, and accuracy. Two-way repeated-measures ANOVA was used to evaluate the significance of the main effects and interactions in RT and accuracy during the Non-phased vs. Phased sessions and in the Cue I vs. Cue E groups, the U360 vs. O360 groups, or the U180 vs. O180 groups. The Wilcoxon signed-rank test was conducted to evaluate the CVs of the ISI and the respiratory rates between relevant pairs.

For comparisons of individual test events, Pearson’s *Χ*^*2*^ test with Yates’ continuity correction and Bonferroni adjustment was used to identify quantitative differences in the frequency distributions of test trials between relevant groups. Two-tailed unpaired *t*-test was used to evaluate the significant differences in RT of test trials between relevant pairs. Two-way ANOVA was also used to assess the significant main effects and their interactions with the RT in the test trials. Fisher exact test was also used to evaluate the significance of differences in accuracy of test trials consulting of “false” or “true” (0 or 1) between relevant pairs. Logistic regression was used to model the relationship between the binary dependent variable (0 or 1), which was used to represent the accuracy of each test trial, and the nominal independent variables (the Non-phased vs. Phased sessions and the O360 vs. O180 groups). The likelihood ratio test (an analysis of the deviance with a type II test using the *Χ*^*2*^ distribution) was conducted to estimate significant differences in deviance between a logistic regression model containing factors (i.e., session and transition) and a model not containing these factors. All statistical analyses were performed using R version 3.3.1 software (R Core Team, R Foundation for Statistical Computing, Vienna, Austria, 2016, https://www.R-project.org/).

## Results

### DMTS task in the Non-phased vs. Phased sessions

We hypothesized that the effect of the respiratory phases on task performance would depend on task difficulty. Because a task with a variable interstimulus interval (ISI) between test cues is typically more difficult than a task with a stable ISI [36, 37], we prepared tasks with two types of ISIs: a Non-phased session with the regularly paced presentation of test cues and a Phased session with the respiratory phase-locked presentation of test cues, both of which randomly had three different time lags (see Methods). We first asked healthy subjects (n = 18) to perform the DMTS task with a short delay (Fig 1A and 1B). Each session of the task consisted of a study section and a test section. Each subject performed eight sessions, with four Non-phased and four Phased sessions presented in random order. We found that the Phased sessions had longer ISIs and higher variability (i.e., coefficient of variation or CV) than the Non-phased sessions during the test section [ISI: *t*(17) = 2.60, *p* = 0.02, paired *t*-test; CV: *p* = 0.02, Wilcoxon signed-rank test; Fig 1C and 1D, see also Table in S1 Table], indicating that Phased sessions resulted in a longer and more variable ISI than did Non-phased sessions. The subjects exhibited a lower respiratory rate and a less variable respiratory rhythm during the Phased sessions than during the Non-phased sessions [respiratory rate: *t*(17) = 2.71, *p* = 0.01; CV: *p* = 0.002; Fig 1E and 1F, see also Table in S2 Table]. Nevertheless, there was no difference in RT or accuracy [RT: *t*(17) = 0.26, n.s., accuracy: *t*(17) = 0.16, n.s., Fig 1G and 1H].

### Non-phased vs. Phased sessions during I and E phases, under and over 360° and under and over 180°

We next examined whether the RT and accuracy during the task were regulated at a certain phase point in the respiratory cycle, which was measured by the nasal cannula. To determine respiratory parameters during the test section between the Non-phased (Fig 2A and 2C) and Phased (Fig 2B) sessions, we calculated the timings of the peak I, IE transition and peak E from the EI transition in the raw respiratory waveforms. No difference in the timing of the peak I, IE transition, or peak E during the respiratory cycle between the Non-phased and Phased sessions was observed [peak I: *t*(17) = 1.38, n.s.; IE transition: *t*(17) = 1.76, n.s.; peak E: *t*(17) = 0.31, n.s., paired *t*-test, Fig 2D, see also Tables in S3 Table and S4 Table].

To identify the distributions of timing of test cues in the respiratory cycle, we also expressed the “I/E circular phase” in degrees based on the convention from a previous study [35]: 0° represented the EI transition, 180°°° represented the IE transition, and 360° represented the next EI transition (Fig 2E). The I phase corresponded to 0–180°, and the E phase corresponded to 180–360°. The respiratory phases between 0° and 180° and between 180° and 360° were based on the time scale. Then, we plotted the timing of test cue exposure against the I/E circular phase in the Non-phased and Phased sessions (Fig 2F). Test cues were found to be differentially distributed between the Non-phased and Phase sessions in the I phase (0–60°, 60–120°, and 120–180°) and E phase (180–240°, 240–300°, and 300–360°) [I phase: *Χ*^*2*^(2) = 35.0, *p* < 0.00001, E phase: *Χ*^*2*^(2) = 69.2, *p* < 0.00001, Fig 2F, see also Tables in S5 Table and S6 Table]. However, there were no differences in the RT (unpaired *t*-test) and accuracy (Fisher exact test) between Non-phased and Phased sessions at each phase section (Fig 2G and 2H).

A “test trial” or “retrieval process” was defined as the period from test cue exposure to final choice. We focused on the test trials in relation to the timings of the EI transition (360°) and IE transition (180°) for further analysis. We divided individual test trials into two groups according to the timing of test cue exposure during the I and E phases, as the Cue I and Cue E groups, respectively (Fig 3A). There was no difference in the distribution of the Cue I and Cue E groups between the Non-phased and Phased sessions [*Χ*^*2*^(1) = 0.00, n.s., Pearson’s *Χ*^*2*^ test with Yates’ continuity correction, Fig 3B], indicating that the distribution of exposing test cues was similar between the Non-phased and Phased sessions in the I and E phases. We next averaged each subject’s RT and accuracy for each group. RT was calculated by the time scale between test cue exposure and final choice. Two-way repeated-measures ANOVA did not reveal a difference in RT or accuracy between the Non-phased and Phased sessions or between the Cue I and Cue E phase groups [RT: session: *F*(1, 51) = 0.25, n.s.; phase: *F*(1, 51) = 2.32, n.s.; session x phase interaction: *F*(1, 51) = 1.22, n.s., Fig 3C; accuracy: session: *F*(1, 51) = 0.19, n.s.; phase: *F*(1, 51) = 0.28, n.s., session x phase interaction: *F*(1, 51) = 0.11, n.s., Fig 3D, see also Tables in S5 Table and S6 Table].

Next, we focused on two respiratory phase transitions: the EI transition (360°) and the IE transition (180°) in the respiratory cycle. We prepared another dataset by dividing individual test trials into two groups: those that did not contain the EI transition point (i.e., those that stayed under 360°: U360, Fig 3E) and those that contained the EI transition point (i.e., those that passed through the 360° phase point: over 360° or O360, Fig 3E). We found that the U360 and O360 groups were differentially distributed between the Non-phased and Phase sessions [*Χ*^*2*^(1) = 28.2, *p* < 0.00001; Fig 3F]. Then, the RT and accuracy of individual test trials were averaged per subject for each group. Two-way repeated-measures ANOVA showed that Phased session trials had a higher average RT than trials of the Non-phased session, and the O360 group had a longer RT than the U360 transition group [session: *F*(1, 50) = 9.08, *p* = 0.004; transition: *F*(1, 50) = 43.3, *p* <0.00001; Fig 3G]. The Phased session was also characterized by lower accuracy than the Non-phased session, and the O360 group exhibited lower accuracy than the U360 group [session: *F*(1, 50) = 5.40, *p* = 0.02; transition: *F*(1, 50) = 5.03, *p* = 0.03; Fig 3H]. Interestingly, the O360 group in the Phased session exhibited a longer RT (an increase by 466 ms) and lower accuracy (a decrease by 21.4%) than the O360 group in the Non-phased session [RT: session x transition interaction: *F*(1, 50) = 9.49, *p* = 0.003; accuracy: session x transition interaction: *F*(1, 50) = 8.89, *p* = 0.004; Fig 3G and 3H, see also Tables in S5 Table and S6 Table].

To determine the effect of the IE transition (180°), we prepared the other dataset in which we divided the test trials into two groups: those that did not contain the IE transition point (i.e., those that stayed under 180°: U180, Fig 3I) and those that contained the IE transition point (i.e., those that passed through the 180° phase point: over 180° or O180, Fig 3I). There was no difference in the distribution of the U180 and O180 groups between the Non-phased and Phased sessions [*Χ*^*2*^(1) = 0.02, n.s., Fig 3J]. Next, the RT and accuracy of the test trials were averaged per subject in each group. We observed no differences in RT or accuracy between the Non-phased and Phased sessions, but the O180 group had a longer RT than the U180 group [RT: session: *F*(1, 51) = 0.42, n.s.; transition: *F*(1, 51) = 11.59, *p* = 0.001; session x transition interaction: *F*(1, 51) = 0.00, n.s. Fig 3K; accuracy: session: *F*(1, 51) = 0.05, n.s.; transition: *F*(1, 51) = 1.04, n.s.; session x transition interaction: *F*(1, 51) = 0.25, n.s.; Fig 3L, see also Tables in S5 Table and S6 Table]. These results revealed that retrieval processing containing the EI transition, but not containing the IE transition, was characterized by increased RT and decreased accuracy in the Phased sessions compared to that in the Non-phased sessions.

In addition, we further divided the three datasets (i.e., the Cue I and Cue E, the U360 and O360, and the U180 and O180 groups) into the following categories and conducted additional analyses for the frequency, RT, and accuracy: i) three different time lags (0, 300 ms, and 600 ms) during the test section (see Figure in S1 Fig); and ii) the early and late sequences with no time lag (0 ms) during the test section (see Figure in S2 Fig). These results showed that the datasets rearranged with the same time lags of test cues, where the variability of ISIs was reduced, had a similar tendency as the EI transition effect on the task.

### Step appearance of the 360° and 180° points during the retrieval process

Although we found that the accuracy of test trials containing the EI transition decreased, whether this effect can be attributed to cognitive functions is unclear. To further characterize the EI transition-dependent effect, we generated datasets containing individual O360 test trials and individual O180 test trials (but neither U360 nor U180 test trials) obtained during Non-phased and Phased sessions for step appearance analysis: Individual O360 and O180 test trials had the value of RT, which was calculated by the time between test cue exposure and final choice. RT was normalized as 1, and divided into five steps (0–20%, 20–40%, 40–60%, 60–80%, and 80–100%). We categorized O360 and O180 test trials into five step groups based on the step at which the EI or IE transition appeared (Fig 4A). Then, we evaluated each step group for a direct comparison of the EI transition-dependent vs. IE transition-dependent effects. Before the analysis, we confirmed that the data samples corresponding to individual test trials were independent for each step group. It is reasonable to assume that the 0–20% step group may reflect a process of visual cue acquisition, whereas the 80–100% step group may reflect the motor response. We found that the distribution of step groups differed at the initial steps (0–40%) between the Non-phased and Phase sessions [0–20%: *Χ*^*2*^(1) = 12.25, *p* = 0.002; 20–40%: *Χ*^*2*^(1) = 13.86, *p* = 0.001, Pearson’s *Χ*^*2*^ test with Yates’ continuity correction and Bonferroni adjustments], whereas no difference was observed for the 40–60%, 60–80%, or 80–100% step group (Fig 4A). The RT in each step group was estimated using two-way ANOVA, and the accuracy in each step group was calculated using the likelihood ratio test with logistic regression (see Methods). A comparison between the O360 and O180 groups revealed that the Phased sessions had a longer RT than the Non-phased sessions in the 0–20% and 20–40% step groups [0–20%: *F*(1, 185) = 4.62, *p* = 0.03; 20–40%: *F*(1, 206) = 4.63, *p* = 0.03, two-way ANOVA; Fig 4B]. The O360 group had a longer RT than the O180 group in the 20–40% and 40–60% step groups [20–40%: *F*(1, 206) = 12.27, *p* = 0.0006; 40–60%: *F*(1, 181) = 16.52, *p* = 0.00007; Fig 4C]. In the 60–80% step group, we observed a significant interaction of the O180-O360 transition and Non-phased-Phased session [*F*(1, 140) = 4.99, *p* = 0.03; Fig 4C]. More interestingly, in the 40–60% step group, the Phased session exhibited lower accuracy (a decrease by 25.5%) than the Non-phased session, and the O360 group exhibited lower accuracy than the O180 group [session: *Χ*^*2*^(1) = 5.23, *p* = 0.02; transition: *Χ*^*2*^(1) = 4.37, *p* = 0.04; session x transition interaction: *Χ*^*2*^(1) = 2.71, *p* < 0.1, likelihood ratio test with logistic regression; Fig 4D, see also Tables in S5 Table and S6 Table]. These analyses reveal that the increase in RT and the decrease in accuracy occurred specifically when the EI transition (360°) in the respiratory cycle appeared in the middle step (40–60%) of the retrieval process.

## Discussion

The present study demonstrated that healthy subjects performing a DMTS task with the respiratory phase-locked presentation of test cues (Phased session) exhibited increased RT and reduced accuracy when their retrieval processes encompassed the EI transition. Meanwhile, neither the RT nor the accuracy changed when the retrieval process encompassed the IE transition. In particular, accuracy was markedly decreased when the EI transition appeared in the middle step (40–60%) of the retrieval process. This is the first demonstration that a certain phase transition in the respiratory cycle modulates performance in a cognitive task.

### Respiration, ISI, RT and accuracy

Our findings show that the task with the respiratory phase-locked presentation (Phased session) had resulted in a longer ISI and more variable ISI than the task with the regularly paced presentation (Non-phased session). A previous study showed that RT did not differ between tasks with a longer ISI and shorter ISI [37]. Thus, we initially believed that the more variable ISIs in the task elicited the higher difficulty, increase in RT, and decrease in accuracy. In fact, we showed that the use of less variable ISIs (Non-phased session) did not elicit these changes, possibly because the use of stable or less variable ISIs resulted in predictable timing of cue exposure [37]. However, our additional results showed that the same time-lag trials between the respirator phase and test cue (see Figures in S1 Fig and S2 Fig) had a similar tendency of the EI transition-dependent performance decrease in the task. Since these datasets for the same time-lag trials reduced the variability of ISIs in the task, it is unlikely that the EI transition-dependent effect was the consequence of variable ISIs that were introduced independent of the respiratory phase.

We observed that RT, calculated by the time scale, was slightly longer when the retrieval process contained the IE or EI transition. It is possible that RT might simply vary over respiratory phases or that the respiratory phases that occurs close to the IE and EI transitions may have a longer RT. Moreover, the distribution of test events during the respiratory cycle was skewed. We suggested at least two possible explanations for why the test cue presentation showed an uneven distribution: i) the datasets of 18 subjects with different inspiratory and expiratory durations were summated, and ii) each respiratory pattern in individual subjects might be modulated differentially by the test cue presentation. However, the importance of the relationship between respiration and task performance is that the appearance of the EI transition decreased accuracy when subjects performed a task with the respiratory phase-locked presentation. The decrease in accuracy corresponded to an increase in RT and a decrease in the frequency of test events. In general, a longer RT reduces performance errors in the task, an effect known as the speed-accuracy tradeoff (e.g., [38, 39]). We found that the conventional tradeoff between RT and accuracy disappeared at a certain point in the respiratory cycle. Therefore, it is reasonable to conclude that the retrieval process does not function properly in the face of EI transition-dependent modulation.

### Respiration and motor response

Although respiratory modulation may affect a motor-response process, we observed no change in RT or accuracy in the last step (80–100%) of the retrieval process even though this step contained the EI transition. Notably, the last step might represent the effect of the motor response process. Previous studies have shown that breathing is associated with manipulative motor controls [40, 41]. In particular, the respiratory phase and motor manipulation exhibit a strong phase-dependent interaction [24, 42, 43]. This phase-dependent interaction has been suggested to reflect the task difficulty of a motor response, resulting in an extended RT in reaction time tasks [24]. In the present study, we found that subjects seemed to avoid passing through 360° during the task with the respiratory phase-locked presentation by accelerating their button-pressing time or by extending their E duration (Fig 3F); the adjustment of either the respiratory phase or motor response may be affected by a phase-locked interaction during the retrieval process.

### Respiration and the retrieval process

Which components of the retrieval process are associated with respiratory-dependent changes in RT and accuracy remain unclear. The retrieval process consists of multiple subprocesses/concepts [25–27] and is affected by various factors, including the encoding process [33, 44], the timing of cue exposure (i.e., stable vs. variable ISIs) [36, 37], and motor responses (pressing a button), that may affect performance during the retrieval process in a complex manner. Despite this, the respiratory-dependent decrease in accuracy was robust and occurred when the EI transition appeared in the middle step (40–60%) of the retrieval process. We suggest that the EI transition-dependent decrease in accuracy might be associated primarily with cognitive processes rather than motor responses. In any case, further work is necessary to clarity the relationship between respiratory-dependent changes in accuracy and cognitive processes.

### Potential neural mechanisms

Recent findings could lead to potential neural mechanisms of a breathing-dependent cognitive decline. Respiration modulates gamma oscillations in the hippocampus, prefrontal cortex, and other areas in awake animals [3–5, 12–15] and humans [2]. This effect depends on respiration-locked oscillations in the olfactory bulb not on brainstem-dependent activity [13, 14]. Another human study showed that the effect of respiration on memory depends on nasal respiration [1]. Thus, the effect of respiration on cortical oscillatory activity and cognitive function is suggested to be driven by respiration-olfactory bulb-locked activity [10, 11]. It will be fascinating to see whether the EI transition-dependent effect on cognitive performance only occurs during nasal breathing. However, unfortunately, the present study could not reliably estimate which test cues were presented during nasal vs. oral breathing from nasal respiratory flow monitoring.

Importantly, during behavioral and cognitive states such as exploration [4] and escape behavior [5] in animals, respiration-coupled gamma oscillations was strongly observed in the prefrontal cortex. It is tempting to predict that the EI transition-dependent cognitive dysfunction could be caused by the disturbance of respiration and gamma oscillation coupling.

Meanwhile, the timing of the I phase-dependent or EI transition-dependent activation in neurons in the olfactory bulb and somatosensory cortex was essential not only for sniffing and whisking [35, 45–47] but also for performance in odor discrimination tasks [45]. The phasic activity occurring at the EI transition might be generated by preBötC neurons, which are the primary inspiratory rhythm generator localized in the ventrolateral medulla [28, 29]. Thus, there is a possibility that EI transition-dependent changes in RT and accuracy could rely on the timing of activation of the preBötC through nasal respiration. Moreover, a recent study demonstrated that the preBötC has efferent projections to the LC, and while ablation of these projections leaves breathing intact, it decreases the time spent in attentional and active states [20] in a manner that could involve prefrontal regulation [16, 18, 48]. The neural mechanism underlying breathing-dependent changes in performance is a subject for future studies.

## Conclusions

We have demonstrated that the EI transition in the respiratory cycle modulates RT and accuracy during the retrieval process. We propose that these respiratory-dependent changes might result in short-term regulation of cognitive and motor processes on a time scale of several seconds. Thus, cancellation of these breathing-dependent cognitive fluctuations could be crucial for the maintenance and stability of successful performance. This respiration-related property might be applied to improve task performance in daily life and sports and could explain the benefit of the breathing exercises utilized in meditation and yoga practices [49]. This modulation might also contribute to a better understanding of processes such as in cognitive impairment.

## Supporting information

S1 Fig RT and accuracy of three different time-lag trials during the test section. (PDF)

S2 Fig RT and accuracy of no time-lag trial in the early and late sequences. (PDF)

S1 Table Individual ISIs during the test section. (PDF)

S2 Table Individual respiratory durations during the test section. (PDF)

S3 Table Timing of peak I, IE transition, and peak E from EI transition during the test section in the Non-phased sessions. (PDF)

S4 Table Timing of peak I, IE transition, and peak E from EI transition during the test section in the phased sessions. (PDF)

S5 Table Individual test trials in the Non-phased sessions. (PDF)

S6 Table Individual test trials in the phased sessions. (PDF)
