# Supplementary material for: Respiratory modulation of cognitive performance during the retrieval process
Source: PLoS One. 2018 Sep 14;13(9):e0204021. doi: 10.1371/journal.pone.0204021 (PMC6138381; doi:10.1371/journal.pone.0204021)
Supplement: S1 Fig — (PDF) [file pone.0204021.s001.pdf]

0 time lag

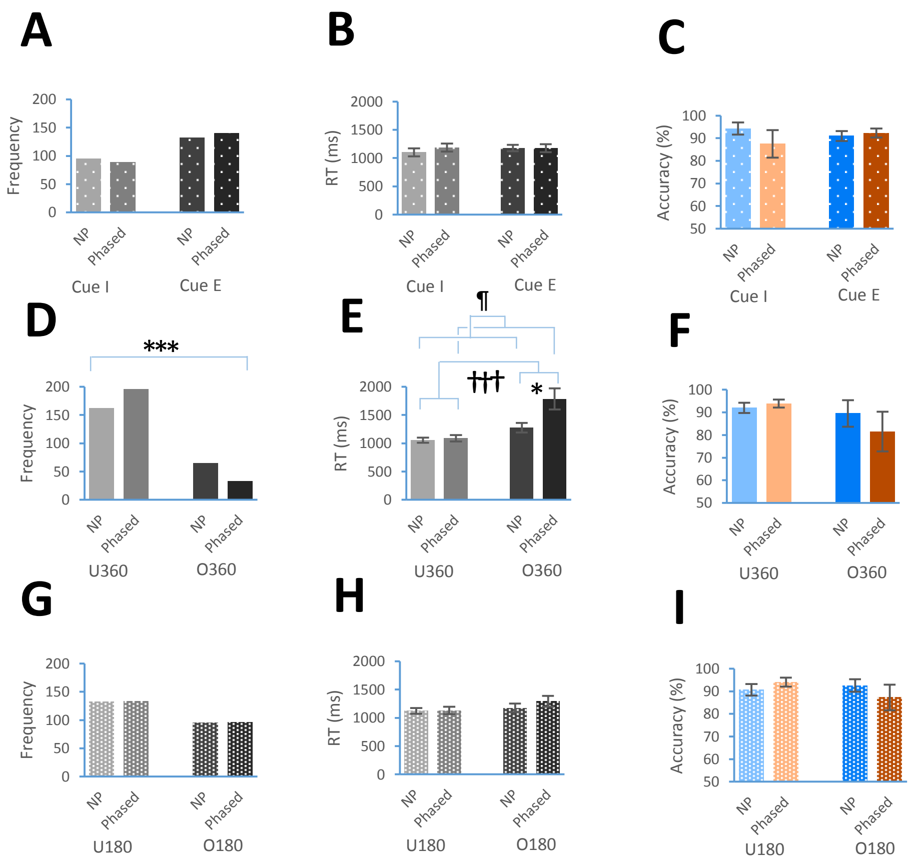

300-ms time lag

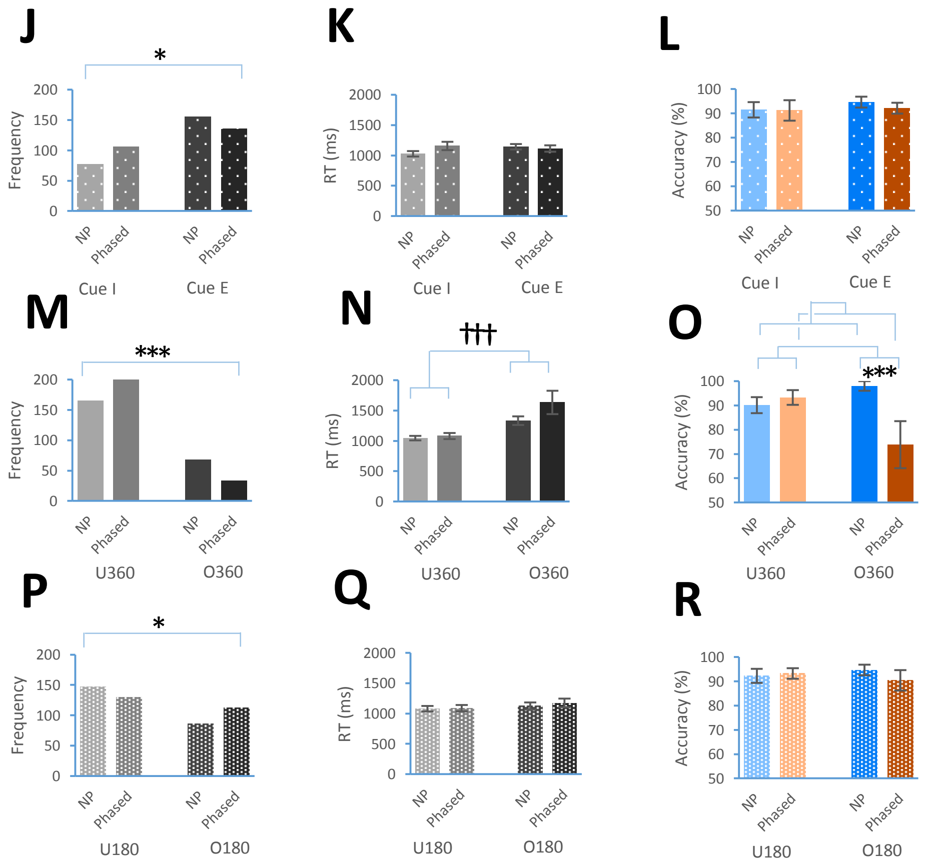

600-ms time lag

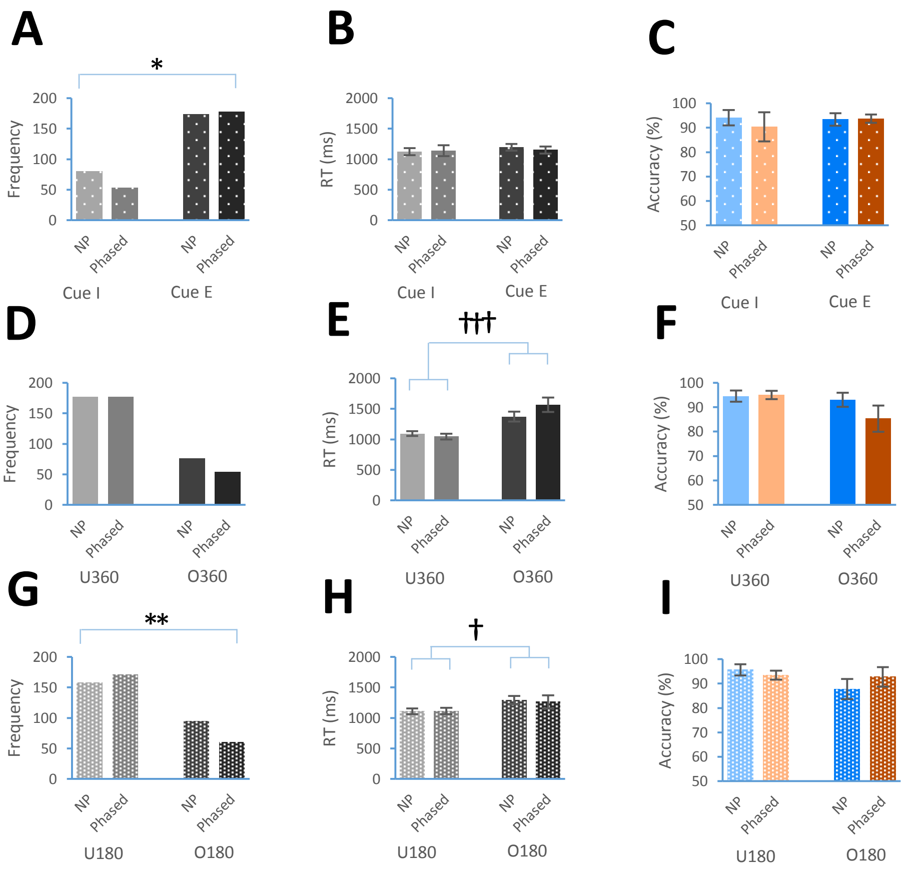

S1 Fig. RT and accuracy of three different time-lag trials during the test section

In no time-lag trial (0 time lag) during the test section, we found that the U360 and O360 groups were differentially distributed between the Non-phased and Phase sessions [$X^2(1) = 12.84$, $p = 0.0003$; S1D Fig]. Two-way repeated-measures ANOVA showed that in S1E Fig, the Phased session trials had a higher average RT than trials of the Non-phased session, and the O360 group had a longer RT than the U360 transition group [session: $F(1, 61) = 4.87$, $p = 0.03$; transition: $F(1, 61) = 21.7$, $p = 0.00002$]. Furthermore, the O360 group in the Phased session exhibited a longer RT than the O360 group in the Non-phased session [RT: session x transition interaction: $F(1, 61) = 6.54$, $p = 0.01$; S1E Fig].

In 300-ms time-lag trial during the test section, we found that the three datasets were differentially distributed between the Non-phased and Phased sessions [Cue I vs. Cue E: $X^2(1) = 5.35$, $p = 0.02$; U360 vs. O360: $X^2(1) = 15.24$, $p = 0.00009$; U180 vs. O180: $X^2(1) = 3.91$, $p = 0.05$; S1J,M,P Fig]. Two-way repeated-measures ANOVA showed that the O360 group had a longer RT than the U360 transition group [transition: $F(1, 59) = 24.5$, $p < 0.00001$; S1N Fig]. There was a significant interaction of session x transition with accuracy in S1O Fig [session x transition interaction: $F(1, 59) = 9.78$, $p = 0.003$].

In 600-ms time-lag trial during the test section, we found differential distributions between the Non-phased and Phase sessions [Cue I vs. Cue E: $X^2(1) = 4.14$, $p = 0.04$; U180 vs. O180: $X^2(1) = 6.91$, $p = 0.009$; S1A,G Fig]. Two-way repeated-measures ANOVA showed that the O360 group had a longer RT than the U360 transition group [transition: $F(1, 64) = 28.4$, $p < 0.00001$; S1E Fig] and the O180 group had a longer RT than the U180 transition group [transition: $F(1, 66) = 5.42$, $p = 0.02$; S1H Fig].
