# Supplementary material for: Respiratory modulation of cognitive performance during the retrieval process
Source: PLoS One. 2018 Sep 14;13(9):e0204021. doi: 10.1371/journal.pone.0204021 (PMC6138381; doi:10.1371/journal.pone.0204021)
Supplement: S2 Fig — (PDF) [file pone.0204021.s002.pdf]

0 time lag during Tests 1-5

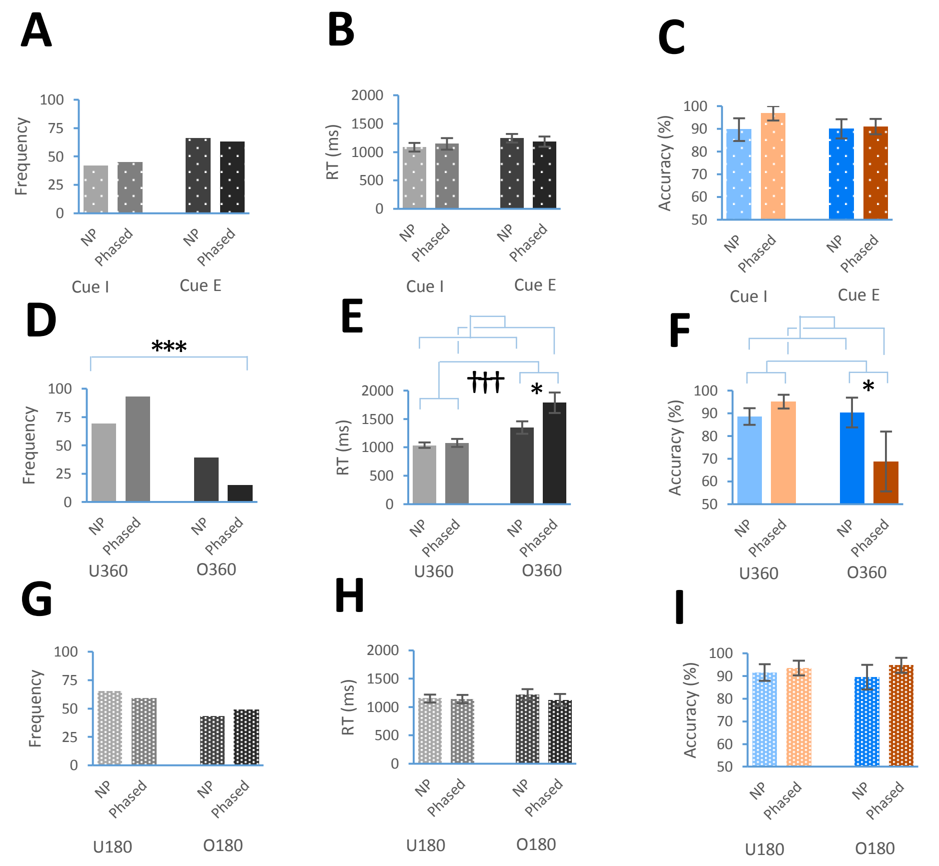

0 time lag during Tests 6-10

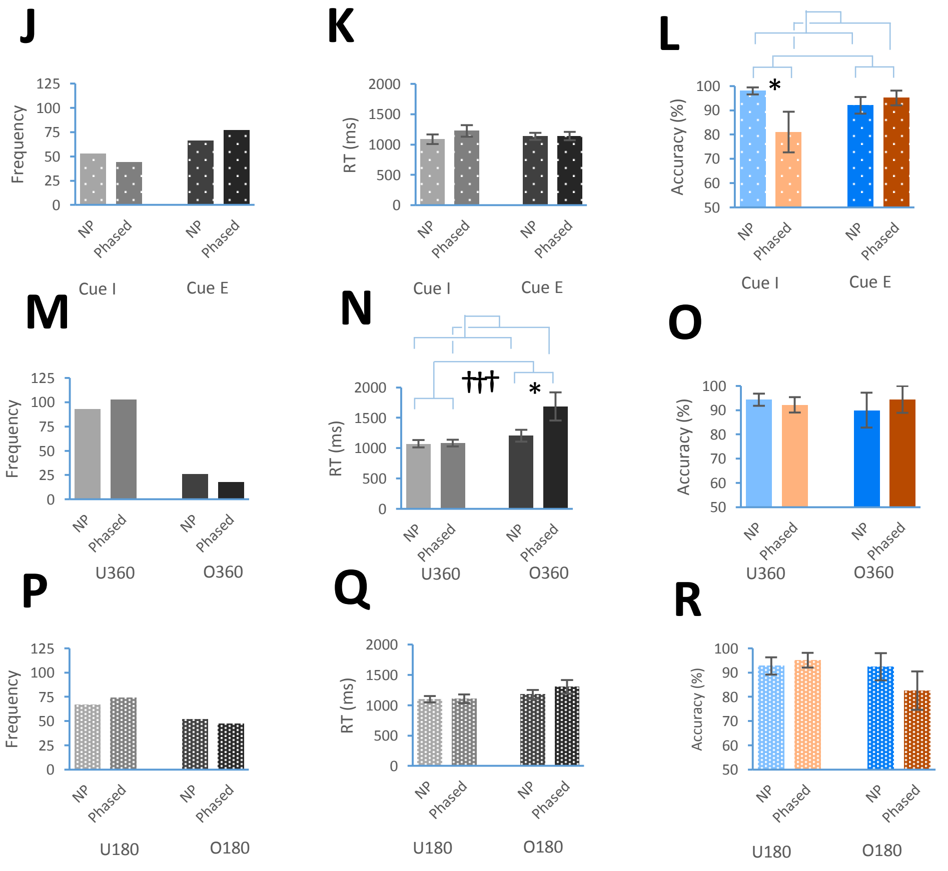

S2 Fig. RT and accuracy of no time-lag trial in the early and late sequences

In the early sequences (Tests 1-5) with 0 time lags during the test section, we found that the U360 and O360 groups were differentially distributed between the Non-phased and Phase sessions [$X^2(1) = 13.1$, $p = 0.0003$; S2D Fig]. Two-way repeated-measures ANOVA showed that the O360 group had a longer RT than the U360 transition group [$F(1, 55) = 24.4$, $p < 0.00001$]. The O360 group in the Phased session exhibited a longer RT than the O360 group in the Non-phased session [session x transition interaction: $F(1, 55) = 4.19$, $p = 0.05$, S2E Fig]. Moreover, there was a significant interaction of session x transition with accuracy in S2F Fig [session x transition interaction: $F(1, 55) = 5.48$, $p = 0.02$].

In the late sequences (Tests 6-10) with 0 time lags during the test section, two-way repeated-measures ANOVA showed that the O360 group had a longer RT than the U360 transition group [$F(1, 55) = 11.3$, $p = 0.001$]. The O360 group in the Phased session exhibited a longer RT than the O360 group in the Non-phased session [session x transition interaction: $F(1, 55) = 5.81$, $p = 0.02$, S2N Fig]. Moreover, there was a significant interaction of session x transition with accuracy in S2L Fig [session x transition interaction: $F(1, 66) = 4.49$, $p = 0.04$].
