# Supplementary material for: Respiratory modulation of cognitive performance during the retrieval process
Source: PLoS One. 2018 Sep 14;13(9):e0204021. doi: 10.1371/journal.pone.0204021 (PMC6138381; doi:10.1371/journal.pone.0204021)
Supplement: S1 Table — (PDF) [file pone.0204021.s003.pdf]

Table S1. Individual ISIs during the test section

name	session	ISI (ms)
Non-phased session		
18	1S	3283
18	1S	3316
18	1S	2416
18	1S	3583
18	1S	3000
18	1S	3000
18	1S	2416
18	1S	3017
18	1S	3283
18	2S	3283
18	2S	3316
18	2S	3000
18	2S	3000
18	2S	2700
18	2S	3000
18	2S	3016
18	2S	3300
18	2S	2700
18	5S	2716
18	5S	3583
18	5S	2700
18	5S	3300
18	5S	2716
18	5S	3300
18	5S	2700
18	5S	3300
18	5S	2416
18	8S	2700
18	8S	2716
18	8S	3017
18	8S	3016
18	8S	3566
18	8S	2417
18	8S	3016
18	8S	3566
18	8S	2417

name	session	ISI (ms)
Non-phased session		
19	1S	2716
19	1S	3283
19	1S	3016
19	1S	2716
19	1S	3283
19	1S	3000
19	1S	2717
19	1S	3016
19	1S	3583
19	2S	2716
19	2S	3016
19	2S	3266
19	2S	3300
19	2S	3000
19	2S	3016
19	2S	2700
19	2S	2716
19	2S	3583
19	5S	3016
19	5S	3283
19	5S	2716
19	5S	3583
19	5S	2417
19	5S	3283
19	5S	2717
19	5S	3600
19	5S	3000
19	8S	2716
19	8S	3016
19	8S	3016
19	8S	3266
19	8S	3300
19	8S	2700
19	8S	2716
19	8S	3600
19	8S	2700

name	session	ISI (ms)
Non-phased session		
20	1S	3283
20	1S	3316
20	1S	3000
20	1S	2417
20	1S	3583
20	1S	2700
20	1S	3316
20	1S	3000
20	1S	3000
20	4S	2700
20	4S	3316
20	4S	2417
20	4S	3283
20	4S	2717
20	4S	3016
20	4S	3016
20	4S	3566
20	4S	3000
20	7S	3283
20	7S	3000
20	7S	3300
20	7S	2700
20	7S	3317
20	7S	2416
20	7S	3017
20	7S	3566
20	7S	2700
20	8S	2716
20	8S	3017
20	8S	3016
20	8S	3566
20	8S	3000
20	8S	3000
20	8S	2700
20	8S	3000
20	8S	3316

name	session	ISI (ms)
Non-phased session		
21	1S	2417
21	1S	3016
21	1S	3583
21	1S	3000
21	1S	2700
21	1S	3300
21	1S	2716
21	1S	3000
21	1S	3300
21	4S	2417
21	4S	3016
21	4S	3283
21	4S	3000
21	4S	2716
21	4S	3016
21	4S	3566
21	4S	2416
21	4S	3016
21	7S	2716
21	7S	3300
21	7S	2700
21	7S	3300
21	7S	3000
21	7S	2716
21	7S	2716
21	7S	3016
21	7S	3266
21	8S	3000
21	8S	2716
21	8S	3583
21	8S	3000
21	8S	2716
21	8S	3300
21	8S	3000
21	8S	3000
21	8S	3000

name	session	ISI (ms)
Non-phased session		
22	1S	3300
22	1S	3000
22	1S	3000
22	1S	3000
22	1S	2416
22	1S	3600
22	1S	2417
22	1S	3583
22	1S	2417
22	4S	3000
22	4S	2416
22	4S	3283
22	4S	2716
22	4S	3600
22	4S	2700
22	4S	3000
22	4S	3300
22	4S	3000
22	7S	3583
22	7S	2700
22	7S	3016
22	7S	3300
22	7S	3000
22	7S	3000
22	7S	2416
22	7S	3016
22	7S	3016
22	8S	3000
22	8S	2700
22	8S	3016
22	8S	3300
22	8S	2417
22	8S	3016
22	8S	3566
22	8S	3016
22	8S	3000

name	session	ISI (ms)
Non-phased session		
23	1S	3000
23	1S	3000
23	1S	3000
23	1S	3000
23	1S	2716
23	1S	3016
23	1S	3583
23	1S	2416
23	1S	3583
23	2S	2716
23	2S	3600
23	2S	2700
23	2S	3300
23	2S	2700
23	2S	2716
23	2S	3300
23	2S	2716
23	2S	3283
23	5S	2416
23	5S	3283
23	5S	3000
23	5S	3316
23	5S	3000
23	5S	3000
23	5S	2416
23	5S	3016
23	5S	3583
23	8S	2716
23	8S	3583
23	8S	2416
23	8S	3016
23	8S	3016
23	8S	3566
23	8S	3000
23	8S	2416
23	8S	3600

name	session	ISI (ms)
Non-phased session		
24	1S	3000
24	1S	3016
24	1S	2716
24	1S	3583
24	1S	2417
24	1S	3583
24	1S	2700
24	1S	3316
24	1S	2416
24	2S	3000
24	2S	3300
24	2S	3000
24	2S	2700
24	2S	3316
24	2S	2417
24	2S	3283
24	2S	3300
24	2S	2417
24	5S	3000
24	5S	2700
24	5S	3300
24	5S	3016
24	5S	2700
24	5S	2716
24	5S	3283
24	5S	3000
24	5S	3000
24	8S	2716
24	8S	3016
24	8S	3266
24	8S	2716
24	8S	3600
24	8S	3000
24	8S	2700
24	8S	3300
24	8S	2416

name	session	ISI (ms)
Non-phased session		
26	1S	3583
26	1S	2700
26	1S	2717
26	1S	3300
26	1S	2716
26	1S	3016
26	1S	3266
26	1S	2716
26	1S	3016
26	4S	2416
26	4S	3300
26	4S	3300
26	4S	2417
26	4S	3283
26	4S	3016
26	4S	2716
26	4S	3016
26	4S	3266
26	7S	2700
26	7S	3300
26	7S	2417
26	7S	3600
26	7S	2700
26	7S	3000
26	7S	3000
26	7S	2717
26	7S	3283
26	8S	2416
26	8S	3016
26	8S	3583
26	8S	3000
26	8S	2700
26	8S	3000
26	8S	3300
26	8S	2417
26	8S	3600

name	session	ISI (ms)
Non-phased session		
27	1S	3283
27	1S	2716
27	1S	3016
27	1S	3583
27	1S	3000
27	1S	3000
27	1S	2417
27	1S	3016
27	1S	3583
27	3S	2416
27	3S	3300
27	3S	3000
27	3S	3300
27	3S	2417
27	3S	3016
27	3S	3266
27	3S	2717
27	3S	3600
27	5S	2417
27	5S	3016
27	5S	3266
27	5S	3016
27	5S	2716
27	5S	3016
27	5S	3566
27	5S	2416
27	5S	3016
27	8S	3000
27	8S	2417
27	8S	3583
27	8S	2416
27	8S	3600
27	8S	2416
27	8S	3583
27	8S	2417
27	8S	3016

name	session	ISI (ms)
Non-phased session		
28	1S	3583
28	1S	3000
28	1S	3000
28	1S	2716
28	1S	3300
28	1S	2700
28	1S	3000
28	1S	3000
28	1S	3316
28	3S	3300
28	3S	2700
28	3S	3300
28	3S	3016
28	3S	3000
28	3S	2416
28	3S	3283
28	3S	2716
28	3S	3583
28	5S	3000
28	5S	2700
28	5S	3300
28	5S	2416
28	5S	3600
28	5S	3000
28	5S	3000
28	5S	3000
28	5S	2417
28	8S	3316
28	8S	2416
28	8S	3016
28	8S	3266
28	8S	3000
28	8S	2716
28	8S	3600
28	8S	2700
28	8S	3000

name	session	ISI (ms)
Non-phased session		
29	1S	3016
29	1S	3283
29	1S	3300
29	1S	2700
29	1S	2716
29	1S	3016
29	1S	3016
29	1S	3566
29	1S	3000
29	4S	3300
29	4S	2417
29	4S	3600
29	4S	2416
29	4S	3016
29	4S	3266
29	4S	3316
29	4S	2700
29	4S	3000
29	7S	3016
29	7S	2716
29	7S	3283
29	7S	3000
29	7S	3000
29	7S	3316
29	7S	2416
29	7S	3016
29	7S	3016
29	8S	2716
29	8S	3000
29	8S	3000
29	8S	3300
29	8S	2416
29	8S	3600
29	8S	2700
29	8S	2717
29	8S	3016

name	session	ISI (ms)
Non-phased session		
30	1S	3600
30	1S	2416
30	1S	3016
30	1S	3566
30	1S	3016
30	1S	3000
30	1S	2700
30	1S	2716
30	1S	3283
30	2S	3300
30	2S	3300
30	2S	2417
30	2S	3016
30	2S	3566
30	2S	2417
30	2S	3300
30	2S	3000
30	2S	2716
30	6S	3000
30	6S	3000
30	6S	2417
30	6S	3300
30	6S	3300
30	6S	2700
30	6S	2716
30	6S	3300
30	6S	3300
30	8S	3016
30	8S	2716
30	8S	3016
30	8S	3016
30	8S	3250
30	8S	3316
30	8S	2416
30	8S	3583
30	8S	3000

name	session	ISI (ms)
Non-phased session		
31	1S	2716
31	1S	2716
31	1S	3283
31	1S	3000
31	1S	3300
31	1S	3000
31	1S	2416
31	1S	3600
31	1S	3000
31	4S	2716
31	4S	3583
31	4S	3000
31	4S	2716
31	4S	2716
31	4S	3283
31	4S	2716
31	4S	3583
31	4S	3000
31	6S	3600
31	6S	2700
31	6S	3000
31	6S	3300
31	6S	3000
31	6S	2416
31	6S	3016
31	6S	3016
31	6S	3566
31	8S	2716
31	8S	3600
31	8S	2700
31	8S	3300
31	8S	2416
31	8S	3283
31	8S	3016
31	8S	3000
31	8S	3000

name	session	ISI (ms)
Non-phased session		
32	1S	3300
32	1S	3000
32	1S	3000
32	1S	2417
32	1S	3016
32	1S	3017
32	1S	3566
32	1S	2417
32	1S	3283
32	3S	2716
32	3S	3300
32	3S	3300
32	3S	2416
32	3S	3017
32	3S	3583
32	3S	2700
32	3S	3000
32	3S	3300
32	6S	3000
32	6S	2717
32	6S	3600
32	6S	3000
32	6S	2417
32	6S	3283
32	6S	3300
32	6S	3016
32	6S	3000
32	8S	3600
32	8S	2700
32	8S	3000
32	8S	3000
32	8S	3000
32	8S	3000
32	8S	2716
32	8S	3300
32	8S	3300

name	session	ISI (ms)
Non-phased session		
33	1S	3600
33	1S	2417
33	1S	3583
33	1S	2700
33	1S	3016
33	1S	3000
33	1S	3300
33	1S	2700
33	1S	2716
33	6S	3000
33	6S	3000
33	6S	2416
33	6S	3583
33	6S	2416
33	6S	3300
33	6S	3300
33	6S	2700
33	6S	3300
33	7S	3016
33	7S	3566
33	7S	3000
33	7S	2700
33	7S	3016
33	7S	2716
33	7S	3583
33	7S	2700
33	7S	2716
33	8S	3000
33	8S	2716
33	8S	3600
33	8S	3000
33	8S	3000
33	8S	2700
33	8S	3316
33	8S	2700
33	8S	3300

name	session	ISI (ms)
Non-phased session		
34	1S	3000
34	1S	2716
34	1S	3300
34	1S	2417
34	1S	3016
34	1S	3266
34	1S	3016
34	1S	3000
34	1S	3000
34	3S	3000
34	3S	2700
34	3S	2716
34	3S	3016
34	3S	3016
34	3S	3016
34	3S	3250
34	3S	3000
34	3S	3016
34	6S	2716
34	6S	3600
34	6S	2700
34	6S	2716
34	6S	3283
34	6S	3000
34	6S	3016
34	6S	2716
34	6S	3583
34	8S	3000
34	8S	2700
34	8S	3016
34	8S	3300
34	8S	2417
34	8S	3583
34	8S	3000
34	8S	3016
34	8S	3000

name	session	ISI (ms)
Non-phased session		
35	1S	2716
35	1S	3583
35	1S	2417
35	1S	3300
35	1S	2716
35	1S	3016
35	1S	3266
35	1S	3316
35	1S	2700
35	3S	3016
35	3S	3300
35	3S	2700
35	3S	3000
35	3S	3000
35	3S	3316
35	3S	2417
35	3S	3583
35	3S	2416
35	6S	2416
35	6S	3300
35	6S	2716
35	6S	3583
35	6S	2417
35	6S	3016
35	6S	3583
35	6S	3000
35	6S	2700
35	8S	3600
35	8S	2416
35	8S	3283
35	8S	3000
35	8S	2716
35	8S	3600
35	8S	3000
35	8S	2416
35	8S	3583

name	session	ISI (ms)
Non-phased session		
36	1S	3300
36	1S	3000
36	1S	2716
36	1S	3000
36	1S	2716
36	1S	3283
36	1S	3000
36	1S	3016
36	1S	3300
36	2S	3300
36	2S	2700
36	2S	2716
36	2S	3016
36	2S	3283
36	2S	3300
36	2S	2700
36	2S	3000
36	2S	2716
36	3S	3016
36	3S	3283
36	3S	2716
36	3S	3016
36	3S	3266
36	3S	2716
36	3S	3300
36	3S	3000
36	3S	3000
36	8S	2416
36	8S	3283
36	8S	3000
36	8S	2716
36	8S	3600
36	8S	2417
36	8S	3283
36	8S	3000
36	8S	3000

name	session	ISI (ms)
Phased session		
18	3E	3883
18	3E	3900
18	3E	4500
18	3E	3000
18	3E	3016
18	3E	3016
18	3E	2417
18	3E	2417
18	3E	3016
18	4I	3016
18	4I	3017
18	4I	2716
18	4I	2716
18	4I	3017
18	4I	3016
18	4I	2416
18	4I	2717
18	4I	
18	6I	2800
18	6I	3600
18	6I	2500
18	6I	2716
18	6I	3017
18	6I	3066
18	6I	2600
18	6I	3000
18	6I	2716
18	7E	3016
18	7E	3017
18	7E	3850
18	7E	2416
18	7E	3016
18	7E	2417
18	7E	2417
18	7E	2416
18	7E	3017

name	session	ISI (ms)
Phased session		
19	3I	3500
19	3I	3300
19	3I	2416
19	3I	2717
19	3I	2716
19	3I	2716
19	3I	3017
19	3I	2416
19	3I	3016
19	4E	2800
19	4E	2716
19	4E	2417
19	4E	3016
19	4E	2416
19	4E	2417
19	4E	2816
19	4E	2417
19	4E	3583
19	6E	2416
19	6E	3783
19	6E	3000
19	6E	3900
19	6E	4100
19	6E	3800
19	6E	3600
19	6E	4300
19	6E	3300
19	7I	4000
19	7I	2716
19	7I	3183
19	7I	3900
19	7I	3100
19	7I	3200
19	7I	2900
19	7I	2800
19	7I	2717

name	session	ISI (ms)
Phased session		
20	2E	3016
20	2E	3016
20	2E	3017
20	2E	2716
20	2E	2817
20	2E	2900
20	2E	2717
20	2E	3483
20	2E	2500
20	3I	3300
20	3I	3200
20	3I	2900
20	3I	3100
20	3I	2800
20	3I	3200
20	3I	2800
20	3I	3300
20	3I	3100
20	5I	3000
20	5I	3000
20	5I	3000
20	5I	2900
20	5I	3600
20	5I	3200
20	5I	3700
20	5I	3200
20	5I	2716
20	6E	4700
20	6E	3500
20	6E	3100
20	6E	4200
20	6E	3800
20	6E	4100
20	6E	3100
20	6E	2900
20	6E	3500

name	session	ISI (ms)
Phased session		
21	2I	3016
21	2I	3016
21	2I	2416
21	2I	3016
21	2I	3016
21	2I	3016
21	2I	
21	2I	
21	2I	2716
21	3E	2500
21	3E	2416
21	3E	2716
21	3E	3016
21	3E	3016
21	3E	2416
21	3E	2716
21	3E	2716
21	3E	
21	5E	2416
21	5E	3016
21	5E	2716
21	5E	2716
21	5E	2716
21	5E	2716
21	5E	3016
21	5E	
21	5E	
21	6I	3016
21	6I	2417
21	6I	3016
21	6I	2716
21	6I	2717
21	6I	2416
21	6I	
21	6I	
21	6I	2716

name	session	ISI (ms)
Phased session		
22	2E	2800
22	2E	3216
22	2E	2716
22	2E	3016
22	2E	3016
22	2E	3016
22	2E	3033
22	2E	2700
22	2E	2600
22	3I	2716
22	3I	2416
22	3I	3016
22	3I	3016
22	3I	2416
22	3I	3016
22	3I	2716
22	3I	3016
22	3I	3016
22	5I	2600
22	5I	2416
22	5I	2716
22	5I	2417
22	5I	2716
22	5I	2716
22	5I	3016
22	5I	3016
22	5I	2416
22	6E	2500
22	6E	3016
22	6E	2983
22	6E	3000
22	6E	2800
22	6E	2716
22	6E	3016
22	6E	2716
22	6E	3016

name	session	ISI (ms)
Phased session		
23	3E	3200
23	3E	3200
23	3E	2700
23	3E	3016
23	3E	3583
23	3E	3000
23	3E	3000
23	3E	3400
23	3E	2600
23	4I	3000
23	4I	3100
23	4I	2900
23	4I	3300
23	4I	2900
23	4I	2716
23	4I	3016
23	4I	3166
23	4I	3000
23	6I	2416
23	6I	3016
23	6I	2966
23	6I	2700
23	6I	2716
23	6I	2783
23	6I	2600
23	6I	3000
23	6I	2800
23	7E	3700
23	7E	3400
23	7E	3300
23	7E	3300
23	7E	2600
23	7E	3200
23	7E	3016
23	7E	3183
23	7E	2800

name	session	ISI (ms)
Phased session		
24	3I	4500
24	3I	3600
24	3I	3400
24	3I	4100
24	3I	2600
24	3I	4800
24	3I	3600
24	3I	5200
24	3I	5200
24	4E	4500
24	4E	3300
24	4E	3400
24	4E	3700
24	4E	4900
24	4E	3900
24	4E	4800
24	4E	3500
24	4E	4000
24	6E	4000
24	6E	4800
24	6E	3600
24	6E	3200
24	6E	3400
24	6E	4800
24	6E	3400
24	6E	4700
24	6E	7199
24	7I	3900
24	7I	3100
24	7I	4500
24	7I	4000
24	7I	4200
24	7I	3600
24	7I	4400
24	7I	3500
24	7I	3900

name	session	ISI (ms)
Phased session		
26	2E	3500
26	2E	2416
26	2E	3016
26	2E	2716
26	2E	2417
26	2E	2716
26	2E	2716
26	2E	3600
26	2E	2416
26	3I	3100
26	3I	2500
26	3I	2900
26	3I	3200
26	3I	2716
26	3I	3016
26	3I	3016
26	3I	3016
26	3I	2416
26	5I	2900
26	5I	2500
26	5I	2716
26	5I	3016
26	5I	3016
26	5I	2716
26	5I	2733
26	5I	2700
26	5I	2417
26	6E	2900
26	6E	2900
26	6E	3600
26	6E	2417
26	6E	3016
26	6E	3566
26	6E	3300
26	6E	3200
26	6E	4300

name	session	ISI (ms)
Phased session		
27	2I	3016
27	2I	3016
27	2I	2866
27	2I	3300
27	2I	2600
27	2I	2716
27	2I	3183
27	2I	2417
27	2I	3016
27	4E	2800
27	4E	3200
27	4E	2800
27	4E	3600
27	4E	2900
27	4E	3000
27	4E	2800
27	4E	3016
27	4E	3283
27	6E	3000
27	6E	3600
27	6E	3100
27	6E	3700
27	6E	3016
27	6E	3283
27	6E	3000
27	6E	3100
27	6E	3700
27	7I	3800
27	7I	3700
27	7I	3200
27	7I	2900
27	7I	3800
27	7I	3700
27	7I	3300
27	7I	3700
27	7I	2800

name	session	ISI (ms)
Phased session		
28	2E	2800
28	2E	2716
28	2E	3016
28	2E	2716
28	2E	3016
28	2E	3016
28	2E	2416
28	2E	2416
28	2E	2883
28	4I	3383
28	4I	3000
28	4I	3100
28	4I	2600
28	4I	2800
28	4I	2900
28	4I	3000
28	4I	2416
28	4I	3016
28	6I	2416
28	6I	2717
28	6I	3016
28	6I	2716
28	6I	3116
28	6I	2800
28	6I	4200
28	6I	2900
28	6I	3100
28	7E	3300
28	7E	2800
28	7E	3200
28	7E	3416
28	7E	2800
28	7E	3500
28	7E	3100
28	7E	3400
28	7E	3200

name	session	ISI (ms)
Phased session		
29	2I	2800
29	2I	2716
29	2I	3016
29	2I	2717
29	2I	2716
29	2I	2416
29	2I	2716
29	2I	3016
29	2I	3783
29	3E	4000
29	3E	4300
29	3E	3300
29	3E	3500
29	3E	3900
29	3E	3100
29	3E	2716
29	3E	2783
29	3E	2417
29	5E	3800
29	5E	2700
29	5E	2716
29	5E	3016
29	5E	2716
29	5E	2716
29	5E	3333
29	5E	2600
29	5E	3400
29	6I	3100
29	6I	3400
29	6I	3700
29	6I	3400
29	6I	3100
29	6I	3016
29	6I	3683
29	6I	3100
29	6I	2417

name	session	ISI (ms)
Phased session		
30	3I	3300
30	3I	4300
30	3I	3500
30	3I	5500
30	3I	3800
30	3I	4700
30	3I	4500
30	3I	4600
30	3I	5000
30	4E	4500
30	4E	3100
30	4E	4700
30	4E	2900
30	4E	4500
30	4E	5300
30	4E	4400
30	4E	6799
30	4E	3200
30	5E	6199
30	5E	4100
30	5E	4200
30	5E	4000
30	5E	4416
30	5E	5600
30	5E	4700
30	5E	4400
30	5E	4700
30	7I	4500
30	7I	4500
30	7I	4400
30	7I	2716
30	7I	3583
30	7I	5200
30	7I	4900
30	7I	5300
30	7I	5100

name	session	ISI (ms)
Phased session		
31	2I	3900
31	2I	3800
31	2I	4300
31	2I	3700
31	2I	4600
31	2I	3400
31	2I	4400
31	2I	4200
31	2I	3700
31	3E	5500
31	3E	5200
31	3E	4700
31	3E	4600
31	3E	5200
31	3E	3300
31	3E	4100
31	3E	4400
31	3E	3900
31	5I	3500
31	5I	4200
31	5I	4600
31	5I	3400
31	5I	3100
31	5I	4100
31	5I	3800
31	5I	3400
31	5I	3200
31	7E	4900
31	7E	4700
31	7E	4500
31	7E	4100
31	7E	3800
31	7E	4500
31	7E	3900
31	7E	4100
31	7E	4700

name	session	ISI (ms)
Phased session		
32	2E	3600
32	2E	3700
32	2E	3500
32	2E	4000
32	2E	3900
32	2E	3500
32	2E	4000
32	2E	3100
32	2E	4100
32	4I	3200
32	4I	3300
32	4I	2417
32	4I	3283
32	4I	3016
32	4I	3017
32	4I	2716
32	4I	3450
32	4I	3700
32	5E	3583
32	5E	3500
32	5E	3200
32	5E	3017
32	5E	4083
32	5E	8299
32	5E	10099
32	5E	4400
32	5E	3000
32	7I	2417
32	7I	4983
32	7I	3500
32	7I	3300
32	7I	2717
32	7I	3383
32	7I	2717
32	7I	3083
32	7I	5300

name	session	ISI (ms)
Phased session		
33	2I	3200
33	2I	5199
33	2I	3000
33	2I	3300
33	2I	3016
33	2I	3016
33	2I	3166
33	2I	2500
33	2I	3300
33	3E	3900
33	3E	3600
33	3E	3000
33	3E	4200
33	3E	3600
33	3E	3800
33	3E	3400
33	3E	3600
33	3E	2700
33	4I	3000
33	4I	3900
33	4I	3600
33	4I	3600
33	4I	3300
33	4I	3100
33	4I	2716
33	4I	3016
33	4I	3016
33	5E	3300
33	5E	4000
33	5E	3400
33	5E	2900
33	5E	3800
33	5E	3100
33	5E	3100
33	5E	3400
33	5E	4300

name	session	ISI (ms)
Phased session		
34	2I	3100
34	2I	3100
34	2I	3200
34	2I	2416
34	2I	3483
34	2I	2900
34	2I	3200
34	2I	3300
34	2I	3100
34	4E	3800
34	4E	3100
34	4E	3500
34	4E	2800
34	4E	3500
34	4E	3500
34	4E	3600
34	4E	4100
34	4E	3900
34	5I	3300
34	5I	3800
34	5I	3000
34	5I	3100
34	5I	3100
34	5I	3300
34	5I	2716
34	5I	3016
34	5I	3016
34	7E	3300
34	7E	3500
34	7E	2800
34	7E	3700
34	7E	3100
34	7E	3100
34	7E	3600
34	7E	2700
34	7E	4000

name	session	ISI (ms)
Phased session		
35	2E	3600
35	2E	3000
35	2E	2716
35	2E	3183
35	2E	3600
35	2E	3200
35	2E	3100
35	2E	2417
35	2E	3283
35	4I	2716
35	4I	3016
35	4I	3016
35	4I	2716
35	4I	2833
35	4I	2700
35	4I	2416
35	4I	2717
35	4I	2983
35	5E	3283
35	5E	3100
35	5E	3800
35	5E	3400
35	5E	2900
35	5E	3300
35	5E	2900
35	5E	3016
35	5E	3083
35	7I	2800
35	7I	2716
35	7I	3483
35	7I	3000
35	7I	2900
35	7I	3300
35	7I	2800
35	7I	2600
35	7I	2716

name	session	ISI (ms)
Phased session		
36	4E	3200
36	4E	3800
36	4E	2416
36	4E	2783
36	4E	3016
36	4E	3683
36	4E	3200
36	4E	3300
36	4E	3600
36	5I	3700
36	5I	3600
36	5I	3300
36	5I	3500
36	5I	2900
36	5I	3900
36	5I	3700
36	5I	2716
36	5I	2716
36	6I	4000
36	6I	3700
36	6I	3200
36	6I	3500
36	6I	3600
36	6I	3500
36	6I	2900
36	6I	3300
36	6I	3500
36	7E	4300
36	7E	3500
36	7E	3000
36	7E	3800
36	7E	3100
36	7E	3800
36	7E	4200
36	7E	2417
36	7E	3016
