# Supplementary material for: Respiratory modulation of cognitive performance during the retrieval process
Source: PLoS One. 2018 Sep 14;13(9):e0204021. doi: 10.1371/journal.pone.0204021 (PMC6138381; doi:10.1371/journal.pone.0204021)
Supplement: S2 Table — (PDF) [file pone.0204021.s004.pdf]

**Table S2. Individual respiratory durations during the test section**

| name, session | respiratory duration (ms) | | | | | | | | | | | | | |
|---|---|---|---|---|---|---|---|---|---|---|---|---|---|---|
| Non-phased session | | | | | | | | | | | | | | |
| 18-1S | 1374 | 1973 | 2216 | 2286 | 2417 | 2587 | 2924 | 2951 | 3201 | 3234 | 3317 | 4859 | | |
| 18-2S | 2628 | 2641 | 2694 | 2760 | 2814 | 2854 | 2987 | 3411 | 3498 | 4424 | | | | |
| 18-5S | 1494 | 1750 | 1894 | 1920 | 2282 | 2381 | 2512 | 2689 | 2734 | 3014 | 3021 | 5618 | | |
| 18-8S | 1281 | 1298 | 1543 | 1611 | 1796 | 1871 | 2087 | 2333 | 2832 | 2834 | 2919 | 3337 | 3388 | 7259 |
| 19-1S | 2398 | 2670 | 3116 | 3167 | 3190 | 3266 | 3644 | 3722 | 3746 | | | | | |
| 19-2S | 2172 | 2736 | 3051 | 3182 | 3184 | 3451 | 3560 | 3693 | 3807 | 4042 | | | | |
| 19-5S | 1519 | 2534 | 2707 | 2956 | 3062 | 3346 | 3418 | 3532 | 3763 | 3764 | | | | |
| 19-8S | 2427 | 2637 | 2882 | 2905 | 2987 | 3121 | 3299 | 3409 | 3593 | 3675 | | | | |
| 20-1S | 2612 | 2651 | 2683 | 2756 | 2776 | 2924 | 2975 | 3000 | 3127 | 3181 | 3527 | | | |
| 20-4S | 2773 | 2818 | 2823 | 2836 | 2847 | 2929 | 2954 | 2970 | 3041 | 4305 | | | | |
| 20-7S | 2907 | 3143 | 3694 | 4165 | 4180 | 4471 | 8524 | | | | | | | |
| 20-8S | 1709 | 2081 | 2670 | 2891 | 3010 | 3230 | 3334 | 3376 | 3545 | 3955 | | | | |
| 21-1S | 2066 | 2219 | 2228 | 2279 | 2304 | 2313 | 2314 | 2332 | 2360 | 2382 | 2394 | 2452 | 2454 | |
| 21-4S | 2136 | 2148 | 2220 | 2235 | 2248 | 2277 | 2363 | 2419 | 2470 | 2484 | 2490 | 2584 | 2610 | |
| 21-7S | 1152 | 2142 | 2158 | 2367 | 2425 | 2451 | 2547 | 2619 | 2652 | 2868 | 3705 | 3854 | | |
| 21-8S | 2242 | 2276 | 2282 | 2284 | 2377 | 2450 | 2483 | 2493 | 2512 | 2513 | 2527 | 2528 | 2630 | |
| 22-1S | 1973 | 2156 | 2165 | 2188 | 2252 | 2319 | 2371 | 2413 | 2456 | 2498 | 2505 | 2606 | 2741 | |
| 22-4S | 2139 | 2301 | 2327 | 2358 | 2387 | 2434 | 2482 | 2593 | 2658 | 2760 | 2812 | 2886 | | |
| 22-7S | 2311 | 2325 | 2350 | 2472 | 2496 | 2509 | 2622 | 2683 | 2750 | 2881 | 2888 | 3390 | | |
| 22-8S | 2593 | 2630 | 2655 | 2701 | 2709 | 2886 | 2962 | 3107 | 3328 | 3399 | | | | |
| 23-1S | 1698 | 1736 | 2022 | 2151 | 2170 | 2199 | 2271 | 2607 | 2628 | 2887 | 3150 | 3176 | 3191 | |
| 23-2S | 2813 | 2814 | 2825 | 2888 | 2914 | 2976 | 2980 | 2982 | 3166 | 3589 | | | | |
| 23-5S | 2295 | 2608 | 2642 | 2651 | 2721 | 2723 | 2742 | 2748 | 2852 | 2871 | 3111 | | | |
| 23-8S | 2788 | 2866 | 2930 | 2939 | 2974 | 3074 | 3197 | 3245 | 3658 | 3873 | | | | |
| 24-1S | 2759 | 3214 | 3254 | 3279 | 3304 | 3705 | 3883 | 3954 | 3993 | 4297 | 4374 | 4454 | | |
| 24-2S | 2980 | 3092 | 3172 | 3251 | 3545 | 3745 | 3799 | 4034 | 4088 | 4222 | 5044 | | | |
| 24-5S | 3145 | 3368 | 3450 | 3571 | 3598 | 5073 | 5095 | 5544 | 6578 | | | | | |
| 24-8S | 3527 | 3580 | 3713 | 4129 | 4224 | 4464 | 4495 | 5722 | 5782 | | | | | |
| 26-1S | 2602 | 2784 | 2830 | 2831 | 2908 | 2942 | 3098 | 3118 | 3120 | 3454 | | | | |
| 26-4S | 2583 | 2927 | 2928 | 2984 | 3058 | 3071 | 3072 | 3266 | 3316 | 3495 | | | | |
| 26-7S | 2639 | 2771 | 2946 | 3053 | 3133 | 3285 | 3316 | 3386 | 3415 | 3481 | | | | |
| 26-8S | 2447 | 2449 | 2495 | 2576 | 2613 | 2618 | 2621 | 2660 | 2670 | 2778 | 2810 | 2866 | | |
| 27-1S | 2691 | 2850 | 2873 | 2877 | 2949 | 3001 | 3099 | 3156 | 3204 | 3381 | 3412 | | | |

| | | | | | | | | | | | |
|---|---|---|---|---|---|---|---|---|---|---|---|
| 27-3S | 2694 | 2722 | 2808 | 2989 | 3040 | 3041 | 3121 | 3196 | 3273 | 3282 | |
| 27-5S | 2723 | 2740 | 2763 | 2942 | 2979 | 2992 | 3096 | 3131 | 3178 | 3876 | |
| 27-8S | 2419 | 2633 | 2843 | 3016 | 3016 | 3183 | 3471 | 3693 | 4761 | | |
| 28-1S | 2769 | 2936 | 2948 | 2958 | 3024 | 3041 | 3101 | 3206 | 3329 | 3355 | |
| 28-3S | 1108 | 2588 | 2686 | 2716 | 2724 | 2806 | 2868 | 2868 | 2981 | 3022 | 3538 |
| 28-5S | 2702 | 2725 | 2744 | 2842 | 2947 | 3019 | 3027 | 3100 | 3108 | 3154 | |
| 28-8S | 2743 | 2804 | 2872 | 2912 | 2978 | 2983 | 3015 | 3017 | 3034 | 3068 | 3230 |
| 29-1S | 2383 | 2516 | 2642 | 2682 | 2706 | 2710 | 2730 | 2799 | 3080 | 3094 | 4047 |
| 29-4S | 2227 | 2568 | 2710 | 2731 | 3033 | 3054 | 3079 | 3239 | 3416 | 3434 | 3708 |
| 29-7S | 1906 | 2603 | 2905 | 3034 | 3091 | 3199 | 3316 | 3399 | 3654 | 4027 | |
| 29-8S | 2368 | 2578 | 2804 | 2831 | 2906 | 2952 | 3161 | 3229 | 3275 | 3314 | 3627 |
| 30-1S | 4498 | 4919 | 5130 | 5175 | 5347 | 5560 | | | | | |
| 30-2S | 2734 | 3218 | 3334 | 3365 | 4758 | 5162 | 5275 | 6223 | | | |
| 30-6S | 2264 | 2664 | 2990 | 3314 | 3353 | 3381 | 3876 | 4103 | 4173 | 4590 | |
| 30-8S | 4158 | 4169 | 4420 | 4467 | 4590 | 4767 | 5684 | | | | |
| 31-1S | 3660 | 4387 | 4500 | 4531 | 4715 | 4990 | 5724 | | | | |
| 31-4S | 3980 | 4152 | 4169 | 4417 | 4458 | 4694 | 5361 | | | | |
| 31-6S | 3587 | 3939 | 4154 | 4291 | 4304 | 4439 | 4485 | | | | |
| 31-8S | 4662 | 4701 | 4872 | 5116 | 5669 | 6287 | | | | | |
| 32-1S | 2903 | 2915 | 3157 | 3708 | 3736 | 3887 | 4842 | 5648 | | | |
| 32-3S | 2905 | 2913 | 2963 | 3053 | 3070 | 3215 | 3220 | 3369 | 3392 | 3457 | |
| 32-6S | 2137 | 2511 | 2834 | 2869 | 3161 | 3190 | 3312 | 4174 | 4458 | 4618 | |
| 32-8S | 2310 | 2449 | 2456 | 2722 | 2756 | 2769 | 2920 | 2933 | 2937 | 3233 | 4123 |
| 33-1S | 2559 | 2614 | 2735 | 2930 | 3020 | 3025 | 3060 | 3082 | 3155 | 3207 | 4809 |
| 33-6S | 3194 | 3247 | 3510 | 3522 | 3624 | 3789 | 3814 | 3823 | 3831 | | |
| 33-7S | 2784 | 2944 | 3027 | 3074 | 3109 | 3123 | 3132 | 3214 | 3574 | 3632 | |
| 33-8S | 2981 | 3012 | 3053 | 3087 | 3098 | 3127 | 3207 | 3332 | 3466 | 3860 | |
| 34-1S | 2961 | 3107 | 3177 | 3401 | 3529 | 3889 | 3898 | 3948 | 5127 | | |
| 34-3S | 2312 | 3118 | 3174 | 3290 | 3316 | 4121 | 4402 | 4624 | 6782 | | |
| 34-6S | 2637 | 2718 | 2834 | 2852 | 2932 | 2938 | 2974 | 2999 | 3141 | 3348 | |
| 34-8S | 2560 | 2925 | 3011 | 3066 | 3124 | 3171 | 3359 | 3420 | 3474 | 3689 | |
| 35-1S | 2728 | 2743 | 2882 | 2929 | 2936 | 2961 | 3047 | 3050 | 3128 | 3141 | 3534 |
| 35-3S | 2769 | 2787 | 2922 | 3095 | 3160 | 3185 | 3239 | 3363 | 3456 | 3523 | |
| 35-6S | 2509 | 2611 | 2682 | 2774 | 2784 | 2942 | 2973 | 3027 | 3163 | 3318 | |
| 35-8S | 2786 | 2848 | 2858 | 2875 | 2934 | 3003 | 3003 | 3098 | 3100 | 3301 | |
| 36-1S | 1720 | 3069 | 3241 | 3270 | 3357 | 3608 | 3621 | 3680 | 3755 | 4067 | |
| 36-2S | 2219 | 2629 | 2819 | 2843 | 2977 | 3832 | 3919 | 3941 | 4048 | 4100 | |
| 36-3S | 1635 | 2106 | 2625 | 2864 | 2937 | 3045 | 3221 | 3506 | 4022 | 4404 | 4538 |
| 36-8S | 3139 | 3277 | 3320 | 3360 | 3372 | 3375 | 3400 | 3459 | 3566 | 3790 | |

Phased session

| | | | | | | | | | | | | | | |
|---|---|---|---|---|---|---|---|---|---|---|---|---|---|---|
| 18-3E | 2406 | 2485 | 2604 | 2765 | 2802 | 3455 | 3938 | 3965 | 4423 | 4671 | | | | |
| 18-4I | 1712 | 2123 | 2364 | 2674 | 2870 | 4028 | 4527 | 4808 | | | | | | |
| 18-6I | 2702 | 2836 | 2838 | 2843 | 2863 | 2952 | 3033 | 3410 | 5674 | | | | | |
| 18-7E | 1977 | 2290 | 2544 | 2631 | 2708 | 3123 | 3133 | 3293 | 4147 | 4375 | | | | |
| 19-3I | 1986 | 2267 | 2418 | 2541 | 2562 | 2608 | 2825 | 2929 | 3009 | 3336 | 3824 | | | |
| 19-4E | 1995 | 2198 | 2260 | 2366 | 2375 | 3116 | 3202 | 3408 | 3636 | 3660 | | | | |
| 19-6E | 2275 | 2305 | 2973 | 3308 | 3400 | 3617 | 3667 | 3717 | 3789 | 3840 | 4289 | | | |
| 19-7I | 2591 | 2765 | 2773 | 2800 | 2870 | 2890 | 3105 | 3645 | 3985 | 4222 | | | | |
| 20-2E | 2404 | 2766 | 2852 | 2870 | 2872 | 2887 | 2894 | 2930 | 2945 | 3010 | 3144 | | | |
| 20-3I | 2854 | 2898 | 2951 | 3043 | 3088 | 3121 | 3213 | 3250 | 3314 | 3341 | | | | |
| 20-5I | 2885 | 2995 | 3014 | 3023 | 3106 | 3111 | 3359 | 3380 | 3483 | 3621 | | | | |
| 20-6E | 3251 | 3275 | 3381 | 3454 | 3484 | 3608 | 3698 | 3784 | 4155 | 4496 | | | | |
| 21-2I | 2136 | 2167 | 2222 | 2231 | 2332 | 2373 | 2454 | 2526 | 2619 | 2655 | 2668 | | | |
| 21-3E | 2256 | 2271 | 2287 | 2298 | 2315 | 2350 | 2386 | 2474 | 2515 | 2530 | 2532 | | | |
| 21-5E | 2230 | 2314 | 2356 | 2394 | 2404 | 2418 | 2432 | 2461 | 2467 | 2526 | 2667 | | | |
| 21-6I | 2195 | 2247 | 2283 | 2326 | 2345 | 2351 | 2357 | 2401 | 2424 | 2452 | 2485 | | | |
| 22-2E | 2695 | 2696 | 2744 | 2774 | 2846 | 2935 | 2970 | 3060 | 3092 | 3326 | | | | |
| 22-3I | 2144 | 2362 | 2408 | 2465 | 2477 | 2493 | 2702 | 2715 | 2785 | 2831 | 2983 | | | |
| 22-5I | 1928 | 2305 | 2331 | 2364 | 2449 | 2466 | 2483 | 2537 | 2559 | 2605 | 2734 | | | |
| 22-6E | 2698 | 2723 | 2775 | 2782 | 2815 | 2902 | 2965 | 2983 | 3003 | 3053 | | | | |
| 23-3E | 2810 | 2951 | 3061 | 3076 | 3125 | 3178 | 3204 | 3215 | 3250 | 3364 | | | | |
| 23-4I | 2706 | 2711 | 2818 | 2912 | 2920 | 3030 | 3044 | 3108 | 3199 | 3306 | | | | |
| 23-7E | 2750 | 2956 | 3001 | 3075 | 3090 | 3171 | 3196 | 3316 | 3682 | 3737 | | | | |
| 24-3I | 3239 | 3384 | 3612 | 3806 | 3868 | 4078 | 4090 | 4158 | 4173 | 4507 | 5129 | 5222 | 5400 | |
| 24-4E | 3467 | 3473 | 3485 | 3591 | 3619 | 3640 | 3771 | 3870 | 3986 | 4200 | 4383 | 4470 | 5332 | |
| 24-6E | 3147 | 3468 | 3646 | 3932 | 4157 | 4262 | 4347 | 4361 | 4752 | 5094 | 5333 | 6646 | 7437 | |
| 24-7I | 2301 | 2855 | 3426 | 3440 | 3528 | 3634 | 3733 | 3761 | 3770 | 4061 | 4114 | 4195 | 4266 | 5824 |
| 26-2E | 2408 | 2599 | 2691 | 2740 | 2775 | 2867 | 2905 | 2989 | 3157 | 3390 | | | | |
| 26-3I | 2504 | 2585 | 2619 | 2735 | 2775 | 2788 | 2861 | 2886 | 2967 | 3372 | | | | |
| 26-5I | 2621 | 2680 | 2703 | 2728 | 2736 | 2809 | 2856 | 2916 | 2931 | 2981 | | | | |
| 26-6E | 2836 | 2936 | 2994 | 3031 | 3204 | 3331 | 3417 | 3576 | 3590 | 4133 | | | | |
| 27-2I | 2685 | 2752 | 2800 | 2826 | 2846 | 2854 | 2880 | 2914 | 2951 | 2958 | | | | |
| 27-4E | 2857 | 2896 | 2949 | 3023 | 3069 | 3082 | 3126 | 3149 | 3192 | 3326 | | | | |
| 27-6E | 2856 | 2889 | 2982 | 3036 | 3149 | 3296 | 3323 | 3329 | 3480 | 3535 | 3622 | | | |
| 27-7I | 3020 | 3168 | 3241 | 3268 | 3335 | 3381 | 3438 | 3618 | 3663 | 3689 | | | | |
| 28-2E | 2538 | 2566 | 2673 | 2785 | 2801 | 2808 | 2814 | 2877 | 2921 | 2963 | 3038 | | | |
| 28-4I | 2734 | 2852 | 2861 | 2861 | 2873 | 2921 | 2947 | 2970 | 3112 | 3203 | | | | |

| | | | | | | | | | | | |
|---|---|---|---|---|---|---|---|---|---|---|---|
| 28-6I | 2839 | 2877 | 2940 | 2974 | 3081 | 3109 | 3149 | 3211 | 3322 | 3656 | |
| 28-7E | 2918 | 3003 | 3105 | 3194 | 3205 | 3222 | 3229 | 3243 | 3272 | 3357 | |
| 29-2I | 2849 | 2883 | 2908 | 3126 | 3234 | 3296 | 3675 | 3705 | 4112 | | |
| 29-3E | 2177 | 2716 | 2853 | 3093 | 3438 | 3653 | 3776 | 4163 | 4510 | 5440 | |
| 29-5E | 2680 | 2700 | 2719 | 2806 | 2852 | 2967 | 3054 | 3072 | 3155 | 3236 | 3897 |
| 29-6I | 2873 | 2988 | 3037 | 3114 | 3120 | 3146 | 3318 | 3343 | 3429 | 3445 | |
| 30-3I | 2871 | 4050 | 4156 | 4224 | 4534 | 4591 | 4691 | 4802 | 5009 | 6616 | |
| 30-4E | 3045 | 3469 | 3829 | 4244 | 4589 | 4604 | 4672 | 5004 | 6215 | 6698 | |
| 30-5E | 4033 | 4177 | 4347 | 4625 | 4694 | 4813 | 4944 | 5023 | 5695 | 5696 | |
| 30-7I | 3013 | 3649 | 3995 | 4431 | 4603 | 4657 | 4765 | 5072 | 5371 | 7388 | |
| 31-2I | 3533 | 3827 | 3896 | 3907 | 3935 | 3981 | 4022 | 4043 | 4305 | 4367 | |
| 31-3E | 3900 | 4028 | 4101 | 4124 | 4165 | 4288 | 4804 | 4895 | 4974 | 5655 | |
| 31-5I | 3380 | 3418 | 3496 | 3718 | 3722 | 3751 | 3837 | 3879 | 4061 | 4134 | |
| 31-7E | 4007 | 4043 | 4054 | 4158 | 4327 | 4342 | 4398 | 4404 | 4681 | 5132 | |
| 32-2E | 3239 | 3380 | 3443 | 3496 | 3549 | 3642 | 3785 | 3887 | 4194 | 4231 | |
| 32-4I | 2183 | 2502 | 2683 | 2841 | 2929 | 3209 | 3495 | 3553 | 3860 | 4057 | |
| 32-5E | 2552 | 2950 | 3108 | 3133 | 3538 | 3615 | 3813 | 4780 | 8911 | 10167 | |
| 32-7I | 2868 | 3008 | 3131 | 3132 | 3166 | 3432 | 3507 | 4441 | 4707 | 5350 | |
| 33-2I | 2891 | 2906 | 2906 | 2996 | 3065 | 3135 | 3247 | 3598 | 3657 | 4769 | |
| 33-3E | 3303 | 3306 | 3341 | 3391 | 3558 | 3562 | 3645 | 3728 | 3732 | 3932 | |
| 33-4I | 2686 | 2906 | 2955 | 3150 | 3292 | 3413 | 3441 | 3471 | 3709 | 3850 | |
| 33-5E | 3161 | 3216 | 3337 | 3344 | 3426 | 3434 | 3458 | 3462 | 3711 | 4275 | |
| 34-2I | 2792 | 2884 | 2894 | 2971 | 2981 | 3112 | 3157 | 3183 | 3197 | 3225 | |
| 34-4E | 3052 | 3152 | 3473 | 3480 | 3548 | 3601 | 3751 | 3850 | 4168 | 4596 | |
| 34-5I | 2707 | 3040 | 3089 | 3111 | 3114 | 3175 | 3188 | 3303 | 3518 | 4019 | |
| 34-7E | 3155 | 3177 | 3192 | 3227 | 3280 | 3343 | 3390 | 3395 | 3448 | 3517 | |
| 35-2E | 2758 | 2997 | 3027 | 3059 | 3089 | 3142 | 3201 | 3254 | 3290 | 3334 | |
| 35-4I | 2597 | 2665 | 2720 | 2734 | 2757 | 2762 | 2763 | 2796 | 2839 | 2840 | |
| 35-5E | 2919 | 3035 | 3068 | 3114 | 3142 | 3240 | 3243 | 3311 | 3312 | 3399 | 3431 |
| 35-7I | 2813 | 2814 | 2852 | 2897 | 2956 | 2985 | 3016 | 3071 | 3189 | 3284 | |
| 36-4E | 2777 | 3046 | 3082 | 3128 | 3223 | 3329 | 3393 | 3401 | 3531 | 3700 | |
| 36-5I | 1650 | 3076 | 3253 | 3341 | 3431 | 3455 | 3501 | 3507 | 3644 | 3668 | |
| 36-6I | 3181 | 3195 | 3229 | 3243 | 3287 | 3300 | 3388 | 3450 | 3726 | 3803 | |
| 36-7E | 2778 | 2972 | 3124 | 3243 | 3385 | 3425 | 3464 | 3700 | 3741 | 3815 | 3943 |
