# Supplementary material for: Respiratory modulation of cognitive performance during the retrieval process
Source: PLoS One. 2018 Sep 14;13(9):e0204021. doi: 10.1371/journal.pone.0204021 (PMC6138381; doi:10.1371/journal.pone.0204021)
Supplement: S3 Table — (PDF) [file pone.0204021.s005.pdf]

Table S3. Timing of peak I, IE transition, and peak E from EI transition during the test section in the Non-phased sessions

name, session	parameter	time (ms)	ratio (%)
18-1S	EI transition	31415	0.0
18-1S	peak I	31907	21.5
18-1S	IE transition	32178	33.4
18-1S	peak E	32534	49.0
18-1S	EI transition	33701	100.0
18-1S	peak I	34097	20.1
18-1S	IE transition	34428	36.8
18-1S	peak E	34607	45.9
18-1S	EI transition	35674	100.0
18-1S	peak I	35963	13.0
18-1S	IE transition	36283	27.5
18-1S	peak E	36731	47.7
18-1S	EI transition	37890	100.0
18-1S	peak I	38387	15.4
18-1S	IE transition	38746	26.5
18-1S	peak E	38922	31.9
18-1S	EI transition	41123	100.0
18-1S	peak I	41772	13.4
18-1S	IE transition	42028	18.6
18-1S	peak E	42554	29.5
18-1S	EI transition	45982	100.0
18-1S	peak I	46209	16.5
18-1S	IE transition	46428	32.5
18-1S	peak E	47185	87.6
18-1S	EI transition	47356	100.0
18-1S	peak I	47918	17.6
18-1S	IE transition	48195	26.2
18-1S	peak E	48722	42.7
18-1S	EI transition	50557	100.0
18-1S	peak I	51109	18.7
18-1S	IE transition	51654	37.2
18-1S	peak E	52316	59.6
18-1S	EI transition	53508	100.0
18-1S	peak I	53882	15.5
18-1S	IE transition	54268	31.4
18-1S	peak E	54669	48.0
18-1S	EI transition	55925	100.0
18-1S	peak I	56471	21.1
18-1S	IE transition	56988	41.1
18-1S	peak E	57189	48.9
18-1S	EI transition	58512	100.0
18-1S	peak I	59097	17.6
18-1S	IE transition	59612	33.2
18-1S	peak E	60137	49.0
18-2S	EI transition	31496	0.0
18-2S	peak I	31936	14.7
18-2S	IE transition	32254	25.4
18-2S	peak E	32609	37.3
18-2S	EI transition	34483	100.0
18-2S	peak I	35075	13.4
18-2S	IE transition	35714	27.8
18-2S	peak E	36329	41.7
18-2S	EI transition	38906	100.0
18-2S	peak I	39583	24.1
18-2S	IE transition	39866	34.1

name, session	parameter	time (ms)	ratio (%)
19-1S	EI transition	32442	0.0
19-1S	peak I	32743	9.4
19-1S	IE transition	33386	29.6
19-1S	peak E	33786	42.1
19-1S	EI transition	35632	100.0
19-1S	peak I	36006	10.0
19-1S	IE transition	36680	28.2
19-1S	peak E	37196	42.0
19-1S	EI transition	39354	100.0
19-1S	peak I	39676	8.6
19-1S	IE transition	40395	27.8
19-1S	peak E	40684	35.5
19-1S	EI transition	43099	100.0
19-1S	peak I	43541	18.4
19-1S	IE transition	43855	31.5
19-1S	peak E	44679	65.9
19-1S	EI transition	45497	100.0
19-1S	peak I	45856	11.0
19-1S	IE transition	46500	30.7
19-1S	peak E	46882	42.4
19-1S	EI transition	48763	100.0
19-1S	peak I	49096	9.1
19-1S	IE transition	49708	25.9
19-1S	peak E	49991	33.7
19-1S	EI transition	52407	100.0
19-1S	peak I	52695	9.2
19-1S	IE transition	53196	25.3
19-1S	peak E	53690	41.2
19-1S	EI transition	55523	100.0
19-1S	peak I	55876	13.2
19-1S	IE transition	56287	28.6
19-1S	peak E	56652	42.3
19-1S	EI transition	58193	100.0
19-1S	peak I	58473	10.0
19-1S	IE transition	58904	25.3
19-2S	EI transition	30492	0.0
19-2S	peak I	30756	8.7
19-2S	IE transition	31383	29.2
19-2S	peak E	31714	40.1
19-2S	EI transition	33543	100.0
19-2S	peak I	33786	11.2
19-2S	IE transition	34250	32.6
19-2S	peak E	34902	62.6
19-2S	EI transition	35715	100.0
19-2S	peak I	36102	10.9
19-2S	IE transition	36867	32.4
19-2S	peak E	37136	39.9
19-2S	EI transition	39274	100.0
19-2S	peak I	39566	7.7
19-2S	IE transition	40378	29.0
19-2S	peak E	40647	36.1
19-2S	EI transition	43081	100.0
19-2S	peak I	43393	11.4
19-2S	IE transition	43854	28.3
19-2S	peak E	44909	66.8

18-2S	peak E	40193	45.7
18-2S	EI transition	41720	100.0
18-2S	peak I	42229	18.9
18-2S	IE transition	42524	29.8
18-2S	peak E	43169	53.8
18-2S	EI transition	44414	100.0
18-2S	peak I	44907	18.8
18-2S	IE transition	45119	26.8
18-2S	peak E	45482	40.6
18-2S	EI transition	47042	100.0
18-2S	peak I	47614	20.0
18-2S	IE transition	47950	31.8
18-2S	peak E	48559	53.2
18-2S	EI transition	49896	100.0
18-2S	peak I	50891	28.4
18-2S	IE transition	51377	42.3
18-2S	peak E	51623	49.4
18-2S	EI transition	53394	100.0
18-2S	peak I	53835	12.9
18-2S	IE transition	54210	23.9
18-2S	peak E	54823	41.9
18-2S	EI transition	56805	100.0
18-2S	peak I	57370	20.5
18-2S	IE transition	57782	35.4
18-2S	peak E	58873	74.9
18-2S	EI transition	59565	100.0
18-5S	EI transition	30774	0.0
18-5S	peak I	31275	26.5
18-5S	IE transition	31517	39.2
18-5S	peak E	31904	59.7
18-5S	EI transition	32668	100.0
18-5S	peak I	33170	21.1
18-5S	IE transition	33533	36.3
18-5S	peak E	33975	54.9
18-5S	EI transition	35049	100.0
18-5S	peak I	35662	24.4
18-5S	IE transition	35916	34.5
18-5S	peak E	36543	59.5
18-5S	EI transition	37561	100.0
18-5S	peak I	37891	14.5
18-5S	IE transition	38276	31.3
18-5S	peak E	38620	46.4
18-5S	EI transition	39842	100.0
18-5S	peak I	40178	22.5
18-5S	IE transition	40394	36.9
18-5S	peak E	40861	68.2
18-5S	EI transition	41336	100.0
18-5S	peak I	41880	9.7
18-5S	IE transition	42238	16.1
18-5S	peak E	42709	24.4
18-5S	EI transition	46954	100.0
18-5S	peak I	47493	28.1
18-5S	IE transition	47783	43.2
18-5S	peak E	48358	73.1
18-5S	EI transition	48874	100.0
18-5S	peak I	49280	23.2
18-5S	IE transition	49599	41.4
18-5S	peak E	49859	56.3
18-5S	EI transition	50624	100.0
19-2S	EI transition	45817	100.0
19-2S	peak I	46081	8.3
19-2S	IE transition	46740	29.0
19-2S	peak E	47015	37.6
19-2S	EI transition	49001	100.0
19-2S	peak I	49281	7.6
19-2S	IE transition	50010	27.3
19-2S	peak E	50249	33.8
19-2S	EI transition	52694	100.0
19-2S	peak I	53026	10.4
19-2S	IE transition	53452	23.8
19-2S	peak E	53763	33.6
19-2S	EI transition	55876	100.0
19-2S	peak I	56191	7.8
19-2S	IE transition	56868	24.5
19-2S	peak E	59476	89.1
19-2S	EI transition	59918	100.0
19-5S	EI transition	29379	0.0
19-5S	peak I	29680	8.0
19-5S	IE transition	30423	27.7
19-5S	peak E	30868	39.6
19-5S	EI transition	33142	100.0
19-5S	peak I	33433	8.2
19-5S	IE transition	34145	28.4
19-5S	peak E	34516	38.9
19-5S	EI transition	36674	100.0
19-5S	peak I	36920	9.1
19-5S	IE transition	37456	28.9
19-5S	peak E	38172	55.3
19-5S	EI transition	39381	100.0
19-5S	peak I	39701	12.7
19-5S	IE transition	40093	28.1
19-5S	peak E	41224	72.8
19-5S	EI transition	41914	100.0
19-5S	peak I	42230	8.4
19-5S	IE transition	43083	31.1
19-5S	peak E	43392	39.3
19-5S	EI transition	45678	100.0
19-5S	peak I	45888	13.8
19-5S	IE transition	46143	30.6
19-5S	peak E	46465	51.8
19-5S	EI transition	47197	100.0
19-5S	peak I	47458	8.8
19-5S	IE transition	47990	26.8
19-5S	peak E	48362	39.4
19-5S	EI transition	50153	100.0
19-5S	peak I	50510	10.7
19-5S	IE transition	51075	27.6
19-5S	peak E	51502	40.3
19-5S	EI transition	53499	100.0
19-5S	peak I	53810	9.1
19-5S	IE transition	54435	27.4
19-5S	peak E	54892	40.8
19-5S	EI transition	56917	100.0
19-5S	peak I	57179	8.6
19-5S	IE transition	57660	24.3
19-5S	peak E	57999	35.3
19-5S	EI transition	59979	100.0
19-8S	EI transition	30522	0.0

18-5S	peak I	51389	25.4
18-5S	IE transition	51774	38.2
18-5S	peak E	51949	44.0
18-5S	EI transition	53638	100.0
18-5S	peak I	54307	22.1
18-5S	IE transition	54728	36.1
18-5S	peak E	54950	43.4
18-5S	EI transition	56659	100.0
18-5S	peak I	57177	19.3
18-5S	IE transition	57628	36.0
18-5S	peak E	58433	66.0
18-5S	EI transition	59348	100.0
18-8S	EI transition	30981	0.0
18-8S	peak I	31423	28.6
18-8S	IE transition	31611	40.8
18-8S	peak E	32026	67.7
18-8S	EI transition	32524	100.0
18-8S	peak I	32907	23.8
18-8S	IE transition	33091	35.2
18-8S	peak E	33499	60.5
18-8S	EI transition	34135	100.0
18-8S	peak I	34454	24.6
18-8S	IE transition	34649	39.6
18-8S	peak E	34974	64.6
18-8S	EI transition	35433	100.0
18-8S	peak I	36033	21.2
18-8S	IE transition	36337	31.9
18-8S	peak E	36517	38.2
18-8S	EI transition	38267	100.0
18-8S	peak I	38713	23.8
18-8S	IE transition	38929	35.4
18-8S	peak E	39150	47.2
18-8S	EI transition	40137	100.0
18-8S	peak I	40601	36.2
18-8S	IE transition	40846	55.3
18-8S	peak E	41209	83.7
18-8S	EI transition	41418	100.0
18-8S	peak I	41860	15.1
18-8S	IE transition	42333	31.3
18-8S	peak E	42769	46.3
18-8S	EI transition	44337	100.0
18-8S	peak I	44732	11.7
18-8S	IE transition	44965	18.5
18-8S	peak E	45111	22.8
18-8S	EI transition	47725	100.0
18-8S	peak I	48156	20.7
18-8S	IE transition	48428	33.7
18-8S	peak E	48645	44.1
18-8S	EI transition	49812	100.0
18-8S	peak I	50382	17.1
18-8S	IE transition	50975	34.9
18-8S	peak E	51358	46.3
18-8S	EI transition	53149	100.0
18-8S	peak I	53692	19.2
18-8S	IE transition	54057	32.1
18-8S	peak E	54753	56.6
18-8S	EI transition	55981	100.0
18-8S	peak I	56397	23.2
18-8S	IE transition	56700	40.0

19-8S	peak I	30824	10.1
19-8S	IE transition	31456	31.3
19-8S	peak E	31796	42.7
19-8S	EI transition	33509	100.0
19-8S	peak I	33823	10.9
19-8S	IE transition	34231	25.1
19-8S	peak E	35442	67.1
19-8S	EI transition	36391	100.0
19-8S	peak I	36683	8.6
19-8S	IE transition	37438	30.7
19-8S	peak E	37777	40.7
19-8S	EI transition	39799	100.0
19-8S	peak I	40074	11.3
19-8S	IE transition	40475	27.9
19-8S	peak E	40765	39.8
19-8S	EI transition	42226	100.0
19-8S	peak I	42498	7.6
19-8S	IE transition	43144	25.5
19-8S	peak E	43426	33.4
19-8S	EI transition	45819	100.0
19-8S	peak I	46091	8.7
19-8S	IE transition	46782	30.9
19-8S	peak E	47168	43.2
19-8S	EI transition	48940	100.0
19-8S	peak I	49155	8.2
19-8S	IE transition	49599	25.0
19-8S	peak E	49812	33.1
19-8S	EI transition	51577	100.0
19-8S	peak I	51865	9.9
19-8S	IE transition	52395	28.2
19-8S	peak E	53043	50.5
19-8S	EI transition	54482	100.0
19-8S	peak I	54853	10.1
19-8S	IE transition	55630	31.2
19-8S	peak E	55896	38.5
19-8S	EI transition	58157	100.0

18-8S	peak E	57425	80.4
18-8S	EI transition	57777	100.0
18-8S	peak I	59302	21.0
18-8S	IE transition	61456	50.7

name, session	parameter	time (ms)	ratio (%)
20-1S	EI transition	30819	0.0
20-1S	peak I	31134	10.5
20-1S	IE transition	31834	33.8
20-1S	peak E	32158	44.6
20-1S	EI transition	33819	100.0
20-1S	peak I	34251	16.1
20-1S	IE transition	34802	36.6
20-1S	peak E	35170	50.4
20-1S	EI transition	36502	100.0
20-1S	peak I	37122	19.5
20-1S	IE transition	37631	35.5
20-1S	peak E	37957	45.7
20-1S	EI transition	39683	100.0
20-1S	peak I	40159	16.3
20-1S	IE transition	40790	37.9
20-1S	peak E	41056	47.0
20-1S	EI transition	42607	100.0
20-1S	peak I	43102	19.0
20-1S	IE transition	43757	44.0
20-1S	peak E	44051	55.3
20-1S	EI transition	45218	100.0
20-1S	peak I	45507	10.5
20-1S	IE transition	46171	34.6
20-1S	peak E	46611	50.5
20-1S	EI transition	47974	100.0
20-1S	peak I	48481	16.2
20-1S	IE transition	49050	34.4
20-1S	peak E	50283	73.8
20-1S	EI transition	51101	100.0
20-1S	peak I	51471	10.5
20-1S	IE transition	52194	31.0
20-1S	peak E	52482	39.2
20-1S	EI transition	54628	100.0
20-1S	peak I	55104	17.1
20-1S	IE transition	55563	33.7
20-1S	peak E	57156	91.1
20-1S	EI transition	57404	100.0
20-1S	peak I	57827	16.0
20-1S	IE transition	58357	35.9
20-1S	peak E	58849	54.5
20-1S	EI transition	60055	100.0
20-4S	EI transition	29725	0.0
20-4S	peak I	30156	15.3
20-4S	IE transition	30723	35.4
20-4S	peak E	31217	52.9
20-4S	EI transition	32548	100.0
20-4S	peak I	33011	15.2
20-4S	IE transition	33604	34.7
20-4S	peak E	34063	49.8
20-4S	EI transition	35589	100.0
20-4S	peak I	36186	20.1
20-4S	IE transition	36676	36.6
20-4S	peak E	37007	47.7
20-4S	EI transition	38559	100.0
20-4S	peak I	39069	17.4
20-4S	IE transition	39660	37.6
20-4S	peak E	40148	54.3

name, session	parameter	time (ms)	ratio (%)
21-1S	EI transition	31776	0.0
21-1S	peak I	32086	13.1
21-1S	IE transition	32798	43.3
21-1S	peak E	33044	53.7
21-1S	EI transition	34136	100.0
21-1S	peak I	34457	14.5
21-1S	IE transition	35065	41.9
21-1S	peak E	35227	49.2
21-1S	EI transition	36355	100.0
21-1S	peak I	36585	9.7
21-1S	IE transition	37333	41.1
21-1S	peak E	37942	66.6
21-1S	EI transition	38737	100.0
21-1S	peak I	39008	11.7
21-1S	IE transition	39716	42.3
21-1S	peak E	40059	57.2
21-1S	EI transition	41050	100.0
21-1S	peak I	41341	13.1
21-1S	IE transition	41942	40.0
21-1S	peak E	42124	48.2
21-1S	EI transition	43278	100.0
21-1S	peak I	43603	15.7
21-1S	IE transition	44208	45.0
21-1S	peak E	44453	56.9
21-1S	EI transition	45344	100.0
21-1S	peak I	45677	14.3
21-1S	IE transition	46307	41.3
21-1S	peak E	47069	74.0
21-1S	EI transition	47675	100.0
21-1S	peak I	47921	10.0
21-1S	IE transition	48665	40.3
21-1S	peak E	49222	63.0
21-1S	EI transition	50129	100.0
21-1S	peak I	50427	12.9
21-1S	IE transition	51111	42.4
21-1S	peak E	51707	68.2
21-1S	EI transition	52443	100.0
21-1S	peak I	52754	13.6
21-1S	IE transition	53381	41.2
21-1S	peak E	53841	61.3
21-1S	EI transition	54722	100.0
21-1S	peak I	55039	13.8
21-1S	IE transition	55718	43.2
21-1S	peak E	56019	56.3
21-1S	EI transition	57026	100.0
21-1S	peak I	57316	12.1
21-1S	IE transition	58053	42.9
21-1S	peak E	58568	64.4
21-1S	EI transition	59420	100.0
21-4S	EI transition	29765	0.0
21-4S	peak I	30142	15.1
21-4S	IE transition	30837	43.1
21-4S	peak E	31585	73.1
21-4S	EI transition	32255	100.0
21-4S	peak I	32574	12.9
21-4S	IE transition	33281	41.5
21-4S	peak E	33645	56.3

20-4S	EI transition	41488	100.0
20-4S	peak I	42065	20.3
20-4S	IE transition	42512	36.0
20-4S	peak E	42893	49.4
20-4S	EI transition	44335	100.0
20-4S	peak I	44805	16.7
20-4S	IE transition	45303	34.4
20-4S	peak E	45657	46.9
20-4S	EI transition	47153	100.0
20-4S	peak I	47637	17.1
20-4S	IE transition	48190	36.6
20-4S	peak E	48542	49.0
20-4S	EI transition	49988	100.0
20-4S	peak I	50464	11.1
20-4S	IE transition	51304	30.6
20-4S	peak E	53246	75.7
20-4S	EI transition	54293	100.0
20-4S	peak I	55010	24.3
20-4S	IE transition	55409	37.8
20-4S	peak E	55734	48.8
20-4S	EI transition	57247	100.0
20-4S	peak I	57735	17.6
20-4S	IE transition	58262	36.6
20-4S	peak E	58611	49.2
20-4S	EI transition	60020	100.0
20-7S	EI transition	29575	0.0
20-7S	peak I	30025	5.3
20-7S	IE transition	32624	35.8
20-7S	peak E	33073	41.0
20-7S	EI transition	38099	100.0
20-7S	peak I	38861	18.2
20-7S	IE transition	39210	26.6
20-7S	peak E	39503	33.6
20-7S	EI transition	42279	100.0
20-7S	peak I	43102	18.4
20-7S	IE transition	43445	26.1
20-7S	peak E	43602	29.6
20-7S	EI transition	46749	100.0
20-7S	peak I	47629	21.1
20-7S	IE transition	47988	29.7
20-7S	peak E	48578	43.9
20-7S	EI transition	50914	100.0
20-7S	peak I	51529	16.6
20-7S	IE transition	51932	27.6
20-7S	peak E	52289	37.2
20-7S	EI transition	54608	100.0
20-7S	peak I	55355	23.8
20-7S	IE transition	55836	39.1
20-7S	peak E	56220	51.3
20-7S	EI transition	57751	100.0
20-7S	peak I	58268	17.8
20-7S	IE transition	58738	34.0
20-7S	peak E	59296	53.1
20-8S	EI transition	30115	0.0
20-8S	peak I	30803	17.4
20-8S	IE transition	31237	28.4
20-8S	peak E	33804	93.3
20-8S	EI transition	34070	100.0
20-8S	peak I	34713	18.1
21-4S	EI transition	34725	100.0
21-4S	peak I	35083	13.9
21-4S	IE transition	35717	38.4
21-4S	peak E	35967	48.1
21-4S	EI transition	37309	100.0
21-4S	peak I	37581	11.5
21-4S	IE transition	38311	42.4
21-4S	peak E	38590	54.2
21-4S	EI transition	39672	100.0
21-4S	peak I	40035	15.9
21-4S	IE transition	40589	40.3
21-4S	peak E	40815	50.2
21-4S	EI transition	41949	100.0
21-4S	peak I	42259	14.0
21-4S	IE transition	42916	43.6
21-4S	peak E	43447	67.5
21-4S	EI transition	44169	100.0
21-4S	peak I	44523	13.6
21-4S	IE transition	45153	37.7
21-4S	peak E	45612	55.3
21-4S	EI transition	46779	100.0
21-4S	peak I	47071	13.7
21-4S	IE transition	47733	44.7
21-4S	peak E	47947	54.7
21-4S	EI transition	48914	100.0
21-4S	peak I	49278	16.9
21-4S	IE transition	49860	44.0
21-4S	peak E	50267	63.0
21-4S	EI transition	51062	100.0
21-4S	peak I	51412	14.1
21-4S	IE transition	51984	37.1
21-4S	peak E	52285	49.2
21-4S	EI transition	53546	100.0
21-4S	peak I	53870	13.4
21-4S	IE transition	54511	39.9
21-4S	peak E	54778	50.9
21-4S	EI transition	55965	100.0
21-4S	peak I	56267	13.4
21-4S	IE transition	56949	43.8
21-4S	peak E	57103	50.6
21-4S	EI transition	58213	100.0
21-4S	peak I	58595	17.1
21-4S	IE transition	59163	42.5
21-4S	peak E	59402	53.2
21-7S	EI transition	30697	0.0
21-7S	peak I	30985	11.8
21-7S	IE transition	31671	39.7
21-7S	peak E	32576	76.7
21-7S	EI transition	33148	100.0
21-7S	peak I	33443	12.2
21-7S	IE transition	34135	40.7
21-7S	peak E	34437	53.2
21-7S	EI transition	35573	100.0
21-7S	peak I	36011	18.5
21-7S	IE transition	36481	38.4
21-7S	peak E	36990	59.9
21-7S	EI transition	37940	100.0
21-7S	peak I	38237	13.8
21-7S	IE transition	38869	43.0

20-8S	IE transition	35224	32.6
20-8S	peak E	35707	46.2
20-8S	EI transition	37615	100.0
20-8S	peak I	38234	23.2
20-8S	IE transition	38708	40.9
20-8S	peak E	39004	52.0
20-8S	EI transition	40285	100.0
20-8S	peak I	40982	20.6
20-8S	IE transition	41378	32.4
20-8S	peak E	41909	48.1
20-8S	EI transition	43661	100.0
20-8S	peak I	44453	26.3
20-8S	IE transition	44942	42.6
20-8S	peak E	45257	53.1
20-8S	EI transition	46670	100.0
20-8S	peak I	47076	19.5
20-8S	IE transition	47403	35.2
20-8S	peak E	48089	68.2
20-8S	EI transition	48751	100.0
20-8S	peak I	49577	28.6
20-8S	IE transition	49990	42.9
20-8S	peak E	50992	77.5
20-8S	EI transition	51642	100.0
20-8S	peak I	52408	23.7
20-8S	IE transition	52731	33.7
20-8S	peak E	53842	68.1
20-8S	EI transition	54872	100.0
20-8S	peak I	55258	22.6
20-8S	IE transition	55488	36.0
20-8S	peak E	56304	83.8
20-8S	EI transition	56581	100.0
20-8S	peak I	56778	5.9
20-8S	IE transition	57345	22.9
20-8S	peak E	57557	29.3
20-8S	EI transition	59915	100.0

21-7S	peak E	39208	58.8
21-7S	EI transition	40098	100.0
21-7S	peak I	40456	16.7
21-7S	IE transition	40957	40.1
21-7S	peak E	41349	58.4
21-7S	EI transition	42240	100.0
21-7S	peak I	42564	28.1
21-7S	IE transition	43076	72.6
21-7S	peak E	43162	80.0
21-7S	EI transition	43392	100.0
21-7S	peak I	43681	7.5
21-7S	IE transition	44255	22.4
21-7S	peak E	46429	78.8
21-7S	EI transition	47245	100.0
21-7S	peak I	47489	9.2
21-7S	IE transition	48210	36.4
21-7S	peak E	48917	63.0
21-7S	EI transition	49897	100.0
21-7S	peak I	50269	13.0
21-7S	IE transition	50890	34.6
21-7S	peak E	51828	67.3
21-7S	EI transition	52765	100.0
21-7S	peak I	53287	14.1
21-7S	IE transition	53655	24.0
21-7S	peak E	53772	27.2
21-7S	EI transition	56470	100.0
21-7S	peak I	56746	10.8
21-7S	IE transition	57432	37.8
21-7S	peak E	58384	75.1
21-7S	EI transition	59017	100.0
21-8S	EI transition	30743	0.0
21-8S	peak I	31040	11.8
21-8S	IE transition	31748	40.0
21-8S	peak E	31941	47.7
21-8S	EI transition	33255	100.0
21-8S	peak I	33510	9.7
21-8S	IE transition	34248	37.8
21-8S	peak E	34454	45.6
21-8S	EI transition	35885	100.0
21-8S	peak I	36212	12.9
21-8S	IE transition	36926	41.2
21-8S	peak E	37162	50.5
21-8S	EI transition	38413	100.0
21-8S	peak I	38733	12.9
21-8S	IE transition	39463	42.3
21-8S	peak E	39822	56.7
21-8S	EI transition	40896	100.0
21-8S	peak I	41242	14.6
21-8S	IE transition	41898	42.2
21-8S	peak E	42088	50.1
21-8S	EI transition	43273	100.0
21-8S	peak I	43567	12.9
21-8S	IE transition	44227	41.9
21-8S	peak E	44397	49.4
21-8S	EI transition	45549	100.0
21-8S	peak I	45906	15.6
21-8S	IE transition	46507	42.0
21-8S	peak E	46763	53.2
21-8S	EI transition	47830	100.0

21-8S	peak I	48102	10.8
21-8S	IE transition	48809	38.7
21-8S	peak E	49247	56.1
21-8S	EI transition	50357	100.0
21-8S	peak I	50624	11.7
21-8S	IE transition	51336	42.9
21-8S	peak E	51679	57.9
21-8S	EI transition	52641	100.0
21-8S	peak I	52999	14.6
21-8S	IE transition	53639	40.7
21-8S	peak E	53840	48.9
21-8S	EI transition	55091	100.0
21-8S	peak I	55375	12.7
21-8S	IE transition	56035	42.1
21-8S	peak E	56315	54.6
21-8S	EI transition	57333	100.0
21-8S	peak I	57616	11.3
21-8S	IE transition	58383	41.8
21-8S	peak E	58639	52.0
21-8S	EI transition	59846	100.0

name, session	parameter	time (ms)	ratio (%)
22-1S	EI transition	30812	0.0
22-1S	peak I	31091	12.4
22-1S	IE transition	31790	43.4
22-1S	peak E	32680	82.9
22-1S	EI transition	33064	100.0
22-1S	peak I	33332	12.4
22-1S	IE transition	33938	40.5
22-1S	peak E	34666	74.3
22-1S	EI transition	35220	100.0
22-1S	peak I	35503	11.7
22-1S	IE transition	36119	37.3
22-1S	peak E	37262	84.6
22-1S	EI transition	37633	100.0
22-1S	peak I	37961	13.4
22-1S	IE transition	38621	40.2
22-1S	peak E	39645	81.9
22-1S	EI transition	40089	100.0
22-1S	peak I	40449	16.6
22-1S	IE transition	41000	42.1
22-1S	peak E	42056	90.9
22-1S	EI transition	42254	100.0
22-1S	peak I	42762	23.2
22-1S	IE transition	43155	41.2
22-1S	peak E	44112	84.9
22-1S	EI transition	44442	100.0
22-1S	peak I	44869	17.0
22-1S	IE transition	45366	36.9
22-1S	peak E	45809	54.6
22-1S	EI transition	46947	100.0
22-1S	peak I	47326	14.6
22-1S	IE transition	47886	36.1
22-1S	peak E	49088	82.2
22-1S	EI transition	49552	100.0
22-1S	peak I	49841	11.6
22-1S	IE transition	50593	41.7
22-1S	peak E	51793	89.7
22-1S	EI transition	52050	100.0
22-1S	peak I	52500	19.0
22-1S	IE transition	52999	40.0
22-1S	peak E	53215	49.1
22-1S	EI transition	54421	100.0
22-1S	peak I	54945	22.6
22-1S	IE transition	55373	41.1
22-1S	peak E	56426	86.5
22-1S	EI transition	56740	100.0
22-1S	peak I	57016	14.0
22-1S	IE transition	57640	45.6
22-1S	peak E	58174	72.7
22-1S	EI transition	58713	100.0
22-4S	EI transition	30271	0.0
22-4S	peak I	30645	15.4
22-4S	IE transition	31243	39.9
22-4S	peak E	32077	74.2
22-4S	EI transition	32705	100.0
22-4S	peak I	33081	14.5
22-4S	IE transition	33694	38.1
22-4S	peak E	34796	80.6

name, session	parameter	time (ms)	ratio (%)
23-1S	EI transition	31124	0.0
23-1S	peak I	31764	20.3
23-1S	IE transition	32265	36.2
23-1S	peak E	32645	48.3
23-1S	EI transition	34274	100.0
23-1S	peak I	34817	18.8
23-1S	IE transition	35256	34.0
23-1S	peak E	35507	42.7
23-1S	EI transition	37161	100.0
23-1S	peak I	38077	28.7
23-1S	IE transition	38321	36.4
23-1S	peak E	38617	45.6
23-1S	EI transition	40352	100.0
23-1S	peak I	40705	20.3
23-1S	IE transition	41374	58.9
23-1S	peak E	41654	75.0
23-1S	EI transition	42088	100.0
23-1S	peak I	42326	11.1
23-1S	IE transition	42982	41.6
23-1S	peak E	43199	51.7
23-1S	EI transition	44239	100.0
23-1S	peak I	44663	18.7
23-1S	IE transition	45216	43.0
23-1S	peak E	45888	72.6
23-1S	EI transition	46510	100.0
23-1S	peak I	46928	16.0
23-1S	IE transition	47446	35.9
23-1S	peak E	47836	50.9
23-1S	EI transition	49116	100.0
23-1S	peak I	49816	26.6
23-1S	IE transition	50158	39.6
23-1S	peak E	50451	50.8
23-1S	EI transition	51744	100.0
23-1S	peak I	52183	25.9
23-1S	IE transition	52404	38.9
23-1S	peak E	52502	44.6
23-1S	EI transition	53442	100.0
23-1S	peak I	53635	8.9
23-1S	IE transition	54291	39.1
23-1S	peak E	54686	57.3
23-1S	EI transition	55612	100.0
23-1S	peak I	55980	16.7
23-1S	IE transition	56481	39.5
23-1S	peak E	57072	66.4
23-1S	EI transition	57811	100.0
23-1S	peak I	58286	23.5
23-1S	IE transition	58681	43.0
23-1S	peak E	59063	61.9
23-1S	EI transition	59833	100.0
23-2S	EI transition	31726	0.0
23-2S	peak I	32234	18.0
23-2S	IE transition	32732	35.6
23-2S	peak E	33077	47.8
23-2S	EI transition	34551	100.0
23-2S	peak I	35090	15.0
23-2S	IE transition	35610	29.5
23-2S	peak E	35806	35.0

22-4S	EI transition	35298	100.0	23-2S	EI transition	38140	100.0
22-4S	peak I	35596	12.5	23-2S	peak I	38740	21.3
22-4S	IE transition	36313	42.5	23-2S	IE transition	39036	31.8
22-4S	peak E	37221	80.6	23-2S	peak E	39262	39.9
22-4S	EI transition	37685	100.0	23-2S	EI transition	40954	100.0
22-4S	peak I	37984	10.6	23-2S	peak I	41535	19.5
22-4S	IE transition	38680	35.4	23-2S	IE transition	42029	36.1
22-4S	peak E	39940	80.2	23-2S	peak E	42216	42.3
22-4S	EI transition	40497	100.0	23-2S	EI transition	43934	100.0
22-4S	peak I	40911	15.6	23-2S	peak I	44448	18.3
22-4S	IE transition	41440	35.5	23-2S	IE transition	45025	38.8
22-4S	peak E	42592	78.8	23-2S	peak E	45327	49.5
22-4S	EI transition	43155	100.0	23-2S	EI transition	46747	100.0
22-4S	peak I	43609	21.2	23-2S	peak I	47317	19.1
22-4S	IE transition	44066	42.6	23-2S	IE transition	47863	37.5
22-4S	peak E	44751	74.6	23-2S	peak E	48057	44.0
22-4S	EI transition	45294	100.0	23-2S	EI transition	49728	100.0
22-4S	peak I	45577	12.0	23-2S	peak I	50334	21.0
22-4S	IE transition	46235	39.9	23-2S	IE transition	50771	36.1
22-4S	peak E	46787	63.3	23-2S	peak E	51174	50.1
22-4S	EI transition	47651	100.0	23-2S	EI transition	52616	100.0
22-4S	peak I	48015	14.7	23-2S	peak I	53129	16.2
22-4S	IE transition	48643	40.0	23-2S	IE transition	53724	35.0
22-4S	peak E	49168	61.1	23-2S	peak E	53989	43.4
22-4S	EI transition	50133	100.0	23-2S	EI transition	55782	100.0
22-4S	peak I	50501	13.3	23-2S	peak I	56361	19.5
22-4S	IE transition	51167	37.5	23-2S	IE transition	56820	34.9
22-4S	peak E	52275	77.6	23-2S	peak E	57046	42.5
22-4S	EI transition	52893	100.0	23-2S	EI transition	58758	100.0
22-4S	peak I	53363	16.3	23-5S	EI transition	29862	0.0
22-4S	IE transition	53906	35.1	23-5S	peak I	30317	16.7
22-4S	peak E	54608	59.4	23-5S	IE transition	30992	41.5
22-4S	EI transition	55779	100.0	23-5S	peak E	31393	56.3
22-4S	peak I	56161	16.4	23-5S	EI transition	32583	100.0
22-4S	IE transition	56732	41.0	23-5S	peak I	33150	21.7
22-4S	peak E	57046	54.4	23-5S	IE transition	33660	41.3
22-4S	EI transition	58106	100.0	23-5S	peak E	34012	54.8
22-4S	peak I	58514	17.7	23-5S	EI transition	35191	100.0
22-4S	IE transition	59123	44.2	23-5S	peak I	35816	23.0
22-4S	peak E	60017	83.1	23-5S	IE transition	36212	37.5
22-7S	EI transition	31033	0.0	23-5S	peak E	36437	45.8
22-7S	peak I	31456	14.7	23-5S	EI transition	37914	100.0
22-7S	IE transition	32022	34.3	23-5S	peak I	38349	15.8
22-7S	peak E	33291	78.4	23-5S	IE transition	38932	37.0
22-7S	EI transition	33914	100.0	23-5S	peak E	39195	46.6
22-7S	peak I	34371	17.4	23-5S	EI transition	40662	100.0
22-7S	IE transition	34973	40.4	23-5S	peak I	41102	14.1
22-7S	peak E	36216	87.8	23-5S	IE transition	41657	32.0
22-7S	EI transition	36536	100.0	23-5S	peak E	42047	44.5
22-7S	peak I	36972	18.8	23-5S	EI transition	43773	100.0
22-7S	IE transition	37451	39.4	23-5S	peak I	44349	21.7
22-7S	peak E	38489	84.0	23-5S	IE transition	44802	38.8
22-7S	EI transition	38861	100.0	23-5S	peak E	45121	50.8
22-7S	peak I	39193	14.1	23-5S	EI transition	46424	100.0
22-7S	IE transition	39785	39.3	23-5S	peak I	47279	32.4
22-7S	peak E	40723	79.2	23-5S	IE transition	47521	41.6
22-7S	EI transition	41211	100.0	23-5S	peak E	47885	55.3
22-7S	peak I	41575	13.2	23-5S	EI transition	49065	100.0
22-7S	IE transition	42176	35.1	23-5S	peak I	49645	21.2

22-7S	peak E	43514	83.7
22-7S	EI transition	43961	100.0
22-7S	peak I	44265	11.3
22-7S	IE transition	44952	36.9
22-7S	peak E	46301	87.2
22-7S	EI transition	46644	100.0
22-7S	peak I	47137	17.1
22-7S	IE transition	47710	36.9
22-7S	peak E	49014	82.1
22-7S	EI transition	49531	100.0
22-7S	peak I	50174	27.8
22-7S	IE transition	50503	42.1
22-7S	peak E	51125	69.0
22-7S	EI transition	51842	100.0
22-7S	peak I	52211	14.9
22-7S	IE transition	52783	38.1
22-7S	peak E	53714	75.7
22-7S	EI transition	54314	100.0
22-7S	peak I	54789	18.9
22-7S	IE transition	55347	41.2
22-7S	peak E	56338	80.7
22-7S	EI transition	56823	100.0
22-7S	peak I	57142	12.8
22-7S	IE transition	57816	39.8
22-7S	peak E	58993	86.9
22-7S	EI transition	59319	100.0
22-8S	EI transition	29871	0.0
22-8S	peak I	30299	13.8
22-8S	IE transition	30871	32.2
22-8S	peak E	31198	42.7
22-8S	EI transition	32978	100.0
22-8S	peak I	33432	17.1
22-8S	IE transition	33942	36.3
22-8S	peak E	34573	60.1
22-8S	EI transition	35633	100.0
22-8S	peak I	36088	17.5
22-8S	IE transition	36570	36.1
22-8S	peak E	37716	80.3
22-8S	EI transition	38226	100.0
22-8S	peak I	38587	12.5
22-8S	IE transition	39215	34.3
22-8S	peak E	40516	79.3
22-8S	EI transition	41112	100.0
22-8S	peak I	41605	16.6
22-8S	IE transition	42138	34.6
22-8S	peak E	43157	69.0
22-8S	EI transition	44074	100.0
22-8S	peak I	44490	15.4
22-8S	IE transition	45074	36.9
22-8S	peak E	45655	58.4
22-8S	EI transition	46782	100.0
22-8S	peak I	47261	17.7
22-8S	IE transition	47830	38.8
22-8S	peak E	49015	82.7
22-8S	EI transition	49483	100.0
22-8S	peak I	49787	11.6
22-8S	IE transition	50435	36.2
22-8S	peak E	51436	74.3
22-8S	EI transition	52113	100.0
23-5S	IE transition	50124	38.6
23-5S	peak E	50300	45.0
23-5S	EI transition	51807	100.0
23-5S	peak I	52376	20.0
23-5S	IE transition	52886	37.8
23-5S	peak E	53220	49.5
23-5S	EI transition	54659	100.0
23-5S	peak I	55480	28.6
23-5S	IE transition	55766	38.6
23-5S	peak E	56072	49.2
23-5S	EI transition	57530	100.0
23-5S	peak I	57854	14.1
23-5S	IE transition	58581	45.8
23-5S	peak E	59021	65.0
23-5S	EI transition	59825	100.0
23-8S	EI transition	31377	0.0
23-8S	peak I	32139	23.5
23-8S	IE transition	32361	30.3
23-8S	peak E	33423	63.1
23-8S	EI transition	34622	100.0
23-8S	peak I	35166	14.9
23-8S	IE transition	35742	30.6
23-8S	peak E	36373	47.9
23-8S	EI transition	38280	100.0
23-8S	peak I	38763	16.2
23-8S	IE transition	39419	38.3
23-8S	peak E	40131	62.2
23-8S	EI transition	41254	100.0
23-8S	peak I	41960	24.6
23-8S	IE transition	42345	38.1
23-8S	peak E	42968	59.8
23-8S	EI transition	44120	100.0
23-8S	peak I	44697	18.0
23-8S	IE transition	45260	35.7
23-8S	peak E	45586	45.9
23-8S	EI transition	47316	100.0
23-8S	peak I	48064	26.8
23-8S	IE transition	48420	39.6
23-8S	peak E	48682	49.0
23-8S	EI transition	50104	100.0
23-8S	peak I	50880	25.2
23-8S	IE transition	51288	38.5
23-8S	peak E	51462	44.2
23-8S	EI transition	53178	100.0
23-8S	peak I	53720	18.5
23-8S	IE transition	54381	41.1
23-8S	peak E	55335	73.6
23-8S	EI transition	56108	100.0
23-8S	peak I	56656	18.6
23-8S	IE transition	57194	37.0
23-8S	peak E	57607	51.0
23-8S	EI transition	59047	100.0

22-8S	peak I	52895	23.0
22-8S	IE transition	53265	33.9
22-8S	peak E	54033	56.5
22-8S	EI transition	55512	100.0
22-8S	peak I	56088	17.3
22-8S	IE transition	56673	34.9
22-8S	peak E	58383	86.3
22-8S	EI transition	58840	100.0

name, session	parameter	time (ms)	ratio (%)
24-1S	EI transition	32184	0.0
24-1S	peak I	32518	7.6
24-1S	IE transition	33432	28.5
24-1S	peak E	33772	36.3
24-1S	EI transition	36558	100.0
24-1S	peak I	36932	13.6
24-1S	IE transition	37742	42.9
24-1S	peak E	37987	51.8
24-1S	EI transition	39317	100.0
24-1S	peak I	39511	6.0
24-1S	IE transition	40533	37.8
24-1S	peak E	40775	45.4
24-1S	EI transition	42531	100.0
24-1S	peak I	42806	8.5
24-1S	IE transition	43671	35.0
24-1S	peak E	43883	41.5
24-1S	EI transition	45785	100.0
24-1S	peak I	46109	9.8
24-1S	IE transition	47062	38.7
24-1S	peak E	47269	44.9
24-1S	EI transition	49088	100.0
24-1S	peak I	49397	9.4
24-1S	IE transition	50397	39.9
24-1S	peak E	50651	47.7
24-1S	EI transition	52367	100.0
24-1S	peak I	52907	13.5
24-1S	IE transition	53785	35.5
24-1S	peak E	53973	40.2
24-1S	EI transition	56360	100.0
24-1S	peak I	56639	7.5
24-1S	IE transition	57624	34.1
24-1S	peak E	57915	42.0
24-1S	EI transition	60065	100.0
24-2S	EI transition	32143	0.0
24-2S	peak I	32512	11.6
24-2S	IE transition	33411	40.0
24-2S	peak E	33696	49.0
24-2S	EI transition	35315	100.0
24-2S	peak I	35646	10.7
24-2S	IE transition	36536	39.5
24-2S	peak E	36842	49.4
24-2S	EI transition	38407	100.0
24-2S	peak I	38770	8.6
24-2S	IE transition	39635	29.1
24-2S	peak E	39805	33.1
24-2S	EI transition	42629	100.0
24-2S	peak I	42978	11.7
24-2S	IE transition	43912	43.1
24-2S	peak E	44259	54.7
24-2S	EI transition	45609	100.0
24-2S	peak I	45997	11.9
24-2S	IE transition	46810	36.9
24-2S	peak E	47027	43.6
24-2S	EI transition	48859	100.0
24-2S	peak I	49274	10.3
24-2S	IE transition	50168	32.4
24-2S	peak E	50399	38.2

name, session	parameter	time (ms)	ratio (%)
26-1S	EI transition	29791	0.0
26-1S	peak I	30453	22.5
26-1S	IE transition	30952	39.5
26-1S	peak E	31117	45.1
26-1S	EI transition	32732	100.0
26-1S	peak I	33224	17.4
26-1S	IE transition	33860	39.8
26-1S	peak E	34211	52.2
26-1S	EI transition	35563	100.0
26-1S	peak I	36033	18.1
26-1S	IE transition	36611	40.3
26-1S	peak E	36855	49.7
26-1S	EI transition	38165	100.0
26-1S	peak I	38627	16.6
26-1S	IE transition	39239	38.6
26-1S	peak E	39709	55.5
26-1S	EI transition	40949	100.0
26-1S	peak I	41520	19.6
26-1S	IE transition	42032	37.2
26-1S	peak E	42296	46.3
26-1S	EI transition	43857	100.0
26-1S	peak I	44456	21.2
26-1S	IE transition	44866	35.7
26-1S	peak E	45093	43.7
26-1S	EI transition	46687	100.0
26-1S	peak I	47156	15.0
26-1S	IE transition	47784	35.2
26-1S	peak E	47998	42.0
26-1S	EI transition	49805	100.0
26-1S	peak I	50338	17.2
26-1S	IE transition	50862	34.1
26-1S	peak E	51093	41.6
26-1S	EI transition	52903	100.0
26-1S	peak I	53324	12.2
26-1S	IE transition	53955	30.5
26-1S	peak E	54270	39.6
26-1S	EI transition	56356	100.0
26-1S	peak I	56836	15.4
26-1S	IE transition	57440	34.7
26-1S	peak E	57732	44.1
26-1S	EI transition	59476	100.0
26-4S	EI transition	31171	0.0
26-4S	peak I	31726	21.5
26-4S	IE transition	32146	37.8
26-4S	peak E	32364	46.2
26-4S	EI transition	33753	100.0
26-4S	peak I	34215	15.8
26-4S	IE transition	34763	34.5
26-4S	peak E	35024	43.4
26-4S	EI transition	36681	100.0
26-4S	peak I	37146	15.2
26-4S	IE transition	37624	30.8
26-4S	peak E	38001	43.2
26-4S	EI transition	39739	100.0
26-4S	peak I	40216	13.6
26-4S	IE transition	40907	33.4
26-4S	peak E	41154	40.5

24-2S	EI transition	52893	100.0
24-2S	peak I	53239	9.8
24-2S	IE transition	54186	36.5
24-2S	peak E	54472	44.5
24-2S	EI transition	56438	100.0
24-2S	peak I	56784	9.1
24-2S	IE transition	57678	32.6
24-2S	peak E	58006	41.3
24-2S	EI transition	60237	100.0
24-5S	EI transition	32498	0.0
24-5S	peak I	32975	8.6
24-5S	IE transition	33812	23.7
24-5S	peak E	34155	29.9
24-5S	EI transition	38042	100.0
24-5S	peak I	38547	14.1
24-5S	IE transition	39402	38.1
24-5S	peak E	39686	46.0
24-5S	EI transition	41613	100.0
24-5S	peak I	41997	7.6
24-5S	IE transition	42884	25.1
24-5S	peak E	43068	28.7
24-5S	EI transition	46685	100.0
24-5S	peak I	47361	21.5
24-5S	IE transition	47937	39.8
24-5S	peak E	48179	47.5
24-5S	EI transition	49830	100.0
24-5S	peak I	50160	9.2
24-5S	IE transition	51138	36.4
24-5S	peak E	51420	44.2
24-5S	EI transition	53428	100.0
24-5S	peak I	53812	11.1
24-5S	IE transition	54753	38.4
24-5S	peak E	55349	55.7
24-5S	EI transition	56878	100.0
24-5S	peak I	57356	14.2
24-5S	IE transition	58041	34.5
24-5S	peak E	58353	43.8
24-5S	EI transition	60246	100.0
24-8S	EI transition	29230	0.0
24-8S	peak I	29579	8.5
24-8S	IE transition	30529	31.5
24-8S	peak E	33139	94.7
24-8S	EI transition	33359	100.0
24-8S	peak I	33554	4.6
24-8S	IE transition	34659	30.8
24-8S	peak E	34833	34.9
24-8S	EI transition	37583	100.0
24-8S	peak I	37914	7.4
24-8S	IE transition	38879	29.0
24-8S	peak E	39202	36.3
24-8S	EI transition	42047	100.0
24-8S	peak I	43576	26.7
24-8S	IE transition	44381	40.8
24-8S	peak E	44560	43.9
24-8S	EI transition	47768	100.0
24-8S	peak I	48078	6.9
24-8S	IE transition	49036	28.2
24-8S	peak E	49411	36.6
24-8S	EI transition	52263	100.0
26-4S	EI transition	43234	100.0
26-4S	peak I	43626	12.0
26-4S	IE transition	44296	32.5
26-4S	peak E	44508	39.0
26-4S	EI transition	46500	100.0
26-4S	peak I	47018	17.4
26-4S	IE transition	47644	38.3
26-4S	peak E	47842	45.0
26-4S	EI transition	49484	100.0
26-4S	peak I	49948	14.0
26-4S	IE transition	50606	33.8
26-4S	peak E	50788	39.3
26-4S	EI transition	52800	100.0
26-4S	peak I	53194	12.8
26-4S	IE transition	53857	34.4
26-4S	peak E	54147	43.9
26-4S	EI transition	55871	100.0
26-4S	peak I	56314	14.4
26-4S	IE transition	56925	34.3
26-4S	peak E	57154	41.8
26-4S	EI transition	58942	100.0
26-7S	EI transition	31426	0.0
26-7S	peak I	31814	11.8
26-7S	IE transition	32524	33.5
26-7S	peak E	32799	41.8
26-7S	EI transition	34710	100.0
26-7S	peak I	35121	12.0
26-7S	IE transition	35767	31.0
26-7S	peak E	35928	35.7
26-7S	EI transition	38125	100.0
26-7S	peak I	38555	12.7
26-7S	IE transition	39288	34.3
26-7S	peak E	39475	39.9
26-7S	EI transition	41511	100.0
26-7S	peak I	41939	14.0
26-7S	IE transition	42626	36.5
26-7S	peak E	43134	53.2
26-7S	EI transition	44564	100.0
26-7S	peak I	44993	15.5
26-7S	IE transition	45683	40.4
26-7S	peak E	45887	47.7
26-7S	EI transition	47335	100.0
26-7S	peak I	47786	17.1
26-7S	IE transition	48396	40.2
26-7S	peak E	49203	70.8
26-7S	EI transition	49974	100.0
26-7S	peak I	50402	14.5
26-7S	IE transition	51101	38.3
26-7S	peak E	51254	43.4
26-7S	EI transition	52920	100.0
26-7S	peak I	53364	14.2
26-7S	IE transition	54010	34.8
26-7S	peak E	54173	40.0
26-7S	EI transition	56052	100.0
26-7S	peak I	56488	12.5
26-7S	IE transition	57166	32.0
26-7S	peak E	57380	38.1
26-7S	EI transition	59533	100.0
26-8S	EI transition	30654	0.0

24-8S	peak I	52776	14.3
24-8S	IE transition	53600	37.3
24-8S	peak E	53782	42.4
24-8S	EI transition	55843	100.0
24-8S	peak I	56241	10.7
24-8S	IE transition	57143	35.0
24-8S	peak E	57514	45.0
24-8S	EI transition	59556	100.0

26-8S	peak I	31208	22.6
26-8S	IE transition	31725	43.7
26-8S	peak E	31994	54.8
26-8S	EI transition	33102	100.0
26-8S	peak I	33493	14.9
26-8S	IE transition	34158	40.3
26-8S	peak E	34346	47.5
26-8S	EI transition	35720	100.0
26-8S	peak I	36136	17.0
26-8S	IE transition	36747	42.0
26-8S	peak E	37313	65.1
26-8S	EI transition	38167	100.0
26-8S	peak I	38555	13.8
26-8S	IE transition	39193	36.5
26-8S	peak E	39344	41.9
26-8S	EI transition	40977	100.0
26-8S	peak I	41415	15.8
26-8S	IE transition	42047	38.5
26-8S	peak E	42269	46.5
26-8S	EI transition	43755	100.0
26-8S	peak I	44110	13.3
26-8S	IE transition	44785	38.6
26-8S	peak E	44934	44.2
26-8S	EI transition	46425	100.0
26-8S	peak I	46771	13.4
26-8S	IE transition	47357	36.2
26-8S	peak E	47546	43.5
26-8S	EI transition	49001	100.0
26-8S	peak I	49304	11.4
26-8S	IE transition	49998	37.5
26-8S	peak E	50134	42.6
26-8S	EI transition	51661	100.0
26-8S	peak I	52087	16.3
26-8S	IE transition	52773	42.4
26-8S	peak E	53121	55.7
26-8S	EI transition	54281	100.0
26-8S	peak I	54696	15.9
26-8S	IE transition	55344	40.7
26-8S	peak E	55553	48.7
26-8S	EI transition	56894	100.0
26-8S	peak I	57317	17.0
26-8S	IE transition	57923	41.2
26-8S	peak E	58172	51.2
26-8S	EI transition	59389	100.0

name, session	parameter	time (ms)	ratio (%)
27-1S	EI transition	32034	0.0
27-1S	peak I	32658	23.2
27-1S	IE transition	33005	36.1
27-1S	peak E	33321	47.8
27-1S	EI transition	34725	100.0
27-1S	peak I	35050	10.8
27-1S	IE transition	35746	34.0
27-1S	peak E	36048	44.1
27-1S	EI transition	37726	100.0
27-1S	peak I	38018	9.4
27-1S	IE transition	38838	35.9
27-1S	peak E	39082	43.8
27-1S	EI transition	40825	100.0
27-1S	peak I	41159	11.6
27-1S	IE transition	41927	38.3
27-1S	peak E	42200	47.8
27-1S	EI transition	43702	100.0
27-1S	peak I	44137	12.7
27-1S	IE transition	44861	34.0
27-1S	peak E	45230	44.8
27-1S	EI transition	47114	100.0
27-1S	peak I	47577	13.7
27-1S	IE transition	48278	34.4
27-1S	peak E	48719	47.5
27-1S	EI transition	50495	100.0
27-1S	peak I	50827	11.6
27-1S	IE transition	51574	37.6
27-1S	peak E	51859	47.5
27-1S	EI transition	53367	100.0
27-1S	peak I	53794	15.0
27-1S	IE transition	54460	38.4
27-1S	peak E	54736	48.0
27-1S	EI transition	56217	100.0
27-1S	peak I	56673	14.2
27-1S	IE transition	57337	35.0
27-1S	peak E	57618	43.7
27-1S	EI transition	59421	100.0
27-1S	peak I	59867	15.1
27-1S	IE transition	60581	39.3
27-3S	EI transition	32149	0.0
27-3S	peak I	32537	14.4
27-3S	IE transition	33192	38.7
27-3S	peak E	33520	50.9
27-3S	EI transition	34843	100.0
27-3S	peak I	35340	15.1
27-3S	IE transition	35956	33.9
27-3S	peak E	36339	45.6
27-3S	EI transition	38125	100.0
27-3S	peak I	38423	9.5
27-3S	IE transition	39104	31.4
27-3S	peak E	39442	42.2
27-3S	EI transition	41246	100.0
27-3S	peak I	41665	13.1
27-3S	IE transition	42364	35.0
27-3S	peak E	42622	43.1
27-3S	EI transition	44442	100.0
27-3S	peak I	44961	17.1

name, session	parameter	time (ms)	ratio (%)
28-1S	EI transition	31649	0.0
28-1S	peak I	32087	14.9
28-1S	IE transition	32744	37.2
28-1S	peak E	32868	41.4
28-1S	EI transition	34596	100.0
28-1S	peak I	35018	14.4
28-1S	IE transition	35626	35.1
28-1S	peak E	35903	44.5
28-1S	EI transition	37532	100.0
28-1S	peak I	38049	17.0
28-1S	IE transition	38611	35.5
28-1S	peak E	39141	52.9
28-1S	EI transition	40573	100.0
28-1S	peak I	41289	23.1
28-1S	IE transition	41684	35.8
28-1S	peak E	41923	43.5
28-1S	EI transition	43674	100.0
28-1S	peak I	44329	23.7
28-1S	IE transition	44709	37.4
28-1S	peak E	45054	49.8
28-1S	EI transition	46443	100.0
28-1S	peak I	46839	12.4
28-1S	IE transition	47427	30.7
28-1S	peak E	48752	72.0
28-1S	EI transition	49649	100.0
28-1S	peak I	50022	11.2
28-1S	IE transition	50631	29.5
28-1S	peak E	51103	43.7
28-1S	EI transition	52978	100.0
28-1S	peak I	53456	15.8
28-1S	IE transition	53891	30.2
28-1S	peak E	54071	36.2
28-1S	EI transition	56001	100.0
28-1S	peak I	56494	16.7
28-1S	IE transition	57043	35.2
28-1S	peak E	57265	42.7
28-1S	EI transition	58959	100.0
28-3S	EI transition	32026	0.0
28-3S	peak I	32484	15.2
28-3S	IE transition	33056	34.1
28-3S	peak E	33283	41.6
28-3S	EI transition	35048	100.0
28-3S	peak I	35642	19.9
28-3S	IE transition	36124	36.1
28-3S	peak E	36397	45.3
28-3S	EI transition	38029	100.0
28-3S	peak I	38550	18.2
28-3S	IE transition	39083	36.8
28-3S	peak E	39309	44.6
28-3S	EI transition	40897	100.0
28-3S	peak I	41358	13.0
28-3S	IE transition	41927	29.1
28-3S	peak E	42105	34.1
28-3S	EI transition	44435	100.0
28-3S	peak I	44883	40.4
28-3S	IE transition	44961	47.5
28-3S	peak E	45247	73.3

27-3S	IE transition	45623	38.8
27-3S	peak E	45917	48.5
27-3S	EI transition	47483	100.0
27-3S	peak I	47816	12.2
27-3S	IE transition	48591	40.7
27-3S	peak E	48926	53.0
27-3S	EI transition	50205	100.0
27-3S	peak I	50565	11.8
27-3S	IE transition	51255	34.5
27-3S	peak E	51554	44.4
27-3S	EI transition	53245	100.0
27-3S	peak I	53555	11.1
27-3S	IE transition	54270	36.5
27-3S	peak E	54544	46.3
27-3S	EI transition	56052	100.0
27-3S	peak I	56568	15.8
27-3S	IE transition	57273	37.3
27-3S	peak E	57595	47.1
27-3S	EI transition	59325	100.0
27-5S	EI transition	32249	0.0
27-5S	peak I	32610	11.7
27-5S	IE transition	33279	33.3
27-5S	peak E	33699	46.8
27-5S	EI transition	35345	100.0
27-5S	peak I	35675	12.1
27-5S	IE transition	36438	40.1
27-5S	peak E	36837	54.8
27-5S	EI transition	38068	100.0
27-5S	peak I	38506	14.9
27-5S	IE transition	39188	38.1
27-5S	peak E	39597	52.0
27-5S	EI transition	41010	100.0
27-5S	peak I	41520	16.3
27-5S	IE transition	42113	35.2
27-5S	peak E	42530	48.5
27-5S	EI transition	44141	100.0
27-5S	peak I	44678	18.0
27-5S	IE transition	45284	38.4
27-5S	peak E	45639	50.3
27-5S	EI transition	47120	100.0
27-5S	peak I	47480	13.0
27-5S	IE transition	48187	38.6
27-5S	peak E	48574	52.6
27-5S	EI transition	49883	100.0
27-5S	peak I	50280	13.3
27-5S	IE transition	51026	38.2
27-5S	peak E	51512	54.4
27-5S	EI transition	52875	100.0
27-5S	peak I	53201	10.3
27-5S	IE transition	53975	34.6
27-5S	peak E	54632	55.3
27-5S	EI transition	56052	100.0
27-5S	peak I	56462	10.6
27-5S	IE transition	58977	75.5
27-5S	peak E	59147	79.9
27-8S	EI transition	29439	0.0
27-8S	peak I	30296	23.2
27-8S	IE transition	30669	33.3
27-8S	peak E	30954	41.0

28-3S	EI transition	45543	100.0
28-3S	peak I	45830	10.6
28-3S	IE transition	46518	35.9
28-3S	peak E	46785	45.7
28-3S	EI transition	48259	100.0
28-3S	peak I	48563	11.7
28-3S	IE transition	49203	36.5
28-3S	peak E	49488	47.5
28-3S	EI transition	50847	100.0
28-3S	peak I	51209	13.3
28-3S	IE transition	51915	39.2
28-3S	peak E	52454	59.0
28-3S	EI transition	53570	100.0
28-3S	peak I	53969	13.9
28-3S	IE transition	54564	34.7
28-3S	peak E	54830	43.9
28-3S	EI transition	56438	100.0
28-3S	peak I	56747	11.0
28-3S	IE transition	57502	37.9
28-3S	peak E	57989	55.3
28-3S	EI transition	59244	100.0
28-5S	EI transition	32395	0.0
28-5S	peak I	32977	19.7
28-5S	IE transition	33494	37.3
28-5S	peak E	34124	58.7
28-5S	EI transition	35342	100.0
28-5S	peak I	35864	17.3
28-5S	IE transition	36345	33.2
28-5S	peak E	36776	47.5
28-5S	EI transition	38361	100.0
28-5S	peak I	38929	20.7
28-5S	IE transition	39391	37.5
28-5S	peak E	39667	47.6
28-5S	EI transition	41105	100.0
28-5S	peak I	41654	20.3
28-5S	IE transition	42093	36.6
28-5S	peak E	42442	49.5
28-5S	EI transition	43807	100.0
28-5S	peak I	44260	14.6
28-5S	IE transition	44811	32.4
28-5S	peak E	45155	43.5
28-5S	EI transition	46907	100.0
28-5S	peak I	47563	21.7
28-5S	IE transition	48058	38.0
28-5S	peak E	48493	52.4
28-5S	EI transition	49934	100.0
28-5S	peak I	50688	24.3
28-5S	IE transition	51027	35.2
28-5S	peak E	51444	48.6
28-5S	EI transition	53042	100.0
28-5S	peak I	53629	18.6
28-5S	IE transition	54154	35.3
28-5S	peak E	54498	46.2
28-5S	EI transition	56195	100.0
28-5S	peak I	56780	21.5
28-5S	IE transition	57216	37.5
28-5S	peak E	57887	62.1
28-5S	EI transition	58920	100.0
28-8S	EI transition	29403	0.0

27-8S	EI transition	33131	100.0
27-8S	peak I	33470	11.2
27-8S	IE transition	34240	36.8
27-8S	peak E	34608	49.0
27-8S	EI transition	36147	100.0
27-8S	peak I	36615	16.5
27-8S	IE transition	37256	39.0
27-8S	peak E	37611	51.5
27-8S	EI transition	38990	100.0
27-8S	peak I	39338	10.9
27-8S	IE transition	40131	35.8
27-8S	peak E	40391	44.0
27-8S	EI transition	42173	100.0
27-8S	peak I	42474	11.4
27-8S	IE transition	43328	43.9
27-8S	peak E	43756	60.1
27-8S	EI transition	44806	100.0
27-8S	peak I	45133	13.5
27-8S	IE transition	45916	45.9
27-8S	peak E	46948	88.5
27-8S	EI transition	47225	100.0
27-8S	peak I	47499	5.8
27-8S	IE transition	48405	24.8
27-8S	peak E	48754	32.1
27-8S	EI transition	51986	100.0
27-8S	peak I	52303	9.1
27-8S	IE transition	53065	31.1
27-8S	peak E	53453	42.3
27-8S	EI transition	55456	100.0
27-8S	peak I	55740	9.4
27-8S	IE transition	56521	35.3
27-8S	peak E	56918	48.5
27-8S	EI transition	58472	100.0

28-8S	peak I	29904	15.5
28-8S	IE transition	30524	34.7
28-8S	peak E	30763	42.1
28-8S	EI transition	32632	100.0
28-8S	peak I	33134	17.2
28-8S	IE transition	33653	35.1
28-8S	peak E	33986	46.5
28-8S	EI transition	35544	100.0
28-8S	peak I	36257	23.2
28-8S	IE transition	36668	36.6
28-8S	peak E	38233	87.6
28-8S	EI transition	38612	100.0
28-8S	peak I	39131	17.2
28-8S	IE transition	39675	35.3
28-8S	peak E	40888	75.5
28-8S	EI transition	41627	100.0
28-8S	peak I	41955	11.7
28-8S	IE transition	42631	35.8
28-8S	peak E	42811	42.2
28-8S	EI transition	44431	100.0
28-8S	peak I	44922	16.2
28-8S	IE transition	45506	35.4
28-8S	peak E	46034	52.8
28-8S	EI transition	47465	100.0
28-8S	peak I	48045	21.1
28-8S	IE transition	48447	35.8
28-8S	peak E	48766	47.4
28-8S	EI transition	50207	100.0
28-8S	peak I	50678	15.8
28-8S	IE transition	51227	34.2
28-8S	peak E	52130	64.5
28-8S	EI transition	53190	100.0
28-8S	peak I	53787	20.8
28-8S	IE transition	54230	36.2
28-8S	peak E	55059	65.1
28-8S	EI transition	56062	100.0
28-8S	peak I	56487	14.1
28-8S	IE transition	57046	32.6
28-8S	peak E	57382	43.8
28-8S	EI transition	59079	100.0
28-8S	peak I	59758	22.8
28-8S	IE transition	60196	37.5

name, session	parameter	time (ms)	ratio (%)
29-1S	EI transition	29662	0.0
29-1S	peak I	30143	18.2
29-1S	IE transition	30848	44.9
29-1S	peak E	31165	56.9
29-1S	EI transition	32304	100.0
29-1S	peak I	32808	18.8
29-1S	IE transition	33476	43.7
29-1S	peak E	34454	80.2
29-1S	EI transition	34986	100.0
29-1S	peak I	35491	18.6
29-1S	IE transition	36236	46.1
29-1S	peak E	37295	85.2
29-1S	EI transition	37696	100.0
29-1S	peak I	38531	29.8
29-1S	IE transition	38998	46.5
29-1S	peak E	40014	82.8
29-1S	EI transition	40495	100.0
29-1S	peak I	41231	23.8
29-1S	IE transition	41816	42.7
29-1S	peak E	42637	69.2
29-1S	EI transition	43589	100.0
29-1S	peak I	44243	16.2
29-1S	IE transition	44997	34.8
29-1S	peak E	46108	62.3
29-1S	EI transition	47635	100.0
29-1S	peak I	48235	19.5
29-1S	IE transition	48970	43.3
29-1S	peak E	49867	72.5
29-1S	EI transition	50715	100.0
29-1S	peak I	51174	19.3
29-1S	IE transition	51816	46.2
29-1S	peak E	52127	59.3
29-1S	EI transition	53098	100.0
29-1S	peak I	53518	16.7
29-1S	IE transition	54356	50.0
29-1S	peak E	55135	81.0
29-1S	EI transition	55614	100.0
29-1S	peak I	55730	4.3
29-1S	IE transition	56577	35.6
29-1S	peak E	57431	67.1
29-1S	EI transition	58320	100.0
29-1S	peak I	59202	32.3
29-1S	IE transition	59362	38.2
29-4S	EI transition	30511	0.0
29-4S	peak I	31288	28.7
29-4S	IE transition	31587	39.7
29-4S	peak E	31884	50.7
29-4S	EI transition	33220	100.0
29-4S	peak I	33712	19.2
29-4S	IE transition	34274	41.0
29-4S	peak E	35418	85.6
29-4S	EI transition	35788	100.0
29-4S	peak I	36368	19.0
29-4S	IE transition	36832	34.2
29-4S	peak E	37812	66.3
29-4S	EI transition	38842	100.0
29-4S	peak I	39480	19.7

name, session	parameter	time (ms)	ratio (%)
30-1S	EI transition	32333	0.0
30-1S	peak I	32798	9.1
30-1S	IE transition	33672	26.1
30-1S	peak E	33995	32.4
30-1S	EI transition	37463	100.0
30-1S	peak I	37847	7.8
30-1S	IE transition	38890	29.0
30-1S	peak E	39392	39.2
30-1S	EI transition	42382	100.0
30-1S	peak I	42940	10.4
30-1S	IE transition	43609	22.9
30-1S	peak E	44152	33.1
30-1S	EI transition	47729	100.0
30-1S	peak I	48202	8.5
30-1S	IE transition	49121	25.0
30-1S	peak E	49445	30.9
30-1S	EI transition	53288	100.0
30-1S	peak I	53767	9.3
30-1S	IE transition	54604	25.4
30-1S	peak E	55045	34.0
30-1S	EI transition	58463	100.0
30-2S	EI transition	30439	0.0
30-2S	peak I	30779	10.1
30-2S	IE transition	31564	33.4
30-2S	peak E	31939	44.6
30-2S	EI transition	33803	100.0
30-2S	peak I	34039	7.3
30-2S	IE transition	35014	37.6
30-2S	peak E	35390	49.3
30-2S	EI transition	37021	100.0
30-2S	peak I	37398	13.8
30-2S	IE transition	38162	41.7
30-2S	peak E	38699	61.4
30-2S	EI transition	39755	100.0
30-2S	peak I	39977	4.7
30-2S	IE transition	40770	21.3
30-2S	peak E	41498	36.6
30-2S	EI transition	44513	100.0
30-2S	peak I	45244	13.9
30-2S	IE transition	46127	30.6
30-2S	peak E	46638	40.3
30-2S	EI transition	49788	100.0
30-2S	peak I	50126	6.5
30-2S	IE transition	51208	27.5
30-2S	peak E	51684	36.7
30-2S	EI transition	54949	100.0
30-2S	peak I	55514	9.1
30-2S	IE transition	56529	25.4
30-2S	peak E	57132	35.1
30-2S	EI transition	61172	100.0
30-6S	EI transition	30909	0.0
30-6S	peak I	31204	8.8
30-6S	IE transition	31870	28.7
30-6S	peak E	32273	40.7
30-6S	EI transition	34261	100.0
30-6S	peak I	34649	13.0
30-6S	IE transition	35215	31.9

29-4S	IE transition	40027	36.6
29-4S	peak E	41333	76.9
29-4S	EI transition	42081	100.0
29-4S	peak I	42697	18.0
29-4S	IE transition	43402	38.7
29-4S	peak E	44273	64.2
29-4S	EI transition	45497	100.0
29-4S	peak I	45996	18.3
29-4S	IE transition	46621	41.2
29-4S	peak E	46819	48.4
29-4S	EI transition	48228	100.0
29-4S	peak I	49210	26.5
29-4S	IE transition	49687	39.3
29-4S	peak E	50239	54.2
29-4S	EI transition	51936	100.0
29-4S	peak I	52267	14.9
29-4S	IE transition	52793	38.5
29-4S	peak E	53178	55.8
29-4S	EI transition	54162	100.0
29-4S	peak I	55167	32.6
29-4S	IE transition	55420	40.9
29-4S	peak E	55952	58.1
29-4S	EI transition	57241	100.0
29-4S	peak I	58158	26.7
29-4S	IE transition	58615	40.0
29-4S	peak E	59061	53.0
29-4S	EI transition	60675	100.0
29-7S	EI transition	30280	0.0
29-7S	peak I	30654	14.4
29-7S	IE transition	31414	43.6
29-7S	peak E	32129	71.1
29-7S	EI transition	32882	100.0
29-7S	peak I	33344	24.2
29-7S	IE transition	33976	57.4
29-7S	peak E	34344	76.7
29-7S	EI transition	34788	100.0
29-7S	peak I	35345	19.2
29-7S	IE transition	35682	30.8
29-7S	peak E	35878	37.5
29-7S	EI transition	37693	100.0
29-7S	peak I	38266	17.9
29-7S	IE transition	38875	36.9
29-7S	peak E	39620	60.2
29-7S	EI transition	40892	100.0
29-7S	peak I	41214	8.0
29-7S	IE transition	42135	30.9
29-7S	peak E	43421	62.8
29-7S	EI transition	44919	100.0
29-7S	peak I	45384	15.0
29-7S	IE transition	46161	40.2
29-7S	peak E	46943	65.5
29-7S	EI transition	48010	100.0
29-7S	peak I	48360	10.3
29-7S	IE transition	49353	39.5
29-7S	peak E	49871	54.8
29-7S	EI transition	51409	100.0
29-7S	peak I	52365	28.8
29-7S	IE transition	52835	43.0
29-7S	peak E	53365	59.0

30-6S	peak E	35540	42.8
30-6S	EI transition	37251	100.0
30-6S	peak I	37548	13.1
30-6S	IE transition	37875	27.6
30-6S	peak E	38473	54.0
30-6S	EI transition	39515	100.0
30-6S	peak I	39867	10.6
30-6S	IE transition	40770	37.9
30-6S	peak E	41323	54.6
30-6S	EI transition	42829	100.0
30-6S	peak I	43116	8.5
30-6S	IE transition	44094	37.4
30-6S	peak E	44361	45.3
30-6S	EI transition	46210	100.0
30-6S	peak I	46481	6.6
30-6S	IE transition	47094	21.5
30-6S	peak E	47776	38.2
30-6S	EI transition	50313	100.0
30-6S	peak I	50613	7.2
30-6S	IE transition	51598	30.8
30-6S	peak E	51990	40.2
30-6S	EI transition	54485	100.0
30-6S	peak I	54771	7.4
30-6S	IE transition	55638	29.7
30-6S	peak E	56081	41.2
30-6S	EI transition	58361	100.0
30-6S	peak I	58618	9.6
30-6S	IE transition	58971	22.9
30-8S	EI transition	31539	0.0
30-8S	peak I	31945	8.8
30-8S	IE transition	32773	26.9
30-8S	peak E	33288	38.1
30-8S	EI transition	36128	100.0
30-8S	peak I	36521	9.5
30-8S	IE transition	37380	30.1
30-8S	peak E	37817	40.6
30-8S	EI transition	40286	100.0
30-8S	peak I	40651	8.3
30-8S	IE transition	41601	29.8
30-8S	peak E	42035	39.6
30-8S	EI transition	44706	100.0
30-8S	peak I	45140	10.4
30-8S	IE transition	46045	32.1
30-8S	peak E	46539	44.0
30-8S	EI transition	48875	100.0
30-8S	peak I	49267	6.9
30-8S	IE transition	50143	22.3
30-8S	peak E	50468	28.0
30-8S	EI transition	54558	100.0
30-8S	peak I	55015	9.6
30-8S	IE transition	55830	26.7
30-8S	peak E	56179	34.0
30-8S	EI transition	59325	100.0

29-7S	EI transition	54724	100.0
29-7S	peak I	55131	13.4
29-7S	IE transition	56054	43.8
29-7S	peak E	56771	67.5
29-7S	EI transition	57758	100.0
29-7S	peak I	58497	20.2
29-7S	IE transition	59170	38.6
29-8S	EI transition	31781	0.0
29-8S	peak I	32258	16.2
29-8S	IE transition	33032	42.4
29-8S	peak E	33848	70.1
29-8S	EI transition	34732	100.0
29-8S	peak I	35243	19.8
29-8S	IE transition	35932	46.5
29-8S	peak E	36915	84.7
29-8S	EI transition	37310	100.0
29-8S	peak I	37794	20.4
29-8S	IE transition	38275	40.8
29-8S	peak E	38648	56.5
29-8S	EI transition	39678	100.0
29-8S	peak I	40380	21.4
29-8S	IE transition	40832	35.2
29-8S	peak E	41424	53.3
29-8S	EI transition	42953	100.0
29-8S	peak I	43512	19.2
29-8S	IE transition	44254	44.8
29-8S	peak E	44562	55.4
29-8S	EI transition	45859	100.0
29-8S	peak I	46911	33.3
29-8S	IE transition	47243	43.8
29-8S	peak E	47505	52.1
29-8S	EI transition	49020	100.0
29-8S	peak I	49450	15.3
29-8S	IE transition	50160	40.7
29-8S	peak E	50352	47.5
29-8S	EI transition	51824	100.0
29-8S	peak I	52633	25.1
29-8S	IE transition	53208	42.9
29-8S	peak E	53847	62.7
29-8S	EI transition	55052	100.0
29-8S	peak I	55544	17.4
29-8S	IE transition	56315	44.6
29-8S	peak E	57173	74.9
29-8S	EI transition	57883	100.0
29-8S	peak I	58424	14.9
29-8S	IE transition	58978	30.2
29-8S	peak E	59255	37.8

name, session	parameter	time (ms)	ratio (%)
31-1S	EI transition	31850	0.0
31-1S	peak I	32577	12.7
31-1S	IE transition	33427	27.6
31-1S	peak E	34214	41.3
31-1S	EI transition	37573	100.0
31-1S	peak I	38247	13.5
31-1S	IE transition	39149	31.6
31-1S	peak E	40658	61.8
31-1S	EI transition	42563	100.0
31-1S	peak I	43115	12.3
31-1S	IE transition	44043	32.9
31-1S	peak E	44323	39.1
31-1S	EI transition	47063	100.0
31-1S	peak I	47763	14.8
31-1S	IE transition	48650	33.7
31-1S	peak E	49518	52.1
31-1S	EI transition	51778	100.0
31-1S	peak I	52519	16.9
31-1S	IE transition	53230	33.1
31-1S	peak E	53898	48.3
31-1S	EI transition	56165	100.0
31-1S	peak I	56993	18.3
31-1S	IE transition	57789	35.8
31-1S	peak E	58797	58.1
31-1S	EI transition	60696	100.0
31-4S	EI transition	28943	0.0
31-4S	peak I	29574	13.4
31-4S	IE transition	30638	36.1
31-4S	peak E	31684	58.4
31-4S	EI transition	33637	100.0
31-4S	peak I	34638	22.5
31-4S	IE transition	35288	37.0
31-4S	peak E	35638	44.9
31-4S	EI transition	38094	100.0
31-4S	peak I	38536	10.0
31-4S	IE transition	39579	33.6
31-4S	peak E	41773	83.3
31-4S	EI transition	42511	100.0
31-4S	peak I	43199	16.6
31-4S	IE transition	44140	39.2
31-4S	peak E	44537	48.8
31-4S	EI transition	46663	100.0
31-4S	peak I	47229	10.6
31-4S	IE transition	48132	27.4
31-4S	peak E	49575	54.3
31-4S	EI transition	52024	100.0
31-4S	peak I	52890	20.8
31-4S	IE transition	53426	33.6
31-4S	peak E	54135	50.6
31-4S	EI transition	56193	100.0
31-4S	peak I	56749	14.0
31-4S	IE transition	57765	39.5
31-4S	peak E	58306	53.1
31-4S	EI transition	60173	100.0
31-6S	EI transition	29873	0.0
31-6S	peak I	30591	17.3
31-6S	IE transition	31271	33.7

name, session	parameter	time (ms)	ratio (%)
32-1S	EI transition	31260	0.0
32-1S	peak I	31618	9.6
32-1S	IE transition	32393	30.3
32-1S	peak E	32595	35.7
32-1S	EI transition	34996	100.0
32-1S	peak I	35514	16.4
32-1S	IE transition	36114	35.4
32-1S	peak E	36598	50.7
32-1S	EI transition	38153	100.0
32-1S	peak I	38543	10.0
32-1S	IE transition	39171	26.2
32-1S	peak E	39508	34.9
32-1S	EI transition	42040	100.0
32-1S	peak I	42540	10.3
32-1S	IE transition	43032	20.5
32-1S	peak E	46801	98.3
32-1S	EI transition	46882	100.0
32-1S	peak I	47109	4.0
32-1S	IE transition	47926	18.5
32-1S	peak E	48072	21.1
32-1S	EI transition	52529	100.0
32-1S	peak I	52932	10.9
32-1S	IE transition	53543	27.3
32-1S	peak E	54389	50.2
32-1S	EI transition	56237	100.0
32-1S	peak I	56568	11.4
32-1S	IE transition	57330	37.5
32-1S	peak E	57523	44.1
32-1S	EI transition	59152	100.0
32-3S	EI transition	31680	0.0
32-3S	peak I	32193	16.7
32-3S	IE transition	32681	32.6
32-3S	peak E	33127	47.1
32-3S	EI transition	34750	100.0
32-3S	peak I	35149	11.5
32-3S	IE transition	35706	27.7
32-3S	peak E	35956	34.9
32-3S	EI transition	38207	100.0
32-3S	peak I	38737	18.2
32-3S	IE transition	39260	36.1
32-3S	peak E	39476	43.6
32-3S	EI transition	41120	100.0
32-3S	peak I	41617	16.8
32-3S	IE transition	42177	35.7
32-3S	peak E	42518	47.2
32-3S	EI transition	44083	100.0
32-3S	peak I	44406	10.6
32-3S	IE transition	45069	32.3
32-3S	peak E	45466	45.3
32-3S	EI transition	47136	100.0
32-3S	peak I	47521	11.4
32-3S	IE transition	48098	28.4
32-3S	peak E	48509	40.5
32-3S	EI transition	50527	100.0
32-3S	peak I	50807	9.6
32-3S	IE transition	51583	36.4
32-3S	peak E	51818	44.4

31-6S	peak E	31606	41.7
31-6S	EI transition	34027	100.0
31-6S	peak I	34952	21.5
31-6S	IE transition	35692	38.7
31-6S	peak E	36459	56.5
31-6S	EI transition	38330	100.0
31-6S	peak I	38803	13.2
31-6S	IE transition	39634	36.4
31-6S	peak E	40487	60.1
31-6S	EI transition	41917	100.0
31-6S	peak I	42428	11.9
31-6S	IE transition	43245	30.9
31-6S	peak E	43531	37.6
31-6S	EI transition	46208	100.0
31-6S	peak I	46997	17.8
31-6S	IE transition	47683	33.2
31-6S	peak E	48097	42.6
31-6S	EI transition	50647	100.0
31-6S	peak I	51252	13.5
31-6S	IE transition	52029	30.8
31-6S	peak E	52272	36.2
31-6S	EI transition	55132	100.0
31-6S	peak I	55599	11.9
31-6S	IE transition	56595	37.1
31-6S	peak E	56855	43.7
31-6S	EI transition	59070	100.0
31-8S	EI transition	29586	0.0
31-8S	peak I	30153	12.1
31-8S	IE transition	30575	21.0
31-8S	peak E	31569	42.2
31-8S	EI transition	34287	100.0
31-8S	peak I	34831	8.7
31-8S	IE transition	35739	23.1
31-8S	peak E	37721	54.6
31-8S	EI transition	40573	100.0
31-8S	peak I	41321	13.2
31-8S	IE transition	41877	23.0
31-8S	peak E	43641	54.1
31-8S	EI transition	46242	100.0
31-8S	peak I	46979	14.4
31-8S	IE transition	47752	29.5
31-8S	peak E	47921	32.8
31-8S	EI transition	51358	100.0
31-8S	peak I	51818	9.9
31-8S	IE transition	52769	30.3
31-8S	peak E	53381	43.4
31-8S	EI transition	56020	100.0
31-8S	peak I	56777	15.5
31-8S	IE transition	57542	31.2
31-8S	peak E	58149	43.7
31-8S	EI transition	60892	100.0
32-3S	EI transition	53432	100.0
32-3S	peak I	53826	11.7
32-3S	IE transition	54444	30.0
32-3S	peak E	54760	39.4
32-3S	EI transition	56801	100.0
32-3S	peak I	57332	16.5
32-3S	IE transition	57896	34.0
32-3S	peak E	58242	44.8
32-3S	EI transition	60021	100.0
32-6S	EI transition	30686	0.0
32-6S	peak I	30943	5.8
32-6S	IE transition	31915	27.6
32-6S	peak E	32413	38.7
32-6S	EI transition	35144	100.0
32-6S	peak I	35461	6.9
32-6S	IE transition	36232	23.6
32-6S	peak E	36681	33.3
32-6S	EI transition	39762	100.0
32-6S	peak I	40046	8.6
32-6S	IE transition	40943	35.7
32-6S	peak E	41480	51.9
32-6S	EI transition	43074	100.0
32-6S	peak I	43651	23.0
32-6S	IE transition	44039	38.4
32-6S	peak E	44559	59.1
32-6S	EI transition	45585	100.0
32-6S	peak I	45972	18.1
32-6S	IE transition	46452	40.6
32-6S	peak E	46906	61.8
32-6S	EI transition	47722	100.0
32-6S	peak I	48124	12.6
32-6S	IE transition	48692	30.4
32-6S	peak E	48982	39.5
32-6S	EI transition	50912	100.0
32-6S	peak I	51196	9.0
32-6S	IE transition	51873	30.4
32-6S	peak E	52124	38.3
32-6S	EI transition	54072	100.0
32-6S	peak I	54429	12.6
32-6S	IE transition	54873	28.3
32-6S	peak E	55272	42.3
32-6S	EI transition	56906	100.0
32-6S	peak I	57274	12.8
32-6S	IE transition	57887	34.2
32-6S	peak E	58286	48.1
32-6S	EI transition	59775	100.0
32-8S	EI transition	31543	0.0
32-8S	peak I	31911	12.5
32-8S	IE transition	32526	33.5
32-8S	peak E	33932	81.3
32-8S	EI transition	34480	100.0
32-8S	peak I	34854	13.5
32-8S	IE transition	35304	29.8
32-8S	peak E	35863	49.9
32-8S	EI transition	37249	100.0
32-8S	peak I	37717	14.5
32-8S	IE transition	38081	25.7
32-8S	peak E	38364	34.5
32-8S	EI transition	40482	100.0

32-8S	peak I	40995	17.6
32-8S	IE transition	41565	37.1
32-8S	peak E	41760	43.8
32-8S	EI transition	43402	100.0
32-8S	peak I	43945	22.2
32-8S	IE transition	44391	40.4
32-8S	peak E	44603	49.0
32-8S	EI transition	45851	100.0
32-8S	peak I	46450	22.0
32-8S	IE transition	47005	42.4
32-8S	peak E	47340	54.7
32-8S	EI transition	48573	100.0
32-8S	peak I	48932	14.6
32-8S	IE transition	49552	39.9
32-8S	peak E	49786	49.4
32-8S	EI transition	51028	100.0
32-8S	peak I	51303	11.9
32-8S	IE transition	51896	37.6
32-8S	peak E	52259	53.3
32-8S	EI transition	53338	100.0
32-8S	peak I	53752	15.0
32-8S	IE transition	54287	34.4
32-8S	peak E	54670	48.3
32-8S	EI transition	56094	100.0
32-8S	peak I	56489	13.5
32-8S	IE transition	57111	34.7
32-8S	peak E	57520	48.6
32-8S	EI transition	59027	100.0

name, session	parameter	time (ms)	ratio (%)
33-1S	EI transition	30186	0.0
33-1S	peak I	31052	33.8
33-1S	IE transition	31333	44.8
33-1S	peak E	31598	55.2
33-1S	EI transition	32745	100.0
33-1S	peak I	33617	27.2
33-1S	IE transition	34016	39.6
33-1S	peak E	34293	48.3
33-1S	EI transition	35952	100.0
33-1S	peak I	36390	16.0
33-1S	IE transition	36976	37.4
33-1S	peak E	37408	53.2
33-1S	EI transition	38687	100.0
33-1S	peak I	39205	16.4
33-1S	IE transition	39728	33.0
33-1S	peak E	40050	43.2
33-1S	EI transition	41842	100.0
33-1S	peak I	42441	19.8
33-1S	IE transition	42936	36.2
33-1S	peak E	43287	47.8
33-1S	EI transition	44862	100.0
33-1S	peak I	45473	19.8
33-1S	IE transition	45956	35.5
33-1S	peak E	46328	47.6
33-1S	EI transition	47943	100.0
33-1S	peak I	48520	19.7
33-1S	IE transition	49135	40.7
33-1S	peak E	49443	51.2
33-1S	EI transition	50873	100.0
33-1S	peak I	51483	19.9
33-1S	IE transition	52024	37.6
33-1S	peak E	52524	54.0
33-1S	EI transition	53933	100.0
33-1S	peak I	54409	15.7
33-1S	IE transition	55027	36.2
33-1S	peak E	55632	56.2
33-1S	EI transition	56958	100.0
33-1S	peak I	57517	21.4
33-1S	IE transition	58022	40.7
33-1S	peak E	58554	61.1
33-6S	EI transition	31876	0.0
33-6S	peak I	32721	22.1
33-6S	IE transition	33148	33.3
33-6S	peak E	33377	39.3
33-6S	EI transition	35699	100.0
33-6S	peak I	36515	21.5
33-6S	IE transition	37004	34.4
33-6S	peak E	37160	38.6
33-6S	EI transition	39488	100.0
33-6S	peak I	40314	25.9
33-6S	IE transition	40661	36.7
33-6S	peak E	40956	46.0
33-6S	EI transition	42682	100.0
33-6S	peak I	43568	25.2
33-6S	IE transition	44000	37.5
33-6S	peak E	44447	50.3
33-6S	EI transition	46192	100.0

name, session	parameter	time (ms)	ratio (%)
34-1S	EI transition	32624	0.0
34-1S	peak I	33190	16.0
34-1S	IE transition	33906	36.3
34-1S	peak E	35132	71.1
34-1S	EI transition	36152	100.0
34-1S	peak I	36737	17.2
34-1S	IE transition	37448	38.1
34-1S	peak E	38809	78.1
34-1S	EI transition	39553	100.0
34-1S	peak I	40152	11.7
34-1S	IE transition	40668	21.7
34-1S	peak E	40906	26.4
34-1S	EI transition	44680	100.0
34-1S	peak I	45103	10.9
34-1S	IE transition	45903	31.4
34-1S	peak E	46991	59.4
34-1S	EI transition	48569	100.0
34-1S	peak I	49067	16.8
34-1S	IE transition	49727	39.1
34-1S	peak E	50111	52.1
34-1S	EI transition	51530	100.0
34-1S	peak I	52147	19.9
34-1S	IE transition	52620	35.1
34-1S	peak E	52891	43.8
34-1S	EI transition	54637	100.0
34-1S	peak I	55061	13.3
34-1S	IE transition	55823	37.3
34-1S	peak E	56172	48.3
34-1S	EI transition	57813	100.0
34-1S	peak I	58220	10.3
34-1S	IE transition	58769	24.2
34-1S	peak E	59046	31.2
34-3S	EI transition	32159	0.0
34-3S	peak I	32536	8.6
34-3S	IE transition	33458	29.5
34-3S	peak E	35356	72.6
34-3S	EI transition	36560	100.0
34-3S	peak I	37304	23.4
34-3S	IE transition	37724	36.7
34-3S	peak E	38255	53.4
34-3S	EI transition	39734	100.0
34-3S	peak I	40378	13.9
34-3S	IE transition	40964	26.6
34-3S	peak E	41670	41.9
34-3S	EI transition	44358	100.0
34-3S	peak I	44799	10.7
34-3S	IE transition	45521	28.2
34-3S	peak E	46373	48.9
34-3S	EI transition	48479	100.0
34-3S	peak I	48784	9.8
34-3S	IE transition	49589	35.6
34-3S	peak E	49903	45.7
34-3S	EI transition	51597	100.0
34-3S	peak I	52262	20.2
34-3S	IE transition	52743	34.8
34-3S	peak E	53069	44.7
34-3S	EI transition	54887	100.0

33-6S	peak I	46686	12.9	34-3S	peak I	55904	30.7
33-6S	IE transition	47349	30.2	34-3S	IE transition	56171	38.7
33-6S	peak E	47661	38.4	34-3S	peak E	56611	52.0
33-6S	EI transition	50022	100.0	34-3S	EI transition	58202	100.0
33-6S	peak I	50803	21.6	34-3S	peak I	58690	21.1
33-6S	IE transition	51385	37.6	34-3S	IE transition	58914	30.8
33-6S	peak E	51691	46.1	34-6S	EI transition	30191	0.0
33-6S	EI transition	53646	100.0	34-6S	peak I	30750	21.2
33-6S	peak I	54082	12.4	34-6S	IE transition	31205	38.5
33-6S	IE transition	54820	33.3	34-6S	peak E	31546	51.4
33-6S	peak E	55165	43.1	34-6S	EI transition	32828	100.0
33-6S	EI transition	57168	100.0	34-6S	peak I	33362	19.6
33-6S	peak I	57743	17.7	34-6S	IE transition	33918	40.1
33-6S	IE transition	58370	37.0	34-6S	peak E	34134	48.1
33-6S	peak E	59551	73.4	34-6S	EI transition	35545	100.0
33-6S	EI transition	60415	100.0	34-6S	peak I	35987	15.6
33-7S	EI transition	32321	0.0	34-6S	IE transition	36696	40.6
33-7S	peak I	32859	17.5	34-6S	peak E	37214	58.9
33-7S	IE transition	33353	33.6	34-6S	EI transition	38379	100.0
33-7S	peak E	34161	59.9	34-6S	peak I	38872	17.3
33-7S	EI transition	35395	100.0	34-6S	IE transition	39465	38.1
33-7S	peak I	35888	15.8	34-6S	peak E	40132	61.5
33-7S	IE transition	36535	36.5	34-6S	EI transition	41231	100.0
33-7S	peak E	36818	45.6	34-6S	peak I	41645	14.1
33-7S	EI transition	38518	100.0	34-6S	IE transition	42331	37.5
33-7S	peak I	39174	20.4	34-6S	peak E	43532	78.5
33-7S	IE transition	39685	36.3	34-6S	EI transition	44163	100.0
33-7S	peak E	39880	42.4	34-6S	peak I	44599	13.9
33-7S	EI transition	41732	100.0	34-6S	IE transition	45452	41.0
33-7S	peak I	42376	20.6	34-6S	peak E	46156	63.5
33-7S	IE transition	42828	35.0	34-6S	EI transition	47304	100.0
33-7S	peak E	43059	42.4	34-6S	peak I	47707	13.7
33-7S	EI transition	44864	100.0	34-6S	IE transition	48386	36.8
33-7S	peak I	45552	23.4	34-6S	peak E	48766	49.8
33-7S	IE transition	46189	45.0	34-6S	EI transition	50242	100.0
33-7S	peak E	46823	66.5	34-6S	peak I	50676	13.0
33-7S	EI transition	47807	100.0	34-6S	IE transition	51531	38.5
33-7S	peak I	48208	13.2	34-6S	peak E	52223	59.2
33-7S	IE transition	48836	34.0	34-6S	EI transition	53590	100.0
33-7S	peak E	49253	47.8	34-6S	peak I	54098	16.9
33-7S	EI transition	50834	100.0	34-6S	IE transition	54667	35.9
33-7S	peak I	51273	15.8	34-6S	peak E	55169	52.7
33-7S	IE transition	51937	39.6	34-6S	EI transition	56589	100.0
33-7S	peak E	52270	51.6	34-6S	peak I	57120	17.9
33-7S	EI transition	53618	100.0	34-6S	IE transition	57693	37.2
33-7S	peak I	54050	12.1	34-6S	peak E	58028	48.4
33-7S	IE transition	54684	29.8	34-6S	EI transition	59562	100.0
33-7S	peak E	54958	37.5	34-8S	EI transition	31502	0.0
33-7S	EI transition	57192	100.0	34-8S	peak I	32071	16.4
33-7S	peak I	57695	16.2	34-8S	IE transition	32761	36.2
33-7S	IE transition	58401	38.9	34-8S	peak E	33132	46.9
33-7S	peak E	58766	50.6	34-8S	EI transition	34975	100.0
33-8S	EI transition	31412	0.0	34-8S	peak I	35503	15.7
33-8S	peak I	32085	21.5	34-8S	IE transition	36136	34.6
33-8S	IE transition	32520	35.4	34-8S	peak E	36817	54.8
33-8S	peak E	32799	44.4	34-8S	EI transition	38334	100.0
33-8S	EI transition	34539	100.0	34-8S	peak I	38799	14.7
33-8S	peak I	35000	15.1	34-8S	IE transition	39486	36.3
33-8S	IE transition	35584	34.2	34-8S	peak E	40403	65.2

33-8S	peak E	35897	44.5
33-8S	EI transition	37592	100.0
33-8S	peak I	38095	14.5
33-8S	IE transition	38660	30.8
33-8S	peak E	38978	40.0
33-8S	EI transition	41058	100.0
33-8S	peak I	41439	11.4
33-8S	IE transition	42148	32.7
33-8S	peak E	42603	46.4
33-8S	EI transition	44390	100.0
33-8S	peak I	44876	16.3
33-8S	IE transition	45445	35.4
33-8S	peak E	45763	46.1
33-8S	EI transition	47370	100.0
33-8S	peak I	47722	11.4
33-8S	IE transition	48436	34.5
33-8S	peak E	48749	44.7
33-8S	EI transition	50457	100.0
33-8S	peak I	50851	13.1
33-8S	IE transition	51549	36.3
33-8S	peak E	52017	51.8
33-8S	EI transition	53469	100.0
33-8S	peak I	53921	14.6
33-8S	IE transition	54598	36.4
33-8S	peak E	54988	49.0
33-8S	EI transition	56567	100.0
33-8S	peak I	57148	18.1
33-8S	IE transition	57743	36.7
33-8S	peak E	58022	45.4
33-8S	EI transition	59774	100.0

34-8S	EI transition	41505	100.0
34-8S	peak I	42082	18.5
34-8S	IE transition	42747	39.8
34-8S	peak E	43767	72.4
34-8S	EI transition	44629	100.0
34-8S	peak I	45108	16.4
34-8S	IE transition	45706	36.8
34-8S	peak E	46158	52.3
34-8S	EI transition	47554	100.0
34-8S	peak I	48051	14.5
34-8S	IE transition	48661	32.4
34-8S	peak E	48861	38.2
34-8S	EI transition	50974	100.0
34-8S	peak I	51452	15.6
34-8S	IE transition	52118	37.3
34-8S	peak E	52545	51.2
34-8S	EI transition	54040	100.0
34-8S	peak I	54475	14.4
34-8S	IE transition	55226	39.4
34-8S	peak E	55772	57.5
34-8S	EI transition	57050	100.0
34-8S	peak I	57695	25.2
34-8S	IE transition	58134	42.3
34-8S	peak E	58471	55.5
34-8S	EI transition	59610	100.0

name, session	parameter	time (ms)	ratio (%)
35-1S	EI transition	31645	0.0
35-1S	peak I	32560	25.9
35-1S	IE transition	32928	36.3
35-1S	peak E	33389	49.4
35-1S	EI transition	35178	100.0
35-1S	peak I	35521	11.2
35-1S	IE transition	36327	37.7
35-1S	peak E	36559	45.3
35-1S	EI transition	38228	100.0
35-1S	peak I	38627	14.6
35-1S	IE transition	39269	38.2
35-1S	peak E	39556	48.7
35-1S	EI transition	40956	100.0
35-1S	peak I	41366	14.9
35-1S	IE transition	42014	38.6
35-1S	peak E	42315	49.5
35-1S	EI transition	43699	100.0
35-1S	peak I	44177	16.3
35-1S	IE transition	44895	40.7
35-1S	peak E	45184	50.6
35-1S	EI transition	46635	100.0
35-1S	peak I	47232	19.0
35-1S	IE transition	47705	34.1
35-1S	peak E	48027	44.3
35-1S	EI transition	49776	100.0
35-1S	peak I	50388	20.7
35-1S	IE transition	50832	35.7
35-1S	peak E	51138	46.0
35-1S	EI transition	52737	100.0
35-1S	peak I	53147	14.2
35-1S	IE transition	53763	35.6
35-1S	peak E	54048	45.5
35-1S	EI transition	55618	100.0
35-1S	peak I	56204	18.7
35-1S	IE transition	56815	38.3
35-1S	peak E	57119	48.0
35-1S	EI transition	58746	100.0
35-1S	peak I	59195	15.3
35-1S	IE transition	59830	37.0
35-3S	EI transition	31356	0.0
35-3S	peak I	31870	15.9
35-3S	IE transition	32521	36.0
35-3S	peak E	32804	44.7
35-3S	EI transition	34594	100.0
35-3S	peak I	35000	11.5
35-3S	IE transition	35716	31.8
35-3S	peak E	36000	39.9
35-3S	EI transition	38117	100.0
35-3S	peak I	38515	12.5
35-3S	IE transition	39202	34.1
35-3S	peak E	39433	41.3
35-3S	EI transition	41302	100.0
35-3S	peak I	41606	11.0
35-3S	IE transition	42392	39.4
35-3S	peak E	42739	51.9
35-3S	EI transition	44071	100.0
35-3S	peak I	44365	10.5

name, session	parameter	time (ms)	ratio (%)
36-1S	EI transition	31681	0.0
36-1S	peak I	32003	8.6
36-1S	IE transition	32773	29.1
36-1S	peak E	33092	37.6
36-1S	EI transition	35436	100.0
36-1S	peak I	35865	12.8
36-1S	IE transition	36469	30.8
36-1S	peak E	36819	41.2
36-1S	EI transition	38793	100.0
36-1S	peak I	39490	40.5
36-1S	IE transition	39934	66.3
36-1S	peak E	40318	88.7
36-1S	EI transition	40513	100.0
36-1S	peak I	40629	3.8
36-1S	IE transition	41073	18.2
36-1S	peak E	41382	28.3
36-1S	EI transition	43582	100.0
36-1S	peak I	43921	9.2
36-1S	IE transition	44589	27.4
36-1S	peak E	44960	37.5
36-1S	EI transition	47261	100.0
36-1S	peak I	47555	9.0
36-1S	IE transition	48124	26.4
36-1S	peak E	48641	42.2
36-1S	EI transition	50531	100.0
36-1S	peak I	50950	12.9
36-1S	IE transition	51508	30.1
36-1S	peak E	51747	37.5
36-1S	EI transition	53772	100.0
36-1S	peak I	54253	13.3
36-1S	IE transition	54850	29.9
36-1S	peak E	55132	37.7
36-1S	EI transition	57380	100.0
36-1S	peak I	57795	11.5
36-1S	IE transition	58436	29.2
36-1S	peak E	58853	40.7
36-1S	EI transition	61001	100.0
36-2S	EI transition	30676	0.0
36-2S	peak I	31144	11.9
36-2S	IE transition	31844	29.8
36-2S	peak E	32137	37.3
36-2S	EI transition	34595	100.0
36-2S	peak I	35156	14.6
36-2S	IE transition	35842	32.5
36-2S	peak E	36116	39.7
36-2S	EI transition	38427	100.0
36-2S	peak I	38849	10.7
36-2S	IE transition	39587	29.4
36-2S	peak E	39923	38.0
36-2S	EI transition	42368	100.0
36-2S	peak I	42796	10.4
36-2S	IE transition	43552	28.9
36-2S	peak E	43885	37.0
36-2S	EI transition	46467	100.0
36-2S	peak I	46742	9.8
36-2S	IE transition	47189	25.6
36-2S	peak E	47495	36.5

35-3S	IE transition	45009	33.7
35-3S	peak E	45316	44.7
35-3S	EI transition	46858	100.0
35-3S	peak I	47403	17.6
35-3S	IE transition	48069	39.1
35-3S	peak E	48319	47.2
35-3S	EI transition	49953	100.0
35-3S	peak I	50262	9.8
35-3S	IE transition	51067	35.3
35-3S	peak E	51407	46.0
35-3S	EI transition	53113	100.0
35-3S	peak I	53464	10.5
35-3S	IE transition	54172	31.5
35-3S	peak E	54527	42.1
35-3S	EI transition	56475	100.0
35-3S	peak I	56818	11.7
35-3S	IE transition	57628	39.5
35-3S	peak E	57899	48.7
35-3S	EI transition	59397	100.0
35-6S	EI transition	30368	0.0
35-6S	peak I	30721	12.7
35-6S	IE transition	31429	38.1
35-6S	peak E	31642	45.8
35-6S	EI transition	33151	100.0
35-6S	peak I	33495	13.7
35-6S	IE transition	34170	40.6
35-6S	peak E	34419	50.5
35-6S	EI transition	35660	100.0
35-6S	peak I	36089	15.5
35-6S	IE transition	36725	38.4
35-6S	peak E	37000	48.3
35-6S	EI transition	38434	100.0
35-6S	peak I	38821	14.4
35-6S	IE transition	39504	39.9
35-6S	peak E	39855	53.0
35-6S	EI transition	41116	100.0
35-6S	peak I	41577	13.9
35-6S	IE transition	42188	32.3
35-6S	peak E	42495	41.6
35-6S	EI transition	44434	100.0
35-6S	peak I	44832	13.4
35-6S	IE transition	45569	38.2
35-6S	peak E	45909	49.6
35-6S	EI transition	47407	100.0
35-6S	peak I	47721	12.0
35-6S	IE transition	48460	40.3
35-6S	peak E	48773	52.3
35-6S	EI transition	50018	100.0
35-6S	peak I	50392	11.8
35-6S	IE transition	51072	33.3
35-6S	peak E	51399	43.7
35-6S	EI transition	53181	100.0
35-6S	peak I	53632	14.9
35-6S	IE transition	54301	37.0
35-6S	peak E	54566	45.8
35-6S	EI transition	56207	100.0
35-6S	peak I	56541	11.4
35-6S	IE transition	57380	39.9
35-6S	peak E	57716	51.3

36-2S	EI transition	49286	100.0
36-2S	peak I	49637	13.4
36-2S	IE transition	50173	33.7
36-2S	peak E	50791	57.2
36-2S	EI transition	51915	100.0
36-2S	peak I	52178	8.8
36-2S	IE transition	52813	30.2
36-2S	peak E	53018	37.1
36-2S	EI transition	54892	100.0
36-2S	peak I	55407	18.1
36-2S	IE transition	55753	30.3
36-2S	peak E	56221	46.7
36-2S	EI transition	57735	100.0
36-2S	peak I	57920	8.3
36-2S	IE transition	58341	27.3
36-2S	peak E	58579	38.0
36-2S	EI transition	59954	100.0
36-3S	EI transition	30841	0.0
36-3S	peak I	31279	12.5
36-3S	IE transition	31829	28.2
36-3S	peak E	32096	35.8
36-3S	EI transition	34347	100.0
36-3S	peak I	34955	13.8
36-3S	IE transition	35490	26.0
36-3S	peak E	35773	32.4
36-3S	EI transition	38751	100.0
36-3S	peak I	39178	26.1
36-3S	IE transition	39813	65.0
36-3S	peak E	40089	81.8
36-3S	EI transition	40386	100.0
36-3S	peak I	40482	3.4
36-3S	IE transition	40774	13.5
36-3S	peak E	40959	20.0
36-3S	EI transition	43250	100.0
36-3S	peak I	43696	9.8
36-3S	IE transition	44299	23.1
36-3S	peak E	44603	29.8
36-3S	EI transition	47787	100.0
36-3S	peak I	48219	14.2
36-3S	IE transition	48766	32.2
36-3S	peak E	49106	43.3
36-3S	EI transition	50832	100.0
36-3S	peak I	51255	14.4
36-3S	IE transition	51681	28.9
36-3S	peak E	52106	43.4
36-3S	EI transition	53769	100.0
36-3S	peak I	54190	16.0
36-3S	IE transition	54533	29.1
36-3S	peak E	54698	35.4
36-3S	EI transition	56394	100.0
36-3S	peak I	56829	20.7
36-3S	IE transition	57491	52.1
36-3S	peak E	57864	69.8
36-3S	EI transition	58500	100.0
36-3S	peak I	58603	3.2
36-3S	IE transition	59138	19.8
36-3S	peak E	61305	87.1
36-3S	EI transition	61721	100.0
36-8S	EI transition	30836	0.0

35-6S	EI transition	59149	100.0
35-8S	EI transition	29041	0.0
35-8S	peak I	29443	13.4
35-8S	IE transition	30140	36.6
35-8S	peak E	30562	50.6
35-8S	EI transition	32043	100.0
35-8S	peak I	32391	10.5
35-8S	IE transition	33222	35.7
35-8S	peak E	33551	45.7
35-8S	EI transition	35344	100.0
35-8S	peak I	36076	25.5
35-8S	IE transition	36703	47.3
35-8S	peak E	36884	53.6
35-8S	EI transition	38219	100.0
35-8S	peak I	38686	15.6
35-8S	IE transition	39380	38.7
35-8S	peak E	39823	53.4
35-8S	EI transition	41222	100.0
35-8S	peak I	41569	12.2
35-8S	IE transition	42348	39.5
35-8S	peak E	42667	50.7
35-8S	EI transition	44070	100.0
35-8S	peak I	44491	15.1
35-8S	IE transition	45189	40.2
35-8S	peak E	45482	50.7
35-8S	EI transition	46856	100.0
35-8S	peak I	47167	10.0
35-8S	IE transition	47922	34.4
35-8S	peak E	48241	44.7
35-8S	EI transition	49954	100.0
35-8S	peak I	50352	13.6
35-8S	IE transition	51049	37.3
35-8S	peak E	51486	52.2
35-8S	EI transition	52888	100.0
35-8S	peak I	53527	22.4
35-8S	IE transition	54016	39.5
35-8S	peak E	54213	46.4
35-8S	EI transition	55745	100.0
35-8S	peak I	56056	10.0
35-8S	IE transition	56828	34.9
35-8S	peak E	57189	46.6
35-8S	EI transition	58845	100.0

36-8S	peak I	31293	13.4
36-8S	IE transition	31802	28.4
36-8S	peak E	32016	34.7
36-8S	EI transition	34236	100.0
36-8S	peak I	34705	13.6
36-8S	IE transition	35301	30.8
36-8S	peak E	35569	38.5
36-8S	EI transition	37695	100.0
36-8S	peak I	38069	11.1
36-8S	IE transition	38669	29.0
36-8S	peak E	38911	36.2
36-8S	EI transition	41055	100.0
36-8S	peak I	41488	12.1
36-8S	IE transition	42135	30.3
36-8S	peak E	42431	38.6
36-8S	EI transition	44621	100.0
36-8S	peak I	45043	12.5
36-8S	IE transition	45823	35.7
36-8S	peak E	46095	43.7
36-8S	EI transition	47992	100.0
36-8S	peak I	48354	10.9
36-8S	IE transition	49041	31.6
36-8S	peak E	49342	40.7
36-8S	EI transition	51312	100.0
36-8S	peak I	51637	10.4
36-8S	IE transition	52397	34.6
36-8S	peak E	52717	44.8
36-8S	EI transition	54451	100.0
36-8S	peak I	54856	10.7
36-8S	IE transition	55557	29.2
36-8S	peak E	55689	32.7
36-8S	EI transition	58241	100.0
36-8S	peak I	58679	13.4
36-8S	IE transition	59309	32.6
36-8S	peak E	59544	39.8
