# Supplementary material for: Respiratory modulation of cognitive performance during the retrieval process
Source: PLoS One. 2018 Sep 14;13(9):e0204021. doi: 10.1371/journal.pone.0204021 (PMC6138381; doi:10.1371/journal.pone.0204021)
Supplement: S4 Table — (PDF) [file pone.0204021.s006.pdf]

Table S4. Timing of peak I, IE transition, and peak E from EI transition during the test section in the Phased sessions

name, session	parameter	time (ms)	ratio (%)
18-3E	EI transition	31037	0.0
18-3E	peak I	31540	12.7
18-3E	IE transition	31972	23.6
18-3E	peak E	32308	32.1
18-3E	EI transition	35002	100.0
18-3E	peak I	35628	14.2
18-3E	IE transition	36216	27.4
18-3E	peak E	36684	38.0
18-3E	EI transition	39424	100.0
18-3E	peak I	40023	17.3
18-3E	IE transition	40377	27.6
18-3E	peak E	40606	34.2
18-3E	EI transition	42879	100.0
18-3E	peak I	43634	16.2
18-3E	IE transition	43972	23.4
18-3E	peak E	44555	35.9
18-3E	EI transition	47550	100.0
18-3E	peak I	48084	19.3
18-3E	IE transition	49153	58.0
18-3E	peak E	49358	65.4
18-3E	EI transition	50315	100.0
18-3E	peak I	50744	16.5
18-3E	IE transition	51161	32.5
18-3E	peak E	52047	66.5
18-3E	EI transition	52919	100.0
18-3E	peak I	53515	24.0
18-3E	IE transition	53764	34.0
18-3E	peak E	54324	56.5
18-3E	EI transition	55404	100.0
18-3E	peak I	55817	17.2
18-3E	IE transition	56135	30.4
18-3E	peak E	56642	51.5
18-3E	EI transition	57810	100.0
18-3E	peak I	58238	15.3
18-3E	IE transition	58719	32.4
18-3E	peak E	59113	46.5
18-3E	EI transition	60612	100.0
18-4I	EI transition	29674	0.0
18-4I	peak I	30246	21.4
18-4I	IE transition	30692	38.1
18-4I	peak E	30813	42.6
18-4I	EI transition	32348	100.0
18-4I	peak I	32945	14.8
18-4I	IE transition	33250	22.4
18-4I	peak E	33768	35.3
18-4I	EI transition	36376	100.0
18-4I	peak I	36877	11.1
18-4I	IE transition	37065	15.2
18-4I	peak E	39528	69.6
18-4I	EI transition	40902	100.0
18-4I	peak I	41440	31.4
18-4I	IE transition	42080	68.8
18-4I	peak E	42258	79.2
18-4I	EI transition	42614	100.0
18-4I	peak I	43021	8.5

name, session	parameter	time (ms)	ratio (%)
19-3I	EI transition	32978	0.0
19-3I	peak I	33239	6.8
19-3I	IE transition	34105	29.5
19-3I	peak E	34687	44.7
19-3I	EI transition	36802	100.0
19-3I	peak I	37091	9.9
19-3I	IE transition	37607	27.5
19-3I	peak E	38097	44.2
19-3I	EI transition	39730	100.0
19-3I	peak I	39967	10.5
19-3I	IE transition	40264	23.6
19-3I	peak E	41451	75.9
19-3I	EI transition	41997	100.0
19-3I	peak I	42262	13.3
19-3I	IE transition	42620	31.4
19-3I	peak E	43465	73.9
19-3I	EI transition	43983	100.0
19-3I	peak I	44250	10.2
19-3I	IE transition	44862	33.7
19-3I	peak E	45342	52.1
19-3I	EI transition	46591	100.0
19-3I	peak I	46851	10.2
19-3I	IE transition	47359	30.2
19-3I	peak E	47797	47.5
19-3I	EI transition	49132	100.0
19-3I	peak I	49392	7.8
19-3I	IE transition	50062	27.9
19-3I	peak E	50482	40.5
19-3I	EI transition	52468	100.0
19-3I	peak I	52780	12.9
19-3I	IE transition	53154	28.4
19-3I	peak E	53825	56.1
19-3I	EI transition	54886	100.0
19-3I	peak I	55136	9.8
19-3I	IE transition	55708	32.1
19-3I	peak E	56120	48.2
19-3I	EI transition	57448	100.0
19-3I	peak I	57710	9.3
19-3I	IE transition	58298	30.1
19-3I	peak E	58711	44.7
19-3I	EI transition	60273	100.0
19-3I	peak I	60546	9.1
19-3I	IE transition	61065	26.3
19-4E	EI transition	39288	0.0
19-4E	peak I	39544	11.3
19-4E	IE transition	39881	26.2
19-4E	peak E	41070	78.8
19-4E	EI transition	41548	100.0
19-4E	peak I	41841	13.3
19-4E	IE transition	42187	29.1
19-4E	peak E	43002	66.2
19-4E	EI transition	43746	100.0
19-4E	peak I	43999	8.1
19-4E	IE transition	44592	27.2
19-4E	peak E	44945	38.5

18-4I	IE transition	43402	16.4
18-4I	peak E	43772	24.1
18-4I	EI transition	47422	100.0
18-4I	peak I	48009	27.6
18-4I	IE transition	48289	40.8
18-4I	peak E	48603	55.6
18-4I	EI transition	49545	100.0
18-4I	peak I	49954	17.3
18-4I	IE transition	50199	27.7
18-4I	peak E	50678	47.9
18-4I	EI transition	51909	100.0
18-4I	peak I	52470	19.5
18-4I	IE transition	52888	34.1
18-4I	peak E	53331	49.5
18-4I	EI transition	54779	100.0
18-6I	EI transition	32520	0.0
18-6I	peak I	33167	19.0
18-6I	IE transition	33770	36.7
18-6I	peak E	34518	58.6
18-6I	EI transition	35930	100.0
18-6I	peak I	36557	20.7
18-6I	IE transition	37039	36.6
18-6I	peak E	37805	61.8
18-6I	EI transition	38962	100.0
18-6I	peak I	39521	19.7
18-6I	IE transition	39904	33.2
18-6I	peak E	40651	59.6
18-6I	EI transition	41798	100.0
18-6I	peak I	42443	21.8
18-6I	IE transition	42944	38.8
18-6I	peak E	43489	57.3
18-6I	EI transition	44750	100.0
18-6I	peak I	45338	20.7
18-6I	IE transition	45611	30.3
18-6I	peak E	45886	40.0
18-6I	EI transition	47588	100.0
18-6I	peak I	48175	21.7
18-6I	IE transition	48566	36.2
18-6I	peak E	49083	55.3
18-6I	EI transition	50290	100.0
18-6I	peak I	50761	16.6
18-6I	IE transition	51237	33.3
18-6I	peak E	51950	58.4
18-6I	EI transition	53133	100.0
18-6I	peak I	53877	26.0
18-6I	IE transition	54222	38.0
18-6I	peak E	54534	48.9
18-6I	EI transition	55996	100.0
18-6I	peak I	56464	8.2
18-6I	IE transition	57223	21.6
18-6I	peak E	57643	29.0
18-6I	EI transition	61670	100.0
18-7E	EI transition	31221	0.0
18-7E	peak I	31851	20.1
18-7E	IE transition	32089	27.7
18-7E	peak E	33395	69.4
18-7E	EI transition	34354	100.0
18-7E	peak I	35046	15.8
18-7E	IE transition	35618	28.9
19-4E	EI transition	46862	100.0
19-4E	peak I	47154	12.3
19-4E	IE transition	47551	29.0
19-4E	peak E	48020	48.8
19-4E	EI transition	49237	100.0
19-4E	peak I	49493	10.8
19-4E	IE transition	49963	30.7
19-4E	peak E	50442	50.9
19-4E	EI transition	51603	100.0
19-4E	peak I	51976	11.6
19-4E	IE transition	52597	31.0
19-4E	peak E	52968	42.6
19-4E	EI transition	54805	100.0
19-4E	peak I	55071	13.3
19-4E	IE transition	55384	29.0
19-4E	peak E	56097	64.8
19-4E	EI transition	56800	100.0
19-4E	peak I	57082	7.8
19-4E	IE transition	57898	30.2
19-4E	peak E	58308	41.5
19-4E	EI transition	60436	100.0
19-4E	peak I	60765	9.7
19-4E	IE transition	61436	29.3
19-4E	peak E	61794	39.8
19-4E	EI transition	63843	100.0
19-6E	EI transition	34387	0.0
19-6E	peak I	34689	8.9
19-6E	IE transition	35377	29.1
19-6E	peak E	36015	47.9
19-6E	EI transition	37787	100.0
19-6E	peak I	38067	8.5
19-6E	IE transition	38635	25.7
19-6E	peak E	40333	77.0
19-6E	EI transition	41094	100.0
19-6E	peak I	41330	6.1
19-6E	IE transition	42241	29.9
19-6E	peak E	42523	37.2
19-6E	EI transition	44934	100.0
19-6E	peak I	45260	8.9
19-6E	IE transition	45884	25.9
19-6E	peak E	46373	39.2
19-6E	EI transition	48601	100.0
19-6E	peak I	48881	7.4
19-6E	IE transition	49488	23.4
19-6E	peak E	51160	67.5
19-6E	EI transition	52390	100.0
19-6E	peak I	52642	5.9
19-6E	IE transition	53444	24.6
19-6E	peak E	53858	34.2
19-6E	EI transition	56679	100.0
19-6E	peak I	56992	8.4
19-6E	IE transition	57597	24.7
19-6E	peak E	58536	50.0
19-6E	EI transition	60396	100.0
19-6E	peak I	60690	8.1
19-6E	IE transition	61255	23.7
19-6E	peak E	62885	68.8
19-6E	EI transition	64012	100.0
19-6E	peak I	64238	9.8

18-7E	peak E	37301	67.4
18-7E	EI transition	38729	100.0
18-7E	peak I	39298	13.7
18-7E	IE transition	39690	23.2
18-7E	peak E	39933	29.1
18-7E	EI transition	42875	100.0
18-7E	peak I	43395	16.7
18-7E	IE transition	43658	25.1
18-7E	peak E	44417	49.4
18-7E	EI transition	45998	100.0
18-7E	peak I	46440	16.8
18-7E	IE transition	47094	41.7
18-7E	peak E	47612	61.3
18-7E	EI transition	48629	100.0
18-7E	peak I	49176	27.7
18-7E	IE transition	49406	39.3
18-7E	peak E	49747	56.6
18-7E	EI transition	50606	100.0
18-7E	peak I	51117	18.9
18-7E	IE transition	51426	30.3
18-7E	peak E	52552	71.9
18-7E	EI transition	53314	100.0
18-7E	peak I	53748	17.1
18-7E	IE transition	54222	35.7
18-7E	peak E	55481	85.2
18-7E	EI transition	55858	100.0
18-7E	peak I	56683	25.1
18-7E	IE transition	56884	31.2
18-7E	peak E	58106	68.3
18-7E	EI transition	59151	100.0

19-6E	IE transition	64567	24.1
19-6E	peak E	65771	76.3
19-6E	EI transition	66317	100.0
19-6E	peak I	66500	8.0
19-6E	IE transition	66920	26.5
19-7I	EI transition	50946	0.0
19-7I	peak I	51233	10.3
19-7I	IE transition	51621	24.3
19-7I	peak E	51885	33.9
19-7I	EI transition	53719	100.0
19-7I	peak I	54072	12.2
19-7I	IE transition	54455	25.5
19-7I	peak E	54790	37.1
19-7I	EI transition	56609	100.0
19-7I	peak I	56990	9.6
19-7I	IE transition	57601	24.9
19-7I	peak E	57957	33.8
19-7I	EI transition	60594	100.0
19-7I	peak I	60941	9.5
19-7I	IE transition	61501	24.9
19-7I	peak E	62152	42.7
19-7I	EI transition	64238	100.0
19-7I	peak I	64535	11.5
19-7I	IE transition	65046	31.2
19-7I	peak E	65358	43.2
19-7I	EI transition	66829	100.0
19-7I	peak I	67084	8.9
19-7I	IE transition	67605	27.0
19-7I	peak E	67862	36.0
19-7I	EI transition	69699	100.0
19-7I	peak I	70021	10.4
19-7I	IE transition	70474	25.0
19-7I	peak E	70861	37.4
19-7I	EI transition	72804	100.0
19-7I	peak I	73108	10.9
19-7I	IE transition	73562	27.1
19-7I	peak E	74958	76.9
19-7I	EI transition	75604	100.0
19-7I	peak I	75886	10.2
19-7I	IE transition	76355	27.2
19-7I	peak E	77862	81.7
19-7I	EI transition	78369	100.0

name, session	parameter	time (ms)	ratio (%)
20-2E	EI transition	31537	0.0
20-2E	peak I	31965	15.0
20-2E	IE transition	32613	37.7
20-2E	peak E	32922	48.6
20-2E	EI transition	34389	100.0
20-2E	peak I	34863	17.1
20-2E	IE transition	35442	38.1
20-2E	peak E	36106	62.1
20-2E	EI transition	37155	100.0
20-2E	peak I	37576	14.5
20-2E	IE transition	38188	35.7
20-2E	peak E	38887	59.8
20-2E	EI transition	40049	100.0
20-2E	peak I	40442	13.4
20-2E	IE transition	41147	37.5
20-2E	peak E	41706	56.6
20-2E	EI transition	42979	100.0
20-2E	peak I	43399	14.6
20-2E	IE transition	44089	38.6
20-2E	peak E	44537	54.2
20-2E	EI transition	45851	100.0
20-2E	peak I	46294	15.4
20-2E	IE transition	46915	36.9
20-2E	peak E	47359	52.3
20-2E	EI transition	48737	100.0
20-2E	peak I	49276	17.9
20-2E	IE transition	49835	36.5
20-2E	peak E	50139	46.6
20-2E	EI transition	51747	100.0
20-2E	peak I	52172	13.5
20-2E	IE transition	52852	35.1
20-2E	peak E	53201	46.2
20-2E	EI transition	54891	100.0
20-2E	peak I	55378	17.0
20-2E	IE transition	55959	37.2
20-2E	peak E	56276	48.3
20-2E	EI transition	57761	100.0
20-2E	peak I	58189	17.8
20-2E	IE transition	58407	26.9
20-2E	peak E	58499	30.7
20-2E	EI transition	60165	100.0
20-3I	EI transition	42609	0.0
20-3I	peak I	43150	18.3
20-3I	IE transition	43597	33.5
20-3I	peak E	43949	45.4
20-3I	EI transition	45560	100.0
20-3I	peak I	46037	14.8
20-3I	IE transition	46609	32.7
20-3I	peak E	47186	50.6
20-3I	EI transition	48772	100.0
20-3I	peak I	49265	15.2
20-3I	IE transition	49876	34.0
20-3I	peak E	50335	48.1
20-3I	EI transition	52022	100.0
20-3I	peak I	52521	16.4
20-3I	IE transition	53060	34.1
20-3I	peak E	53481	47.9

name, session	parameter	time (ms)	ratio (%)
21-2I	EI transition	26764	0.0
21-2I	peak I	27047	10.7
21-2I	IE transition	27684	34.7
21-2I	peak E	27841	40.6
21-2I	EI transition	29418	100.0
21-2I	peak I	29769	13.9
21-2I	IE transition	30414	39.4
21-2I	peak E	30681	50.0
21-2I	EI transition	31944	100.0
21-2I	peak I	32253	11.8
21-2I	IE transition	32939	38.0
21-2I	peak E	33251	49.9
21-2I	EI transition	34563	100.0
21-2I	peak I	34846	10.6
21-2I	IE transition	35641	40.4
21-2I	peak E	35798	46.3
21-2I	EI transition	37231	100.0
21-2I	peak I	37647	17.0
21-2I	IE transition	38255	41.7
21-2I	peak E	38443	49.4
21-2I	EI transition	39685	100.0
21-2I	peak I	40022	15.1
21-2I	IE transition	40630	42.4
21-2I	peak E	40821	50.9
21-2I	EI transition	41916	100.0
21-2I	peak I	42314	17.9
21-2I	IE transition	42867	42.8
21-2I	peak E	43035	50.4
21-2I	EI transition	44138	100.0
21-2I	peak I	44463	13.9
21-2I	IE transition	45108	41.6
21-2I	peak E	45224	46.6
21-2I	EI transition	46470	100.0
21-2I	peak I	46806	15.6
21-2I	IE transition	47430	44.3
21-2I	peak E	47667	55.3
21-2I	EI transition	48636	100.0
21-2I	peak I	48971	15.7
21-2I	IE transition	49553	42.9
21-2I	peak E	49856	57.1
21-2I	EI transition	50772	100.0
21-2I	peak I	51087	13.3
21-2I	IE transition	51700	39.1
21-2I	peak E	51883	46.8
21-3E	EI transition	24632	0.0
21-3E	peak I	24974	14.6
21-3E	IE transition	25669	44.1
21-3E	peak E	25970	56.9
21-3E	EI transition	26982	100.0
21-3E	peak I	27307	14.2
21-3E	IE transition	27944	42.1
21-3E	peak E	28194	53.0
21-3E	EI transition	29268	100.0
21-3E	peak I	29564	12.4
21-3E	IE transition	30224	40.1
21-3E	peak E	30528	52.8
21-3E	EI transition	31654	100.0

20-3I	EI transition	55065	100.0
20-3I	peak I	55558	17.3
20-3I	IE transition	56133	37.4
20-3I	peak E	56473	49.3
20-3I	EI transition	57919	100.0
20-3I	peak I	58395	16.4
20-3I	IE transition	58913	34.3
20-3I	peak E	59253	46.0
20-3I	EI transition	60817	100.0
20-3I	peak I	61292	15.4
20-3I	IE transition	61920	35.7
20-3I	peak E	62273	47.2
20-3I	EI transition	63905	100.0
20-3I	peak I	64442	16.2
20-3I	IE transition	64979	32.4
20-3I	peak E	65277	41.4
20-3I	EI transition	67219	100.0
20-3I	peak I	67734	16.5
20-3I	IE transition	68304	34.8
20-3I	peak E	68568	43.2
20-3I	EI transition	70340	100.0
20-3I	peak I	70949	18.2
20-3I	IE transition	71423	32.4
20-3I	peak E	71721	41.3
20-3I	EI transition	73681	100.0
20-5I	EI transition	34290	0.0
20-5I	peak I	34916	21.7
20-5I	IE transition	35322	35.8
20-5I	peak E	35556	43.9
20-5I	EI transition	37175	100.0
20-5I	peak I	37715	17.9
20-5I	IE transition	38265	36.2
20-5I	peak E	38819	54.5
20-5I	EI transition	40189	100.0
20-5I	peak I	40794	19.5
20-5I	IE transition	41306	36.0
20-5I	peak E	41679	48.0
20-5I	EI transition	43295	100.0
20-5I	peak I	44058	24.5
20-5I	IE transition	44484	38.2
20-5I	peak E	44834	49.5
20-5I	EI transition	46405	100.0
20-5I	peak I	46926	15.0
20-5I	IE transition	47451	30.0
20-5I	peak E	47906	43.1
20-5I	EI transition	49888	100.0
20-5I	peak I	50471	17.2
20-5I	IE transition	51100	35.9
20-5I	peak E	51283	41.3
20-5I	EI transition	53268	100.0
20-5I	peak I	53815	15.1
20-5I	IE transition	54408	31.5
20-5I	peak E	54921	45.7
20-5I	EI transition	56889	100.0
20-5I	peak I	57410	17.4
20-5I	IE transition	57903	33.9
20-5I	peak E	58251	45.5
20-5I	EI transition	59884	100.0
20-5I	peak I	60431	18.1
21-3E	peak I	31952	12.9
21-3E	IE transition	32652	43.1
21-3E	peak E	32942	55.6
21-3E	EI transition	33969	100.0
21-3E	peak I	34264	11.7
21-3E	IE transition	34984	40.1
21-3E	peak E	35410	57.0
21-3E	EI transition	36499	100.0
21-3E	peak I	36817	12.6
21-3E	IE transition	37515	40.4
21-3E	peak E	37798	51.7
21-3E	EI transition	39014	100.0
21-3E	peak I	39339	13.1
21-3E	IE transition	39999	39.8
21-3E	peak E	40230	49.2
21-3E	EI transition	41488	100.0
21-3E	peak I	41829	14.8
21-3E	IE transition	42520	44.9
21-3E	peak E	42905	61.7
21-3E	EI transition	43786	100.0
21-3E	peak I	44152	16.1
21-3E	IE transition	44754	42.6
21-3E	peak E	45103	58.0
21-3E	EI transition	46057	100.0
21-3E	peak I	46385	14.5
21-3E	IE transition	47006	42.1
21-3E	peak E	47280	54.3
21-5E	EI transition	26271	0.0
21-5E	peak I	26643	14.7
21-5E	IE transition	27310	41.1
21-5E	peak E	27559	51.0
21-5E	EI transition	28796	100.0
21-5E	peak I	29120	13.5
21-5E	IE transition	29765	40.3
21-5E	peak E	30036	51.6
21-5E	EI transition	31200	100.0
21-5E	peak I	31535	13.6
21-5E	IE transition	32245	42.4
21-5E	peak E	32476	51.7
21-5E	EI transition	33667	100.0
21-5E	peak I	33932	11.1
21-5E	IE transition	34655	41.3
21-5E	peak E	35402	72.5
21-5E	EI transition	36061	100.0
21-5E	peak I	36352	11.8
21-5E	IE transition	37045	40.0
21-5E	peak E	37284	49.7
21-5E	EI transition	38522	100.0
21-5E	peak I	38827	11.4
21-5E	IE transition	39517	37.3
21-5E	peak E	39677	43.3
21-5E	EI transition	41189	100.0
21-5E	peak I	41495	13.2
21-5E	IE transition	42164	42.1
21-5E	peak E	42535	58.2
21-5E	EI transition	43503	100.0
21-5E	peak I	43813	13.2
21-5E	IE transition	44430	39.3
21-5E	peak E	44830	56.3

20-5I	IE transition	60919	34.2
20-5I	peak E	61512	53.9
20-5I	EI transition	62907	100.0
20-6E	EI transition	45962	0.0
20-6E	peak I	47005	28.2
20-6E	IE transition	47343	37.3
20-6E	peak E	47780	49.2
20-6E	EI transition	49660	100.0
20-6E	peak I	50625	27.9
20-6E	IE transition	50858	34.7
20-6E	peak E	51128	42.5
20-6E	EI transition	53114	100.0
20-6E	peak I	53845	16.3
20-6E	IE transition	54243	25.1
20-6E	peak E	54806	37.6
20-6E	EI transition	57610	100.0
20-6E	peak I	58243	16.7
20-6E	IE transition	58782	31.0
20-6E	peak E	59431	48.1
20-6E	EI transition	61394	100.0
20-6E	peak I	62133	21.2
20-6E	IE transition	62546	33.1
20-6E	peak E	63185	51.4
20-6E	EI transition	64878	100.0
20-6E	peak I	65518	18.9
20-6E	IE transition	66016	33.7
20-6E	peak E	66807	57.1
20-6E	EI transition	68259	100.0
20-6E	peak I	68998	22.6
20-6E	IE transition	69511	38.2
20-6E	peak E	69935	51.2
20-6E	EI transition	71534	100.0
20-6E	peak I	72232	21.5
20-6E	IE transition	72621	33.4
20-6E	peak E	73282	53.8
20-6E	EI transition	74785	100.0
20-6E	peak I	75417	17.5
20-6E	IE transition	75949	32.3
20-6E	peak E	76515	48.0
20-6E	EI transition	78392	100.0

21-5E	EI transition	45859	100.0
21-5E	peak I	46174	13.0
21-5E	IE transition	46861	41.2
21-5E	peak E	47019	47.7
21-5E	EI transition	48290	100.0
21-5E	peak I	48589	13.4
21-5E	IE transition	49207	41.1
21-5E	peak E	49468	52.8
21-6I	EI transition	28622	0.0
21-6I	peak I	28971	14.4
21-6I	IE transition	29610	40.8
21-6I	peak E	29767	47.2
21-6I	EI transition	31046	100.0
21-6I	peak I	31376	13.7
21-6I	IE transition	32023	40.7
21-6I	peak E	32246	50.0
21-6I	EI transition	33447	100.0
21-6I	peak I	33780	14.8
21-6I	IE transition	34372	41.2
21-6I	peak E	34651	53.6
21-6I	EI transition	35694	100.0
21-6I	peak I	36014	13.6
21-6I	IE transition	36707	43.2
21-6I	peak E	37207	64.5
21-6I	EI transition	38039	100.0
21-6I	peak I	38334	11.9
21-6I	IE transition	39014	39.2
21-6I	peak E	39365	53.4
21-6I	EI transition	40524	100.0
21-6I	peak I	40826	12.8
21-6I	IE transition	41490	41.0
21-6I	peak E	41751	52.1
21-6I	EI transition	42881	100.0
21-6I	peak I	43236	15.3
21-6I	IE transition	43848	41.6
21-6I	peak E	44104	52.6
21-6I	EI transition	45207	100.0
21-6I	peak I	45576	15.7
21-6I	IE transition	46185	41.6
21-6I	peak E	46311	47.0
21-6I	EI transition	47557	100.0
21-6I	peak I	47882	14.2
21-6I	IE transition	48454	39.3
21-6I	peak E	48654	48.1
21-6I	EI transition	49840	100.0
21-6I	peak I	50120	12.8
21-6I	IE transition	50770	42.4
21-6I	peak E	51083	56.6

name, session	parameter	time (ms)	ratio (%)
22-2E	EI transition	35935	0.0
22-2E	peak I	36330	13.5
22-2E	IE transition	37008	36.6
22-2E	peak E	37261	45.2
22-2E	EI transition	38870	100.0
22-2E	peak I	39260	14.1
22-2E	IE transition	39837	34.9
22-2E	peak E	41173	83.0
22-2E	EI transition	41644	100.0
22-2E	peak I	42139	18.0
22-2E	IE transition	42627	35.8
22-2E	peak E	43916	82.8
22-2E	EI transition	44388	100.0
22-2E	peak I	44685	9.6
22-2E	IE transition	45428	33.6
22-2E	peak E	47159	89.7
22-2E	EI transition	47479	100.0
22-2E	peak I	47826	11.3
22-2E	IE transition	48528	34.3
22-2E	peak E	50149	87.3
22-2E	EI transition	50539	100.0
22-2E	peak I	50992	15.9
22-2E	IE transition	51494	33.6
22-2E	peak E	52956	84.9
22-2E	EI transition	53385	100.0
22-2E	peak I	53722	12.5
22-2E	IE transition	54422	38.5
22-2E	peak E	54664	47.4
22-2E	EI transition	56081	100.0
22-2E	peak I	56600	17.5
22-2E	IE transition	57107	34.5
22-2E	peak E	58722	88.9
22-2E	EI transition	59051	100.0
22-2E	peak I	59449	14.8
22-2E	IE transition	60009	35.5
22-2E	peak E	61523	91.7
22-2E	EI transition	61746	100.0
22-3I	EI transition	41047	0.0
22-3I	peak I	41278	9.8
22-3I	IE transition	41973	39.2
22-3I	peak E	42978	81.8
22-3I	EI transition	43409	100.0
22-3I	peak I	43727	12.8
22-3I	IE transition	44299	35.7
22-3I	peak E	45426	80.9
22-3I	EI transition	45902	100.0
22-3I	peak I	46199	12.0
22-3I	IE transition	46840	38.1
22-3I	peak E	47901	81.1
22-3I	EI transition	48366	100.0
22-3I	peak I	48671	12.3
22-3I	IE transition	49235	35.1
22-3I	peak E	50058	68.3
22-3I	EI transition	50843	100.0
22-3I	peak I	51229	13.6
22-3I	IE transition	51779	33.1
22-3I	peak E	53080	79.0

name, session	parameter	time (ms)	ratio (%)
23-3E	EI transition	35425	0.0
23-3E	peak I	36109	21.3
23-3E	IE transition	36419	31.0
23-3E	peak E	36708	40.0
23-3E	EI transition	38629	100.0
23-3E	peak I	39146	16.1
23-3E	IE transition	39547	28.6
23-3E	peak E	40008	42.9
23-3E	EI transition	41844	100.0
23-3E	peak I	42456	21.8
23-3E	IE transition	42884	37.0
23-3E	peak E	43389	55.0
23-3E	EI transition	44654	100.0
23-3E	peak I	45597	30.7
23-3E	IE transition	45775	36.4
23-3E	peak E	46125	47.8
23-3E	EI transition	47729	100.0
23-3E	peak I	48255	16.2
23-3E	IE transition	48854	34.6
23-3E	peak E	49224	46.0
23-3E	EI transition	50979	100.0
23-3E	peak I	51722	23.8
23-3E	IE transition	52110	36.2
23-3E	peak E	52423	46.2
23-3E	EI transition	54104	100.0
23-3E	peak I	54661	18.2
23-3E	IE transition	55207	36.0
23-3E	peak E	55355	40.9
23-3E	EI transition	57165	100.0
23-3E	peak I	57960	25.0
23-3E	IE transition	58210	32.9
23-3E	peak E	58393	38.6
23-3E	EI transition	60343	100.0
23-3E	peak I	60734	13.2
23-3E	IE transition	61407	36.1
23-3E	peak E	61856	51.3
23-3E	EI transition	63294	100.0
23-4I	EI transition	33147	0.0
23-4I	peak I	33777	21.6
23-4I	IE transition	34197	36.0
23-4I	peak E	34826	57.5
23-4I	EI transition	36067	100.0
23-4I	peak I	36889	26.4
23-4I	IE transition	37207	36.7
23-4I	peak E	37854	57.5
23-4I	EI transition	39175	100.0
23-4I	peak I	39754	18.1
23-4I	IE transition	40389	37.9
23-4I	peak E	40622	45.2
23-4I	EI transition	42374	100.0
23-4I	peak I	42830	16.9
23-4I	IE transition	43459	40.1
23-4I	peak E	43804	52.8
23-4I	EI transition	45080	100.0
23-4I	peak I	45928	25.7
23-4I	IE transition	46250	35.4
23-4I	peak E	47735	80.3

22-3I	EI transition	53674	100.0
22-3I	peak I	54031	13.1
22-3I	IE transition	54673	36.8
22-3I	peak E	56076	88.5
22-3I	EI transition	56389	100.0
22-3I	peak I	56722	11.2
22-3I	IE transition	57393	33.7
22-3I	peak E	59101	90.9
22-3I	EI transition	59372	100.0
22-3I	peak I	59824	16.7
22-3I	IE transition	60351	36.2
22-3I	peak E	61685	85.6
22-3I	EI transition	62074	100.0
22-3I	peak I	62407	12.0
22-3I	IE transition	62996	33.1
22-3I	peak E	64382	82.9
22-3I	EI transition	64859	100.0
22-3I	peak I	65236	15.7
22-3I	IE transition	65877	42.3
22-3I	peak E	66926	85.9
22-3I	EI transition	67266	100.0
22-3I	peak I	67463	9.2
22-3I	IE transition	68124	40.0
22-3I	peak E	68913	76.8
22-5I	EI transition	36728	0.0
22-5I	peak I	36991	10.1
22-5I	IE transition	37667	36.0
22-5I	peak E	38876	82.5
22-5I	EI transition	39333	100.0
22-5I	peak I	39655	13.6
22-5I	IE transition	40270	39.6
22-5I	peak E	41125	75.8
22-5I	EI transition	41697	100.0
22-5I	peak I	41945	10.1
22-5I	IE transition	42663	39.2
22-5I	peak E	43659	79.6
22-5I	EI transition	44163	100.0
22-5I	peak I	44459	10.8
22-5I	IE transition	45130	35.4
22-5I	peak E	46464	84.2
22-5I	EI transition	46896	100.0
22-5I	peak I	47244	14.2
22-5I	IE transition	47898	40.9
22-5I	peak E	48704	73.8
22-5I	EI transition	49345	100.0
22-5I	peak I	49638	11.8
22-5I	IE transition	50299	38.4
22-5I	peak E	51238	76.2
22-5I	EI transition	51828	100.0
22-5I	peak I	52164	14.6
22-5I	IE transition	52816	42.9
22-5I	peak E	53547	74.6
22-5I	EI transition	54133	100.0
22-5I	peak I	54530	20.6
22-5I	IE transition	54937	41.7
22-5I	peak E	55319	61.5
22-5I	EI transition	56061	100.0
22-5I	peak I	56361	12.9
22-5I	IE transition	56986	39.7
23-4I	EI transition	48385	100.0
23-4I	peak I	49195	27.8
23-4I	IE transition	49455	36.7
23-4I	peak E	50071	57.9
23-4I	EI transition	51297	100.0
23-4I	peak I	51748	16.6
23-4I	IE transition	52395	40.5
23-4I	peak E	53074	65.5
23-4I	EI transition	54008	100.0
23-4I	peak I	54419	13.5
23-4I	IE transition	55218	39.8
23-4I	peak E	55428	46.6
23-4I	EI transition	57052	100.0
23-4I	peak I	57495	14.6
23-4I	IE transition	58102	34.7
23-4I	peak E	58457	46.4
23-4I	EI transition	60082	100.0
23-4I	peak I	60608	18.7
23-4I	IE transition	61164	38.4
23-4I	peak E	61583	53.3
23-4I	EI transition	62900	100.0
23-7E	EI transition	39022	0.0
23-7E	peak I	39541	15.7
23-7E	IE transition	40035	30.5
23-7E	peak E	40255	37.2
23-7E	EI transition	42338	100.0
23-7E	peak I	43213	23.8
23-7E	IE transition	43422	29.4
23-7E	peak E	43820	40.2
23-7E	EI transition	46020	100.0
23-7E	peak I	46717	22.6
23-7E	IE transition	46973	30.9
23-7E	peak E	47235	39.4
23-7E	EI transition	49109	100.0
23-7E	peak I	49776	21.0
23-7E	IE transition	50115	31.7
23-7E	peak E	50343	38.9
23-7E	EI transition	52280	100.0
23-7E	peak I	52870	18.5
23-7E	IE transition	53300	31.9
23-7E	peak E	53504	38.3
23-7E	EI transition	55476	100.0
23-7E	peak I	55948	15.7
23-7E	IE transition	56478	33.4
23-7E	peak E	56793	43.9
23-7E	EI transition	58477	100.0
23-7E	peak I	59034	20.3
23-7E	IE transition	59447	35.3
23-7E	peak E	59667	43.3
23-7E	EI transition	61227	100.0
23-7E	peak I	62176	30.9
23-7E	IE transition	62357	36.7
23-7E	peak E	62564	43.5
23-7E	EI transition	64302	100.0
23-7E	peak I	65149	28.7
23-7E	IE transition	65488	40.2
23-7E	peak E	65789	50.3
23-7E	EI transition	67257	100.0

22-5I	peak E	57909	79.3
22-5I	EI transition	58392	100.0
22-5I	peak I	58735	13.4
22-5I	IE transition	59274	34.5
22-5I	peak E	60529	83.5
22-5I	EI transition	60951	100.0
22-6E	EI transition	36018	0.0
22-6E	peak I	36537	19.1
22-6E	IE transition	37015	36.6
22-6E	peak E	38391	87.1
22-6E	EI transition	38741	100.0
22-6E	peak I	39089	12.0
22-6E	IE transition	39667	31.9
22-6E	peak E	41277	87.4
22-6E	EI transition	41643	100.0
22-6E	peak I	42048	13.5
22-6E	IE transition	42670	34.2
22-6E	peak E	44209	85.4
22-6E	EI transition	44646	100.0
22-6E	peak I	45037	14.1
22-6E	IE transition	45643	35.8
22-6E	peak E	46812	77.9
22-6E	EI transition	47428	100.0
22-6E	peak I	47834	14.4
22-6E	IE transition	48444	36.1
22-6E	peak E	49733	81.9
22-6E	EI transition	50242	100.0
22-6E	peak I	50534	10.5
22-6E	IE transition	51245	36.1
22-6E	peak E	52687	88.1
22-6E	EI transition	53017	100.0
22-6E	peak I	53387	12.4
22-6E	IE transition	54008	33.2
22-6E	peak E	55685	89.4
22-6E	EI transition	56000	100.0
22-6E	peak I	56421	15.6
22-6E	IE transition	56963	35.7
22-6E	peak E	58242	83.1
22-6E	EI transition	58698	100.0
22-6E	peak I	59022	10.9
22-6E	IE transition	59673	32.9
22-6E	peak E	61048	79.3
22-6E	EI transition	61663	100.0

name, session	parameter	time (ms)	ratio (%)
24-3I	EI transition	56086	0.0
24-3I	peak I	56341	6.1
24-3I	IE transition	57321	29.7
24-3I	peak E	57608	36.6
24-3I	EI transition	60244	100.0
24-3I	peak I	60427	4.7
24-3I	IE transition	61495	32.3
24-3I	peak E	61840	41.3
24-3I	EI transition	64112	100.0
24-3I	peak I	64473	10.7
24-3I	IE transition	65315	35.5
24-3I	peak E	65733	47.9
24-3I	EI transition	67495	100.0
24-3I	peak I	67905	10.8
24-3I	IE transition	68781	33.8
24-3I	peak E	69299	47.4
24-3I	EI transition	71301	100.0
24-3I	peak I	72018	22.1
24-3I	IE transition	72566	39.1
24-3I	peak E	72815	46.7
24-3I	EI transition	74540	100.0
24-3I	peak I	74837	7.3
24-3I	IE transition	75710	28.6
24-3I	peak E	75969	34.9
24-3I	EI transition	78630	100.0
24-3I	peak I	79214	14.3
24-3I	IE transition	80054	34.9
24-3I	peak E	80268	40.2
24-3I	EI transition	82708	100.0
24-3I	peak I	82953	4.8
24-3I	IE transition	84016	25.5
24-3I	peak E	84289	30.8
24-3I	EI transition	87836	100.0
24-3I	peak I	88444	11.6
24-3I	IE transition	89120	24.6
24-3I	peak E	89747	36.6
24-3I	EI transition	93058	100.0
24-3I	peak I	94034	18.1
24-3I	IE transition	94367	24.2
24-3I	peak E	95385	43.1
24-3I	EI transition	98458	100.0
24-4E	EI transition	43206	0.0
24-4E	peak I	43607	11.2
24-4E	IE transition	44554	37.5
24-4E	peak E	44782	43.9
24-4E	EI transition	46796	100.0
24-4E	peak I	47217	12.1
24-4E	IE transition	48148	38.8
24-4E	peak E	48345	44.4
24-4E	EI transition	50281	100.0
24-4E	peak I	50646	10.5
24-4E	IE transition	51547	36.5
24-4E	peak E	51777	43.1
24-4E	EI transition	53754	100.0
24-4E	peak I	54016	6.8
24-4E	IE transition	54958	31.1
24-4E	peak E	55152	36.1

name, session	parameter	time (ms)	ratio (%)
26-2E	EI transition	32799	0.0
26-2E	peak I	33325	19.5
26-2E	IE transition	33931	42.1
26-2E	peak E	34248	53.8
26-2E	EI transition	35490	100.0
26-2E	peak I	35920	17.9
26-2E	IE transition	36507	42.2
26-2E	peak E	36814	55.0
26-2E	EI transition	37898	100.0
26-2E	peak I	38633	25.6
26-2E	IE transition	39046	40.0
26-2E	peak E	39399	52.4
26-2E	EI transition	40765	100.0
26-2E	peak I	41134	13.5
26-2E	IE transition	41744	35.7
26-2E	peak E	42076	47.8
26-2E	EI transition	43505	100.0
26-2E	peak I	43934	15.5
26-2E	IE transition	44640	40.9
26-2E	peak E	44844	48.3
26-2E	EI transition	46280	100.0
26-2E	peak I	46617	13.0
26-2E	IE transition	47340	40.8
26-2E	peak E	47751	56.6
26-2E	EI transition	48879	100.0
26-2E	peak I	49552	19.9
26-2E	IE transition	50159	37.8
26-2E	peak E	50596	50.6
26-2E	EI transition	52269	100.0
26-2E	peak I	52802	16.9
26-2E	IE transition	53542	40.4
26-2E	peak E	53892	51.4
26-2E	EI transition	55425	100.0
26-2E	peak I	55821	13.6
26-2E	IE transition	56441	35.0
26-2E	peak E	56633	41.6
26-2E	EI transition	58330	100.0
26-3I	EI transition	31567	0.0
26-3I	peak I	32186	22.2
26-3I	IE transition	32625	38.0
26-3I	peak E	32922	48.6
26-3I	EI transition	34354	100.0
26-3I	peak I	34853	17.4
26-3I	IE transition	35485	39.5
26-3I	peak E	35895	53.9
26-3I	EI transition	37215	100.0
26-3I	peak I	37623	14.9
26-3I	IE transition	38235	37.3
26-3I	peak E	38567	49.4
26-3I	EI transition	39950	100.0
26-3I	peak I	40433	14.3
26-3I	IE transition	41102	34.2
26-3I	peak E	42874	86.7
26-3I	EI transition	43322	100.0
26-3I	peak I	43758	15.7
26-3I	IE transition	44404	39.0
26-3I	peak E	44608	46.3

24-4E	EI transition	57624	100.0
24-4E	peak I	58834	22.7
24-4E	IE transition	59604	37.1
24-4E	peak E	59844	41.6
24-4E	EI transition	62956	100.0
24-4E	peak I	63342	8.6
24-4E	IE transition	64132	26.3
24-4E	peak E	64443	33.3
24-4E	EI transition	67425	100.0
24-4E	peak I	68203	21.4
24-4E	IE transition	68895	40.4
24-4E	peak E	69167	47.9
24-4E	EI transition	71065	100.0
24-4E	peak I	71689	18.0
24-4E	IE transition	72406	38.7
24-4E	peak E	72691	46.9
24-4E	EI transition	74532	100.0
24-4E	peak I	74889	9.0
24-4E	IE transition	75760	30.8
24-4E	peak E	75985	36.5
24-4E	EI transition	78518	100.0
24-6E	EI transition	48516	0.0
24-6E	peak I	48887	8.9
24-6E	IE transition	49822	31.4
24-6E	peak E	50124	38.7
24-6E	EI transition	52673	100.0
24-6E	peak I	53144	12.9
24-6E	IE transition	54003	36.5
24-6E	peak E	54970	63.0
24-6E	EI transition	56319	100.0
24-6E	peak I	56818	14.4
24-6E	IE transition	57840	43.9
24-6E	peak E	58159	53.1
24-6E	EI transition	59787	100.0
24-6E	peak I	60226	13.9
24-6E	IE transition	61047	40.0
24-6E	peak E	61828	64.9
24-6E	EI transition	62934	100.0
24-6E	peak I	63283	7.3
24-6E	IE transition	64152	25.6
24-6E	peak E	64706	37.3
24-6E	EI transition	67685	100.0
24-6E	peak I	68069	9.8
24-6E	IE transition	68973	32.8
24-6E	peak E	70205	64.1
24-6E	EI transition	71617	100.0
24-6E	peak I	72170	12.7
24-6E	IE transition	72972	31.1
24-6E	peak E	75560	90.4
24-6E	EI transition	75978	100.0
24-6E	peak I	76261	3.8
24-6E	IE transition	77132	15.5
24-6E	peak E	78259	30.7
24-6E	EI transition	83415	100.0
24-6E	peak I	84298	17.4
24-6E	IE transition	84843	28.1
24-6E	peak E	85185	34.8
24-6E	EI transition	88508	100.0
24-7I	EI transition	61266	0.0

26-3I	EI transition	46097	100.0
26-3I	peak I	46553	18.2
26-3I	IE transition	47064	38.6
26-3I	peak E	47424	53.0
26-3I	EI transition	48601	100.0
26-3I	peak I	49061	17.8
26-3I	IE transition	49630	39.8
26-3I	peak E	49816	47.0
26-3I	EI transition	51186	100.0
26-3I	peak I	51606	14.6
26-3I	IE transition	52197	35.0
26-3I	peak E	52398	42.0
26-3I	EI transition	54071	100.0
26-3I	peak I	54568	16.8
26-3I	IE transition	55148	36.3
26-3I	peak E	55417	45.4
26-3I	EI transition	57038	100.0
26-3I	peak I	57555	19.7
26-3I	IE transition	58109	40.9
26-3I	peak E	58470	54.7
26-3I	EI transition	59657	100.0
26-5I	EI transition	29928	0.0
26-5I	peak I	30400	16.2
26-5I	IE transition	31074	39.3
26-5I	peak E	31751	62.5
26-5I	EI transition	32843	100.0
26-5I	peak I	33281	15.6
26-5I	IE transition	33956	39.6
26-5I	peak E	34244	49.9
26-5I	EI transition	35652	100.0
26-5I	peak I	36105	15.9
26-5I	IE transition	36671	35.7
26-5I	peak E	37545	66.3
26-5I	EI transition	38508	100.0
26-5I	peak I	38911	15.0
26-5I	IE transition	39624	41.6
26-5I	peak E	40180	62.4
26-5I	EI transition	41188	100.0
26-5I	peak I	41598	15.6
26-5I	IE transition	42203	38.7
26-5I	peak E	42705	57.9
26-5I	EI transition	43809	100.0
26-5I	peak I	44275	17.1
26-5I	IE transition	44900	40.0
26-5I	peak E	45134	48.6
26-5I	EI transition	46537	100.0
26-5I	peak I	47028	16.8
26-5I	IE transition	47729	40.7
26-5I	peak E	47929	47.5
26-5I	EI transition	49468	100.0
26-5I	peak I	49897	15.9
26-5I	IE transition	50534	39.4
26-5I	peak E	50769	48.1
26-5I	EI transition	52171	100.0
26-5I	peak I	52639	17.1
26-5I	IE transition	53247	39.3
26-5I	peak E	53810	59.9
26-5I	EI transition	54906	100.0
26-5I	peak I	55369	15.5

24-7I	peak I	61746	13.6
24-7I	IE transition	62614	38.2
24-7I	peak E	62989	48.8
24-7I	EI transition	64794	100.0
24-7I	peak I	65278	12.8
24-7I	IE transition	65922	29.9
24-7I	peak E	66364	41.7
24-7I	EI transition	68563	100.0
24-7I	peak I	68835	6.6
24-7I	IE transition	69809	30.3
24-7I	peak E	70125	38.0
24-7I	EI transition	72677	100.0
24-7I	peak I	73411	17.2
24-7I	IE transition	73977	30.5
24-7I	peak E	74378	39.9
24-7I	EI transition	76943	100.0
24-7I	peak I	77541	14.7
24-7I	IE transition	78264	32.5
24-7I	peak E	78462	37.4
24-7I	EI transition	81004	100.0
24-7I	peak I	81490	14.1
24-7I	IE transition	82271	36.8
24-7I	peak E	82624	47.1
24-7I	EI transition	84443	100.0
24-7I	peak I	84896	10.8
24-7I	IE transition	85647	28.7
24-7I	peak E	85884	34.4
24-7I	EI transition	88638	100.0
24-7I	peak I	89124	13.4
24-7I	IE transition	89835	32.9
24-7I	peak E	90070	39.4
24-7I	EI transition	92272	100.0
24-7I	peak I	92727	12.2
24-7I	IE transition	93531	33.7
24-7I	peak E	93952	45.0
24-7I	EI transition	96005	100.0
24-7I	peak I	96411	11.9
24-7I	IE transition	97291	37.5
24-7I	peak E	97665	48.5
24-7I	EI transition	99431	100.0

26-5I	IE transition	55977	35.9
26-5I	peak E	56277	46.0
26-5I	EI transition	57887	100.0
26-6E	EI transition	35772	0.0
26-6E	peak I	36150	13.3
26-6E	IE transition	36832	37.4
26-6E	peak E	37085	46.3
26-6E	EI transition	38608	100.0
26-6E	peak I	39094	14.2
26-6E	IE transition	39750	33.4
26-6E	peak E	39932	38.7
26-6E	EI transition	42025	100.0
26-6E	peak I	42382	11.9
26-6E	IE transition	43091	35.6
26-6E	peak E	43565	51.4
26-6E	EI transition	45019	100.0
26-6E	peak I	45411	13.4
26-6E	IE transition	46030	34.4
26-6E	peak E	46226	41.1
26-6E	EI transition	47955	100.0
26-6E	peak I	48369	12.4
26-6E	IE transition	49083	33.9
26-6E	peak E	49249	38.8
26-6E	EI transition	51286	100.0
26-6E	peak I	51655	10.3
26-6E	IE transition	52370	30.3
26-6E	peak E	52627	37.5
26-6E	EI transition	54861	100.0
26-6E	peak I	55363	16.6
26-6E	IE transition	55944	35.7
26-6E	peak E	56203	44.3
26-6E	EI transition	57892	100.0
26-6E	peak I	58155	6.4
26-6E	IE transition	58813	22.3
26-6E	peak E	58950	25.6
26-6E	EI transition	62025	100.0
26-6E	peak I	62447	11.8
26-6E	IE transition	63175	32.0
26-6E	peak E	63466	40.1
26-6E	EI transition	65615	100.0

name, session	parameter	time (ms)	ratio (%)
27-2I	EI transition	34142	0.0
27-2I	peak I	34614	16.5
27-2I	IE transition	35235	38.3
27-2I	peak E	35657	53.1
27-2I	EI transition	36996	100.0
27-2I	peak I	37359	13.0
27-2I	IE transition	38026	36.8
27-2I	peak E	38368	49.0
27-2I	EI transition	39796	100.0
27-2I	peak I	40173	12.8
27-2I	IE transition	40848	35.6
27-2I	peak E	41248	49.2
27-2I	EI transition	42747	100.0
27-2I	peak I	43136	13.3
27-2I	IE transition	43805	36.3
27-2I	peak E	44190	49.5
27-2I	EI transition	45661	100.0
27-2I	peak I	46098	14.8
27-2I	IE transition	46766	37.4
27-2I	peak E	47139	50.0
27-2I	EI transition	48619	100.0
27-2I	peak I	49013	13.9
27-2I	IE transition	49697	38.1
27-2I	peak E	49999	48.8
27-2I	EI transition	51445	100.0
27-2I	peak I	51871	14.8
27-2I	IE transition	52564	38.9
27-2I	peak E	53012	54.4
27-2I	EI transition	54324	100.0
27-2I	peak I	54667	12.5
27-2I	IE transition	55388	38.7
27-2I	peak E	55593	46.1
27-2I	EI transition	57076	100.0
27-2I	peak I	57396	11.9
27-2I	IE transition	58024	35.3
27-2I	peak E	58582	56.1
27-2I	EI transition	59761	100.0
27-2I	peak I	60121	12.6
27-2I	IE transition	60771	35.5
27-2I	peak E	61204	50.7
27-2I	EI transition	62607	100.0
27-4E	EI transition	33614	0.0
27-4E	peak I	33908	10.3
27-4E	IE transition	34665	36.8
27-4E	peak E	35166	54.3
27-4E	EI transition	36471	100.0
27-4E	peak I	36844	11.7
27-4E	IE transition	37599	35.3
27-4E	peak E	38003	48.0
27-4E	EI transition	39663	100.0
27-4E	peak I	40025	12.5
27-4E	IE transition	40697	35.7
27-4E	peak E	41075	48.8
27-4E	EI transition	42559	100.0
27-4E	peak I	42948	12.6
27-4E	IE transition	43669	36.0
27-4E	peak E	44026	47.6

name, session	parameter	time (ms)	ratio (%)
28-2E	EI transition	31289	0.0
28-2E	peak I	31706	14.3
28-2E	IE transition	32391	37.8
28-2E	peak E	32577	44.1
28-2E	EI transition	34209	100.0
28-2E	peak I	34573	14.3
28-2E	IE transition	35142	36.8
28-2E	peak E	35925	67.6
28-2E	EI transition	36747	100.0
28-2E	peak I	37137	13.9
28-2E	IE transition	37852	39.5
28-2E	peak E	38352	57.3
28-2E	EI transition	39548	100.0
28-2E	peak I	40033	17.4
28-2E	IE transition	40609	38.1
28-2E	peak E	41307	63.2
28-2E	EI transition	42333	100.0
28-2E	peak I	42696	12.9
28-2E	IE transition	43428	38.9
28-2E	peak E	43619	45.7
28-2E	EI transition	45147	100.0
28-2E	peak I	45495	13.0
28-2E	IE transition	46156	37.7
28-2E	peak E	46938	67.0
28-2E	EI transition	47820	100.0
28-2E	peak I	48168	13.6
28-2E	IE transition	48877	41.2
28-2E	peak E	49722	74.1
28-2E	EI transition	50386	100.0
28-2E	peak I	50844	16.3
28-2E	IE transition	51402	36.2
28-2E	peak E	51625	44.1
28-2E	EI transition	53194	100.0
28-2E	peak I	53946	26.2
28-2E	IE transition	54226	35.9
28-2E	peak E	54547	47.1
28-2E	EI transition	56070	100.0
28-2E	peak I	56705	20.9
28-2E	IE transition	57137	35.1
28-4I	EI transition	33562	0.0
28-4I	peak I	34206	21.7
28-4I	IE transition	34565	33.8
28-4I	peak E	34917	45.6
28-4I	EI transition	36532	100.0
28-4I	peak I	37001	16.1
28-4I	IE transition	37500	33.1
28-4I	peak E	38067	52.6
28-4I	EI transition	39453	100.0
28-4I	peak I	39942	15.3
28-4I	IE transition	40495	32.5
28-4I	peak E	40808	42.3
28-4I	EI transition	42656	100.0
28-4I	peak I	43105	15.7
28-4I	IE transition	43612	33.4
28-4I	peak E	44048	48.7
28-4I	EI transition	45517	100.0
28-4I	peak I	45907	13.6

27-4E	EI transition	45641	100.0
27-4E	peak I	45994	11.5
27-4E	IE transition	46826	38.6
27-4E	peak E	47200	50.8
27-4E	EI transition	48710	100.0
27-4E	peak I	49101	12.4
27-4E	IE transition	49881	37.2
27-4E	peak E	50329	51.4
27-4E	EI transition	51859	100.0
27-4E	peak I	52245	13.1
27-4E	IE transition	52934	36.5
27-4E	peak E	53313	49.3
27-4E	EI transition	54807	100.0
27-4E	peak I	55232	14.1
27-4E	IE transition	55860	34.8
27-4E	peak E	56264	48.2
27-4E	EI transition	57830	100.0
27-4E	peak I	58224	12.6
27-4E	IE transition	58940	35.5
27-4E	peak E	59323	47.8
27-4E	EI transition	60956	100.0
27-6E	EI transition	31984	0.0
27-6E	peak I	32310	10.7
27-6E	IE transition	32988	33.1
27-6E	peak E	33395	46.5
27-6E	EI transition	35020	100.0
27-6E	peak I	35394	11.3
27-6E	IE transition	36019	30.3
27-6E	peak E	36417	42.4
27-6E	EI transition	38316	100.0
27-6E	peak I	38729	11.4
27-6E	IE transition	39394	29.8
27-6E	peak E	39857	42.5
27-6E	EI transition	41938	100.0
27-6E	peak I	42983	33.2
27-6E	IE transition	43284	42.7
27-6E	peak E	43650	54.4
27-6E	EI transition	45087	100.0
27-6E	peak I	45554	16.2
27-6E	IE transition	46167	37.4
27-6E	peak E	46468	47.8
27-6E	EI transition	47976	100.0
27-6E	peak I	48430	13.7
27-6E	IE transition	49113	34.2
27-6E	peak E	49480	45.3
27-6E	EI transition	51299	100.0
27-6E	peak I	51887	19.7
27-6E	IE transition	52411	37.3
27-6E	peak E	54057	92.5
27-6E	EI transition	54280	100.0
27-6E	peak I	54723	12.7
27-6E	IE transition	55566	37.0
27-6E	peak E	55991	49.2
27-6E	EI transition	57760	100.0
27-6E	peak I	58409	19.5
27-6E	IE transition	58872	33.4
27-6E	peak E	59211	43.6
27-6E	EI transition	61089	100.0
27-6E	peak I	61432	12.0

28-4I	IE transition	46500	34.2
28-4I	peak E	46819	45.3
28-4I	EI transition	48390	100.0
28-4I	peak I	48927	18.8
28-4I	IE transition	49345	33.5
28-4I	peak E	50055	58.4
28-4I	EI transition	51242	100.0
28-4I	peak I	51704	16.9
28-4I	IE transition	52179	34.3
28-4I	peak E	52907	60.9
28-4I	EI transition	53976	100.0
28-4I	peak I	54433	16.0
28-4I	IE transition	54939	33.7
28-4I	peak E	55356	48.3
28-4I	EI transition	56836	100.0
28-4I	peak I	57491	21.0
28-4I	IE transition	57857	32.8
28-4I	peak E	58584	56.2
28-4I	EI transition	59948	100.0
28-4I	peak I	60377	14.6
28-4I	IE transition	60892	32.0
28-4I	peak E	61978	68.9
28-4I	EI transition	62895	100.0
28-6I	EI transition	34287	0.0
28-6I	peak I	35063	24.2
28-6I	IE transition	35468	36.8
28-6I	peak E	35812	47.5
28-6I	EI transition	37498	100.0
28-6I	peak I	37960	16.1
28-6I	IE transition	38556	36.8
28-6I	peak E	39085	55.2
28-6I	EI transition	40375	100.0
28-6I	peak I	40814	13.9
28-6I	IE transition	41354	31.1
28-6I	peak E	41550	37.3
28-6I	EI transition	43524	100.0
28-6I	peak I	44062	18.3
28-6I	IE transition	44531	34.3
28-6I	peak E	44684	39.5
28-6I	EI transition	46464	100.0
28-6I	peak I	46876	12.4
28-6I	IE transition	47463	30.1
28-6I	peak E	47803	40.3
28-6I	EI transition	49786	100.0
28-6I	peak I	50285	13.6
28-6I	IE transition	50916	30.9
28-6I	peak E	51096	35.8
28-6I	EI transition	53442	100.0
28-6I	peak I	54067	22.1
28-6I	IE transition	54509	37.6
28-6I	peak E	54758	46.4
28-6I	EI transition	56280	100.0
28-6I	peak I	56832	17.8
28-6I	IE transition	57339	34.1
28-6I	peak E	57659	44.4
28-6I	EI transition	59389	100.0
28-6I	peak I	59864	16.0
28-6I	IE transition	60438	35.3
28-6I	peak E	60993	53.9

27-6E	IE transition	62229	39.9
27-6E	peak E	62700	56.4
27-6E	EI transition	63945	100.0
27-7I	EI transition	38206	0.0
27-7I	peak I	38755	16.8
27-7I	IE transition	39379	35.9
27-7I	peak E	39749	47.2
27-7I	EI transition	41474	100.0
27-7I	peak I	41737	8.1
27-7I	IE transition	42716	38.3
27-7I	peak E	43223	54.0
27-7I	EI transition	44715	100.0
27-7I	peak I	45219	13.9
27-7I	IE transition	45964	34.5
27-7I	peak E	46342	45.0
27-7I	EI transition	48333	100.0
27-7I	peak I	48834	15.8
27-7I	IE transition	49492	36.6
27-7I	peak E	49908	49.7
27-7I	EI transition	51501	100.0
27-7I	peak I	51950	12.2
27-7I	IE transition	52738	33.5
27-7I	peak E	53246	47.3
27-7I	EI transition	55189	100.0
27-7I	peak I	55825	17.4
27-7I	IE transition	56465	34.8
27-7I	peak E	56946	48.0
27-7I	EI transition	58852	100.0
27-7I	peak I	59240	11.3
27-7I	IE transition	60049	34.8
27-7I	peak E	60527	48.7
27-7I	EI transition	62290	100.0
27-7I	peak I	62729	14.5
27-7I	IE transition	63396	36.6
27-7I	peak E	63753	48.4
27-7I	EI transition	65310	100.0
27-7I	peak I	65926	18.2
27-7I	IE transition	66552	36.7
27-7I	peak E	66958	48.7
27-7I	EI transition	68691	100.0

28-6I	EI transition	62363	100.0
28-7E	EI transition	36186	0.0
28-7E	peak I	36768	18.1
28-7E	IE transition	37316	35.1
28-7E	peak E	37915	53.7
28-7E	EI transition	39408	100.0
28-7E	peak I	39952	18.6
28-7E	IE transition	40483	36.8
28-7E	peak E	41013	55.0
28-7E	EI transition	42326	100.0
28-7E	peak I	42718	13.1
28-7E	IE transition	43365	34.6
28-7E	peak E	43746	47.3
28-7E	EI transition	45329	100.0
28-7E	peak I	45889	16.7
28-7E	IE transition	46487	34.5
28-7E	peak E	47130	53.6
28-7E	EI transition	48686	100.0
28-7E	peak I	49167	15.1
28-7E	IE transition	49830	35.8
28-7E	peak E	50587	59.5
28-7E	EI transition	51880	100.0
28-7E	peak I	52391	15.9
28-7E	IE transition	52963	33.8
28-7E	peak E	53609	53.9
28-7E	EI transition	55084	100.0
28-7E	peak I	55762	21.0
28-7E	IE transition	56177	33.8
28-7E	peak E	57476	74.1
28-7E	EI transition	58313	100.0
28-7E	peak I	58650	10.9
28-7E	IE transition	59376	34.2
28-7E	peak E	60215	61.3
28-7E	EI transition	61418	100.0
28-7E	peak I	61782	11.2
28-7E	IE transition	62498	33.3
28-7E	peak E	63030	49.7
28-7E	EI transition	64661	100.0

name, session	parameter	time (ms)	ratio (%)
29-2I	EI transition	34338	0.0
29-2I	peak I	34976	22.1
29-2I	IE transition	35601	43.8
29-2I	peak E	36598	78.4
29-2I	EI transition	37221	100.0
29-2I	peak I	37826	21.2
29-2I	IE transition	38483	44.3
29-2I	peak E	39297	72.9
29-2I	EI transition	40070	100.0
29-2I	peak I	40519	15.4
29-2I	IE transition	41294	42.1
29-2I	peak E	42156	71.7
29-2I	EI transition	42978	100.0
29-2I	peak I	43164	5.1
29-2I	IE transition	44597	44.1
29-2I	peak E	44863	51.3
29-2I	EI transition	46653	100.0
29-2I	peak I	47459	24.9
29-2I	IE transition	47914	39.0
29-2I	peak E	48586	59.8
29-2I	EI transition	49887	100.0
29-2I	peak I	50695	24.5
29-2I	IE transition	51198	39.8
29-2I	peak E	51888	60.7
29-2I	EI transition	53182	100.0
29-2I	peak I	53533	11.2
29-2I	IE transition	54349	37.3
29-2I	peak E	55548	75.7
29-2I	EI transition	56308	100.0
29-2I	peak I	56855	14.8
29-2I	IE transition	57684	37.1
29-2I	peak E	59174	77.4
29-2I	EI transition	60013	100.0
29-2I	peak I	60775	18.5
29-2I	IE transition	61469	35.4
29-2I	peak E	62516	60.9
29-2I	EI transition	64125	100.0
29-3E	EI transition	35556	0.0
29-3E	peak I	35957	9.6
29-3E	IE transition	36882	31.9
29-3E	peak E	37577	48.5
29-3E	EI transition	39719	100.0
29-3E	peak I	40483	22.2
29-3E	IE transition	40881	33.8
29-3E	peak E	41077	39.5
29-3E	EI transition	43157	100.0
29-3E	peak I	44018	23.6
29-3E	IE transition	44474	36.1
29-3E	peak E	45234	56.9
29-3E	EI transition	46810	100.0
29-3E	peak I	47336	17.0
29-3E	IE transition	48017	39.0
29-3E	peak E	48376	50.6
29-3E	EI transition	49903	100.0
29-3E	peak I	50871	25.6
29-3E	IE transition	51200	34.3
29-3E	peak E	51790	50.0

name, session	parameter	time (ms)	ratio (%)
30-3I	EI transition	53026	0.0
30-3I	peak I	53266	8.4
30-3I	IE transition	53786	26.5
30-3I	peak E	54195	40.8
30-3I	EI transition	55896	100.0
30-3I	peak I	56376	11.5
30-3I	IE transition	57375	35.6
30-3I	peak E	57690	43.2
30-3I	EI transition	60052	100.0
30-3I	peak I	60333	6.9
30-3I	IE transition	61356	32.2
30-3I	peak E	62197	53.0
30-3I	EI transition	64102	100.0
30-3I	peak I	64384	5.9
30-3I	IE transition	65376	26.5
30-3I	peak E	65947	38.4
30-3I	EI transition	68904	100.0
30-3I	peak I	69368	11.0
30-3I	IE transition	70359	34.4
30-3I	peak E	70778	44.4
30-3I	EI transition	73128	100.0
30-3I	peak I	73414	6.1
30-3I	IE transition	74397	27.1
30-3I	peak E	74768	35.0
30-3I	EI transition	77818	100.0
30-3I	peak I	78151	7.3
30-3I	IE transition	79153	29.4
30-3I	peak E	79533	37.8
30-3I	EI transition	82352	100.0
30-3I	peak I	82799	9.7
30-3I	IE transition	83662	28.5
30-3I	peak E	83945	34.7
30-3I	EI transition	86943	100.0
30-3I	peak I	87444	10.0
30-3I	IE transition	88236	25.8
30-3I	peak E	88596	33.0
30-3I	EI transition	91952	100.0
30-3I	peak I	92272	4.8
30-3I	IE transition	93307	20.5
30-3I	peak E	93863	28.9
30-3I	EI transition	98567	100.0
30-4E	EI transition	50608	0.0
30-4E	peak I	50958	11.5
30-4E	IE transition	51783	38.6
30-4E	peak E	52369	57.8
30-4E	EI transition	53652	100.0
30-4E	peak I	54102	9.8
30-4E	IE transition	54861	26.3
30-4E	peak E	55250	34.8
30-4E	EI transition	58241	100.0
30-4E	peak I	58719	13.8
30-4E	IE transition	59661	40.9
30-4E	peak E	60099	53.6
30-4E	EI transition	61710	100.0
30-4E	peak I	62061	7.6
30-4E	IE transition	62847	24.7
30-4E	peak E	63264	33.8

29-3E	EI transition	53678	100.0
29-3E	peak I	54219	24.9
29-3E	IE transition	54651	44.7
29-3E	peak E	54875	55.0
29-3E	EI transition	55855	100.0
29-3E	peak I	56646	29.1
29-3E	IE transition	56901	38.5
29-3E	peak E	57550	62.4
29-3E	EI transition	58571	100.0
29-3E	peak I	59384	28.5
29-3E	IE transition	59828	44.1
29-3E	peak E	60513	68.1
29-3E	EI transition	61424	100.0
29-3E	peak I	62357	20.7
29-3E	IE transition	62855	31.7
29-3E	peak E	63325	42.2
29-3E	EI transition	65934	100.0
29-5E	EI transition	49818	0.0
29-5E	peak I	50191	13.3
29-5E	IE transition	50928	39.6
29-5E	peak E	51223	50.1
29-5E	EI transition	52624	100.0
29-5E	peak I	53594	36.2
29-5E	IE transition	53894	47.4
29-5E	peak E	54161	57.4
29-5E	EI transition	55303	100.0
29-5E	peak I	55739	16.0
29-5E	IE transition	56158	31.4
29-5E	peak E	56338	38.1
29-5E	EI transition	58022	100.0
29-5E	peak I	58732	26.3
29-5E	IE transition	59105	40.1
29-5E	peak E	59857	68.0
29-5E	EI transition	60722	100.0
29-5E	peak I	61696	31.7
29-5E	IE transition	62023	42.4
29-5E	peak E	62167	47.0
29-5E	EI transition	63794	100.0
29-5E	peak I	64305	17.2
29-5E	IE transition	64935	38.5
29-5E	peak E	65383	53.6
29-5E	EI transition	66761	100.0
29-5E	peak I	67163	14.1
29-5E	IE transition	67986	43.0
29-5E	peak E	68265	52.7
29-5E	EI transition	69613	100.0
29-5E	peak I	70337	22.4
29-5E	IE transition	70943	41.1
29-5E	peak E	71418	55.8
29-5E	EI transition	72849	100.0
29-5E	peak I	73314	15.2
29-5E	IE transition	74129	41.9
29-5E	peak E	74949	68.8
29-5E	EI transition	75902	100.0
29-5E	peak I	76760	22.0
29-5E	IE transition	77198	33.3
29-6I	EI transition	36057	0.0
29-6I	peak I	36483	13.7
29-6I	IE transition	37243	38.1
30-4E	EI transition	66314	100.0
30-4E	peak I	66775	9.9
30-4E	IE transition	67635	28.3
30-4E	peak E	67950	35.0
30-4E	EI transition	70986	100.0
30-4E	peak I	71440	9.1
30-4E	IE transition	72322	26.7
30-4E	peak E	72691	34.1
30-4E	EI transition	75989	100.0
30-4E	peak I	76299	5.0
30-4E	IE transition	77324	21.5
30-4E	peak E	77661	26.9
30-4E	EI transition	82204	100.0
30-4E	peak I	82672	12.2
30-4E	IE transition	83618	36.9
30-4E	peak E	84069	48.7
30-4E	EI transition	86033	100.0
30-4E	peak I	86498	6.9
30-4E	IE transition	87281	18.6
30-4E	peak E	87606	23.5
30-4E	EI transition	92731	100.0
30-5E	EI transition	59014	0.0
30-5E	peak I	59410	9.8
30-5E	IE transition	60263	31.0
30-5E	peak E	60665	40.9
30-5E	EI transition	63047	100.0
30-5E	peak I	63388	8.2
30-5E	IE transition	64359	31.4
30-5E	peak E	64797	41.9
30-5E	EI transition	67224	100.0
30-5E	peak I	67544	7.4
30-5E	IE transition	68590	31.4
30-5E	peak E	69037	41.7
30-5E	EI transition	71571	100.0
30-5E	peak I	71832	5.6
30-5E	IE transition	72853	27.7
30-5E	peak E	73188	35.0
30-5E	EI transition	76195	100.0
30-5E	peak I	76592	7.9
30-5E	IE transition	77524	26.5
30-5E	peak E	78036	36.7
30-5E	EI transition	81218	100.0
30-5E	peak I	81634	8.9
30-5E	IE transition	82629	30.1
30-5E	peak E	83205	42.3
30-5E	EI transition	85912	100.0
30-5E	peak I	86356	9.0
30-5E	IE transition	87212	26.3
30-5E	peak E	87671	35.6
30-5E	EI transition	90856	100.0
30-5E	peak I	91232	7.8
30-5E	IE transition	92177	27.4
30-5E	peak E	92750	39.4
30-5E	EI transition	95669	100.0
30-5E	peak I	95992	5.7
30-5E	IE transition	96963	22.7
30-5E	peak E	97367	29.8
30-5E	EI transition	101363	100.0
30-7I	EI transition	48641	0.0

29-6I	peak E	38341	73.3
29-6I	EI transition	39171	100.0
29-6I	peak I	39857	20.5
29-6I	IE transition	40334	34.8
29-6I	peak E	40798	48.7
29-6I	EI transition	42514	100.0
29-6I	peak I	43229	20.9
29-6I	IE transition	43909	40.7
29-6I	peak E	44461	56.8
29-6I	EI transition	45943	100.0
29-6I	peak I	46469	15.3
29-6I	IE transition	47423	43.0
29-6I	peak E	47843	55.2
29-6I	EI transition	49388	100.0
29-6I	peak I	50190	24.2
29-6I	IE transition	50716	40.0
29-6I	peak E	51358	59.4
29-6I	EI transition	52706	100.0
29-6I	peak I	53387	22.8
29-6I	IE transition	53927	40.9
29-6I	peak E	54435	57.9
29-6I	EI transition	55693	100.0
29-6I	peak I	56287	19.0
29-6I	IE transition	57031	42.9
29-6I	peak E	58578	92.5
29-6I	EI transition	58813	100.0
29-6I	peak I	59597	24.9
29-6I	IE transition	60103	41.0
29-6I	peak E	60760	61.9
29-6I	EI transition	61959	100.0
29-6I	peak I	62535	20.0
29-6I	IE transition	63175	42.3
29-6I	peak E	63619	57.8
29-6I	EI transition	64832	100.0
29-6I	peak I	65418	19.3
29-6I	IE transition	66132	42.8
29-6I	peak E	66564	57.0
29-6I	EI transition	67869	100.0

30-7I	peak I	48896	5.8
30-7I	IE transition	49900	28.4
30-7I	peak E	50446	40.7
30-7I	EI transition	53072	100.0
30-7I	peak I	53475	10.1
30-7I	IE transition	54346	31.9
30-7I	peak E	54725	41.4
30-7I	EI transition	57066	100.0
30-7I	peak I	57391	7.1
30-7I	IE transition	58424	29.5
30-7I	peak E	58908	40.0
30-7I	EI transition	61669	100.0
30-7I	peak I	61916	8.2
30-7I	IE transition	62429	25.2
30-7I	peak E	63110	47.8
30-7I	EI transition	64682	100.0
30-7I	peak I	65026	9.4
30-7I	IE transition	65965	35.2
30-7I	peak E	66777	57.4
30-7I	EI transition	68331	100.0
30-7I	peak I	68640	6.5
30-7I	IE transition	69663	28.0
30-7I	peak E	70134	37.8
30-7I	EI transition	73096	100.0
30-7I	peak I	73596	9.3
30-7I	IE transition	74599	28.0
30-7I	peak E	75238	39.9
30-7I	EI transition	78466	100.0
30-7I	peak I	78888	9.1
30-7I	IE transition	79873	30.2
30-7I	peak E	80326	39.9
30-7I	EI transition	83123	100.0
30-7I	peak I	83684	11.1
30-7I	IE transition	84575	28.6
30-7I	peak E	85316	43.2
30-7I	EI transition	88195	100.0
30-7I	peak I	88621	5.8
30-7I	IE transition	89526	18.0
30-7I	peak E	89871	22.7
30-7I	EI transition	95583	100.0

name, session	parameter	time (ms)	ratio (%)
31-2I	EI transition	40851	0.0
31-2I	peak I	41425	14.6
31-2I	IE transition	42415	39.7
31-2I	peak E	42829	50.3
31-2I	EI transition	44786	100.0
31-2I	peak I	45307	13.6
31-2I	IE transition	46301	39.6
31-2I	peak E	46864	54.3
31-2I	EI transition	48613	100.0
31-2I	peak I	49104	12.6
31-2I	IE transition	49979	35.0
31-2I	peak E	50838	56.9
31-2I	EI transition	52520	100.0
31-2I	peak I	53113	13.8
31-2I	IE transition	53987	34.1
31-2I	peak E	55562	70.7
31-2I	EI transition	56825	100.0
31-2I	peak I	57334	12.7
31-2I	IE transition	58230	34.9
31-2I	peak E	58641	45.2
31-2I	EI transition	60846	100.0
31-2I	peak I	61294	11.1
31-2I	IE transition	62286	35.6
31-2I	peak E	63442	64.2
31-2I	EI transition	64889	100.0
31-2I	peak I	65238	8.0
31-2I	IE transition	66288	32.0
31-2I	peak E	67509	60.0
31-2I	EI transition	69256	100.0
31-2I	peak I	69755	12.8
31-2I	IE transition	70700	37.1
31-2I	peak E	71615	60.5
31-2I	EI transition	73152	100.0
31-2I	peak I	73589	11.0
31-2I	IE transition	74566	35.5
31-2I	peak E	75154	50.3
31-2I	EI transition	77133	100.0
31-2I	peak I	77662	15.0
31-2I	IE transition	78470	37.8
31-2I	peak E	78834	48.1
31-2I	EI transition	80666	100.0
31-3E	EI transition	56027	0.0
31-3E	peak I	56679	13.6
31-3E	IE transition	57290	26.3
31-3E	peak E	58820	58.1
31-3E	EI transition	60830	100.0
31-3E	peak I	61511	13.7
31-3E	IE transition	62227	28.1
31-3E	peak E	64150	66.7
31-3E	EI transition	65804	100.0
31-3E	peak I	66286	11.6
31-3E	IE transition	67224	34.1
31-3E	peak E	68100	55.1
31-3E	EI transition	69969	100.0
31-3E	peak I	70516	11.2
31-3E	IE transition	71536	32.0
31-3E	peak E	72193	45.4

name, session	parameter	time (ms)	ratio (%)
32-2E	EI transition	33703	0.0
32-2E	peak I	34238	15.5
32-2E	IE transition	34880	34.2
32-2E	peak E	35227	44.3
32-2E	EI transition	37146	100.0
32-2E	peak I	37614	11.2
32-2E	IE transition	38253	26.4
32-2E	peak E	38427	30.5
32-2E	EI transition	41340	100.0
32-2E	peak I	41903	15.5
32-2E	IE transition	42487	31.5
32-2E	peak E	42803	40.2
32-2E	EI transition	44982	100.0
32-2E	peak I	45481	14.3
32-2E	IE transition	46079	31.4
32-2E	peak E	46362	39.5
32-2E	EI transition	48478	100.0
32-2E	peak I	49045	16.0
32-2E	IE transition	49700	34.4
32-2E	peak E	50002	42.9
32-2E	EI transition	52026	100.0
32-2E	peak I	52434	9.6
32-2E	IE transition	53262	29.2
32-2E	peak E	54829	66.2
32-2E	EI transition	56257	100.0
32-2E	peak I	56607	10.4
32-2E	IE transition	57242	29.1
32-2E	peak E	57489	36.4
32-2E	EI transition	59637	100.0
32-2E	peak I	59926	7.4
32-2E	IE transition	60642	25.9
32-2E	peak E	61139	38.6
32-2E	EI transition	63524	100.0
32-2E	peak I	64073	16.9
32-2E	IE transition	64692	36.1
32-2E	peak E	65033	46.6
32-2E	EI transition	66763	100.0
32-4I	EI transition	35709	0.0
32-4I	peak I	36064	11.1
32-4I	IE transition	36763	32.8
32-4I	peak E	36898	37.1
32-4I	EI transition	38918	100.0
32-4I	peak I	39285	13.7
32-4I	IE transition	39945	38.3
32-4I	peak E	40412	55.7
32-4I	EI transition	41601	100.0
32-4I	peak I	41896	11.8
32-4I	IE transition	42535	37.3
32-4I	peak E	42722	44.8
32-4I	EI transition	44103	100.0
32-4I	peak I	44434	8.6
32-4I	IE transition	44679	14.9
32-4I	peak E	47498	88.0
32-4I	EI transition	47963	100.0
32-4I	peak I	48349	13.2
32-4I	IE transition	48985	34.9
32-4I	peak E	49326	46.5

31-3E	EI transition	74864	100.0
31-3E	peak I	75817	24.4
31-3E	IE transition	76388	39.1
31-3E	peak E	77139	58.3
31-3E	EI transition	78764	100.0
31-3E	peak I	79266	12.2
31-3E	IE transition	80302	37.3
31-3E	peak E	80564	43.6
31-3E	EI transition	82888	100.0
31-3E	peak I	83615	18.0
31-3E	IE transition	84450	38.8
31-3E	peak E	84977	51.9
31-3E	EI transition	86915	100.0
31-3E	peak I	87431	12.6
31-3E	IE transition	88511	38.9
31-3E	peak E	89328	58.8
31-3E	EI transition	91016	100.0
31-3E	peak I	91701	16.0
31-3E	IE transition	92409	32.5
31-3E	peak E	93732	63.3
31-3E	EI transition	95304	100.0
31-5I	EI transition	38796	0.0
31-5I	peak I	39290	12.7
31-5I	IE transition	40366	40.5
31-5I	peak E	40941	55.3
31-5I	EI transition	42675	100.0
31-5I	peak I	43409	18.1
31-5I	IE transition	44042	33.7
31-5I	peak E	44319	40.5
31-5I	EI transition	46736	100.0
31-5I	peak I	47299	13.6
31-5I	IE transition	48039	31.5
31-5I	peak E	48809	50.1
31-5I	EI transition	50870	100.0
31-5I	peak I	51338	13.7
31-5I	IE transition	52231	39.8
31-5I	peak E	52990	62.0
31-5I	EI transition	54288	100.0
31-5I	peak I	54813	14.0
31-5I	IE transition	55686	37.3
31-5I	peak E	56209	51.2
31-5I	EI transition	58039	100.0
31-5I	peak I	58583	14.6
31-5I	IE transition	59374	35.9
31-5I	peak E	59541	40.4
31-5I	EI transition	61760	100.0
31-5I	peak I	62212	13.4
31-5I	IE transition	63063	38.6
31-5I	peak E	63503	51.6
31-5I	EI transition	65140	100.0
31-5I	peak I	65720	15.1
31-5I	IE transition	66653	39.4
31-5I	peak E	67186	53.3
31-5I	EI transition	68977	100.0
31-5I	peak I	69437	13.2
31-5I	IE transition	70269	37.0
31-5I	peak E	70667	48.3
31-5I	EI transition	72473	100.0
31-5I	peak I	72943	12.6
32-4I	EI transition	50891	100.0
32-4I	peak I	51301	18.8
32-4I	IE transition	51831	43.1
32-4I	peak E	52464	72.1
32-4I	EI transition	53074	100.0
32-4I	peak I	53369	10.4
32-4I	IE transition	53550	16.8
32-4I	peak E	53880	28.4
32-4I	EI transition	55915	100.0
32-4I	peak I	56368	12.7
32-4I	IE transition	56996	30.4
32-4I	peak E	57145	34.6
32-4I	EI transition	59468	100.0
32-4I	peak I	59921	11.2
32-4I	IE transition	60519	25.9
32-4I	peak E	60899	35.3
32-4I	EI transition	63525	100.0
32-4I	peak I	63989	13.3
32-4I	IE transition	64605	30.9
32-4I	peak E	64791	36.2
32-4I	EI transition	67020	100.0
32-5E	EI transition	30215	0.0
32-5E	peak I	30562	9.1
32-5E	IE transition	31305	28.6
32-5E	peak E	31649	37.6
32-5E	EI transition	34028	100.0
32-5E	peak I	34343	8.9
32-5E	IE transition	35055	29.0
32-5E	peak E	35301	36.0
32-5E	EI transition	37566	100.0
32-5E	peak I	37960	15.4
32-5E	IE transition	38598	40.4
32-5E	peak E	39034	57.5
32-5E	EI transition	40118	100.0
32-5E	peak I	40495	12.8
32-5E	IE transition	40855	25.0
32-5E	peak E	42760	89.6
32-5E	EI transition	43068	100.0
32-5E	peak I	44799	17.0
32-5E	IE transition	45029	19.3
32-5E	peak E	45395	22.9
32-5E	EI transition	53234	100.0
32-5E	peak I	53510	3.1
32-5E	IE transition	53705	5.3
32-5E	peak E	53880	7.2
32-5E	EI transition	62145	100.0
32-5E	peak I	62798	13.7
32-5E	IE transition	63402	26.3
32-5E	peak E	63746	33.5
32-5E	EI transition	66925	100.0
32-5E	peak I	67303	12.1
32-5E	IE transition	67964	33.2
32-5E	peak E	69079	68.8
32-5E	EI transition	70058	100.0
32-5E	peak I	70437	10.5
32-5E	IE transition	71135	29.8
32-5E	peak E	71578	42.0
32-5E	EI transition	73673	100.0
32-7I	EI transition	52849	0.0

31-5I	IE transition	73763	34.7
31-5I	peak E	74682	59.4
31-5I	EI transition	76191	100.0
31-7E	EI transition	48003	0.0
31-7E	peak I	48813	15.8
31-7E	IE transition	49717	33.4
31-7E	peak E	49894	36.8
31-7E	EI transition	53135	100.0
31-7E	peak I	53999	19.6
31-7E	IE transition	54803	37.9
31-7E	peak E	55416	51.8
31-7E	EI transition	57539	100.0
31-7E	peak I	58145	14.9
31-7E	IE transition	59018	36.5
31-7E	peak E	60937	83.8
31-7E	EI transition	61592	100.0
31-7E	peak I	62215	14.4
31-7E	IE transition	63076	34.3
31-7E	peak E	64783	73.7
31-7E	EI transition	65919	100.0
31-7E	peak I	66465	12.6
31-7E	IE transition	67567	38.0
31-7E	peak E	68854	67.6
31-7E	EI transition	70261	100.0
31-7E	peak I	70799	12.9
31-7E	IE transition	71723	35.2
31-7E	peak E	72580	55.8
31-7E	EI transition	74419	100.0
31-7E	peak I	74925	12.6
31-7E	IE transition	75899	36.9
31-7E	peak E	77220	69.9
31-7E	EI transition	78426	100.0
31-7E	peak I	79000	13.1
31-7E	IE transition	80031	36.5
31-7E	peak E	81363	66.8
31-7E	EI transition	82824	100.0
31-7E	peak I	83436	15.1
31-7E	IE transition	84358	37.9
31-7E	peak E	85373	63.1
31-7E	EI transition	86866	100.0

32-7I	peak I	53377	11.9
32-7I	IE transition	53931	24.4
32-7I	peak E	54657	40.7
32-7I	EI transition	57290	100.0
32-7I	peak I	57920	18.0
32-7I	IE transition	58386	31.3
32-7I	peak E	58599	37.3
32-7I	EI transition	60797	100.0
32-7I	peak I	61362	16.5
32-7I	IE transition	61765	28.2
32-7I	peak E	62019	35.6
32-7I	EI transition	64229	100.0
32-7I	peak I	64756	16.8
32-7I	IE transition	65270	33.2
32-7I	peak E	65587	43.4
32-7I	EI transition	67360	100.0
32-7I	peak I	67883	16.7
32-7I	IE transition	68419	33.8
32-7I	peak E	68631	40.6
32-7I	EI transition	70492	100.0
32-7I	peak I	71015	17.4
32-7I	IE transition	71470	32.5
32-7I	peak E	71738	41.4
32-7I	EI transition	73500	100.0
32-7I	peak I	74012	16.2
32-7I	IE transition	74500	31.6
32-7I	peak E	74720	38.5
32-7I	EI transition	76666	100.0
32-7I	peak I	77104	9.3
32-7I	IE transition	77633	20.5
32-7I	peak E	81253	97.5
32-7I	EI transition	81373	100.0
32-7I	peak I	81970	11.2
32-7I	IE transition	85113	69.9
32-7I	peak E	85519	77.5

name, session	parameter	time (ms)	ratio (%)
33-2I	EI transition	37873	0.0
33-2I	peak I	38382	16.2
33-2I	IE transition	38965	34.8
33-2I	peak E	39854	63.2
33-2I	EI transition	41008	100.0
33-2I	peak I	41582	12.0
33-2I	IE transition	42167	24.3
33-2I	peak E	42512	31.5
33-2I	EI transition	45777	100.0
33-2I	peak I	46665	24.3
33-2I	IE transition	47334	42.6
33-2I	peak E	47836	56.3
33-2I	EI transition	49433	100.0
33-2I	peak I	50264	25.6
33-2I	IE transition	50665	37.9
33-2I	peak E	51112	51.7
33-2I	EI transition	52680	100.0
33-2I	peak I	53355	22.5
33-2I	IE transition	53900	40.7
33-2I	peak E	54690	67.1
33-2I	EI transition	55676	100.0
33-2I	peak I	56324	22.3
33-2I	IE transition	56890	41.8
33-2I	peak E	57631	67.3
33-2I	EI transition	58582	100.0
33-2I	peak I	59229	22.3
33-2I	IE transition	59714	39.0
33-2I	peak E	60517	66.6
33-2I	EI transition	61488	100.0
33-2I	peak I	62114	20.4
33-2I	IE transition	62619	36.9
33-2I	peak E	62955	47.9
33-2I	EI transition	64553	100.0
33-2I	peak I	65106	19.1
33-2I	IE transition	65612	36.6
33-2I	peak E	66436	65.1
33-2I	EI transition	67443	100.0
33-2I	peak I	68053	17.0
33-2I	IE transition	68733	35.9
33-2I	peak E	69910	68.6
33-2I	EI transition	71041	100.0
33-3E	EI transition	36809	0.0
33-3E	peak I	37333	15.7
33-3E	IE transition	37982	35.1
33-3E	peak E	38331	45.6
33-3E	EI transition	40150	100.0
33-3E	peak I	40863	19.1
33-3E	IE transition	41629	39.7
33-3E	peak E	42256	56.5
33-3E	EI transition	43878	100.0
33-3E	peak I	44405	14.5
33-3E	IE transition	45137	34.5
33-3E	peak E	45558	46.1
33-3E	EI transition	47522	100.0
33-3E	peak I	48045	13.3
33-3E	IE transition	48750	31.2
33-3E	peak E	49262	44.3

name, session	parameter	time (ms)	ratio (%)
34-2I	EI transition	36573	0.0
34-2I	peak I	37351	27.9
34-2I	IE transition	37742	41.9
34-2I	peak E	38151	56.5
34-2I	EI transition	39365	100.0
34-2I	peak I	39990	20.1
34-2I	IE transition	40528	37.4
34-2I	peak E	40994	52.3
34-2I	EI transition	42477	100.0
34-2I	peak I	42958	16.6
34-2I	IE transition	43633	39.9
34-2I	peak E	44156	58.0
34-2I	EI transition	45371	100.0
34-2I	peak I	46004	21.9
34-2I	IE transition	46575	41.7
34-2I	peak E	47084	59.4
34-2I	EI transition	48255	100.0
34-2I	peak I	48932	21.3
34-2I	IE transition	49440	37.2
34-2I	peak E	49675	44.6
34-2I	EI transition	51438	100.0
34-2I	peak I	52015	19.4
34-2I	IE transition	52583	38.5
34-2I	peak E	53009	52.9
34-2I	EI transition	54409	100.0
34-2I	peak I	54819	12.7
34-2I	IE transition	55490	33.5
34-2I	peak E	55768	42.1
34-2I	EI transition	57633	100.0
34-2I	peak I	58195	18.9
34-2I	IE transition	58778	38.4
34-2I	peak E	58960	44.5
34-2I	EI transition	60614	100.0
34-2I	peak I	61254	20.0
34-2I	IE transition	61801	37.1
34-2I	peak E	62294	52.5
34-2I	EI transition	63811	100.0
34-2I	peak I	64184	11.8
34-2I	IE transition	64978	37.0
34-2I	peak E	65678	59.1
34-2I	EI transition	66968	100.0
34-4E	EI transition	54689	0.0
34-4E	peak I	55363	19.0
34-4E	IE transition	55897	34.0
34-4E	peak E	56127	40.5
34-4E	EI transition	58236	100.0
34-4E	peak I	58829	19.4
34-4E	IE transition	59295	34.7
34-4E	peak E	60073	60.2
34-4E	EI transition	61288	100.0
34-4E	peak I	61916	18.0
34-4E	IE transition	62466	33.9
34-4E	peak E	62921	46.9
34-4E	EI transition	64768	100.0
34-4E	peak I	65332	17.9
34-4E	IE transition	65899	35.9
34-4E	peak E	66394	51.6

33-3E	EI transition	51454	100.0
33-3E	peak I	52097	17.2
33-3E	IE transition	52698	33.3
33-3E	peak E	53295	49.3
33-3E	EI transition	55186	100.0
33-3E	peak I	55901	21.1
33-3E	IE transition	56485	38.3
33-3E	peak E	56829	48.5
33-3E	EI transition	58577	100.0
33-3E	peak I	59096	15.7
33-3E	IE transition	59816	37.5
33-3E	peak E	60373	54.3
33-3E	EI transition	61883	100.0
33-3E	peak I	62381	15.1
33-3E	IE transition	63125	37.6
33-3E	peak E	63602	52.0
33-3E	EI transition	65186	100.0
33-3E	peak I	65731	15.3
33-3E	IE transition	66384	33.7
33-3E	peak E	66925	48.8
33-3E	EI transition	68747	100.0
33-4I	EI transition	35638	0.0
33-4I	peak I	35995	10.3
33-4I	IE transition	36730	31.5
33-4I	peak E	37041	40.4
33-4I	EI transition	39109	100.0
33-4I	peak I	39669	16.4
33-4I	IE transition	40265	33.9
33-4I	peak E	40675	45.9
33-4I	EI transition	42522	100.0
33-4I	peak I	43252	19.0
33-4I	IE transition	43673	29.9
33-4I	peak E	43940	36.8
33-4I	EI transition	46372	100.0
33-4I	peak I	46992	18.0
33-4I	IE transition	47532	33.7
33-4I	peak E	47943	45.7
33-4I	EI transition	49812	100.0
33-4I	peak I	50513	18.9
33-4I	IE transition	51027	32.8
33-4I	peak E	51927	57.0
33-4I	EI transition	53521	100.0
33-4I	peak I	53895	13.9
33-4I	IE transition	54667	42.7
33-4I	peak E	55128	59.8
33-4I	EI transition	56207	100.0
33-4I	peak I	56866	22.3
33-4I	IE transition	57391	40.1
33-4I	peak E	57561	45.8
33-4I	EI transition	59162	100.0
33-4I	peak I	59716	19.1
33-4I	IE transition	60248	37.4
33-4I	peak E	61063	65.4
33-4I	EI transition	62068	100.0
33-4I	peak I	62575	15.4
33-4I	IE transition	63199	34.4
33-4I	peak E	64150	63.2
33-4I	EI transition	65360	100.0
33-4I	peak I	65825	14.8

34-4E	EI transition	67920	100.0
34-4E	peak I	68312	11.3
34-4E	IE transition	69082	33.5
34-4E	peak E	69575	47.7
34-4E	EI transition	71393	100.0
34-4E	peak I	71803	10.9
34-4E	IE transition	72611	32.5
34-4E	peak E	73043	44.0
34-4E	EI transition	75144	100.0
34-4E	peak I	75828	16.4
34-4E	IE transition	76504	32.6
34-4E	peak E	78616	83.3
34-4E	EI transition	79312	100.0
34-4E	peak I	79852	15.0
34-4E	IE transition	80594	35.6
34-4E	peak E	82441	86.9
34-4E	EI transition	82912	100.0
34-4E	peak I	83485	12.5
34-4E	IE transition	84161	27.2
34-4E	peak E	86104	69.5
34-4E	EI transition	87508	100.0
34-5I	EI transition	34922	0.0
34-5I	peak I	35592	20.3
34-5I	IE transition	36209	39.0
34-5I	peak E	36671	53.0
34-5I	EI transition	38225	100.0
34-5I	peak I	38779	13.8
34-5I	IE transition	39572	33.5
34-5I	peak E	40356	53.0
34-5I	EI transition	42244	100.0
34-5I	peak I	42858	19.9
34-5I	IE transition	43551	42.3
34-5I	peak E	43975	56.0
34-5I	EI transition	45333	100.0
34-5I	peak I	45916	18.7
34-5I	IE transition	46518	38.1
34-5I	peak E	46973	52.7
34-5I	EI transition	48444	100.0
34-5I	peak I	48886	14.2
34-5I	IE transition	49607	37.3
34-5I	peak E	50130	54.1
34-5I	EI transition	51558	100.0
34-5I	peak I	52063	15.8
34-5I	IE transition	52686	35.4
34-5I	peak E	53084	47.9
34-5I	EI transition	54746	100.0
34-5I	peak I	55205	15.1
34-5I	IE transition	55960	39.9
34-5I	peak E	56332	52.2
34-5I	EI transition	57785	100.0
34-5I	peak I	58274	18.1
34-5I	IE transition	58895	41.0
34-5I	peak E	59274	55.0
34-5I	EI transition	60492	100.0
34-5I	peak I	60881	12.3
34-5I	IE transition	61524	32.5
34-5I	peak E	61860	43.1
34-5I	EI transition	63667	100.0
34-5I	peak I	64312	18.3

33-4I	IE transition	66441	34.3
33-4I	peak E	66828	46.6
33-4I	EI transition	68509	100.0
33-5E	EI transition	34976	0.0
33-5E	peak I	35449	14.2
33-5E	IE transition	36122	34.3
33-5E	peak E	36488	45.3
33-5E	EI transition	38313	100.0
33-5E	peak I	38974	17.8
33-5E	IE transition	39611	35.0
33-5E	peak E	40345	54.8
33-5E	EI transition	42024	100.0
33-5E	peak I	42696	20.1
33-5E	IE transition	43207	35.4
33-5E	peak E	43619	47.7
33-5E	EI transition	45368	100.0
33-5E	peak I	46051	21.6
33-5E	IE transition	46554	37.5
33-5E	peak E	46957	50.3
33-5E	EI transition	48528	100.0
33-5E	peak I	48972	12.8
33-5E	IE transition	49718	34.4
33-5E	peak E	50077	44.8
33-5E	EI transition	51986	100.0
33-5E	peak I	52525	16.8
33-5E	IE transition	53106	34.8
33-5E	peak E	53528	47.9
33-5E	EI transition	55202	100.0
33-5E	peak I	55756	16.2
33-5E	IE transition	56387	34.6
33-5E	peak E	56847	48.0
33-5E	EI transition	58628	100.0
33-5E	peak I	58977	10.2
33-5E	IE transition	59788	33.8
33-5E	peak E	60179	45.2
33-5E	EI transition	62062	100.0
33-5E	peak I	62754	16.2
33-5E	IE transition	63622	36.5
33-5E	peak E	63931	43.7
33-5E	EI transition	66337	100.0

34-5I	IE transition	65054	39.4
34-5I	peak E	66237	73.1
34-5I	EI transition	67185	100.0
34-7E	EI transition	40064	0.0
34-7E	peak I	40515	13.1
34-7E	IE transition	41239	34.1
34-7E	peak E	42022	56.8
34-7E	EI transition	43512	100.0
34-7E	peak I	44007	14.6
34-7E	IE transition	44623	32.7
34-7E	peak E	45023	44.5
34-7E	EI transition	46907	100.0
34-7E	peak I	47357	14.2
34-7E	IE transition	48029	35.3
34-7E	peak E	48306	44.0
34-7E	EI transition	50084	100.0
34-7E	peak I	50548	13.7
34-7E	IE transition	51200	32.9
34-7E	peak E	51584	44.2
34-7E	EI transition	53474	100.0
34-7E	peak I	54055	18.0
34-7E	IE transition	54563	33.7
34-7E	peak E	54852	42.7
34-7E	EI transition	56700	100.0
34-7E	peak I	57384	21.4
34-7E	IE transition	57874	36.8
34-7E	peak E	58130	44.8
34-7E	EI transition	59892	100.0
34-7E	peak I	60369	14.3
34-7E	IE transition	61014	33.6
34-7E	peak E	61356	43.8
34-7E	EI transition	63235	100.0
34-7E	peak I	63648	12.6
34-7E	IE transition	64298	32.4
34-7E	peak E	64658	43.4
34-7E	EI transition	66515	100.0
34-7E	peak I	67118	17.1
34-7E	IE transition	67697	33.6
34-7E	peak E	68176	47.2
34-7E	EI transition	70032	100.0

name, session	parameter	time (ms)	ratio (%)
35-2E	EI transition	31671	0.0
35-2E	peak I	32048	11.6
35-2E	IE transition	32795	34.5
35-2E	peak E	33074	43.1
35-2E	EI transition	34925	100.0
35-2E	peak I	35290	11.8
35-2E	IE transition	36146	39.5
35-2E	peak E	36477	50.2
35-2E	EI transition	38014	100.0
35-2E	peak I	38436	13.8
35-2E	IE transition	39126	36.4
35-2E	peak E	39362	44.1
35-2E	EI transition	41073	100.0
35-2E	peak I	41605	16.9
35-2E	IE transition	42291	38.8
35-2E	peak E	42580	48.0
35-2E	EI transition	44215	100.0
35-2E	peak I	44586	11.6
35-2E	IE transition	45289	33.6
35-2E	peak E	45559	42.0
35-2E	EI transition	47416	100.0
35-2E	peak I	47795	12.6
35-2E	IE transition	48500	36.2
35-2E	peak E	48760	44.8
35-2E	EI transition	50413	100.0
35-2E	peak I	50930	18.7
35-2E	IE transition	51639	44.5
35-2E	peak E	51907	54.2
35-2E	EI transition	53170	100.0
35-2E	peak I	53608	14.5
35-2E	IE transition	54243	35.4
35-2E	peak E	54588	46.8
35-2E	EI transition	56197	100.0
35-2E	peak I	56598	12.2
35-2E	IE transition	57310	33.8
35-2E	peak E	57734	46.7
35-2E	EI transition	59487	100.0
35-4I	EI transition	29692	0.0
35-4I	peak I	30065	13.5
35-4I	IE transition	30865	42.5
35-4I	peak E	31251	56.5
35-4I	EI transition	32448	100.0
35-4I	peak I	32867	15.4
35-4I	IE transition	33491	38.3
35-4I	peak E	33878	52.6
35-4I	EI transition	35168	100.0
35-4I	peak I	35502	11.8
35-4I	IE transition	36242	37.8
35-4I	peak E	36508	47.2
35-4I	EI transition	38008	100.0
35-4I	peak I	38380	13.3
35-4I	IE transition	39060	37.6
35-4I	peak E	39422	50.6
35-4I	EI transition	40804	100.0
35-4I	peak I	41229	15.0
35-4I	IE transition	41910	39.0
35-4I	peak E	42179	48.4

name, session	parameter	time (ms)	ratio (%)
36-4E	EI transition	37266	0.0
36-4E	peak I	37705	12.4
36-4E	IE transition	38365	31.1
36-4E	peak E	38606	37.9
36-4E	EI transition	40797	100.0
36-4E	peak I	41154	12.9
36-4E	IE transition	41864	38.4
36-4E	peak E	42224	51.4
36-4E	EI transition	43574	100.0
36-4E	peak I	43812	7.7
36-4E	IE transition	44378	26.1
36-4E	peak E	44712	37.0
36-4E	EI transition	46655	100.0
36-4E	peak I	47129	15.2
36-4E	IE transition	47692	33.2
36-4E	peak E	48011	43.4
36-4E	EI transition	49783	100.0
36-4E	peak I	50203	12.6
36-4E	IE transition	50764	29.5
36-4E	peak E	51051	38.1
36-4E	EI transition	53112	100.0
36-4E	peak I	53542	12.6
36-4E	IE transition	54176	31.3
36-4E	peak E	54538	41.9
36-4E	EI transition	56513	100.0
36-4E	peak I	56973	14.3
36-4E	IE transition	57528	31.5
36-4E	peak E	57764	38.8
36-4E	EI transition	59736	100.0
36-4E	peak I	60144	11.0
36-4E	IE transition	60853	30.2
36-4E	peak E	61208	39.8
36-4E	EI transition	63435	100.0
36-4E	peak I	63859	12.5
36-4E	IE transition	64501	31.4
36-4E	peak E	64774	39.5
36-4E	EI transition	66828	100.0
36-5I	EI transition	30747	0.0
36-5I	peak I	31202	13.2
36-5I	IE transition	31769	29.6
36-5I	peak E	32184	41.6
36-5I	EI transition	34202	100.0
36-5I	peak I	34615	12.4
36-5I	IE transition	35223	30.6
36-5I	peak E	35427	36.7
36-5I	EI transition	37543	100.0
36-5I	peak I	38084	16.6
36-5I	IE transition	38580	31.9
36-5I	peak E	38892	41.5
36-5I	EI transition	40796	100.0
36-5I	peak I	41203	11.6
36-5I	IE transition	41982	33.8
36-5I	peak E	42204	40.1
36-5I	EI transition	44303	100.0
36-5I	peak I	44740	12.5
36-5I	IE transition	45460	33.1
36-5I	peak E	45639	38.2

35-4I	EI transition	43643	100.0
35-4I	peak I	44017	14.0
35-4I	IE transition	44674	38.7
35-4I	peak E	45038	52.3
35-4I	EI transition	46308	100.0
35-4I	peak I	46687	13.7
35-4I	IE transition	47296	35.8
35-4I	peak E	47577	45.9
35-4I	EI transition	49071	100.0
35-4I	peak I	49396	12.5
35-4I	IE transition	50044	37.5
35-4I	peak E	50231	44.7
35-4I	EI transition	51668	100.0
35-4I	peak I	51999	12.1
35-4I	IE transition	52646	35.8
35-4I	peak E	52902	45.2
35-4I	EI transition	54401	100.0
35-4I	peak I	54863	16.7
35-4I	IE transition	55485	39.2
35-4I	peak E	55852	52.5
35-4I	EI transition	57163	100.0
35-5E	EI transition	31773	0.0
35-5E	peak I	32028	7.7
35-5E	IE transition	32870	33.2
35-5E	peak E	33174	42.3
35-5E	EI transition	35084	100.0
35-5E	peak I	35395	9.9
35-5E	IE transition	36215	36.0
35-5E	peak E	36555	46.8
35-5E	EI transition	38226	100.0
35-5E	peak I	38620	11.5
35-5E	IE transition	39417	34.7
35-5E	peak E	39793	45.7
35-5E	EI transition	41657	100.0
35-5E	peak I	42037	11.2
35-5E	IE transition	42780	33.0
35-5E	peak E	43009	39.8
35-5E	EI transition	45056	100.0
35-5E	peak I	45459	12.9
35-5E	IE transition	46273	39.1
35-5E	peak E	46559	48.3
35-5E	EI transition	48170	100.0
35-5E	peak I	48560	12.0
35-5E	IE transition	49225	32.5
35-5E	peak E	49430	38.9
35-5E	EI transition	51413	100.0
35-5E	peak I	51776	11.8
35-5E	IE transition	52529	36.4
35-5E	peak E	52783	44.7
35-5E	EI transition	54480	100.0
35-5E	peak I	54815	11.0
35-5E	IE transition	55514	34.1
35-5E	peak E	55867	45.7
35-5E	EI transition	57515	100.0
35-5E	peak I	57818	10.4
35-5E	IE transition	58590	36.8
35-5E	peak E	59042	52.3
35-5E	EI transition	60434	100.0
35-5E	peak I	60963	16.3
36-5I	EI transition	47803	100.0
36-5I	peak I	48267	12.6
36-5I	IE transition	48782	26.7
36-5I	peak E	49070	34.5
36-5I	EI transition	51471	100.0
36-5I	peak I	51922	12.4
36-5I	IE transition	52589	30.7
36-5I	peak E	52826	37.2
36-5I	EI transition	55115	100.0
36-5I	peak I	55568	27.5
36-5I	IE transition	56213	66.5
36-5I	peak E	56501	84.0
36-5I	EI transition	56765	100.0
36-5I	peak I	56859	3.1
36-5I	IE transition	57366	19.5
36-5I	peak E	58393	52.9
36-5I	EI transition	59841	100.0
36-5I	peak I	60237	11.5
36-5I	IE transition	60684	24.6
36-5I	peak E	62772	85.4
36-5I	EI transition	63271	100.0
36-6I	EI transition	36240	0.0
36-6I	peak I	36622	11.3
36-6I	IE transition	37338	32.4
36-6I	peak E	37636	41.2
36-6I	EI transition	39628	100.0
36-6I	peak I	40009	10.2
36-6I	IE transition	40773	30.7
36-6I	peak E	41039	37.9
36-6I	EI transition	43354	100.0
36-6I	peak I	43812	12.0
36-6I	IE transition	44609	33.0
36-6I	peak E	44889	40.4
36-6I	EI transition	47156	100.0
36-6I	peak I	47569	12.0
36-6I	IE transition	48228	31.1
36-6I	peak E	48436	37.1
36-6I	EI transition	50606	100.0
36-6I	peak I	51080	14.4
36-6I	IE transition	51682	32.7
36-6I	peak E	51935	40.4
36-6I	EI transition	53893	100.0
36-6I	peak I	54291	12.5
36-6I	IE transition	54951	33.1
36-6I	peak E	55146	39.2
36-6I	EI transition	57088	100.0
36-6I	peak I	57519	13.3
36-6I	IE transition	58188	33.9
36-6I	peak E	58422	41.1
36-6I	EI transition	60331	100.0
36-6I	peak I	60747	12.6
36-6I	IE transition	61350	30.9
36-6I	peak E	61793	44.3
36-6I	EI transition	63630	100.0
36-6I	peak I	64034	12.7
36-6I	IE transition	64745	35.1
36-6I	peak E	65068	45.2
36-6I	EI transition	66811	100.0
36-6I	peak I	67303	15.2

35-5E	IE transition	61696	39.0
35-7I	EI transition	29426	0.0
35-7I	peak I	30002	18.1
35-7I	IE transition	30760	41.8
35-7I	peak E	30975	48.6
35-7I	EI transition	32615	100.0
35-7I	peak I	32938	10.6
35-7I	IE transition	33708	35.6
35-7I	peak E	34232	52.7
35-7I	EI transition	35685	100.0
35-7I	peak I	36117	15.4
35-7I	IE transition	36824	40.5
35-7I	peak E	37297	57.3
35-7I	EI transition	38498	100.0
35-7I	peak I	38804	10.4
35-7I	IE transition	39565	36.1
35-7I	peak E	39957	49.4
35-7I	EI transition	41454	100.0
35-7I	peak I	41852	12.1
35-7I	IE transition	42721	38.6
35-7I	peak E	43199	53.1
35-7I	EI transition	44738	100.0
35-7I	peak I	45076	11.2
35-7I	IE transition	45844	36.7
35-7I	peak E	46344	53.2
35-7I	EI transition	47754	100.0
35-7I	peak I	48121	13.0
35-7I	IE transition	48859	39.3
35-7I	peak E	49321	55.7
35-7I	EI transition	50568	100.0
35-7I	peak I	50850	9.9
35-7I	IE transition	51584	35.6
35-7I	peak E	52158	55.8
35-7I	EI transition	53420	100.0
35-7I	peak I	53728	10.6
35-7I	IE transition	54502	37.4
35-7I	peak E	54891	50.8
35-7I	EI transition	56316	100.0
35-7I	peak I	56594	9.3
35-7I	IE transition	57371	35.3
35-7I	peak E	57805	49.9
35-7I	EI transition	59301	100.0
36-6I	IE transition	67870	32.8
36-6I	peak E	68116	40.4
36-6I	EI transition	70040	100.0
36-7E	EI transition	37186	0.0
36-7E	peak I	37579	10.0
36-7E	IE transition	38275	27.6
36-7E	peak E	38644	37.0
36-7E	EI transition	41129	100.0
36-7E	peak I	41499	12.4
36-7E	IE transition	42098	32.6
36-7E	peak E	42538	47.4
36-7E	EI transition	44101	100.0
36-7E	peak I	44374	8.0
36-7E	IE transition	44769	19.5
36-7E	peak E	45077	28.5
36-7E	EI transition	47525	100.0
36-7E	peak I	47837	8.3
36-7E	IE transition	48590	28.5
36-7E	peak E	48921	37.3
36-7E	EI transition	51266	100.0
36-7E	peak I	51683	10.9
36-7E	IE transition	52312	27.4
36-7E	peak E	52545	33.5
36-7E	EI transition	55081	100.0
36-7E	peak I	55612	15.3
36-7E	IE transition	56112	29.8
36-7E	peak E	56453	39.6
36-7E	EI transition	58545	100.0
36-7E	peak I	59031	15.0
36-7E	IE transition	59666	34.6
36-7E	peak E	59992	44.6
36-7E	EI transition	61788	100.0
36-7E	peak I	62057	9.7
36-7E	IE transition	62514	26.1
36-7E	peak E	62740	34.3
36-7E	EI transition	64565	100.0
36-7E	peak I	64969	12.9
36-7E	IE transition	65505	30.1
36-7E	peak E	65784	39.0
36-7E	EI transition	67689	100.0
36-7E	peak I	68211	15.4
36-7E	IE transition	68819	33.4
