# Supplementary material for: Respiratory modulation of cognitive performance during the retrieval process
Source: PLoS One. 2018 Sep 14;13(9):e0204021. doi: 10.1371/journal.pone.0204021 (PMC6138381; doi:10.1371/journal.pone.0204021)
Supplement: S5 Table — (PDF) [file pone.0204021.s007.pdf]

Table S5. Individual test trials in the Non-phased sessions

									during retrieval (RT)					
name, session	cue (ms)	cue (deg)	retrieval duration (deg)	RT (ms)	accuracy	phase timing	over EI (O360)	over IE (O180)	I duration, 1st (ms)	I duration, 2nd (ms)	E duration, 1st (ms)	E duration, 2nd (ms)	over, 1st	over, 2nd
18-1S	30051	236.2	129.7	1389	1	E	1	0	25		1364		360	
18-1S	33334	316.6	419.3	2394	0									
18-1S	36650	221.1	132.5	1183	1	E	0	0	0		1183			
18-1S	39065	204.2	119.9	1583	0	E	0	0	0		1583			
18-1S	42648	208.2	62.1	1365	1	E	0	0	0		1365			
18-1S	45647	344.7	270.1	1167	1	E	1	1	446		335	386	360	180
18-1S	48647	214.4	103.1	1353	1	E	0	0	0		1353			
18-1S	51063	83.0	151.8	1156	1	I	0	1	591		565		180	
18-1S	54080	135.5	131.5	989	1	I	0	1	188		801		180	
18-1S	57363	224.3	261.4	1917	0	E	1	0	768		1149		360	
18-2S	30051	206.3	115.2	1083	1	E	0	0	0		1083			
18-2S	33334	267.2	81.7	1012	1	E	0	0	0		1012			
18-2S	36650	232.8	64.9	1151	1	E	0	0	0		1151			
18-2S	39649	139.3	150.3	1346	1	I	0	1	217		1129		180	
18-2S	42649	191.9	120.8	1268	1	E	0	0	0		1268			
18-2S	45349	201.5	113.2	1209	1	E	0	0	0		1209			
18-2S	48349	216.9	97.2	1051	1	E	0	0	0		1051			
18-2S	51366	178.7	159.6	1784	1	I	0	1	11		1773		180	
18-2S	54666	211.6	100.6	1451	1	E	0	0	0		1451			
18-2S	57366	103.4	129.2	937	1	I	0	1	416		521		180	
18-5S	30344	312.6	277.4	1493	1	E	1	1	743		430	320	360	180
18-5S	33061	81.8	159.4	987	1	I	0	1	472		515		180	
18-5S	36644	259.7	276.8	1618	1	E	1	0	701		917		360	
18-5S	39344	302.7	275.5	1251	1	E	1	1	552		499	200	360	180
18-5S	42642	195.4	89.7	2350	1	E	0	0	0		2350			
18-5S	45359	299.1	36.6	959	1	E	0	0	0		959			
18-5S	48659	324.5	219.5	963	1	E	1	1	725		215	23	360	180
18-5S	51359	115.0	121.4	999	1	I	0	1	415		584		180	
18-5S	54659	168.6	128.8	1328	1	I	0	1	69		1259		180	
18-5S	57075	77.3	163.6	1135	1	I	0	1	553		582		180	
18-8S	30645	320.8	322.9	1492	1	E	1	1	630		336	526	360	180
18-8S	33345	223.8	387.8	1616	1									

18-8S	36062	125.2	204.2	1877	1	I	0	1	275	1602		180	
18-8S	39079	202.3	179.8	1146	1	E	1	0	87	1059		360	
18-8S	42095	133.2	113.0	975	1	I	0	1	238	737		180	
18-8S	45661	225.4	131.2	2012	1	E	0	0	0	2012			
18-8S	48077	90.1	201.2	1207	1	I	0	1	351	856		180	
18-8S	51093	189.8	86.1	1040	1	E	0	0	0	1040			
18-8S	54659	236.3	327.4	2183	1	E	1	1	719	1322	142	360	180
18-8S	57075	242.7	139.1	1148	1	E	1	0	446	702		360	
19-1S	30339	195.6	76.4	978	1	E	0	0	0	978			
19-1S	33056	117.1	116.0	992	0	I	0	1	330	662		180	
19-1S	36339	121.4	94.0	867	1	I	0	1	341	526		180	
19-1S	39355	0.2	182.4	1079	1	I	0	1	1041	38		180	
19-1S	42070	291.5	126.6	1273	1	E	1	0	244	1029		360	
19-1S	45353	344.2	144.8	863	1	E	1	0	719	144		360	
19-1S	48353	327.4	119.1	864	1	E	1	0	454	410		360	
19-1S	51069	270.8	57.6	864	1	E	0	0	0	864			
19-1S	54086	248.8	62.3	805	1	E	0	0	0	805			
19-1S	57669	310.5	101.1	728	1	E	1	0	204	524		360	
19-2S	30346	350.0	152.5	851	1	E	1	0	705	146		360	
19-2S	33063	320.0	210.8	1151	1	E	1	0	671	480		360	
19-2S	36080	57.0	122.5	784	1	I	0	0	784	0			
19-2S	39345	11.6	177.8	1174	1	I	0	1	1033	141		180	
19-2S	42645	331.0	93.8	714	1	E	1	0	278	436		360	
19-2S	45645	344.2	162.4	924	1	E	1	0	752	172		360	
19-2S	48661	332.9	163.2	1103	1	E	1	0	763	340		360	
19-2S	51361	270.6	54.4	811	1	E	0	0	0	811			
19-2S	54077	226.4	58.0	781	1	E	0	0	0	781			
19-2S	57660	226.7	48.7	825	1	E	0	0	0	825			
19-5S	30053	116.2	101.0	932	1	I	0	1	370	562		180	
19-5S	33070	355.2	139.2	821	1	E	1	0	749	72		360	
19-5S	36353	337.2	109.2	696	1	E	1	0	375	321		360	
19-5S	39069	330.8	124.1	688	1	E	1	0	376	312		360	
19-5S	42651	113.5	187.4	2174	1	I	0	1	432	1742		180	
19-5S	45068	317.7	262.3	1309	1	E	1	1	465	610	234	360	180
19-5S	48351	210.0	53.2	639	1	E	0	0	0	639			
19-5S	51067	178.4	56.0	741	1	I	0	1	8	733		180	
19-5S	54667	196.8	54.3	749	1	E	0	0	0	749			
19-5S	57667	180.5	73.0	941	1	E	0	0	0	941			
19-8S	30335	346.0	196.0	1144	1	E	1	1	934	187	23	360	180

19-8S	33051	319.8	92.5	668	1	E	1	0	210	458		360	
19-8S	36068	333.1	168.4	1146	0	E	1	0	823	323		360	
19-8S	39083	305.4	206.9	1288	1	E	1	0	572	716		360	
19-8S	42350	24.3	161.4	879	1	I	0	1	794	85		180	
19-8S	45649	348.6	200.9	1247	0	E	1	1	963	170	114	360	180
19-8S	48349	310.7	297.0	1994	1	E	1	1	659	591	744	360	180
19-8S	51065	313.4	217.1	1287	1	E	1	0	775	512		360	
19-8S	54665	28.7	138.9	886	1	I	0	0	886	0			
19-8S	57365	303.6	50.6	711	1	E	0	0	0	711			
20-1S	30051	276.5	192.2	1381	1	E	1	0	613	768		360	
20-1S	33334	316.0	213.7	1412	1	E	1	0	927	485		360	
20-1S	36651	23.8	235.0	1878	1	I	0	1	980	898		180	
20-1S	39651	357.2	211.7	1431	1	E	1	1	1107	32	292	360	180
20-1S	42068	306.6	307.6	2292	1	E	1	1	1150	539	603	360	180
20-1S	45650	81.6	138.7	925	1	I	0	1	521	404		180	
20-1S	48350	62.9	203.9	1689	1	I	0	1	700	989		180	
20-1S	51666	93.0	217.6	2295	1	I	0	1	528	1767		180	
20-1S	54666	7.3	296.4	2162	1	I	0	1	897	1265		180	
20-1S	57665	49.3	160.0	968	1	I	0	1	692	276		180	
20-4S	30634	163.9	116.1	1103	1	I	0	1	89	1014		180	
20-4S	33334	134.0	146.4	1377	0	I	0	1	270	1107		180	
20-4S	36651	175.9	100.2	1030	1	I	0	1	25	1005		180	
20-4S	39067	83.1	141.2	1042	1	I	0	1	593	449		180	
20-4S	42351	151.7	96.2	849	1	I	0	1	161	688		180	
20-4S	45067	136.1	89.0	700	1	I	0	1	236	464		180	
20-4S	48082	161.3	95.0	870	1	I	0	1	108	762		180	
20-4S	51099	152.0	128.4	1871	1	I	0	1	205	1666		180	
20-4S	54666	60.2	120.9	754	1	I	0	1	743	11		180	
20-4S	57666	74.3	134.6	878	1	I	0	1	596	282		180	
20-7S	30060	28.6	121.1	2052	0	I	0	0	2052	0			
20-7S	33343	203.6	52.2	1589	0	E	0	0	0	1589			
20-7S	36343	302.3	93.4	1976	1	E	1	0	220	1756		360	
20-7S	39642	205.3	123.6	2108	0	E	0	0	0	2108			
20-7S	42342	9.7	124.1	804	0	I	0	0	804	0			
20-7S	45658	300.6	59.4	1091	1	E	0	0	0	1091			
20-7S	48075	185.4	77.3	1256	1	E	0	0	0	1256			
20-7S	51092	31.5	286.2	2887	0	I	0	1	840	2047		180	
20-7S	54658	7.3	114.0	778	1	I	0	0	778	0			
20-7S	57358	323.1	132.5	917	1	E	1	0	524	393		360	

20-8S	30342	36.4	176.4	1412	1	I	0	1	895		517		180	
20-8S	33058	295.7	209.7	1944	1	E	1	0	932		1012		360	
20-8S	36074	244.0	86.1	1144	1	E	0	0	0		1144			
20-8S	39091	223.7	354.1	2767	1	E	1	1	1093		1194	480	360	180
20-8S	42658	280.9	339.6	3056	0	E	1	1	1281		1003	773	360	180
20-8S	45657	254.5	73.8	709	0	E	0	0	0		709			
20-8S	48657	347.4	244.2	1807	1	E	1	1	1239		94	474	360	180
20-8S	51357	328.9	282.8	2227	1	E	1	1	1089		285	853	360	180
20-8S	54357	316.7	228.9	1165	1	E	1	1	616		515	34	360	180
20-8S	57674	203.0	136.3	1946	1	E	0	0	0		1946			
21-1S	30634	216.3	116.9	929	1	E	0	0	0		929			
21-1S	33051	214.0	176.0	1240	1	E	1	0	155		1085		360	
21-1S	36067	319.8	278.9	1724	1	E	1	1	978		288	458	360	180
21-1S	39650	167.9	377.8	2334	1									
21-1S	42650	275.4	173.1	1085	1	E	1	0	457		628		360	
21-1S	45350	1.1	245.0	1460	1	I	0	1	957		503		180	
21-1S	48648	176.9	146.7	1185	1	I	0	1	17		1168		180	
21-1S	51364	214.2	157.1	1138	1	E	1	0	59		1079		360	
21-1S	54364	311.9	137.9	855	1	E	1	0	497		358		360	
21-1S	57664	111.8	124.7	818	1	I	0	1	389		429		180	
21-4S	30638	146.6	104.2	757	1	I	0	1	199		558		180	
21-4S	33055	140.4	133.3	977	1	I	0	1	226		751		180	
21-4S	36072	220.1	108.9	963	1	E	0	0	0		963			
21-4S	39355	318.1	182.7	1034	1	E	1	0	717		317		360	
21-4S	42355	75.6	162.3	964	1	I	0	1	561		403		180	
21-4S	45071	165.0	258.5	2045	1	I	1	1	82	337	1626		180	360
21-4S	48087	234.0	217.2	1306	1	E	1	0	479		827		360	
21-4S	51654	115.6	269.6	2027	1	I	1	1	330	135	1562		180	360
21-4S	54070	97.7	225.2	1596	1	I	0	1	441		1155		180	
21-4S	57087	199.7	311.9	1926	1	E	1	0	800		1126		360	
21-7S	30631	352.6	248.7	1543	1	E	1	1	974		66	503	360	180
21-7S	33348	36.5	135.9	745	1	I	0	0	745		0			
21-7S	36648	200.6	354.3	2323	1	E	1	1	929		1292	102	360	180
21-7S	39348	250.2	284.2	1582	1	E	1	0	832		750		360	
21-7S	42647	87.6	279.0	777	1	I	1	1	429	32	316		180	360
21-7S	45647	263.8	75.3	1252	1	E	0	0	0		1252			
21-7S	48363	196.3	118.9	1114	1	E	0	0	0		1114			
21-7S	51080	198.2	153.4	1598	1	E	0	0	0		1598			
21-7S	54097	208.3	67.1	1050	1	E	0	0	0		1050			

21-7S	57364	167.3	120.0	1013	1	I	0	1	68	945		180	
21-8S	30344	312.1	158.9	1019	1	E	1	0	620	399		360	
21-8S	33344	16.1	286.0	2015	0	I	0	1	904	1111		180	
21-8S	36061	30.4	283.9	1975	1	I	0	1	865	1110		180	
21-8S	39644	202.7	99.7	794	1	E	0	0	0	794			
21-8S	42644	277.7	248.8	1511	1	E	1	0	882	629		360	
21-8S	45360	334.3	213.5	1204	1	E	1	1	958	189	57	360	180
21-8S	48658	152.2	202.3	1652	1	I	0	1	151	1501		180	
21-8S	51658	224.4	408.2	2728	0								
21-8S	54658	306.3	145.6	915	1	E	1	0	482	433		360	
21-8S	57658	55.7	255.2	1789	1	I	0	1	725	1064		180	
22-1S	30343	312.5	132.0	928	1	E	1	0	459	469		360	
22-1S	33643	119.2	123.7	743	1	I	0	1	295	448		180	
22-1S	36643	242.3	117.2	986	1	E	0	0	0	986			
22-1S	39643	305.3	120.5	779	1	E	1	0	333	446		360	
22-1S	42643	77.7	217.3	1334	1	I	0	1	512	822		180	
22-1S	45060	120.4	100.7	667	1	I	0	1	306	361		180	
22-1S	48659	263.5	102.0	925	1	E	1	0	32	893		360	
22-1S	51076	239.7	79.3	642	1	E	0	0	0	642			
22-1S	54659	45.0	185.4	1097	1	I	0	1	714	383		180	
22-1S	57076	67.2	156.1	822	1	I	0	1	564	258		180	
22-4S	30643	68.9	172.4	1098	1	I	0	1	600	498		180	
22-4S	33643	170.7	100.3	862	1	I	0	1	51	811		180	
22-4S	36060	135.1	141.4	989	1	I	0	1	253	736		180	
22-4S	39343	245.7	73.7	744	1	E	0	0	0	744			
22-4S	42060	245.1	270.2	1881	1	E	1	0	786	1095		360	
22-4S	45660	70.0	200.4	1287	1	I	0	1	575	712		180	
22-4S	48359	128.5	127.3	911	1	I	0	1	284	627		180	
22-4S	51359	200.0	89.8	861	1	E	0	0	0	861			
22-4S	54659	252.4	254.6	1898	0	E	1	0	778	1120		360	
22-4S	57659	301.4	232.2	1428	1	E	1	0	981	447		360	
22-7S	30054	285.8	84.0	1033	1	E	1	0	54	979		360	
22-7S	33637	333.6	118.8	821	1	E	1	0	544	277		360	
22-7S	36337	337.1	141.1	800	1	E	1	0	601	199		360	
22-7S	39353	95.8	159.1	1026	1	I	0	1	432	594		180	
22-7S	42653	228.1	66.1	655	1	E	0	0	0	655			
22-7S	45653	254.6	63.2	594	1	E	0	0	0	594			
22-7S	48652	273.1	116.3	1038	1	E	1	0	159	879		360	
22-7S	51068	256.0	263.0	1605	0	E	1	0	831	774		360	

22-7S	54085	333.1	207.5	1267	1	E	1	1	1033	229	5	360	180
22-7S	57101	50.4	136.6	773	1	I	0	1	715	58		180	
22-8S	30637	137.9	166.1	1685	1	I	0	1	234	1451		180	
22-8S	33637	123.0	92.9	643	1	I	0	1	305	338		180	
22-8S	36337	135.2	116.9	897	1	I	0	1	233	664		180	
22-8S	39354	193.2	106.2	1119	1	E	0	0	0	1119			
22-8S	42654	228.0	106.3	1143	1	E	0	0	0	1143			
22-8S	45071	179.5	145.2	1376	1	I	0	1	3	1373		180	
22-8S	48087	208.0	85.6	786	1	E	0	0	0	786			
22-8S	51654	310.8	98.3	773	1	E	1	0	314	459		360	
22-8S	54671	292.6	60.4	754	1	E	0	0	0	754			
22-8S	57671	262.9	67.6	814	1	E	0	0	0	814			
23-1S	30345	292.7	103.0	1005	1	E	1	0	226	779		360	
23-1S	33345	276.8	51.2	572	1	E	0	0	0	572			
23-1S	36345	282.9	133.0	1176	1	E	1	0	360	816		360	
23-1S	39345	270.8	64.3	725	1	E	0	0	0	725			
23-1S	42345	51.7	130.3	651	1	I	0	1	637	14		180	
23-1S	45061	151.4	81.4	535	1	I	0	1	155	380		180	
23-1S	48077	248.0	76.7	712	1	E	0	0	0	712			
23-1S	51660	350.5	133.9	540	1	E	1	0	456	84		360	
23-1S	54077	134.6	92.2	558	1	I	0	1	214	344		180	
23-1S	57660	339.6	236.7	1253	1	E	1	1	870	151	232	360	180
23-2S	30331	229.1	68.7	732	0	E	0	0	0	732			
23-2S	33047	211.2	75.8	766	1	E	0	0	0	766			
23-2S	36647	253.8	87.3	1227	1	E	0	0	0	1227			
23-2S	39347	209.2	71.4	761	0	E	0	0	0	761			
23-2S	42646	238.3	84.8	897	1	E	0	0	0	897			
23-2S	45346	213.6	60.7	581	0	E	0	0	0	581			
23-2S	48062	199.2	112.0	1160	0	E	0	0	0	1160			
23-2S	51362	237.7	84.9	870	1	E	0	0	0	870			
23-2S	54078	211.0	109.9	1256	1	E	0	0	0	1256			
23-2S	57361	230.2	93.1	1002	1	E	0	0	0	1002			
23-5S	30641	124.1	84.9	607	1	I	0	1	351	256		180	
23-5S	33057	79.2	109.1	674	1	I	0	1	603	71		180	
23-5S	36341	193.6	102.5	969	0	E	0	0	0	969			
23-5S	39341	222.6	74.1	712	1	E	0	0	0	712			
23-5S	42658	265.2	192.5	1673	1	E	1	0	558	1115		360	
23-5S	45658	275.0	144.7	1130	1	E	1	0	364	766		360	
23-5S	48657	312.4	76.3	577	1	E	1	0	169	408		360	

23-5S	51074	281.6	72.3	676	1	E	0	0	0	676			
23-5S	54091	302.3	84.3	732	1	E	1	0	164	568		360	
23-5S	57674	24.7	210.3	1287	1	I	0	1	907	380		180	
23-8S	30340	289.8	55.6	821	1	E	0	0	0	821			
23-8S	33056	235.3	69.3	870	1	E	0	0	0	870			
23-8S	36640	243.7	86.7	1223	1	E	0	0	0	1223			
23-8S	39056	122.6	88.3	678	1	I	0	1	363	315		180	
23-8S	42072	135.0	95.1	767	1	I	0	1	273	494		180	
23-8S	45088	152.8	131.6	1365	1	I	0	1	172	1193		180	
23-8S	48654	205.0	64.1	600	1	E	0	0	0	600			
23-8S	51654	214.9	62.7	658	1	E	0	0	0	658			
23-8S	54070	133.5	92.7	754	1	I	0	1	311	443		180	
23-8S	57670	226.2	65.1	670	1	E	0	0	0	670			
24-1S	30335	243.2	60.5	958	1	E	0	0	0	958			
24-1S	33335	166.0	47.3	676	1	I	0	1	97	579		180	
24-1S	36352	348.1	135.3	1018	1	E	1	0	812	206		360	
24-1S	39069	331.7	108.3	788	1	E	1	0	540	248		360	
24-1S	42652	19.1	172.0	1150	1	I	0	1	1019	131		180	
24-1S	45069	299.0	63.8	736	1	E	1	0	20	716		360	
24-1S	48651	321.2	78.4	725	1	E	1	0	288	437		360	
24-1S	51351	267.2	142.7	1409	1	E	1	0	393	1016		360	
24-1S	54667	241.7	71.5	1023	1	E	0	0	0	1023			
24-1S	57084	103.1	86.6	672	1	I	0	1	540	132		180	
24-2S	30344	223.2	74.4	978	1	E	0	0	0	978			
24-2S	33344	170.5	88.6	904	1	I	0	1	67	837		180	
24-2S	36643	190.3	70.8	736	1	E	0	0	0	736			
24-2S	39643	180.5	140.7	2340	1	E	0	0	0	2340			
24-2S	42343	342.8	214.9	1736	1	E	1	1	1283	286	167	360	180
24-2S	45660	7.6	106.7	712	1	I	0	0	712	0			
24-2S	48076	291.2	59.4	676	1	E	0	0	0	676			
24-2S	51359	258.7	44.8	678	1	E	0	0	0	678			
24-2S	54658	217.7	62.2	778	1	E	0	0	0	778			
24-2S	57075	92.5	105.5	859	1	I	0	1	603	256		180	
24-5S	30642	290.7	53.1	1421	1	E	0	0	0	1421			
24-5S	33642	156.7	53.6	883	1	I	0	1	170	713		180	
24-5S	36342	287.7	29.4	692	1	E	0	0	0	692			
24-5S	39642	199.5	50.7	623	1	E	0	0	0	623			
24-5S	42659	148.1	53.0	672	1	I	0	1	225	447		180	
24-5S	45359	297.2	110.8	1661	1	E	1	0	334	1327		360	

24-5S	48075	193.1	75.8	797	1	E	0	0	0	797			
24-5S	51358	197.3	85.6	1089	1	E	0	0	0	1089			
24-5S	54357	126.2	96.4	899	1	I	0	1	396	503		180	
24-5S	57357	74.1	108.5	716	1	I	0	1	684	32		180	
24-8S	30339	153.7	159.3	2281	1	I	0	1	190	2091		180	
24-8S	33055	340.7	95.5	854	1	E	1	0	550	304		360	
24-8S	36072	267.0	41.6	676	1	E	0	0	0	676			
24-8S	39338	206.1	36.0	633	1	E	0	0	0	633			
24-8S	42055	0.6	48.6	630	1	I	0	0	630	0			
24-8S	45655	247.7	75.2	1415	1	E	0	0	0	1415			
24-8S	48654	125.8	69.3	653	1	I	0	1	382	271		180	
24-8S	51354	309.3	110.9	1356	1	E	1	0	447	909		360	
24-8S	54654	264.6	105.8	1264	1	E	1	0	75	1189		360	
24-8S	57070	169.9	55.9	687	1	I	0	1	73	614		180	
26-1S	30059	41.6	189.0	1393	1	I	0	1	893	500		180	
26-1S	33641	145.1	83.7	680	1	I	0	1	219	461		180	
26-1S	36341	133.6	145.3	1124	1	I	0	1	270	854		180	
26-1S	39057	149.5	130.1	1128	0	I	0	1	182	946		180	
26-1S	42357	212.1	95.6	969	1	E	0	0	0	969			
26-1S	45074	200.6	65.6	664	1	E	0	0	0	664			
26-1S	48090	207.3	108.0	1213	1	E	0	0	0	1213			
26-1S	51356	223.6	76.6	868	1	E	0	0	0	868			
26-1S	54071	188.7	118.8	1585	0	E	0	0	0	1585			
26-1S	57087	121.4	153.3	1424	1	I	0	1	353	1071		180	
26-4S	30631	308.1	95.3	775	1	E	1	0	235	540		360	
26-4S	33047	280.9	262.1	1748	1	E	1	1	1010	706	32	360	180
26-4S	36347	328.7	287.4	2171	1	E	1	1	943	334	894	360	180
26-4S	39647	352.2	99.5	687	1	E	1	0	595	92		360	
26-4S	42064	269.5	94.1	1191	1	E	1	0	21	1170		360	
26-4S	45347	265.8	49.8	610	1	E	0	0	0	610			
26-4S	48364	250.4	61.4	628	1	E	0	0	0	628			
26-4S	51080	218.9	104.9	1279	0	E	0	0	0	1279			
26-4S	54095	201.3	91.1	1019	1	E	0	0	0	1019			
26-4S	57361	218.9	69.0	773	1	E	0	0	0	773			
26-7S	30643	295.6	52.0	632	1	E	0	0	0	632			
26-7S	33342	247.4	108.3	1315	1	E	0	0	0	1315			
26-7S	36642	246.8	90.5	1186	1	E	0	0	0	1186			
26-7S	39059	144.6	83.0	816	1	I	0	1	229	587		180	
26-7S	42659	183.1	60.1	647	1	E	0	0	0	647			

26-7S	45359	127.9	90.3	674	0	I	0	1	324	350		180	
26-7S	48359	173.7	72.9	621	1	I	0	1	37	584		180	
26-7S	51359	205.5	81.2	821	1	E	0	0	0	821			
26-7S	54075	185.7	51.7	586	1	E	0	0	0	586			
26-7S	57358	194.6	66.9	880	1	E	0	0	0	880			
26-8S	30638	358.4	229.8	1456	1	E	1	1	1071	16	369	360	180
26-8S	33053	353.6	205.3	1269	1	E	1	1	1056	49	164	360	180
26-8S	36069	61.2	176.3	1131	1	I	0	1	678	453		180	
26-8S	39652	226.3	100.9	1000	1	E	0	0	0	1000			
26-8S	42652	243.8	101.0	958	1	E	0	0	0	958			
26-8S	45352	242.2	124.1	1106	1	E	1	0	33	1073		360	
26-8S	48351	288.8	117.0	904	1	E	1	0	254	650		360	
26-8S	51651	358.9	196.7	1253	1	E	1	1	1112	10	131	360	180
26-8S	54066	334.4	119.6	770	1	E	1	0	555	215		360	
26-8S	57666	135.0	96.2	674	1	I	0	1	257	417		180	
27-1S	30052	187.4	113.4	1303	1	E	0	0	0	1303			
27-1S	33334	214.4	203.2	1718	1	E	1	0	327	1391		360	
27-1S	36051	207.7	82.5	907	1	E	0	0	0	907			
27-1S	39067	200.7	123.4	1362	1	E	0	0	0	1362			
27-1S	42650	253.3	71.1	701	1	E	0	0	0	701			
27-1S	45650	243.0	103.6	1297	1	E	0	0	0	1297			
27-1S	48650	210.2	116.8	1439	1	E	0	0	0	1439			
27-1S	51067	95.4	145.9	1118	1	I	0	1	507	611		180	
27-1S	54082	117.7	187.4	1600	1	I	0	1	378	1222		180	
27-1S	57665	208.3	152.6	1762	1	E	1	0	6	1756		360	
27-3S	30636	223.9	117.3	1305	0	E	0	0	0	1305			
27-3S	33051	155.7	135.9	1164	1	I	0	1	141	1023		180	
27-3S	36351	212.8	141.2	1701	1	E	0	0	0	1701			
27-3S	39351	200.8	130.0	1547	1	E	0	0	0	1547			
27-3S	42651	204.9	180.9	1960	1	E	1	0	169	1791		360	
27-3S	45068	95.4	178.2	1522	1	I	0	1	555	967		180	
27-3S	48084	97.6	119.5	840	1	I	0	1	507	333		180	
27-3S	51351	188.7	73.4	811	1	E	0	0	0	811			
27-3S	54067	144.4	155.1	1386	1	I	0	1	203	1183		180	
27-3S	57667	214.6	97.5	1111	1	E	0	0	0	1111			
27-5S	30642	187.1	226.9	1917	1	E	1	0	309	1608		360	
27-5S	33057	141.2	142.0	1407	0	I	0	1	222	1185		180	
27-5S	36074	120.1	138.5	1075	0	I	0	1	364	711		180	
27-5S	39340	195.0	110.9	1123	0	E	0	0	0	1123			

27-5S	42357	201.7	122.5	1380	1	E	0	0	0	1380			
27-5S	45074	146.9	105.4	948	1	I	0	1	210	738		180	
27-5S	48091	163.8	146.3	1322	1	I	0	1	96	1226		180	
27-5S	51657	241.4	92.1	946	1	E	0	0	0	946			
27-5S	54073	188.5	136.0	1569	1	E	0	0	0	1569			
27-5S	57090	63.9	57.9	941	1	I	0	0	941	0			
27-8S	30645	176.5	115.0	1549	1	I	0	1	24	1525		180	
27-8S	33644	83.3	208.4	1779	0	I	0	1	596	1183		180	
27-8S	36061	351.9	129.4	833	1	E	1	0	747	86		360	
27-8S	39644	103.2	190.1	1772	0	I	0	1	487	1285		180	
27-8S	42061	350.1	150.9	1017	1	E	1	0	905	112		360	
27-8S	45661	138.6	120.4	830	1	I	0	1	255	575		180	
27-8S	48078	130.1	118.3	1688	0	I	0	1	327	1361		180	
27-8S	51662	343.7	112.9	903	1	E	1	0	579	324		360	
27-8S	54078	256.3	78.3	1040	1	E	0	0	0	1040			
27-8S	57094	232.9	79.5	862	1	E	0	0	0	862			
28-1S	30057	233.4	180.2	1918	1	E	1	0	326	1592		360	
28-1S	33639	267.0	163.3	1359	1	E	1	0	402	957		360	
28-1S	36639	275.7	287.3	2222	1	E	1	1	1079	893	250	360	180
28-1S	39639	274.3	118.6	1137	1	E	1	0	203	934		360	
28-1S	42356	240.8	64.8	716	1	E	0	0	0	716			
28-1S	45656	278.3	320.2	2493	0	E	1	1	984	787	722	360	180
28-1S	48355	255.2	94.5	1167	1	E	0	0	0	1167			
28-1S	51355	235.5	50.4	657	1	E	0	0	0	657			
28-1S	54354	219.5	105.9	1241	1	E	0	0	0	1241			
28-1S	57671	239.0	77.0	820	1	E	0	0	0	820			
28-3S	30342	185.7	135.9	1314	1	E	0	0	0	1314			
28-3S	33641	232.9	65.1	720	1	E	0	0	0	720			
28-3S	36341	200.5	110.1	1165	1	E	0	0	0	1165			
28-3S	39641	235.4	83.8	845	1	E	0	0	0	845			
28-3S	42657	232.4	53.0	739	1	E	0	0	0	739			
28-3S	45657	21.0	124.8	676	1	I	0	0	676	0			
28-3S	48074	340.9	172.1	987	1	E	1	0	802	185		360	
28-3S	51357	86.0	136.4	948	1	I	0	1	558	390		180	
28-3S	54073	91.1	117.3	787	1	I	0	1	491	296		180	
28-3S	57657	196.0	84.4	817	1	E	0	0	0	817			
28-5S	30641	190.9	117.3	1217	1	E	0	0	0	1217			
28-5S	33640	194.2	73.1	751	1	E	0	0	0	751			
28-5S	36340	179.1	105.3	1174	1	I	0	1	5	1169		180	

28-5S	39640	206.1	79.7	759	1	E	0	0	0		759			
28-5S	42057	173.4	94.4	872	1	I	0	1	36		836		180	
28-5S	45657	252.7	76.3	889	1	E	0	0	0		889			
28-5S	48657	237.5	83.1	866	1	E	0	0	0		866			
28-5S	51657	236.3	74.0	828	1	E	0	0	0		828			
28-5S	54656	224.3	95.2	1079	1	E	0	0	0		1079			
28-5S	57073	154.8	79.9	661	1	I	0	1	143		518		180	
28-8S	30332	149.2	94.3	936	1	I	0	1	192		744		180	
28-8S	33648	179.1	63.1	659	1	I	0	1	5		654		180	
28-8S	36065	83.4	113.0	780	1	I	0	1	603		177		180	
28-8S	39082	79.6	171.3	1362	1	I	0	1	593		769		180	
28-8S	42349	129.4	102.5	801	1	I	0	1	282		519		180	
28-8S	45349	153.7	74.9	686	1	I	0	1	157		529		180	
28-8S	48065	110.0	141.6	1082	1	I	0	1	382		700		180	
28-8S	51664	220.1	86.4	942	1	E	0	0	0		942			
28-8S	54364	193.2	64.4	655	1	E	0	0	0		655			
28-8S	57364	208.2	176.0	1865	1	E	1	0	150		1715		360	
29-1S	30048	58.6	188.2	1340	1	I	0	1	800		540		180	
29-1S	33065	116.9	134.2	1007	1	I	0	1	411		596		180	
29-1S	36349	193.9	111.5	904	1	E	0	0	0		904			
29-1S	39649	258.3	131.3	1063	1	E	1	0	217		846		360	
29-1S	42349	234.1	110.7	1090	1	E	0	0	0		1090			
29-1S	45066	184.7	56.3	825	1	E	0	0	0		825			
29-1S	48081	60.1	114.6	850	1	I	0	0	850		0			
29-1S	51097	62.5	140.0	879	1	I	0	1	719		160		180	
29-1S	54664	224.1	175.0	1159	1	E	1	0	209		950		360	
29-1S	57664	292.3	213.0	1497	1	E	1	0	841		656		360	
29-4S	30336	343.0	224.7	1502	1	E	1	1	1076		175	251	360	180
29-4S	33634	70.7	212.9	1511	1	I	0	1	640		871		180	
29-4S	36050	45.2	208.5	1605	1	I	0	1	782		823		180	
29-4S	39650	122.7	115.4	1040	1	I	0	1	377		663		180	
29-4S	42067	358.8	248.5	2118	0	E	1	1	1321		14	783	360	180
29-4S	45083	324.4	106.8	859	1	E	1	0	445		414		360	
29-4S	48349	14.9	167.0	1362	1	I	0	1	1338		24		180	
29-4S	51665	338.3	197.7	1109	1	E	1	0	838		271		360	
29-4S	54364	28.9	147.4	1030	1	I	0	0	1030		0			
29-4S	57364	16.1	174.1	1368	1	I	0	1	1251		117		180	
29-7S	30332	8.3	206.9	1369	1	I	0	1	1082		287		180	
29-7S	33347	76.5	338.3	1713	1	I	1	1	629	272	812		180	360

29-7S	36063	214.1	100.5	1123	1	E	0	0	0	1123			
29-7S	39346	222.0	77.0	863	1	E	0	0	0	863			
29-7S	42346	193.6	66.8	1033	1	E	0	0	0	1033			
29-7S	45346	61.9	122.0	855	1	I	0	1	815	40		180	
29-7S	48663	87.5	113.0	924	1	I	0	1	690	234		180	
29-7S	51080	331.2	92.2	831	1	E	1	0	502	329		360	
29-7S	54095	300.1	118.0	1058	1	E	1	0	429	629		360	
29-7S	57112	291.8	108.0	958	1	E	1	0	312	646		360	
29-8S	30631	250.2	182.1	1653	1	E	1	0	503	1150		360	
29-8S	33347	213.4	116.0	1096	1	E	0	0	0	1096			
29-8S	36347	234.2	263.4	1701	1	E	1	0	738	963		360	
29-8S	39347	317.5	178.2	1201	1	E	1	0	870	331		360	
29-8S	42647	334.0	153.0	1224	1	E	1	0	918	306		360	
29-8S	45063	270.7	92.5	821	1	E	1	0	25	796		360	
29-8S	48663	323.8	143.8	1039	1	E	1	0	682	357		360	
29-8S	51363	310.1	246.3	2013	1	E	1	1	1384	461	168	360	180
29-8S	54078	265.0	119.4	1145	1	E	1	0	171	974		360	
29-8S	57094	269.4	265.1	1851	1	E	1	0	1062	789		360	
30-1S	30046	230.8	63.5	1124	1	E	0	0	0	1124			
30-1S	33645	176.4	41.8	830	1	I	0	1	27	803		180	
30-1S	36062	293.5	113.2	1771	0	E	1	0	370	1401		360	
30-1S	39078	189.7	58.3	1131	0	E	0	0	0	1131			
30-1S	42644	38.4	203.5	2382	0	I	0	1	965	1417		180	
30-1S	45661	269.7	27.5	630	0	E	0	0	0	630			
30-1S	48661	120.5	80.9	955	1	I	0	1	460	495		180	
30-1S	51361	276.7	30.1	697	1	E	0	0	0	697			
30-1S	54076	107.8	107.1	1276	1	I	0	1	528	748		180	
30-1S	57359	308.5	40.5	868	1	E	0	0	0	868			
30-2S	30047	328.6	151.4	1142	1	E	1	0	750	392		360	
30-2S	33346	323.3	87.7	800	1	E	1	0	343	457		360	
30-2S	36645	326.3	133.0	1005	1	E	1	0	629	376		360	
30-2S	39062	281.7	167.5	1196	0	E	1	0	503	693		360	
30-2S	42078	242.9	57.2	1190	0	E	0	0	0	1190			
30-2S	45645	126.2	62.2	653	1	I	0	1	482	171		180	
30-2S	48062	275.1	68.6	1396	1	E	0	0	0	1396			
30-2S	51362	187.4	84.4	1754	1	E	0	0	0	1754			
30-2S	54361	331.7	62.7	890	1	E	1	0	302	588		360	
30-2S	57078	201.3	51.4	1326	1	E	0	0	0	1326			
30-6S	30639	345.7	190.0	1208	1	E	1	0	938	270		360	

30-6S	33638	313.1	293.2	2327	1	E	1	1	954	623	750	360	180
30-6S	36638	305.8	275.0	1609	1	E	1	1	624	613	372	360	180
30-6S	39055	309.5	136.1	1057	1	E	1	0	597	460		360	
30-6S	42355	318.6	106.5	931	1	E	1	0	457	474		360	
30-6S	45654	312.7	81.1	722	1	E	1	0	166	556		360	
30-6S	48354	250.5	77.8	1391	1	E	0	0	0	1391			
30-6S	51070	106.0	148.3	1721	1	I	0	1	528	1193		180	
30-6S	54369	352.8	169.9	1158	1	E	1	0	1042	116		360	
30-6S	57669	314.3	116.0	930	1	E	1	0	238	692		360	
30-8S	30332	290.5	51.6	896	1	E	0	0	0	896			
30-8S	33347	210.8	110.8	2065	1	E	0	0	0	2065			
30-8S	36064	356.6	111.7	817	1	E	1	0	753	64		360	
30-8S	39081	285.4	48.2	778	1	E	0	0	0	778			
30-8S	42097	208.8	53.0	915	1	E	0	0	0	915			
30-8S	45347	86.2	107.7	916	1	I	0	1	698	218		180	
30-8S	48663	346.5	98.5	811	1	E	1	0	599	212		360	
30-8S	51080	218.2	50.5	1240	1	E	0	0	0	1240			
30-8S	54662	14.7	111.9	791	1	I	0	0	791	0			
30-8S	57662	274.4	44.7	867	1	E	0	0	0	867			
31-1S	30633	274.7	61.2	873	1	E	0	0	0	873			
31-1S	33350	171.2	38.2	755	1	I	0	1	77	678		180	
31-1S	36066	294.5	55.5	1278	1	E	0	0	0	1278			
31-1S	39348	190.5	62.0	1176	1	E	0	0	0	1176			
31-1S	42348	348.7	106.1	994	1	E	1	0	779	215		360	
31-1S	45647	275.6	75.9	1274	1	E	0	0	0	1274			
31-1S	48647	179.7	47.7	826	1	I	0	1	3	823		180	
31-1S	51064	318.9	84.8	1067	1	E	1	0	353	714		360	
31-1S	54664	267.9	156.5	2082	1	E	1	0	581	1501		360	
31-1S	57664	166.1	66.1	968	0	I	0	1	125	843		180	
31-4S	30343	148.7	93.6	1333	1	I	0	1	295	1038		180	
31-4S	33059	325.3	88.7	1073	1	E	1	0	495	578		360	
31-4S	36642	266.8	82.0	1279	1	E	0	0	0	1279			
31-4S	39641	183.8	123.8	2017	1	E	0	0	0	2017			
31-4S	42358	350.6	102.9	999	1	E	1	0	846	153		360	
31-4S	45075	246.7	70.2	984	1	E	0	0	0	984			
31-4S	48359	190.5	114.7	2481	1	E	0	0	0	2481			
31-4S	51076	316.2	36.7	793	1	E	0	0	0	793			
31-4S	54659	260.2	83.8	1288	1	E	0	0	0	1288			
31-4S	57659	167.9	87.7	1117	1	I	0	1	106	1011		180	

31-6S	30047	22.4	142.5	1107	1	I	0	0	1107	0	
31-6S	33647	335.2	77.1	864	1	E	1	0	484	380	360
31-6S	36346	224.6	46.1	676	1	E	0	0	0	676	
31-6S	39346	140.2	96.2	1004	1	I	0	1	288	716	180
31-6S	42646	98.8	89.6	738	1	I	0	1	599	139	180
31-6S	45645	325.8	84.4	974	1	E	1	0	411	563	360
31-6S	48061	203.0	93.4	1538	1	E	0	0	0	1538	
31-6S	51078	56.1	125.8	985	1	I	0	1	951	34	180
31-6S	54095	299.8	145.5	1731	1	E	1	0	694	1037	360
31-6S	57661	257.5	76.0	1046	1	E	0	0	0	1046	
31-8S	30335	136.3	66.5	710	1	I	0	1	240	470	180
31-8S	33052	300.1	32.3	667	1	E	0	0	0	667	
31-8S	36652	214.0	31.3	841	1	E	0	0	0	841	
31-8S	39351	314.5	31.9	857	1	E	0	0	0	857	
31-8S	42650	211.9	34.1	826	1	E	0	0	0	826	
31-8S	45066	311.5	30.8	746	1	E	0	0	0	746	
31-8S	48350	209.9	37.8	757	1	E	0	0	0	757	
31-8S	51366	1.0	84.8	665	0	I	0	0	665	0	
31-8S	54365	268.4	69.8	1260	1	E	0	0	0	1260	
31-8S	57365	159.1	48.9	698	1	I	0	1	177	521	180
32-1S	30346	271.3	75.7	780	1	E	0	0	0	780	
32-1S	33646	266.6	50.5	731	1	E	0	0	0	731	
32-1S	36646	227.0	187.1	1813	1	E	1	0	306	1507	360
32-1S	39646	209.8	128.4	2046	1	E	0	0	0	2046	
32-1S	42063	4.2	136.1	750	1	I	0	0	750	0	
32-1S	45079	275.7	48.4	1036	1	E	0	0	0	1036	
32-1S	48096	186.6	34.8	889	1	E	0	0	0	889	
32-1S	51662	326.1	32.5	830	1	E	0	0	0	830	
32-1S	54077	215.7	63.8	955	1	E	0	0	0	955	
32-1S	57361	183.1	100.6	1018	1	E	0	0	0	1018	
32-3S	30331	248.9	74.1	900	1	E	0	0	0	900	
32-3S	33047	211.8	109.2	1255	1	E	0	0	0	1255	
32-3S	36346	226.1	114.8	1595	1	E	0	0	0	1595	
32-3S	39646	217.4	76.6	792	1	E	0	0	0	792	
32-3S	42063	160.6	52.9	469	1	I	0	1	114	355	180
32-3S	45079	180.9	122.2	1403	1	E	0	0	0	1403	
32-3S	48662	221.8	119.2	1609	1	E	0	0	0	1609	
32-3S	51361	142.2	146.7	1340	1	I	0	1	222	1118	180
32-3S	54361	165.2	68.1	782	1	I	0	1	83	699	180

32-3S	57660	141.2	121.9	1217	1	I	0	1	236	981		180	
32-6S	30337	339.8	107.9	948	1	E	1	0	599	349		360	
32-6S	33337	259.3	50.3	903	0	E	0	0	0	903			
32-6S	36054	150.6	97.4	1511	1	I	0	1	178	1333		180	
32-6S	39654	354.5	151.2	1064	1	E	1	0	956	108		360	
32-6S	42654	324.5	249.5	1677	1	E	1	1	965	420	292	360	180
32-6S	45070	300.0	241.8	1395	0	E	1	1	867	515	13	360	180
32-6S	48353	117.1	124.6	1100	1	I	0	1	339	761		180	
32-6S	51653	138.8	175.1	1857	1	I	0	1	220	1637		180	
32-6S	54669	134.2	159.4	1487	1	I	0	1	204	1283		180	
32-6S	57669	140.0	86.5	706	1	I	0	1	218	488		180	
32-8S	30050	272.4	74.9	1277	1	E	0	0	0	1277			
32-8S	33650	283.5	264.2	1738	1	E	1	1	824	830	84	360	180
32-8S	36350	276.8	288.1	2063	1	E	1	1	832	899	332	360	180
32-8S	39350	275.1	65.4	872	1	E	0	0	0	872			
32-8S	42350	256.9	93.6	955	1	E	0	0	0	955			
32-8S	45350	298.2	153.0	1086	1	E	1	0	585	501		360	
32-8S	48350	334.4	158.2	945	1	E	1	0	722	223		360	
32-8S	51065	7.7	142.3	686	1	I	0	0	686	0			
32-8S	54365	187.8	119.6	1201	1	E	0	0	0	1201			
32-8S	57665	232.0	82.5	878	1	E	0	0	0	878			
33-1S	30045	352.9	116.3	837	1	E	1	0	696	141		360	
33-1S	33645	127.5	88.6	759	1	I	0	1	371	388		180	
33-1S	36062	19.3	113.7	647	1	I	0	0	647	0			
33-1S	39645	165.6	70.9	747	1	I	0	1	83	664		180	
33-1S	42345	82.8	121.0	845	1	I	0	1	591	254		180	
33-1S	45361	82.1	115.0	784	1	I	0	1	595	189		180	
33-1S	48360	63.0	81.4	539	1	I	0	0	539	0			
33-1S	51660	123.1	83.7	648	1	I	0	1	364	284		180	
33-1S	54360	70.3	134.8	936	1	I	0	1	667	269		180	
33-1S	57076	20.0	125.2	740	1	I	0	0	740	0			
33-6S	30643	279.2	157.2	1773	1	E	1	0	540	1233		360	
33-6S	33643	214.9	47.9	679	1	E	0	0	0	679			
33-6S	36643	130.2	112.8	1231	1	I	0	1	361	870		180	
33-6S	39060	329.0	58.9	610	1	E	1	0	182	428		360	
33-6S	42643	356.5	161.6	1197	1	E	1	0	1158	39		360	
33-6S	45059	267.0	62.4	760	1	E	0	0	0	760			
33-6S	48358	247.9	64.2	953	1	E	0	0	0	953			
33-6S	51658	201.7	47.3	594	1	E	0	0	0	594			

33-6S	54358	109.2	109.9	971	1	I	0	1	462	509		180	
33-6S	57658	73.4	144.5	1142	1	I	0	1	712	430		180	
33-7S	30060	197.5	89.4	1244	1	E	0	0	0	1244			
33-7S	33076	131.7	82.4	664	1	I	0	1	277	387		180	
33-7S	36643	189.8	124.4	1370	1	E	0	0	0	1370			
33-7S	39643	173.5	121.0	1344	1	I	0	1	42	1302		180	
33-7S	42343	100.3	134.6	1106	1	I	0	1	485	621		180	
33-7S	45359	67.2	127.8	965	1	I	0	1	830	135		180	
33-7S	48075	46.9	159.5	1054	1	I	0	1	761	293		180	
33-7S	51658	134.5	148.8	1243	1	I	0	1	279	964		180	
33-7S	54358	125.0	182.1	2095	1	I	0	1	326	1770		180	
33-7S	57075	351.6	94.0	692	1	E	1	0	575	117		360	
33-8S	30333	288.9	61.3	931	1	E	0	0	0	931			
33-8S	33333	252.5	73.2	821	1	E	0	0	0	821			
33-8S	36050	221.8	73.1	815	1	E	0	0	0	815			
33-8S	39650	254.3	69.1	921	1	E	0	0	0	921			
33-8S	42650	220.3	89.4	1114	1	E	0	0	0	1114			
33-8S	45649	199.1	59.0	631	1	E	0	0	0	631			
33-8S	48347	165.0	86.5	891	1	I	0	1	89	802		180	
33-8S	51664	190.8	77.4	826	1	E	0	0	0	826			
33-8S	54363	142.5	123.6	1177	1	I	0	1	235	942		180	
33-8S	57662	167.6	91.4	972	1	I	0	1	81	891		180	
34-1S	30636	224.9	53.2	783	1	E	0	0	0	783			
34-1S	33636	142.1	97.7	1016	0	I	0	1	270	746		180	
34-1S	36351	27.6	216.2	1844	0	I	0	1	1097	747		180	
34-1S	39651	15.8	136.9	848	1	I	0	0	848	0			
34-1S	42068	242.8	80.1	1785	1	E	0	0	0	1785			
34-1S	45084	59.5	172.5	1589	1	I	0	1	819	770		180	
34-1S	48351	345.3	201.7	1446	1	E	1	1	1158	218	70	360	180
34-1S	51368	343.8	172.1	1106	1	E	1	0	944	162		360	
34-1S	54368	336.0	179.3	1292	1	E	1	0	1023	269		360	
34-1S	57366	319.6	182.9	1204	1	E	1	0	757	447		360	
34-3S	30640	311.0	30.9	958	1	E	0	0	0	958			
34-3S	33640	190.6	65.9	1136	1	E	0	0	0	1136			
34-3S	36339	347.2	173.5	1260	1	E	1	0	1039	221		360	
34-3S	39056	299.3	130.4	1154	1	E	1	0	476	678		360	
34-3S	42072	238.8	38.1	719	1	E	0	0	0	719			
34-3S	45088	113.0	110.3	1145	1	I	0	1	433	712		180	
34-3S	48105	337.2	170.5	1285	1	E	1	0	911	374		360	

34-3S	51355	338.3	112.8	822	1	E	1	0	580	242	360
34-3S	54355	315.3	159.1	1348	1	E	1	0	816	532	360
34-3S	57370	286.3	179.4	1250	1	E	1	0	418	832	360
34-6S	30333	25.2	151.2	852	1	I	0	0	852	0	
34-6S	33050	36.7	160.1	1020	1	I	0	1	868	152	180
34-6S	36648	172.5	118.1	1082	1	I	0	1	48	1034	180
34-6S	39348	160.6	72.5	638	1	I	0	1	117	521	180
34-6S	42064	136.3	119.1	1034	1	I	0	1	267	767	180
34-6S	45347	165.3	135.7	1350	1	I	0	1	105	1245	180
34-6S	48347	173.5	81.4	811	1	I	0	1	39	772	180
34-6S	51363	156.5	68.6	684	1	I	0	1	168	516	180
34-6S	54080	81.9	120.2	823	1	I	0	1	587	236	180
34-6S	57662	175.0	99.8	1015	1	I	0	1	31	984	180
34-8S	30638	299.2	66.8	906	1	E	1	0	42	864	360
34-8S	33638	251.3	78.9	971	1	E	0	0	0	971	
34-8S	36337	196.5	64.7	790	1	E	0	0	0	790	
34-8S	39353	159.2	91.8	930	1	I	0	1	133	797	180
34-8S	42653	166.4	79.8	786	1	I	0	1	94	692	180
34-8S	45069	73.5	116.7	742	1	I	0	1	637	105	180
34-8S	48652	178.5	136.7	1747	1	I	0	1	9	1738	180
34-8S	51652	106.7	102.4	776	1	I	0	1	466	310	180
34-8S	54669	95.5	119.5	912	1	I	0	1	557	355	180
34-8S	57668	102.6	212.4	1573	1	I	0	1	466	1107	180
35-1S	30339	245.1	155.7	1597	1	E	1	0	291	1306	360
35-1S	33054	190.1	111.9	1399	0	E	0	0	0	1399	
35-1S	36637	209.4	133.6	1411	0	E	0	0	0	1411	
35-1S	39054	142.8	109.3	891	1	I	0	1	215	676	180
35-1S	42354	216.3	252.5	2068	1	E	1	0	723	1345	360
35-1S	45071	198.2	220.7	1914	1	E	1	0	350	1564	360
35-1S	48088	213.3	101.8	1171	1	E	0	0	0	1171	
35-1S	51355	229.4	81.4	862	1	E	0	0	0	862	
35-1S	54670	268.0	120.4	1137	1	E	1	0	189	948	360
35-1S	57370	231.7	132.6	1402	1	E	1	0	26	1376	360
35-3S	30332	285.4	98.2	1177	1	E	1	0	153	1024	360
35-3S	33348	251.8	80.9	932	1	E	0	0	0	932	
35-3S	36648	249.9	79.9	1066	1	E	0	0	0	1066	
35-3S	39348	192.5	116.9	1364	1	E	0	0	0	1364	
35-3S	42348	172.7	91.7	832	1	I	0	1	44	788	180
35-3S	45348	213.0	192.9	1819	1	E	1	0	309	1510	360

35-3S	48665	236.9	85.7	897	1	E	0	0	0	897	
35-3S	51082	181.3	127.1	1445	1	E	0	0	0	1445	
35-3S	54664	218.5	121.3	1552	1	E	0	0	0	1552	
35-3S	57080	94.4	138.2	1065	1	I	0	1	548	517	180
35-6S	30632	44.8	186.7	1290	1	I	0	1	797	493	180
35-6S	33047	349.1	185.6	1093	1	E	1	0	989	104	360
35-6S	36347	116.1	120.1	912	1	I	0	1	378	534	180
35-6S	39063	105.8	107.2	737	1	I	0	1	441	296	180
35-6S	42647	216.8	115.7	1444	1	E	0	0	0	1444	
35-6S	45063	99.8	184.7	1573	1	I	0	1	506	1067	180
35-6S	48080	115.0	114.4	808	1	I	0	1	380	428	180
35-6S	51663	230.4	120.3	1409	1	E	0	0	0	1409	
35-6S	54662	214.1	94.2	998	1	E	0	0	0	998	
35-6S	57362	177.2	89.3	868	1	I	0	1	18	850	180
35-8S	30048	164.9	80.1	780	1	I	0	1	92	688	180
35-8S	33647	216.1	184.7	2005	1	E	1	0	308	1697	360
35-8S	36063	95.2	116.0	903	1	I	0	1	640	263	180
35-8S	39346	174.7	74.9	747	1	I	0	1	34	713	180
35-8S	42346	179.7	94.5	903	1	I	0	1	2	901	180
35-8S	45063	159.7	94.7	815	1	I	0	1	126	689	180
35-8S	48663	245.6	59.5	672	1	E	0	0	0	672	
35-8S	51663	240.1	143.5	1373	1	E	1	0	148	1225	360
35-8S	54078	186.5	88.1	846	1	E	0	0	0	846	
35-8S	57661	254.3	63.2	708	1	E	0	0	0	708	
36-1S	30338	282.3	62.3	1076	1	E	0	0	0	1076	
36-1S	33638	238.5	77.4	1145	1	E	0	0	0	1145	
36-1S	36638	193.1	79.2	1023	1	E	0	0	0	1023	
36-1S	39354	88.5	469.9	1976	1						
36-1S	42354	271.9	56.6	789	1	E	0	0	0	789	
36-1S	45070	212.5	139.0	2063	0	E	0	0	0	2063	
36-1S	48354	197.2	61.3	820	0	E	0	0	0	820	
36-1S	51354	151.6	148.8	1669	1	I	0	1	154	1515	180
36-1S	54371	100.0	92.3	652	1	I	0	1	479	173	180
36-1S	57671	49.6	191.8	1640	1	I	0	1	765	875	180
36-2S	30344	339.2	139.0	1099	1	E	1	0	767	332	360
36-2S	33644	297.8	206.1	1948	1	E	1	0	997	951	360
36-2S	36344	215.0	96.3	1383	1	E	0	0	0	1383	
36-2S	39061	98.4	93.3	706	1	I	0	1	526	180	180
36-2S	42077	341.2	133.8	1047	1	E	1	0	756	291	360

36-2S	45359	291.6	261.8	1986	1	E	1	1	722		1108	156	360	180
36-2S	48658	306.1	297.3	2129	0	E	1	1	887		628	614	360	180
36-2S	51358	302.4	213.1	1333	1	E	1	0	776		557		360	
36-2S	54358	313.8	116.7	871	1	E	1	0	337		534		360	
36-2S	57074	300.0	259.7	1443	1	E	1	1	606		661	176	360	180
36-3S	30050	314.4	229.4	1832	1	E	1	1	988		791	53	360	180
36-3S	33067	268.5	51.2	716	1	E	0	0	0		716			
36-3S	36350	227.5	112.6	2040	1	E	0	0	0		2040			
36-3S	39067	53.6	419.6	1563	1									
36-3S	42084	275.2	57.8	795	1	E	0	0	0		795			
36-3S	45350	234.3	61.2	1186	1	E	0	0	0		1186			
36-3S	48066	51.3	218.0	1725	1	I	0	1	700		1025		180	
36-3S	51366	113.2	112.0	839	0	I	0	1	315		524		180	
36-3S	54366	140.7	181.4	1636	1	I	0	1	167		1469		180	
36-3S	57366	159.5	491.0	3356	0									
36-8S	30645	345.3	224.0	1553	1	E	1	1	966		191	396	360	180
36-8S	33061	273.1	59.7	807	1	E	0	0	0		807			
36-8S	36344	258.4	93.0	1237	1	E	0	0	0		1237			
36-8S	39344	230.9	57.4	761	1	E	0	0	0		761			
36-8S	42061	167.7	222.6	2761	1	I	1	1	74	202	2486		180	360
36-8S	45660	155.6	76.6	792	1	I	0	1	163		629		180	
36-8S	48077	14.6	139.2	811	1	I	0	0	811		0			
36-8S	51360	8.0	220.4	1589	1	I	0	1	1037		552		180	
36-8S	54360	352.0	193.6	1281	1	E	1	1	1106		91	84	360	180
36-8S	57359	300.8	177.0	1581	1	E	1	0	699		882		360	
