# Supplementary material for: Respiratory modulation of cognitive performance during the retrieval process
Source: PLoS One. 2018 Sep 14;13(9):e0204021. doi: 10.1371/journal.pone.0204021 (PMC6138381; doi:10.1371/journal.pone.0204021)
Supplement: S6 Table — (PDF) [file pone.0204021.s008.pdf]

Table S6. Individual test trials in the Phased sessions

									during retrieval (RT)					
name, session	cue (ms)	cue (deg)	retrieval duration (deg)	RT (ms)	accuracy	phase timing	over EI (O360)	over IE (O180)	I duration, 1st (ms)	I duration, 2nd (ms)	E duration, 1st (ms)	E duration, 2nd (ms)	over, 1st	over, 2nd
18-3E	29119	228.6	82.9	1211	1	E	0	0	0		1211			
18-3E	33002	241.2	76.0	1280	1	E	0	0	0		1280			
18-3E	36902	218.5	86.0	1533	1	E	0	0	0		1533			
18-3E	41401	253.7	79.1	1099	1	E	0	0	0		1099			
18-3E	44401	201.6	96.5	1919	1	E	0	0	0		1919			
18-3E	47417	353.3	181.5	1690	1	E	1	0	1557		133		360	
18-3E	50433	25.1	247.1	1629	1	I	0	1	728		901		180	
18-3E	52850	352.9	295.7	1904	1	E	1	1	845		69	990	360	180
18-3E	55267	345.0	282.8	1685	1	E	1	1	731		137	817	360	180
18-3E	58284	93.9	179.0	1412	1	I	0	1	435		977		180	
18-4I	30214	95.5	176.5	1324	1	I	0	1	478		846		180	
18-4I	33230	176.0	132.3	2248	1	I	0	1	20		2228		180	
18-4I	36246	352.5	220.4	1521	1	E	1	1	689		130	702	360	180
18-4I	38962	269.0	57.3	1221	1	E	0	0	0		1221			
18-4I	41678	118.6	798.6	2107	1									
18-4I	44694	237.9	101.6	2269	1	E	0	0	0		2269			
18-4I	47710	59.8	248.8	1476	1	I	0	1	579		897		180	
18-4I	50126	159.9	125.0	1070	1	I	0	1	73		997		180	
18-4I	52842	171.5	123.4	1254	1	I	0	1	46		1208		180	
18-6I	33714	171.9	123.7	1444	1	I	0	1	56		1388		180	
18-6I	36514	94.8	185.4	1596	1	I	0	1	525		1071		180	
18-6I	40113	199.9	110.2	1160	1	E	0	0	0		1160			
18-6I	42613	128.0	145.0	1264	1	I	0	1	331		933		180	
18-6I	45329	121.0	182.1	1635	1	I	0	1	282		1353		180	
18-6I	48345	139.3	135.3	1127	1	I	0	1	221		906		180	
18-6I	51411	196.5	94.7	998	1	E	0	0	0		998			
18-6I	54011	145.1	172.4	1566	1	I	0	1	211		1355		180	
18-6I	57011	148.9	113.5	2247	1	I	0	1	212		2035		180	
18-6I	59727	281.4	43.4	1073	1	E	0	0	0		1073			
18-7E	30829	314.8	268.9	1810	0	E	1	1	868		392	550	360	180
18-7E	33845	319.5	284.3	2877	1	E	1	1	1264		509	1104	360	180
18-7E	36862	252.0	84.1	1453	1	E	0	0	0		1453			

18-7E	40710	237.6	102.2	1808	1	E	0	0	0	1808			
18-7E	43127	57.9	280.4	2589	1	I	0	1	531	2058		180	
18-7E	46144	24.0	207.0	1385	1	I	0	1	950	435		180	
18-7E	48560	351.9	209.7	990	1	E	1	1	777	69	144	360	180
18-7E	50976	81.2	192.2	1430	1	I	0	1	450	980		180	
18-7E	53392	15.5	236.7	1486	1	I	0	1	830	656		180	
18-7E	56408	96.5	153.4	1356	1	I	0	1	476	880		180	
19-3I	34003	163.7	84.2	1120	1	I	0	1	102	1018		180	
19-3I	37503	156.7	74.9	713	1	I	0	1	104	609		180	
19-3I	40802	235.9	95.9	923	1	E	0	0	0	923			
19-3I	43218	259.0	189.5	1197	1	E	1	0	432	765		360	
19-3I	45935	291.7	104.9	812	1	E	1	0	156	656		360	
19-3I	48652	311.3	107.2	782	1	E	1	0	302	480		360	
19-3I	51369	277.8	78.9	1055	1	E	0	0	0	1055			
19-3I	54385	307.9	127.6	846	1	E	1	0	345	501		360	
19-3I	56801	293.1	74.1	681	1	E	1	0	34	647		360	
19-3I	59818	318.5	100.1	713	1	E	1	0	258	455		360	
19-4E	37808	253.3	61.1	848	1	E	0	0	0	848			
19-4E	40607	258.4	80.9	749	1	E	0	0	0	749			
19-4E	43324	311.3	124.3	777	1	E	1	0	355	422		360	
19-4E	45740	271.0	57.5	725	1	E	0	0	0	725			
19-4E	48756	308.6	109.9	717	1	E	1	0	236	481		360	
19-4E	51172	312.7	149.3	994	1	E	1	0	563	431		360	
19-4E	53588	260.8	81.0	993	1	E	0	0	0	993			
19-4E	56404	309.7	255.9	1855	1	E	1	1	1098	396	361	360	180
19-4E	58821	245.5	42.5	599	1	E	0	0	0	599			
19-4E	62404	252.4	57.9	774	1	E	0	0	0	774			
19-6E	33413	274.1	198.9	1595	1	E	1	0	621	974		360	
19-6E	35829	213.8	52.7	705	1	E	0	0	0	705			
19-6E	39611	251.4	148.6	1738	1	E	1	0	255	1483		360	
19-6E	42611	204.7	60.2	901	1	E	0	0	0	901			
19-6E	46511	221.5	47.4	716	1	E	0	0	0	716			
19-6E	50611	249.7	46.4	748	1	E	0	0	0	748			
19-6E	54411	233.8	47.7	857	1	E	0	0	0	857			
19-6E	58011	206.6	62.7	975	1	E	0	0	0	975			
19-6E	62311	248.9	48.4	741	1	E	0	0	0	741			
19-6E	65610	287.3	84.4	746	1	E	1	0	39	707		360	
19-7I	47807	183.8	56.0	998	1	E	0	0	0	998			
19-7I	51807	196.0	90.7	1057	1	E	0	0	0	1057			

19-7I	54524	185.8	58.0	694	1	E	0	0	0	694	
19-7I	57708	186.4	51.2	852	1	E	0	0	0	852	
19-7I	61608	187.0	50.8	773	1	E	0	0	0	773	
19-7I	64707	104.5	158.8	1164	0	I	0	1	339	825	180
19-7I	67907	206.0	70.1	815	1	E	0	0	0	815	
19-7I	70807	205.7	54.4	704	1	E	0	0	0	704	
19-7I	73607	184.0	65.0	737	1	E	0	0	0	737	
19-7I	76324	172.6	71.1	743	1	I	0	1	31	712	180
20-2E	30219	235.9	100.0	1062	1	E	0	0	0	1062	
20-2E	33235	243.0	113.3	1118	1	E	0	0	0	1118	
20-2E	36251	265.0	93.4	889	1	E	0	0	0	889	
20-2E	39268	284.5	179.8	1417	0	E	1	0	636	781	360
20-2E	41985	262.3	197.9	1612	1	E	1	0	618	994	360
20-2E	44802	252.8	174.9	1450	1	E	1	0	401	1049	360
20-2E	47700	257.6	92.4	935	1	E	0	0	0	935	
20-2E	50417	234.8	87.1	925	1	E	0	0	0	925	
20-2E	53901	272.6	246.5	1934	0	E	1	0	944	990	360
20-2E	56400	224.1	465.8	3470	0						
20-3I	43409	145.7	115.8	1077	1	I	0	1	188	889	180
20-3I	46708	188.2	144.8	1740	1	E	0	0	0	1740	
20-3I	49908	182.7	160.5	1913	1	E	0	0	0	1913	
20-3I	52808	136.3	129.4	1207	1	I	0	1	252	955	180
20-3I	55908	142.1	96.6	807	1	I	0	1	225	582	180
20-3I	58708	142.9	144.7	1343	1	I	0	1	205	1138	180
20-3I	61908	178.0	92.2	1007	1	I	0	1	12	995	180
20-3I	64708	134.6	193.3	2111	1	I	0	1	271	1840	180
20-3I	68008	130.9	180.1	1778	1	I	0	1	296	1482	180
20-3I	71108	127.6	185.8	1989	1	I	0	1	315	1674	180
20-5I	32014	183.3	139.9	1802	0	E	0	0	0	1802	
20-5I	35014	126.3	101.6	801	1	I	0	1	308	493	180
20-5I	38014	138.6	88.4	753	1	I	0	1	251	502	180
20-5I	41014	132.9	109.3	980	1	I	0	1	292	688	180
20-5I	43914	93.7	123.4	966	1	I	0	1	570	396	180
20-5I	47513	184.6	166.8	2258	1	E	0	0	0	2258	
20-5I	50713	122.5	163.1	1659	0	I	0	1	387	1272	180
20-5I	54413	180.4	155.9	2149	1	E	0	0	0	2149	
20-5I	57613	128.5	130.9	1164	1	I	0	1	290	874	180
20-5I	60329	77.4	130.1	894	1	I	0	1	590	304	180
20-6E	43712	224.5	121.2	2014	1	E	0	0	0	2014	

20-6E	48411	263.0	293.2	2650	1	E	1	1	1198		1249	203	360	180
20-6E	51911	264.0	66.6	835	1	E	0	0	0		835			
20-6E	55011	221.1	126.4	2364	1	E	0	0	0		2364			
20-6E	59211	209.6	92.8	1346	0	E	0	0	0		1346			
20-6E	63010	215.8	96.7	1253	1	E	0	0	0		1253			
20-6E	67110	267.8	63.1	786	1	E	0	0	0		786			
20-6E	70210	242.2	103.7	1165	1	E	0	0	0		1165			
20-6E	73110	220.7	109.7	1319	1	E	0	0	0		1319			
20-6E	76609	228.6	126.1	1712	1	E	0	0	0		1712			
21-2I	27204	86.1	217.8	1674	1	I	0	1	480		1194		180	
21-2I	30219	144.8	115.8	880	1	I	0	1	195		685		180	
21-2I	33235	212.8	132.1	1192	1	E	0	0	0		1192			
21-2I	35651	181.1	195.7	1676	1	E	1	0	96		1580		360	
21-2I	38668	232.0	243.8	1625	1	E	1	0	608		1017		360	
21-2I	41684	327.5	183.3	1029	1	E	1	0	797		232		360	
21-2I	44700	104.3	155.9	1015	1	I	0	1	408		607		180	
21-2I	47715	222.5	256.0	1525	1	E	1	0	604		921		360	
21-2I	50432	309.8	223.2	1232	1	E	1	0	892		340		360	
21-3E	24216	309.8	168.5	1098	1	E	1	0	682		416		360	
21-3E	26716	323.5	138.3	810	1	E	1	0	544		266		360	
21-3E	29132	341.5	213.2	1209	1	E	1	1	956		136	117	360	180
21-3E	31849	35.2	167.4	968	1	I	0	1	803		165		180	
21-3E	34866	159.1	156.4	1258	1	I	0	1	118		1140		180	
21-3E	37883	224.2	101.0	841	1	E	0	0	0		841			
21-3E	40299	216.3	114.6	948	1	E	0	0	0		948			
21-3E	43016	250.5	152.8	1003	1	E	1	0	233		770		360	
21-3E	45732	315.1	147.8	868	1	E	1	0	543		325		360	
21-5E	25902	313.6	122.8	810	1	E	1	0	441		369		360	
21-5E	28318	302.1	131.8	876	1	E	1	0	398		478		360	
21-5E	31334	23.1	167.0	991	1	I	0	1	911		80		180	
21-5E	34051	70.0	167.1	1050	1	I	0	1	604		446		180	
21-5E	36768	129.3	134.3	963	1	I	0	1	277		686		180	
21-5E	39485	174.2	230.8	1948	1	I	1	1	32	244	1672		180	360
21-5E	42201	185.0	350.4	2205	0	E	1	0	903		1302		360	
21-5E	45217	279.1	328.8	2184	0	E	1	1	1002		642	540	360	180
21-5E	47932	314.9	188.2	1087	0	E	1	0	729		358		360	
21-6I	27319	201.2	208.2	1575	1	E	1	0	271		1304		360	
21-6I	30335	270.9	353.1	2352	1	E	1	1	977		711	664	360	180
21-6I	32752	272.1	271.4	1646	1	E	1	1	925		695	26	360	180

21-6I	35769	13.3	172.9	984	1	I	0	1	938		46		180	
21-6I	38485	82.3	248.9	1798	1	I	0	1	529		1269		180	
21-6I	41202	126.3	272.9	1890	0	I	1	1	288	211	1391		180	360
21-6I	43619	137.4	194.8	1378	1	I	0	1	229		1149		180	
21-6I	46636	239.1	203.5	1334	1	E	1	0	412		922		360	
21-6I	49351	296.5	238.7	1394	1	E	1	0	905		489		360	
22-2E	34600	256.6	81.9	1057	1	E	0	0	0		1057			
22-2E	37399	217.8	94.2	974	1	E	0	0	0		974			
22-2E	40616	257.6	67.9	682	1	E	0	0	0		682			
22-2E	43333	252.2	59.2	579	1	E	0	0	0		579			
22-2E	46349	260.9	63.2	721	1	E	0	0	0		721			
22-2E	49366	255.0	75.6	845	1	E	0	0	0		845			
22-2E	52383	264.6	184.8	1517	1	E	1	0	515		1002		360	
22-2E	55416	287.8	115.3	911	1	E	1	0	246		665		360	
22-2E	58116	273.4	60.5	653	1	E	0	0	0		653			
22-2E	60716	253.3	68.6	662	1	E	0	0	0		662			
22-3I	41821	150.5	128.3	940	1	I	0	1	152		788		180	
22-3I	44538	206.8	79.2	705	1	E	0	0	0		705			
22-3I	46954	193.4	67.1	569	1	E	0	0	0		569			
22-3I	49971	262.4	67.3	601	1	E	0	0	0		601			
22-3I	52988	294.8	78.1	758	1	E	1	0	72		686		360	
22-3I	55405	256.8	65.1	621	1	E	0	0	0		621			
22-3I	58421	273.5	110.0	1079	1	E	1	0	128		951		360	
22-3I	61138	262.2	67.9	650	1	E	0	0	0		650			
22-3I	64155	292.0	124.9	1026	1	E	1	0	322		704		360	
22-3I	67170	347.6	173.8	865	1	E	1	0	769		96		360	
22-5I	35200	193.7	91.0	836	1	E	0	0	0		836			
22-5I	37800	194.4	89.0	824	1	E	0	0	0		824			
22-5I	40217	169.8	81.4	618	1	I	0	1	53		565		180	
22-5I	42934	212.5	98.6	822	0	E	0	0	0		822			
22-5I	45351	202.5	136.6	1341	0	E	0	0	0		1341			
22-5I	48067	201.0	100.3	806	1	E	0	0	0		806			
22-5I	50784	237.1	355.8	2419	1	E	1	1	988		1044	387	360	180
22-5I	53801	314.6	204.3	1042	1	E	1	0	710		332		360	
22-5I	56817	147.1	103.4	720	1	I	0	1	169		551		180	
22-5I	59234	171.8	111.3	1001	1	I	0	1	40		961		180	
22-6E	34915	264.2	53.4	615	1	E	0	0	0		615			
22-6E	37415	221.7	68.3	655	0	E	0	0	0		655			
22-6E	40431	249.6	70.3	772	1	E	0	0	0		772			

22-6E	43414	247.8	107.9	1185	1	E	0	0	0	1185	
22-6E	46414	257.7	104.2	1025	1	E	1	0	11	1014	360
22-6E	49213	257.0	65.7	656	1	E	0	0	0	656	
22-6E	51930	249.6	50.9	501	1	E	0	0	0	501	
22-6E	54946	264.8	109.4	1130	1	E	1	0	76	1054	360
22-6E	57663	252.6	62.1	599	1	E	0	0	0	599	
22-6E	60680	271.1	63.3	700	1	E	0	0	0	700	
23-3E	34201	263.7	139.6	1463	1	E	1	0	239	1224	360
23-3E	37401	260.0	83.4	1024	1	E	0	0	0	1024	
23-3E	40601	262.6	84.3	1076	1	E	0	0	0	1076	
23-3E	43301	222.4	75.2	739	1	E	0	0	0	739	
23-3E	46318	230.0	63.7	692	1	E	0	0	0	692	
23-3E	49900	268.6	76.5	903	1	E	0	0	0	903	
23-3E	52900	251.3	66.3	734	1	E	0	0	0	734	
23-3E	55900	243.7	70.8	770	1	E	0	0	0	770	
23-3E	59300	272.0	63.6	754	1	E	0	0	0	754	
23-3E	61900	227.0	64.3	674	1	E	0	0	0	674	
23-4I	34002	146.6	76.5	642	1	I	0	1	195	447	180
23-4I	37002	147.6	78.3	707	1	I	0	1	205	502	180
23-4I	40102	137.4	122.6	1170	1	I	0	1	287	883	180
23-4I	43002	104.2	85.1	541	1	I	0	1	457	84	180
23-4I	46302	184.4	99.0	1175	1	E	0	0	0	1175	
23-4I	49201	137.3	79.8	633	1	I	0	1	254	379	180
23-4I	51917	101.6	90.1	583	1	I	0	1	478	105	180
23-4I	54934	137.8	78.2	650	1	I	0	1	284	366	180
23-4I	58101	179.8	49.3	541	1	I	0	1	1	540	180
23-4I	61101	169.5	64.2	581	1	I	0	1	63	518	180
23-7E	37403	250.5	95.4	1411	0	E	0	0	0	1411	
23-7E	41103	263.5	64.2	821	1	E	0	0	0	821	
23-7E	44503	254.9	91.0	1313	1	E	0	0	0	1313	
23-7E	47801	249.8	53.3	632	1	E	0	0	0	632	
23-7E	51101	262.0	44.6	537	1	E	0	0	0	537	
23-7E	53701	213.2	121.9	1474	1	E	0	0	0	1474	
23-7E	56901	218.1	73.8	820	1	E	0	0	0	820	
23-7E	59918	227.6	61.5	608	1	E	0	0	0	608	
23-7E	63101	248.9	62.7	677	1	E	0	0	0	677	
23-7E	65900	221.9	66.4	653	1	E	0	0	0	653	
24-3I	56802	104.4	116.3	1180	1	I	0	1	519	661	180
24-3I	61302	152.2	67.3	768	1	I	0	1	193	575	180

24-3I	64902	118.2	95.7	824	1	I	0	1	413	411	180
24-3I	68301	112.8	83.1	703	1	I	0	1	480	223	180
24-3I	72401	156.5	93.8	936	1	I	0	1	165	771	180
24-3I	75001	70.9	109.8	721	1	I	0	1	709	12	180
24-3I	79801	148.0	132.7	1738	1	I	0	1	253	1485	180
24-3I	83401	95.4	96.1	859	1	I	0	1	615	244	180
24-3I	88600	107.1	97.5	1059	1	I	0	1	520	539	180
24-3I	93800	102.0	117.7	1470	1	I	0	1	567	903	180
24-4E	40815	220.4	101.2	1733	1	E	0	0	0	1733	
24-4E	45314	241.0	94.9	1182	1	E	0	0	0	1182	
24-4E	48612	219.2	90.5	1073	1	E	0	0	0	1073	
24-4E	52012	217.9	59.4	728	1	E	0	0	0	728	
24-4E	55712	230.9	52.4	776	1	E	0	0	0	776	
24-4E	60612	234.1	66.6	1241	1	E	0	0	0	1241	
24-4E	64511	200.7	200.2	3249	1	E	1	0	334	2915	360
24-4E	69309	214.3	66.4	801	1	E	0	0	0	801	
24-4E	72809	214.1	60.5	715	1	E	0	0	0	715	
24-4E	76809	248.5	49.3	755	1	E	0	0	0	755	
24-6E	46208	220.9	75.2	1249	1	E	0	0	0	1249	
24-6E	50207	204.3	47.6	754	1	E	0	0	0	754	
24-6E	55007	258.0	186.6	2027	1	E	1	0	715	1312	360
24-6E	58607	250.9	98.0	1060	1	E	0	0	0	1060	
24-6E	61806	252.4	90.8	952	1	E	0	0	0	952	
24-6E	65206	233.7	46.6	915	1	E	0	0	0	915	
24-6E	70005	250.3	67.1	986	1	E	0	0	0	986	
24-6E	73405	205.9	46.3	773	1	E	0	0	0	773	
24-6E	78105	207.9	22.3	780	1	E	0	0	0	780	
24-6E	85304	202.6	63.7	1297	1	E	0	0	0	1297	
24-7I	62012	99.6	99.4	832	1	I	0	1	602	230	180
24-7I	65911	178.2	84.8	1229	1	I	0	1	11	1218	180
24-7I	69011	64.7	175.8	1762	1	I	0	1	798	964	180
24-7I	73510	115.3	87.6	845	1	I	0	1	467	378	180
24-7I	77510	77.3	212.2	2420	1	I	0	1	754	1666	180
24-7I	81710	100.3	99.4	799	1	I	0	1	561	238	180
24-7I	85309	129.5	88.2	964	1	I	0	1	338	626	180
24-7I	89709	161.1	100.0	1223	1	I	0	1	126	1097	180
24-7I	93209	134.0	164.0	1942	1	I	0	1	322	1621	180
24-7I	97109	154.5	80.3	834	1	I	0	1	182	652	180
26-2E	31506	239.9	82.2	885	1	E	0	0	0	885	

26-2E	35005	304.0	266.5	1738	1	E	1	1	1017		485	236	360	180
26-2E	37422	298.4	113.0	804	1	E	1	0	328		476		360	
26-2E	40439	325.9	227.6	1437	1	E	1	1	979		326	132	360	180
26-2E	43155	324.2	99.1	749	1	E	1	0	399		350		360	
26-2E	45572	282.3	269.4	1868	0	E	1	1	1060		708	100	360	180
26-2E	48288	290.9	89.1	733	1	E	1	0	142		591		360	
26-2E	51004	252.1	100.3	1176	1	E	0	0	0		1176			
26-2E	54603	281.4	230.6	1680	0	E	1	0	858		822		360	
26-2E	57020	235.2	77.7	815	1	E	0	0	0		815			
26-3I	32401	141.9	88.0	704	1	I	0	1	224		480		180	
26-3I	35501	181.7	83.7	804	1	E	0	0	0		804			
26-3I	38001	138.7	74.3	548	0	I	0	1	234		314		180	
26-3I	40901	148.6	247.3	2637	0	I	1	1	201	216	2220		180	360
26-3I	44100	129.4	137.5	1122	1	I	0	1	304		818		180	
26-3I	46817	134.0	105.9	759	1	I	0	1	247		512		180	
26-3I	49834	203.6	66.2	572	1	E	0	0	0		572			
26-3I	52851	242.8	137.3	1341	1	E	1	0	120		1221		360	
26-3I	55867	248.5	74.7	784	1	E	0	0	0		784			
26-3I	58283	200.2	70.3	605	1	E	0	0	0		605			
26-5I	31010	169.9	167.1	1608	1	I	0	1	64		1544		180	
26-5I	33909	172.4	236.7	2021	1	I	1	1	47	278	1696		180	360
26-5I	36409	133.7	192.2	1751	1	I	0	1	262		1489		180	
26-5I	39126	99.7	90.6	587	0	I	0	1	498		89		180	
26-5I	42143	169.4	114.0	982	1	I	0	1	60		922		180	
26-5I	45160	208.6	71.4	649	1	E	0	0	0		649			
26-5I	47877	195.3	58.4	564	1	E	0	0	0		564			
26-5I	50610	188.4	66.6	606	1	E	0	0	0		606			
26-5I	53310	186.8	74.3	685	1	E	0	0	0		685			
26-5I	55726	137.8	158.6	1486	1	I	0	1	251		1235		180	
26-6E	34712	270.7	79.3	942	1	E	0	0	0		942			
26-6E	37612	259.1	151.9	1319	1	E	1	0	323		996		360	
26-6E	40512	240.3	85.1	1076	1	E	0	0	0		1076			
26-6E	44111	275.2	163.5	1350	1	E	1	0	442		908		360	
26-6E	46528	226.6	196.9	1825	0	E	1	0	398		1427		360	
26-6E	49545	217.7	72.2	884	1	E	0	0	0		884			
26-6E	53111	233.5	110.3	1527	0	E	0	0	0		1527			
26-6E	56409	223.0	194.1	1775	1	E	1	0	292		1483		360	
26-6E	59609	224.6	40.1	716	1	E	0	0	0		716			
26-6E	63908	234.1	55.9	758	1	E	0	0	0		758			

27-2I	34606	76.4	137.0	956	1	I	0	1	629	327		180	
27-2I	37622	109.4	115.8	848	1	I	0	1	404	444		180	
27-2I	40639	144.2	168.2	1606	1	I	0	1	209	1397		180	
27-2I	43506	129.1	180.1	1632	1	I	0	1	299	1333		180	
27-2I	46805	183.8	85.7	882	1	E	0	0	0	882			
27-2I	49405	131.2	150.3	1278	1	I	0	1	292	986		180	
27-2I	52121	108.7	155.3	1265	1	I	0	1	443	822		180	
27-2I	55303	165.6	91.3	806	1	I	0	1	85	721		180	
27-2I	57719	122.1	98.6	698	1	I	0	1	305	393		180	
27-2I	60736	173.8	141.6	1416	1	I	0	1	35	1381		180	
27-4E	32302	249.0	90.3	1068	1	E	0	0	0	1068			
27-4E	35101	223.5	102.7	1030	1	E	0	0	0	1030			
27-4E	38300	241.1	73.3	841	1	E	0	0	0	841			
27-4E	41100	219.0	109.0	1128	1	E	0	0	0	1128			
27-4E	44700	274.1	80.6	883	1	E	0	0	0	883			
27-4E	47600	253.9	81.7	855	1	E	0	0	0	855			
27-4E	50600	245.4	167.0	1572	1	E	1	0	313	1259		360	
27-4E	53400	224.8	81.6	850	1	E	0	0	0	850			
27-4E	56416	230.8	61.8	676	1	E	0	0	0	676			
27-4E	59700	247.9	80.1	897	1	E	0	0	0	897			
27-6E	30406	250.7	108.9	1573	1	E	0	0	0	1573			
27-6E	33404	216.9	106.6	1203	1	E	0	0	0	1203			
27-6E	37003	257.1	88.9	1134	1	E	0	0	0	1134			
27-6E	40103	230.2	226.1	2555	0	E	1	0	720	1835		360	
27-6E	43803	231.8	174.0	1559	1	E	1	0	275	1284		360	
27-6E	46820	245.0	69.4	697	1	E	0	0	0	697			
27-6E	50103	261.5	132.8	1408	1	E	1	0	212	1196		360	
27-6E	53103	246.6	85.2	885	1	E	0	0	0	885			
27-6E	56202	232.2	117.2	1428	1	E	0	0	0	1428			
27-6E	59902	263.6	334.3	2879	1	E	1	1	1140	1187	552	360	180
27-7I	36316	210.2	125.9	1589	1	E	0	0	0	1589			
27-7I	40116	243.3	58.7	683	1	E	0	0	0	683			
27-7I	43816	279.0	80.1	890	1	E	0	0	0	890			
27-7I	47016	259.9	74.9	986	1	E	0	0	0	986			
27-7I	49916	218.0	91.0	1016	1	E	0	0	0	1016			
27-7I	53716	251.8	75.2	1024	1	E	0	0	0	1024			
27-7I	57415	251.6	94.5	1253	1	E	0	0	0	1253			
27-7I	60715	233.5	77.1	960	1	E	0	0	0	960			
27-7I	64415	275.8	142.7	1299	1	E	1	0	404	895		360	

27-7I	67215	235.8	100.6	1195	1	E	0	0	0	1195			
28-2E	30309	272.0	101.1	1060	1	E	1	0	80	980		360	
28-2E	33108	251.0	244.6	1804	0	E	1	0	703	1101		360	
28-2E	35824	256.5	99.3	885	1	E	0	0	0	885			
28-2E	38840	284.9	97.2	838	1	E	1	0	130	708		360	
28-2E	41557	279.0	127.9	1061	1	E	1	0	285	776		360	
28-2E	44573	299.9	288.6	2031	1	E	1	1	1009	574	448	360	180
28-2E	47589	335.0	114.1	754	1	E	1	0	523	231		360	
28-2E	50006	314.7	123.8	823	1	E	1	0	443	380		360	
28-2E	52422	282.5	88.3	834	1	E	1	0	62	772		360	
28-2E	55304	285.2	78.0	785	1	E	1	0	19	766		360	
28-4I	34228	119.5	113.5	916	1	I	0	1	337	579		180	
28-4I	37612	190.3	83.3	904	1	E	0	0	0	904			
28-4I	40611	189.7	70.1	841	1	E	0	0	0	841			
28-4I	43711	189.4	107.2	1135	1	E	0	0	0	1135			
28-4I	46311	145.4	109.3	973	1	I	0	1	189	784		180	
28-4I	49111	135.9	101.8	842	1	I	0	1	234	608		180	
28-4I	52011	147.7	94.3	787	1	I	0	1	168	619		180	
28-4I	55010	186.7	87.4	921	1	E	0	0	0	921			
28-4I	57427	104.2	103.9	756	1	I	0	1	430	326		180	
28-4I	60444	94.6	183.2	1536	1	I	0	1	448	1088		180	
28-6I	33520	296.1	111.3	1078	1	E	1	0	311	767		360	
28-6I	35937	221.6	78.0	880	1	E	0	0	0	880			
28-6I	38653	189.6	91.4	924	1	E	0	0	0	924			
28-6I	41669	206.1	109.5	1320	1	E	0	0	0	1320			
28-6I	44386	154.1	72.3	643	1	I	0	1	145	498		180	
28-6I	47503	183.1	131.2	1693	1	E	0	0	0	1693			
28-6I	50303	82.4	177.3	1731	0	I	0	1	613	1118		180	
28-6I	54502	178.8	72.2	706	1	I	0	1	7	699		180	
28-6I	57402	185.5	94.5	1076	1	E	0	0	0	1076			
28-6I	60502	186.0	82.6	883	1	E	0	0	0	883			
28-7E	34800	243.8	72.4	864	1	E	0	0	0	864			
28-7E	38100	247.5	65.7	764	1	E	0	0	0	764			
28-7E	40900	220.7	75.5	773	1	E	0	0	0	773			
28-7E	44100	247.4	76.5	835	1	E	0	0	0	835			
28-7E	47516	264.2	53.4	652	1	E	0	0	0	652			
28-7E	50316	222.7	90.4	1030	1	E	0	0	0	1030			
28-7E	53816	252.4	85.8	1012	1	E	0	0	0	1012			
28-7E	56915	242.2	58.8	698	1	E	0	0	0	698			

28-7E	60315	262.8	65.5	743	1	E	0	0	0	743			
28-7E	63515	264.6	66.2	795	1	E	0	0	0	795			
29-2I	35214	124.8	177.5	1488	1	I	0	1	387	1101		180	
29-2I	38014	113.1	180.8	1473	1	I	0	1	469	1004		180	
29-2I	40730	97.1	246.2	2091	1	I	0	1	564	1527		180	
29-2I	43747	85.5	125.2	1201	1	I	0	1	850	351		180	
29-2I	46464	343.5	157.1	1174	1	E	1	0	985	189		360	
29-2I	49180	295.5	112.4	1056	1	E	1	0	349	707		360	
29-2I	51596	216.1	101.0	1114	1	E	0	0	0	1114			
29-2I	54312	174.3	82.3	871	1	I	0	1	37	834		180	
29-2I	57328	133.4	152.5	1727	1	I	0	1	356	1371		180	
29-2I	61111	135.7	108.8	1311	1	I	0	1	358	953		180	
29-3E	33301	226.7	66.5	1125	1	E	0	0	0	1125			
29-3E	37301	206.6	106.2	1674	1	E	0	0	0	1674			
29-3E	41601	236.9	85.0	1075	1	E	0	0	0	1075			
29-3E	44901	212.9	86.9	1128	1	E	0	0	0	1128			
29-3E	48401	216.6	263.0	2364	0	E	1	0	862	1502		360	
29-3E	52300	259.9	72.4	997	1	E	0	0	0	997			
29-3E	55400	292.0	120.9	762	1	E	1	0	307	455		360	
29-3E	58116	311.0	94.0	769	1	E	1	0	314	455		360	
29-3E	60899	300.8	238.8	1953	1	E	1	0	1428	525		360	
29-3E	63316	207.0	110.3	1886	1	E	0	0	0	1886			
29-5E	48107	207.7	129.3	1453	1	E	0	0	0	1453			
29-5E	51906	283.8	109.2	951	1	E	1	0	233	718		360	
29-5E	54605	270.8	234.4	1388	1	E	1	0	690	698		360	
29-5E	57322	292.4	297.2	2229	1	E	1	1	1083	700	446	360	180
29-5E	60339	317.4	120.1	943	1	E	1	0	560	383		360	
29-5E	63055	284.9	294.0	2275	1	E	1	1	1141	739	395	360	180
29-5E	65772	262.5	63.3	642	1	E	0	0	0	642			
29-5E	69106	303.9	98.2	818	1	E	1	0	311	507		360	
29-5E	71706	252.1	79.9	846	1	E	0	0	0	846			
29-5E	75105	279.1	128.4	1139	1	E	1	0	342	797		360	
29-6I	36509	68.6	146.7	1112	1	I	0	1	734	378		180	
29-6I	39609	67.8	133.6	984	1	I	0	1	725	259		180	
29-6I	43009	63.9	141.1	1182	1	I	0	1	900	282		180	
29-6I	46709	93.2	104.0	901	1	I	0	1	714	187		180	
29-6I	50109	97.7	122.2	1048	1	I	0	1	607	441		180	
29-6I	53208	74.1	188.7	1531	1	I	0	1	719	812		180	
29-6I	56225	71.6	227.8	1988	1	I	0	1	806	1182		180	

29-6I	59908	152.8	93.1	874	0	I	0	1	195	679	180
29-6I	63008	155.3	117.1	1017	1	I	0	1	167	850	180
29-6I	65425	82.1	169.0	1393	1	I	0	1	707	686	180
30-3I	53504	113.1	149.2	1247	1	I	0	1	283	964	180
30-3I	56802	110.3	111.9	1200	1	I	0	1	573	627	180
30-3I	61102	144.9	78.0	909	1	I	0	1	254	655	180
30-3I	64602	70.6	129.0	1159	1	I	0	1	774	385	180
30-3I	70102	148.2	119.2	1602	1	I	0	1	257	1345	180
30-3I	73902	109.8	165.7	2310	0	I	0	1	495	1815	180
30-3I	78601	105.6	149.9	1894	0	I	0	1	552	1342	180
30-3I	83101	102.9	159.9	2070	0	I	0	1	561	1509	180
30-3I	87700	105.4	107.6	1217	0	I	0	1	536	681	180
30-3I	92700	99.4	137.2	2259	1	I	0	1	607	1652	180
30-4E	48310	221.8	78.1	1299	1	E	0	0	0	1299	
30-4E	52810	278.9	240.0	1910	1	E	1	0	1067	843	360
30-4E	55908	235.8	65.9	1238	1	E	0	0	0	1238	
30-4E	60607	263.1	272.8	2214	1	E	1	0	1111	1103	360
30-4E	63507	214.3	63.1	1215	0	E	0	0	0	1215	
30-4E	68007	200.0	52.1	970	1	E	0	0	0	970	
30-4E	73307	228.3	64.2	1309	1	E	0	0	0	1309	
30-4E	77706	194.1	32.0	867	1	E	0	0	0	867	
30-4E	84506	246.2	273.2	2632	0	E	1	0	1105	1527	360
30-4E	87706	194.0	37.2	1126	0	E	0	0	0	1126	
30-5E	55000	195.1	53.4	1301	1	E	0	0	0	1301	
30-5E	61199	240.5	53.5	828	1	E	0	0	0	828	
30-5E	65299	239.1	70.3	1119	1	E	0	0	0	1119	
30-5E	69499	234.9	90.8	1503	1	E	0	0	0	1503	
30-5E	73499	214.8	51.1	949	1	E	0	0	0	949	
30-5E	77914	199.0	43.9	901	1	E	0	0	0	901	
30-5E	83514	228.5	41.7	761	1	E	0	0	0	761	
30-5E	88214	229.5	53.4	1081	1	E	0	0	0	1081	
30-5E	92614	202.5	40.7	790	1	E	0	0	0	790	
30-5E	97313	194.3	35.7	872	1	E	0	0	0	872	
30-7I	49106	66.5	129.4	1074	1	I	0	1	794	280	180
30-7I	53605	75.4	96.8	686	1	I	0	0	686	0	
30-7I	58105	137.7	91.7	1210	1	I	0	1	319	891	180
30-7I	62505	186.1	67.8	849	1	E	0	0	0	849	
30-7I	65222	75.8	113.7	868	1	I	0	1	743	125	180
30-7I	68805	64.1	170.5	1898	1	I	0	1	858	1040	180

30-7I	74005	108.8	92.3	1048	1	I	0	1	595	453	180
30-7I	78903	55.9	144.9	1345	0	I	0	1	970	375	180
30-7I	84203	133.9	145.9	2379	1	I	0	1	372	2007	180
30-7I	89303	149.8	51.7	948	1	I	0	1	223	725	180
31-2I	41701	97.8	95.0	883	1	I	0	1	714	169	180
31-2I	45601	96.8	99.0	903	1	I	0	1	700	203	180
31-2I	49400	103.7	120.8	1207	0	I	0	1	579	628	180
31-2I	53700	144.8	129.1	1768	0	I	0	1	287	1481	180
31-2I	57400	73.7	112.5	919	1	I	0	1	830	89	180
31-2I	61998	144.0	104.5	1279	0	I	0	1	288	991	180
31-2I	65397	65.4	171.6	1830	0	I	0	1	891	939	180
31-2I	69797	67.4	210.1	2232	0	I	0	1	903	1329	180
31-2I	73997	107.6	104.0	1019	1	I	0	1	569	450	180
31-2I	77697	75.9	109.6	841	1	I	0	1	773	68	180
31-3E	52202	200.8	57.2	1374	1	E	0	0	0	1374	
31-3E	57702	200.9	44.6	878	1	E	0	0	0	878	
31-3E	62901	213.9	57.0	1132	1	E	0	0	0	1132	
31-3E	67601	204.7	75.7	1154	1	E	0	0	0	1154	
31-3E	72201	216.0	225.3	3351	0	E	1	0	688	2663	360
31-3E	77400	256.7	77.7	1025	1	E	0	0	0	1025	
31-3E	80700	207.7	108.4	1557	1	E	0	0	0	1557	
31-3E	84799	205.5	127.4	1744	1	E	0	0	0	1744	
31-3E	89199	229.4	67.2	935	1	E	0	0	0	935	
31-3E	93099	222.9	98.1	1577	1	E	0	0	0	1577	
31-5I	39709	104.7	112.4	1132	1	I	0	1	657	475	180
31-5I	43208	70.2	110.2	839	1	I	0	1	834	5	180
31-5I	47408	92.8	119.0	1131	1	I	0	1	631	500	180
31-5I	52008	150.5	83.7	843	1	I	0	1	223	620	180
31-5I	55408	144.2	65.0	660	1	I	0	1	278	382	180
31-5I	58508	63.2	119.8	906	1	I	0	1	866	40	180
31-5I	62607	117.0	147.3	1429	1	I	0	1	456	973	180
31-5I	66407	150.7	65.9	719	1	I	0	1	246	473	180
31-5I	69807	115.6	76.5	610	1	I	0	1	462	148	180
31-5I	73007	74.5	163.8	1543	1	I	0	1	756	787	180
31-7E	45914	239.1	91.6	1583	1	E	0	0	0	1583	
31-7E	50814	237.8	99.4	1888	1	E	0	0	0	1888	
31-7E	55513	226.7	61.7	938	1	E	0	0	0	938	
31-7E	60013	249.6	61.7	882	1	E	0	0	0	882	
31-7E	64112	245.6	89.7	1416	1	E	0	0	0	1416	

31-7E	67911	203.0	56.0	838	1	E	0	0	0	838			
31-7E	72411	225.9	94.5	1415	1	E	0	0	0	1415			
31-7E	76311	209.3	114.8	1612	1	E	0	0	0	1612			
31-7E	80411	204.5	51.0	792	1	E	0	0	0	792			
31-7E	85110	234.0	99.4	1385	0	E	0	0	0	1385			
32-2E	32003	247.1	49.4	743	1	E	0	0	0	743			
32-2E	35603	237.4	73.8	929	1	E	0	0	0	929			
32-2E	39303	241.2	61.0	1047	1	E	0	0	0	1047			
32-2E	42803	202.8	86.1	1194	1	E	0	0	0	1194			
32-2E	46803	234.3	62.8	837	1	E	0	0	0	837			
32-2E	50703	257.6	50.2	649	1	E	0	0	0	649			
32-2E	54202	236.5	78.6	1308	1	E	0	0	0	1308			
32-2E	58202	252.2	67.0	892	1	E	0	0	0	892			
32-2E	61302	221.2	103.8	1662	1	E	0	0	0	1662			
32-2E	65402	241.7	78.3	901	1	E	0	0	0	901			
32-4I	36210	85.6	143.3	1138	1	I	0	1	553	585		180	
32-4I	39409	86.1	167.0	1208	1	I	0	1	536	672		180	
32-4I	42709	200.0	82.2	716	1	E	0	0	0	716			
32-4I	45125	204.4	105.1	1917	1	E	0	0	0	1917			
32-4I	48408	78.4	118.0	750	1	I	0	1	577	173		180	
32-4I	51424	102.1	127.6	750	1	I	0	1	407	343		180	
32-4I	54440	247.7	65.2	857	1	E	0	0	0	857			
32-4I	57157	191.7	57.6	791	1	E	0	0	0	791			
32-4I	60607	185.3	106.3	1775	1	E	0	0	0	1775			
32-4I	64307	130.3	156.3	1729	1	I	0	1	298	1431		180	
32-5E	28726	230.6	74.0	851	1	E	0	0	0	851			
32-5E	32309	246.4	67.7	1024	1	E	0	0	0	1024			
32-5E	35809	234.1	94.9	1324	1	E	0	0	0	1324			
32-5E	39009	228.7	355.0	2383	0	E	1	1	737	1109	537	360	180
32-5E	42026	275.2	64.6	794	1	E	0	0	0	794			
32-5E	46109	203.7	23.1	1054	1	E	0	0	0	1054			
32-5E	54408	195.0	33.5	1573	1	E	0	0	0	1573			
32-5E	64507	236.5	76.3	1494	0	E	0	0	0	1494			
32-5E	68907	261.1	77.8	905	1	E	0	0	0	905			
32-5E	71907	234.8	99.9	1409	1	E	0	0	0	1409			
32-7I	51102	200.1	100.7	1100	1	E	0	0	0	1100			
32-7I	53519	111.5	91.6	843	1	I	0	1	412	431		180	
32-7I	58502	188.7	55.3	741	1	E	0	0	0	741			
32-7I	62002	197.3	61.9	847	1	E	0	0	0	847			

32-7I	65302	182.8	66.2	769	1	E	0	0	0	769	
32-7I	68018	111.8	110.6	890	1	I	0	1	401	489	180
32-7I	71401	167.3	63.8	645	1	I	0	1	69	576	180
32-7I	74118	111.2	121.9	1022	1	I	0	1	382	640	180
32-7I	77201	99.6	112.1	1091	1	I	0	1	432	659	180
32-7I	82500	54.2	73.7	1532	1	I	0	0	1532	0	
33-2I	38611	121.6	97.3	796	1	I	0	1	354	442	180
33-2I	41811	124.7	65.6	562	1	I	0	1	356	206	180
33-2I	47011	142.7	66.7	666	1	I	0	1	323	343	180
33-2I	50010	84.3	106.1	771	1	I	0	1	655	116	180
33-2I	53310	93.0	109.0	807	1	I	0	1	590	217	180
33-2I	56327	96.5	92.8	651	1	I	0	1	563	88	180
33-2I	59344	121.2	100.0	776	1	I	0	1	370	406	180
33-2I	62510	162.7	112.3	1129	1	I	0	1	109	1020	180
33-2I	65010	77.7	138.8	973	1	I	0	1	602	371	180
33-2I	68308	120.7	110.3	1079	1	I	0	1	425	654	180
33-3E	35108	230.8	93.8	1235	1	E	0	0	0	1235	
33-3E	39008	265.2	66.7	803	1	E	0	0	0	803	
33-3E	42608	258.4	56.6	707	1	E	0	0	0	707	
33-3E	45608	215.5	69.9	926	1	E	0	0	0	926	
33-3E	49807	250.4	78.4	1177	1	E	0	0	0	1177	
33-3E	53407	231.3	116.3	1608	1	E	0	0	0	1608	
33-3E	57207	242.1	58.2	676	1	E	0	0	0	676	
33-3E	60607	248.9	67.0	769	1	E	0	0	0	769	
33-3E	64207	274.5	70.9	812	1	E	0	0	0	812	
33-3E	66906	219.8	94.2	1236	1	E	0	0	0	1236	
33-4I	36612	160.5	99.3	1174	1	I	0	1	118	1056	180
33-4I	39611	78.2	138.6	1115	1	I	0	1	654	461	180
33-4I	43510	154.5	61.4	701	1	I	0	1	163	538	180
33-4I	47109	114.4	86.7	690	1	I	0	1	423	267	180
33-4I	50708	132.7	78.7	755	1	I	0	1	319	436	180
33-4I	54008	76.5	113.0	740	1	I	0	1	659	81	180
33-4I	57108	137.0	91.1	756	1	I	0	1	283	473	180
33-4I	59824	109.7	104.7	772	1	I	0	1	424	348	180
33-4I	62840	122.9	95.3	817	1	I	0	1	359	458	180
33-4I	65856	82.6	118.4	826	1	I	0	1	585	241	180
33-5E	33303	230.6	72.0	931	1	E	0	0	0	931	
33-5E	36603	219.5	60.5	736	1	E	0	0	0	736	
33-5E	40602	253.9	120.1	1514	1	E	1	0	92	1422	360

33-5E	44002	246.2	62.9	755	1	E	0	0	0	755	
33-5E	46902	211.7	65.9	723	1	E	0	0	0	723	
33-5E	50701	258.0	256.3	2245	0	E	1	0	960	1285	360
33-5E	53801	239.7	65.1	758	1	E	0	0	0	758	
33-5E	56901	221.3	64.6	804	1	E	0	0	0	804	
33-5E	60301	220.6	50.2	634	1	E	0	0	0	634	
33-5E	64601	244.9	57.7	870	1	E	0	0	0	870	
34-2I	37010	67.3	141.5	992	1	I	0	1	732	260	180
34-2I	40110	115.3	143.6	1272	1	I	0	1	418	854	180
34-2I	43210	114.1	101.6	768	1	I	0	1	423	345	180
34-2I	46409	155.2	109.8	959	1	I	0	1	166	793	180
34-2I	48825	86.6	198.4	1780	1	I	0	1	615	1165	180
34-2I	52308	136.8	168.4	1545	1	I	0	1	275	1270	180
34-2I	55208	133.0	135.4	1336	1	I	0	1	282	1054	180
34-2I	58406	121.5	106.8	865	1	I	0	1	372	493	180
34-2I	61706	165.6	145.2	1556	1	I	0	1	95	1461	180
34-2I	64806	153.5	86.0	829	1	I	0	1	172	657	180
34-4E	53106	250.7	59.7	865	1	E	0	0	0	865	
34-4E	56905	257.6	70.5	917	1	E	0	0	0	917	
34-4E	60005	244.1	74.7	827	1	E	0	0	0	827	
34-4E	63505	261.2	61.0	780	1	E	0	0	0	780	
34-4E	66305	216.2	69.6	782	1	E	0	0	0	782	
34-4E	69805	236.3	87.5	1123	1	E	0	0	0	1123	
34-4E	73305	229.3	54.6	769	1	E	0	0	0	769	
34-4E	76905	205.7	90.1	1405	1	E	0	0	0	1405	
34-4E	81004	211.8	77.4	997	1	E	0	0	0	997	
34-4E	84904	220.0	38.8	722	1	E	0	0	0	722	
34-5I	36006	151.6	141.2	1466	1	I	0	1	203	1263	180
34-5I	39306	144.5	67.5	741	1	I	0	1	266	475	180
34-5I	43106	118.7	184.4	1664	1	I	0	1	445	1219	180
34-5I	46106	117.4	163.4	1491	1	I	0	1	412	1079	180
34-5I	49206	117.9	96.3	772	1	I	0	1	401	371	180
34-5I	52306	119.4	91.8	737	1	I	0	1	380	357	180
34-5I	55606	127.5	150.1	1344	1	I	0	1	354	990	180
34-5I	58321	86.9	163.1	1195	1	I	0	1	574	621	180
34-5I	61338	147.6	104.9	1049	1	I	0	1	186	863	180
34-5I	64354	89.2	154.7	1456	1	I	0	1	700	756	180
34-7E	38903	253.5	74.7	814	1	E	0	0	0	814	
34-7E	42203	256.3	66.1	835	1	E	0	0	0	835	

34-7E	45703	265.1	69.4	880	1	E	0	0	0	880	
34-7E	48503	221.5	100.6	1149	1	E	0	0	0	1149	
34-7E	52203	259.4	66.5	840	1	E	0	0	0	840	
34-7E	55303	242.3	80.7	958	1	E	0	0	0	958	
34-7E	58401	227.0	88.9	997	1	E	0	0	0	997	
34-7E	62001	260.0	61.8	762	1	E	0	0	0	762	
34-7E	64701	212.7	100.8	1241	1	E	0	0	0	1241	
34-7E	68701	257.4	65.1	844	1	E	0	0	0	844	
35-2E	30213	239.5	77.5	938	1	E	0	0	0	938	
35-2E	33812	265.9	191.6	1775	1	E	1	0	662	1113	360
35-2E	36812	244.2	103.1	1070	1	E	0	0	0	1070	
35-2E	39528	217.2	103.0	1114	1	E	0	0	0	1114	
35-2E	42711	219.3	86.9	929	1	E	0	0	0	929	
35-2E	46311	266.5	62.7	741	1	E	0	0	0	741	
35-2E	49511	275.1	245.5	1996	0	E	1	0	1094	902	360
35-2E	52609	294.1	175.5	1214	0	E	1	0	653	561	360
35-2E	55026	252.1	101.3	1100	1	E	0	0	0	1100	
35-2E	58309	262.6	72.5	877	1	E	0	0	0	877	
35-4I	30402	109.0	107.0	779	1	I	0	1	463	316	180
35-4I	33117	115.5	164.3	1303	1	I	0	1	374	929	180
35-4I	36134	161.9	110.8	1017	1	I	0	1	108	909	180
35-4I	39150	189.3	114.0	1105	1	E	0	0	0	1105	
35-4I	41866	172.8	88.6	828	1	I	0	1	44	784	180
35-4I	44699	182.8	94.8	861	1	E	0	0	0	861	
35-4I	47399	190.4	85.0	838	1	E	0	0	0	838	
35-4I	49816	137.8	101.3	761	1	I	0	1	228	533	180
35-4I	52533	159.2	131.1	1189	1	I	0	1	113	1076	180
35-4I	55515	183.2	101.3	944	1	E	0	0	0	944	
35-5E	30222	242.4	88.9	1172	1	E	0	0	0	1172	
35-5E	33504	231.5	90.6	1114	1	E	0	0	0	1114	
35-5E	36604	214.8	183.7	1877	1	E	1	0	255	1622	360
35-5E	40404	259.3	66.4	826	1	E	0	0	0	826	
35-5E	43804	261.0	174.3	1761	1	E	1	0	509	1252	360
35-5E	46704	220.9	85.4	900	1	E	0	0	0	900	
35-5E	50004	244.1	61.0	742	1	E	0	0	0	742	
35-5E	52904	214.6	77.8	844	1	E	0	0	0	844	
35-5E	55920	216.5	85.2	947	1	E	0	0	0	947	
35-5E	59003	220.3	158.8	1565	1	E	1	0	134	1431	360
35-7I	30607	159.4	113.7	1112	1	I	0	1	153	959	180

35-7I	33406	130.3	154.8	1457	1	I	0	1	302	1155		180	
35-7I	36122	69.1	141.8	989	1	I	0	1	702	287		180	
35-7I	39606	183.9	121.3	1273	1	E	0	0	0	1273			
35-7I	42606	163.7	75.8	781	1	I	0	1	115	666		180	
35-7I	45506	125.0	123.0	1059	1	I	0	1	338	721		180	
35-7I	48806	171.4	100.9	929	1	I	0	1	53	876		180	
35-7I	51605	182.1	88.7	905	1	E	0	0	0	905			
35-7I	54205	130.5	109.4	901	1	I	0	1	298	603		180	
35-7I	56921	103.2	199.7	1768	1	I	0	1	450	1318		180	
36-4E	35903	244.3	95.4	1124	1	E	0	0	0	1124			
36-4E	39102	234.5	85.7	1158	1	E	0	0	0	1158			
36-4E	42902	289.3	270.5	1727	1	E	1	1	805	672	250	360	180
36-4E	45318	254.3	66.5	841	1	E	0	0	0	841			
36-4E	48101	215.2	137.6	1599	1	E	0	0	0	1599			
36-4E	51117	207.1	63.2	824	1	E	0	0	0	824			
36-4E	54800	228.1	80.1	1040	0	E	0	0	0	1040			
36-4E	57999	218.4	76.8	942	1	E	0	0	0	942			
36-4E	61298	211.0	132.0	1894	1	E	0	0	0	1894			
36-4E	64897	210.6	82.5	1066	1	E	0	0	0	1066			
36-5I	31302	97.7	175.8	1732	1	I	0	1	467	1265		180	
36-5I	35001	140.9	108.2	1112	1	I	0	1	222	890		180	
36-5I	38600	181.6	78.1	962	1	E	0	0	0	962			
36-5I	41900	167.6	131.3	1614	1	I	0	1	82	1532		180	
36-5I	45399	170.5	63.9	769	1	I	0	1	61	708		180	
36-5I	48299	91.2	197.2	2102	0	I	0	1	483	1619		180	
36-5I	52199	117.2	186.1	2121	0	I	0	1	390	1731		180	
36-5I	55899	128.5	447.0	1955	0								
36-5I	58616	270.9	61.0	839	1	E	0	0	0	839			
36-5I	61333	225.1	96.3	1385	1	E	0	0	0	1385			
36-6I	36705	76.2	198.2	1834	1	I	0	1	633	1201		180	
36-6I	40705	169.3	145.1	1994	1	I	0	1	68	1927		180	
36-6I	44404	150.6	70.7	789	1	I	0	1	205	584		180	
36-6I	47604	75.2	166.7	1442	1	I	0	1	624	818		180	
36-6I	51104	83.3	107.8	714	1	I	0	1	578	136		180	
36-6I	54704	138.0	97.1	901	1	I	0	1	247	654		180	
36-6I	58204	181.3	102.2	1217	1	E	0	0	0	1217			
36-6I	61103	136.4	187.9	2075	0	I	0	1	247	1828		180	
36-6I	64402	124.6	105.8	922	1	I	0	1	343	579		180	
36-6I	67902	182.7	53.5	645	1	E	0	0	0	645			

36-7E	35008	208.5	75.4	1084	1	E	0	0	0	1084	
36-7E	39308	245.2	101.1	1603	1	E	0	0	0	1603	
36-7E	42807	243.7	230.5	1718	1	E	1	0	424	1294	360
36-7E	45806	247.8	40.9	627	0	E	0	0	0	627	
36-7E	49606	248.3	55.8	830	1	E	0	0	0	830	
36-7E	52706	205.6	87.6	1348	0	E	0	0	0	1348	
36-7E	56506	209.1	65.5	885	0	E	0	0	0	885	
36-7E	60706	268.2	254.7	1739	1	E	1	0	657	1082	360
36-7E	63123	233.4	181.9	1732	1	E	1	0	289	1443	360
36-7E	66138	232.2	172.8	1832	1	E	1	0	282	1551	360
